# Phylogenetic revision of Gymnotidae (Teleostei: Gymnotiformes), with descriptions of six subgenera

**DOI:** 10.1371/journal.pone.0224599

**Published:** 2019-11-07

**Authors:** Jack M. Craig, Lesley Y. Kim, Victor A. Tagliacollo, James S. Albert

**Affiliations:** 1 Department of Biology, University of Louisiana at Lafayette, Lafayette, LA, United States of America; 2 Museu de Zoologia da Universidade de São Paulo, São Paulo, São Paulo, Brazil; Pontificia Universidade Catolica do Rio Grande do Sul, BRAZIL

## Abstract

The diversity of gymnotid electric fishes has been intensely studied over the past 25 years, with 35 species named since 1994, compared to 11 species in the previous 236 years. Substantial effort has also been applied in recent years to documenting gymnotid interrelationships, with seven systematic studies published using morphological and molecular datasets. Nevertheless, until now, all gymnotids have been assigned to one of just two supraspecific taxa, the subfamily Electrophorinae with one genus *Electrophorus* and three valid species and the subfamily Gymnotine also with one genus *Gymnotus* and 43 valid species. This simple classification has obscured the substantial phenotypic and lineage diversity within the subfamily Gymnotine and hampered ecological and evolutionary studies of gymnotid biology. Here we present the most well-resolved and taxon-complete phylogeny of the Gymnotidae to date, including materials from all but one of the valid species. This phylogeny was constructed using a five-gene molecular dataset and a 115-character morphological dataset, enabling the inclusion of several species for which molecular data are still lacking. This phylogeny was time-calibrated using biogeographical priors in the absence of a fossil record. The tree topology is similar to those of previous studies, recovering all the major clades previously recognized with informal names. We propose a new gymnotid classification including two subfamilies (Electrophorinae and Gymnotinae) and six subgenera within the genus *Gymnotus*. Each subgenus exhibits a distinctive biogeographic distribution, within which most species have allopatric distributions and the subgenera are diverged from one another by an estimated 5–35 million years. We further provide robust taxonomic diagnoses, descriptions and identification keys to all gymnotid subgenera and all but four species. This new taxonomy more equitably partitions species diversity among supra-specific taxa, employing the previously vacant subgenus and subfamily ranks. This new taxonomy renders known gymnotid diversity more accessible to study by highlighting the deep divergences (chronological, geographical, genetic and morphological) among its several clades.

## Introduction

The family Gymnotidae Rafinesque is represented by 46 valid species and seven valid subspecies [1], with at least two additional species currently known and being described elsewhere (Table 1). Despite its relative species richness, the family Gymnotidae is currently divided into only two supraspecific (higher) taxa, one of which is the subfamily Electrophorinae with a single genus (*Electrophorus*) and three valid species and the other the subfamily Gymnotinae also with a single genus (*Gymnotus*) and 43 valid species. The geographic distributions of *Gymnotus* species span two continents, from the Rio Motagua in Guatemala (*G*. *cylindricus* LaMonte, at 15°N) to the Rio Salado in Argentina (*G*. *carapo australis* Craig *et al*., at 35°S) [2] (Table 2). *Gymnotus* species exhibit a variety of phenotypes, ranging from meter-long floodplain piscivores (e.g. *G*. *inaequilabiatus*) to small-bodied forms of 15 cm (e.g. *G*. *coropinae*) that inhabit in small forest streams and consume small benthic animals [3–5].

**Table 1:** List of the 43 valid and two undescribed gymnotine species, their name–bearing types (asterisks denote syntypes, double asterisks denote neotypes, all others are holotypes) and the geographic coordinates and localities of these type localities.

Subgenus	Species	Author(s)	Year	Type(s)
*Gymnotus*	*carapo*	Linnaeus/Artedi	1758	NRM 764* & 8224*, UUZM 56*
*Gymnotus*	*bahianus*	Campos-da-Paz	1996	MNRJ 12316
*Gymnotus*	*sylvius*	Albert *et*. *al*.	1999	LGP 0925.1
*Gymnotus*	*mamiraua*	Albert & Crampton	2001	INPA 13503
*Gymnotus*	*arapaima*	Albert & Crampton	2001	INPA 13505
*Gymnotus*	*diamantinensis*	Campos-da-Paz	2002	MZUSP 57505
*Gymnotus*	*ucamara*	Crampton *et*. *al*.	2003	UF 126182
*Gymnotus*	*choco*	Albert & Crampton	2003	ICNMHN 6621
*Gymnotus*	*ardilai*	Maldonado-Ocampo & Albert	2004	IAvHP 3477
*Gymnotus*	*pantanal*	Fernandes *et*. *al*.	2005	MZUSP 67874
*Gymnotus*	*curupira*	Crampton *et*. *al*.	2005	MZUSP 60607
*Gymnotus*	*obscurus*	Crampton *et*. *al*.	2005	BMNH 1998.3.12.19
*Gymnotus*	*varzea*	Crampton *et*. *al*.	2005	MZUSP 60601
*Gymnotus*	*chimarrao*	Cognato *et*. *al*.	2007	UFRGS 6774
*Gymnotus*	*chaviro*	Maxime & Albert	2009	MUSM 33715
*Gymnotus*	*omarorum*	Richer-de-Forges *et*. *al*.	2009	ZVC-P 6480
*Gymnotus*	*interruptus*	Rangel-Pereira	2012	UFRJ 8218
*Gymnotus*	*capanema*	Milhomem *et*. *al*.	2012	MPEG 15170
*Gymnotus*	*cuia*	Craig *et*. *al*.	2017	UFRGS 23700
*Gymnotus*	*eyra*	Craig *et*. *al*.	2017	MUSM 60276
*Gymnotus*	*riberalta*	Craig *et*. *al*.	2017	CBF 10248
*Gymnotus*	*darwini *	de Santana & Campos-da-Paz	2019	MNRJ 51333
*Lamontianus*	*anguillaris*	Hoedeman	1962	ZMA 100.338
*Lamontianus*	*cataniapo*	Mago-Leccia	1994	MBUCV-V 14736
*Lamontianus*	*pedanopterus*	Mago-Leccia	1994	MBUCV-V 14738
*Lamontianus*	*tiquie*	Maxime *et*. *al*.	2011	MZUSP 104507
*Lamontianus*	*n*. *sp*. *ARAP*	Kim *et*. *al*.	in prep	ZUEC 16727
*Lamontianus*	*n*. *sp*. *ARIP*	Kim *et*. *al*.	in prep	INPA 6390
*Pantherus*	*pantherinus*	Steindachner	1908	NMW 76443*
*Pantherus*	*capitimaculatus*	Rangel-Pereira	2014	UFRJ 9785
*Pantherus*	*refugio*	Giora & Malabarba	2016	UFRGS 8752
*Tigre*	*inaequilabiatus*	Valenciennes	1847	MNHN 4615**
*Tigre*	*esmeraldas*	Albert & Crampton	2003	MCZ 58729
*Tigre*	*henni*	Albert & Crampton	2003	CAS 47290
*Tigre*	*paraguensis*	Albert & Crampton	2003	UMMZ 206155
*Tigre*	*tigre*	Albert & Crampton	2003	UF 25552
*Tigrinus*	*coatesi*	LaMonte	1935	AMNH 12624
*Tigrinus*	*coropinae*	Hoedeman	1962	ZMA 100.185
*Tigrinus*	*stenoleucas*	Mago-Leccia	1994	MBUCV-V 6218
*Tigrinus*	*jonasi*	Albert & Crampton	2001	INPA 13507
*Tigrinus*	*melanopleura*	Albert & Crampton	2001	INPA 9966
*Tigrinus*	*onca*	Albert & Crampton	2001	INPA 11512
*Tigrinus*	*javari*	Albert & Crampton	2003	UMMZ 224599
*Tijax*	*cylindricus*	LaMonte	1935	AMNH 1358
*Tijax*	*maculosus*	Albert & Miller	1995	UMMZ 230830
*Tijax*	*panamensis*	Albert & Crampton	2003	CAS 72209

**Table 2:** Type localities of all valid gymnotine species. In cases where geographical coordinates were not included in the original description, estimates are provided indicated by “~”.

Subgenus Species		Coordinates	Locality
*Gymnotus*	*carapo*	~5°47’57”N, 55°10’33”W	Suriname, near Paramaribo
*Gymnotus*	*bahianus*	14°49'S, 39°02'W	Brazil, Bahia, Rio Almada basin
*Gymnotus*	*sylvius*	24°32'50"S, 47°26'13"W	Brazil, São Paulo State, Rio Ribeira de Iguape
*Gymnotus*	*mamiraua*	03°02'36"S, 64°51'02"W	Brazil, Amazonas, Cano do Lago Rato, Mamirauá Reserve
*Gymnotus*	*arapaima*	03°02'S, 64°51'W	Brazil, Amazonas, Paraná Apara, 10 km NW of confluence of Juruá and Solimões rivers, Mamirauá Reserve
*Gymnotus*	*diamantinensis*	14°20'S, 56°30'W	Brazil, Mato Grosso, Diamantino, upper rio Arinos at Rio Tapajós system, tributary of rio Preto, at road to São Francisco
*Gymnotus*	*ucamara*	05°20'S, 74°29'W	Peru, Loreto, Rio Ucayali, Rio Pacaya, Cocha Zapote, in Pacaya-Samiria National Reserve
*Gymnotus*	*choco*	05°03'N, 77°03'W	Colombia, Chocó, Río Baudó, Boca de Pepé
*Gymnotus*	*ardilai*	~07°07'02"N, 73°09'41"W	Colombia, Santander, Girón, Río de Oro
*Gymnotus*	*pantanal*	20°11'78"S, 56°30'13"W	Brazil, Mato Grosso do Sul, Rio Miranda
*Gymnotus*	*curupira*	03°26'01"S, 64°43'47"W	Brazil, Amazonas, Rio Tefé, Lago Tefé, Igarapé Curupira, terra firme swamp
*Gymnotus*	*obscurus*	03°06'37"S, 64°47'49"W	Brazil, Amazonas, Municipality of Álvares, Mamirauá Sustainable Development Reserve, Caño do Lago Mamirauá at Comunidade Boca do Mamiraua
*Gymnotus*	*varzea*	03°07'42"S, 64°48'02"W	Brazil, Amazonas, Municipality of Álvares, Mamirauá Sustainable Development Reserve, Ressaca da Vila Alencar
*Gymnotus*	*chimarrao*	29°21'09"S, 51°57'28"W	Brazil, Rio Grande do Sul, Taquari Drainage, Arroio do Meio, Arroio Grande
*Gymnotus*	*chaviro*	09°31'11"S, 72°45'45"W	Peru, Ucayali, Alto Yuruá drainage, Quebrada Dos y medio, small stream ca. 2 km NW the town of Breu
*Gymnotus*	*omarorum*	34°50'20"S, 55°06'52"W	Uruguay, Maldonado, Rio Cisne drainage, Laguna del Sauce
*Gymnotus*	*interruptus*	14°36'17"S, 40°06'09"W	Brazil, Bahia, Rio Gongogi drainage, Rio de Contas basin, Riacho Cambiriba, Guaíra balneary, Iguaí
*Gymnotus*	*capanema*	01°07'54"S, 47°03'53"W	Brazil, Pará, municipality of Capanema, Açaiteuazinho River
*Gymnotus*	*cuia*	30°22’52”S, 51°01’25”W	Brazil, Rio Grande do Sul, Viamão, Lagoa Verde, Itapuã State Park, Rio Grande, do Sul
*Gymnotus*	*eyra*	12°33'37"S, 70°06'03"W	Peru, Madre de Dios, Manu, Cuenca Río Los Amigos, Aguajal cicra Pozo Pedro
*Gymnotus*	*riberalta*	10°54’47”S, 65°59’49”W	Bolivia, Beni, Riberalta, Rio Beni, Rio Madeira drainage, Rio Amazonas drainage, Arroyo near Lago de San Jose
*Gymnotus*	* darwini*	07°48’37”S, 34°57’25”W	Brazil, Pernambuco, Igarassu, igarapé Jacoca (or Tabatinga), rio Botafogo basin at Refúgio Ecológico Charles Darwin
*Lamontianus*	*anguillaris*	05°32'N, 55°10'W	Surinam, Commewijne, Coropina Creek
*Lamontianus*	*cataniapo*	01°55'N, 67°02'W	Venezuela, Amazonas, lagoon NE of airport of San Carlos de Rio Negro
*Lamontianus*	*pedanopterus*	01°58'N, 67°00'W	Venezuela, Amazonas, where Caño Temblador crosses road from San Carlos de Rio Negro to Solano
*Lamontianus*	*tiquie*	00°13'00"N, 69°36'00"W	Brazil, Amazonas, Rio Tiquié drainage, comunidade de São José, Igarapé Espuma
*Lamontianus*	*n*. *sp*. *ARAP*	02°43'49"S, 55°35'35"W	Brazil, Santarém, Rio Amazonas drainage, Rio Tapajós, Rio Arapiuns, Rio Mentai, Igarapé Jararaca, 1.4 km and 118° from Comunidade Cachoeirinha do Mentai
*Lamontianus*	*n*. *sp*. *ARIP*	10°22'03"S, 59°24'25"W	Brazil, Mato Grosso, Rio Amazonas drainage, Rio Aripuanã
*Pantherus*	*pantherinus*	~23°55’10”S, 46°24’17”W	Brazil, São Paulo, Santos
*Pantherus*	*capitimaculatus*	16°57'04"S, 39°33'21"W	Brazil, Bahia, Itamaraju, ~7 km north of Itamaraju where Rio do Ouro crosses BR-101
*Pantherus*	*refugio*	30°50'54"S, 52°23'19"W	Brazil, Rio Grande do Sul, Laguna Dos Patos drainage, Amaral Ferrador
*Tigre*	*inaequilabiatus*	~40°40'S, 58°30'W	Argentina, Buenos Aires, Rio de La Plata estuary environment near Buenos Aires
*Tigre*	*esmeraldas*	01°05'N, 79°03'W	Ecuador, Bolívar, Ríos Cayapas drainage, Hoja Blanca near San Miguel
*Tigre*	*henni*	03°53'N, 77°04'W	Colombia, Valle de Cauca, Río San Juan drainage, creek near mouth of Río Calima N of Buenaventura
*Tigre*	*paraguensis*	26°35'S, 55°34'W	Paraguay, Department of Paraguay, Itapuã, Río Paraná drainage, Arroyo Tembey
*Tigre*	*tigre*	04°09'S, 69°57'W	Colombia, Amazonas, floating macrophytes along N shore of Río Amazonas near Leticia
*Tigrinus*	*coatesi*	~02°25'S, 54°10'W	Brazil, Pará, S bank affluent of Rio Amazonas near Santarém
*Tigrinus*	*coropinae*	05°32'N, 55°10'W	Surinam, Commewijne, Coropina Creek
*Tigrinus*	*stenoleucas*	~03°8’14”N, 65°52’43”W	Venezuela, Amazonas, Rio Casiquiare drainage, Caño Caripo, near the bifurcation with the Rio Orinoco
*Tigrinus*	*jonasi*	03°02'48"S, 64°51'22"W	Brazil, Amazonas, Cano do Lago Rato, Mamirauá Reserve
*Tigrinus*	*melanopleura*	03°02'36"S, 64°51'02"W	Brazil, Amazonas, Cano do Lago Rato, Mamirauá Reserve
*Tigrinus*	*onca*	03°02'36"S, 64°51'02"W	Brazil, Amazonas, Cano do Lago Rato, Mamirauá Reserve
*Tigrinus*	*javari*	04°22'S, 70°31'W	Peru, Loreto, Río Yavari (Rio Javarí) drainage, Quebrada Caraná near Buen Sucesso
*Tijax*	*cylindricus*	15°15’16”N, 89°05’06”W	Guatemala, Rio Motagua drainage, brook E of Los Amates
*Tijax*	*maculosus*	14°04'N, 90°37'W	Guatemala, Santa Rosa, diversion of channel from Maria Linda ~20 km. E of Escuintla
*Tijax*	*panamensis*	08°59'N, 81°55'W	Panama, Bocas del Toro, small creek into the Río Cricamola near Konkitu

**Fig 1:**
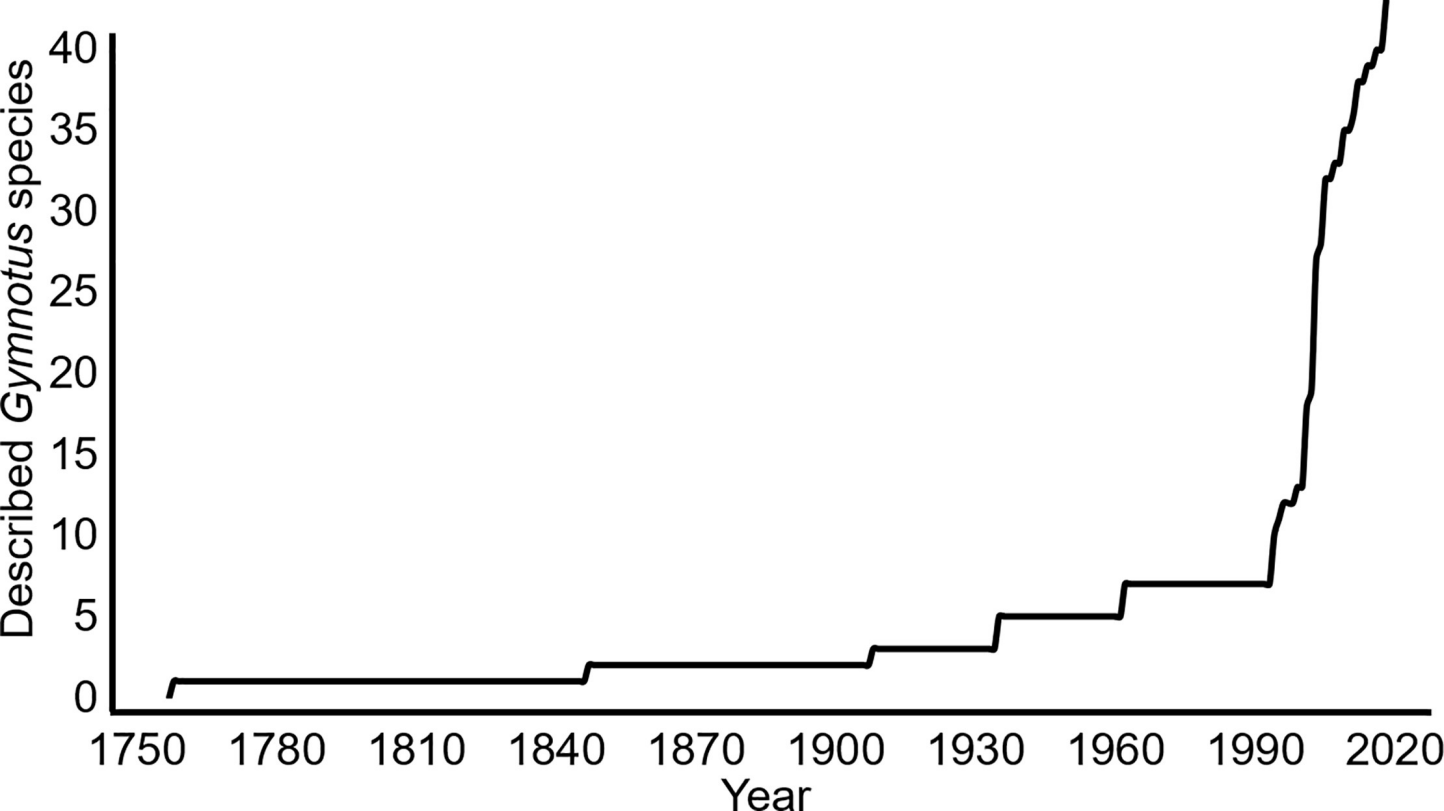
Timeline for the description of new gymnotine species, starting with *G*. *carapo* (Linnaeus) in 1758. Note the rapid acceleration species descriptions in the last three decades, similar to that observed in many clades of Neotropical freshwater fishes.

Knowledge of *Gymnotus* species diversity has expanded rapidly in the past 20 years [5–7] (Fig 1), with the descriptions of 34 species or 77% of the currently-known diversity of this clade. This expansion of knowledge at the species level has been accompanied by seven major efforts to create a systematic classification of the genus. Seven publications [5,8–13] each introduced species-group taxonomies within *Gymnotus*, which although lacking some species and occasionally incongruent in their inter-group relationships, are notable for their broad similarities (Tables 3 and 4). Thus, concurrent with a rapid increase in our understanding of *Gymnotus* as a species-rich genus, an informal but widely-accepted taxonomy was developed for use below the genus level.

**Table 3:** List of valid gymnotine species and higher taxa to which each was assigned in three taxonomic revisions from 1995–2003. Species are marked "undescribed" if they had not been described at the time of the study, "not investigated" if they fell outside the scope of that study, and "not grouped" if they were not assigned to a higher taxon.

		Previous Higher Classifications		
Subgenus	Species	Albert & Miller 1995	Albert 2001	Albert & Crampton 2003
*Gymnotus*	*carapo*	*G*. *carapo* species group	*G*. *carapo* species group	*G*. *carapo* species group
*Gymnotus*	*bahianus*	Undescribed	*G*. *carapo* species group	*G*. *carapo* species group
*Gymnotus*	*sylvius*	Undescribed	*G*. *carapo* species group	*G*. *carapo* species group
*Gymnotus*	*mamiraua*	Undescribed	*G*. *carapo* species group	*G*. *carapo* species group
*Gymnotus*	*arapaima*	Undescribed	*G*. *carapo* species group	*G*. *carapo* species group
*Gymnotus*	*diamantinensis*	Undescribed	Undescribed	*G*. *carapo* species group
*Gymnotus*	*ucamara*	Undescribed	Undescribed	Undescribed
*Gymnotus*	*choco*	Undescribed	Undescribed	Undescribed
*Gymnotus*	*ardilai*	Undescribed	Undescribed	Undescribed
*Gymnotus*	*pantanal*	Undescribed	Undescribed	*G*. *pantherinus* species group
*Gymnotus*	*curupira*	Undescribed	Undescribed	*G*. *carapo* species group
*Gymnotus*	*obscurus*	Undescribed	Undescribed	*G*. *carapo* species group
*Gymnotus*	*varzea*	Undescribed	Undescribed	*G*. *carapo* species group
*Gymnotus*	*chimarrao*	Undescribed	Undescribed	Undescribed
*Gymnotus*	*chaviro*	Undescribed	Undescribed	Undescribed
*Gymnotus*	*omarorum*	Undescribed	Undescribed	Undescribed
*Gymnotus*	*interruptus*	Undescribed	Undescribed	Undescribed
*Gymnotus*	*capanema*	Undescribed	Undescribed	Undescribed
*Gymnotus*	*cuia*	Undescribed	Undescribed	Undescribed
*Gymnotus*	*eyra*	Undescribed	Undescribed	Undescribed
*Gymnotus*	*riberalta*	Undescribed	Undescribed	Undescribed
*Gymnotus*	*darwini*	Undescribed	Undescribed	Undescribed
*Lamontianus*	*anguillaris*	*G*. *anguillaris* species group	*G*. *anguillaris* species group	*G*. *pantherinus* species group
*Lamontianus*	*cataniapo*	*G*. *anguillaris* species group	*G*. *anguillaris* species group	*G*. *pantherinus* species group
*Lamontianus*	*pedanopterus*	*G*. *anguillaris* species group	*G*. *anguillaris* species group	*G*. *pantherinus* species group
*Lamontianus*	*tiquie*	Undescribed	Undescribed	Undescribed
*Pantherus*	*pantherinus*	*G*. *anguillaris* species group	*G*. *anguillaris* species group	*G*. *pantherinus* species group
*Pantherus*	*capitimaculatus*	Undescribed	Undescribed	Undescribed
*Pantherus*	*refugio*	Undescribed	Undescribed	Undescribed
*Tigre*	*inaequilabiatus*	*G*. *carapo* species group	*G*. *carapo* species group	*G*. *carapo* species group
*Tigre*	*esmeraldas*	Undescribed	Undescribed	*G*. *carapo* species group
*Tigre*	*henni*	Undescribed	Undescribed	*G*. *carapo* species group
*Tigre*	*paraguensis*	Undescribed	Undescribed	*G*. *carapo* species group
*Tigre*	*tigre*	Undescribed	Undescribed	*G*. *carapo* species group
*Tigrinus*	*coatesi*	*G*. *anguillaris* species group	*G*. *anguillaris* species group	*G*. *pantherinus* species group
*Tigrinus*	*coropinae*	*G*. *anguillaris* species group	*G*. *anguillaris* species group	*G*. *pantherinus* species group
*Tigrinus*	*stenoleucas*	*G*. *anguillaris* species group	*G*. *anguillaris* species group	*G*. *pantherinus* species group
*Tigrinus*	*jonasi*	Undescribed	*G*. *anguillaris* species group	*G*. *pantherinus* species group
*Tigrinus*	*melanopleura*	Undescribed	*G*. *anguillaris* species group	*G*. *pantherinus* species group
*Tigrinus*	*onca*	Undescribed	*G*. *anguillaris* species group	*G*. *pantherinus* species group
*Tigrinus*	*javari*	Undescribed	Undescribed	*G*. *pantherinus* species group
*Tijax*	*cylindricus*	*G*. *cylindricus* species group	*G*. *cylindricus* species group	*G*. *cylindricus* species group
*Tijax*	*maculosus*	*G*. *cylindricus* species group	*G*. *cylindricus* species group	*G*. *cylindricus* species group
*Tijax*	*panamensis*	Undescribed	Undescribed	Undescribed

**Table 4:** List of valid gymnotine species and higher taxa to which each was assigned in three taxonomic revisions from 2010–2017. Species are marked "undescribed" if they had not been described at the time of the study, "not investigated" if they fell outside the scope of that study, and "not grouped" if they were not assigned to a higher taxon.

		Previous Higher Classifications			
Subgenus	Species	Lovejoy *et*. *al*. 2010	Crampton *et al*. 2013	Tagliacollo *et al*. 2016	van der Sleen & Albert 2017
*Gymnotus*	*carapo*	*G*. *carapo* clade	*G*. *carapo*-D clade	*G*. *carapo* clade	*G*. *carapo* group
*Gymnotus*	*bahianus*	*G*. *carapo* clade	*G*. *carapo*-D clade	*G*. *carapo* clade	*G*. *carapo* group
*Gymnotus*	*sylvius*	*G*. *carapo* clade	*G*. *carapo*-D clade	*G*. *carapo* clade	*G*. *carapo* group
*Gymnotus*	*mamiraua*	*G*. *carapo* clade	*G*. *carapo*-B clade	*G*. *carapo* clade	*G*. *varzea* group
*Gymnotus*	*arapaima*	*G*. *carapo* clade	*G*. *carapo*-D clade	*G*. *carapo* clade	*G*. *carapo* group
*Gymnotus*	*diamantinensis*	*G*. *carapo* clade	Not Grouped	Not Investigated	*G*. *carapo* group
*Gymnotus*	*ucamara*	*G*. *carapo* clade	*G*. *carapo*-D clade	*G*. *carapo* clade	*G*. *carapo* group
*Gymnotus*	*choco*	*G*. *carapo* clade	*G*. *carapo*-C clade	*G*. *carapo* clade	*G*. *carapo* group
*Gymnotus*	*ardilai*	Not Investigated	*G*. *carapo*-C clade	*G*. *carapo* clade	*G*. *carapo* group
*Gymnotus*	*pantanal*	Not Investigated	*G*. *carapo*-A clade	*G*. *carapo* clade	*G*. *varzea* group
*Gymnotus*	*curupira*	*G*. *carapo* clade	*G*. *carapo*-A clade	*G*. *carapo* clade	*G*. *varzea* group
*Gymnotus*	*obscurus*	*G*. *carapo* clade	*G*. *carapo*-A clade	*G*. *carapo* clade	*G*. *varzea* group
*Gymnotus*	*varzea*	*G*. *carapo* clade	*G*. *carapo*-A clade	*G*. *carapo* clade	*G*. *varzea* group
*Gymnotus*	*chimarrao*	Undescribed	Not Grouped	Not Investigated	*G*. *carapo* group
*Gymnotus*	*chaviro*	Undescribed	*G*. *carapo*-A clade	*G*. *carapo* clade	*G*. *carapo* group
*Gymnotus*	*omarorum*	Undescribed	*G*. *carapo*-B clade	*G*. *carapo* clade	*G*. *carapo* group
*Gymnotus*	*interruptus*	Undescribed	Not Investigated	Not Investigated	*G*. *carapo* group
*Gymnotus*	*capanema*	Undescribed	Not Grouped	Not Investigated	*G*. *varzea* group
*Gymnotus*	*cuia*	Undescribed	*G*. *carapo*-B clade	*G*. *carapo* clade	Undescribed
*Gymnotus*	*eyra*	Undescribed	Undescribed	Not Investigated	Undescribed
*Gymnotus*	*riberalta*	Undescribed	*G*. *carapo*-A clade	Not Investigated	Undescribed
*Gymnotus*	*darwini*	Undescribed	Undescribed	Undescribed	Undescribed
*Lamontianus*	*anguillaris*	G2 clade	*G*. *cataniapo* clade	*G*. *anguillaris* clade	*G*. *anguillaris* group
*Lamontianus*	*cataniapo*	G2 clade	*G*. *cataniapo* clade	*G*. *anguillaris* clade	*G*. *anguillaris* group
*Lamontianus*	*pedanopterus*	G2 clade	*G*. *cataniapo* clade	*G*. *anguillaris* clade	*G*. *anguillaris* group
*Lamontianus*	*tiquie*	Undescribed	Not Grouped	Not Investigated	*G*. *anguillaris* group
*Pantherus*	*pantherinus*	Not Grouped	*G*. *pantherinus* clade	*G*. *pantherinus* clade	Not Investigated
*Pantherus*	*capitimaculatus*	Undescribed	Undescribed	Not Investigated	Not Investigated
*Pantherus*	*refugio*	Undescribed	Undescribed	Undescribed	Not Investigated
*Tigre*	*inaequilabiatus*	*G*. *carapo* clade	Not Grouped	Not Investigated	*G*. *tigre* group
*Tigre*	*esmeraldas*	*G*. *carapo* clade	*G*. *henni* clade	Not Investigated	*G*. *tigre* group
*Tigre*	*henni*	*G*. *carapo* clade	*G*. *henni* clade	*G*. *tigre* clade	*G*. *tigre* group
*Tigre*	*paraguensis*	*G*. *carapo* clade	Not Grouped	Not Investigated	*G*. *tigre* group
*Tigre*	*tigre*	*G*. *carapo* clade	*G*. *henni* clade	*G*. *tigre* clade	*G*. *tigre* group
*Tigrinus*	*coatesi*	G1 clade	*G*. *coatesi* clade	*G*. *coatesi* clade	*G*. *coatesi* group
*Tigrinus*	*coropinae*	G1 clade	*G*. *coatesi* clade	*G*. *coatesi* clade	*G*. *coatesi* group
*Tigrinus*	*stenoleucas*	G1 clade	*G*. *coatesi* clade	*G*. *coatesi* clade	*G*. *coatesi* group
*Tigrinus*	*jonasi*	G1 clade	*G*. *coatesi* clade	*G*. *coatesi* clade	*G*. *coatesi* group
*Tigrinus*	*melanopleura*	G1 clade	*G*. *coatesi* clade	Not Investigated	*G*. *coatesi* group
*Tigrinus*	*onca*	G1 clade	*G*. *coatesi* clade	Not Investigated	*G*. *coatesi* group
*Tigrinus*	*javari*	G1 clade	*G*. *coatesi* clade	*G*. *coatesi* clade	*G*. *coatesi* group
*Tijax*	*cylindricus*	Not Grouped	*G*. *cylindricus* clade	*G*. *cylindricus* clade	Not Investigated
*Tijax*	*maculosus*	Not Grouped	*G*. *cylindricus* clade	*G*. *cylindricus* clade	Not Investigated
*Tijax*	*panamensis*	G1 clade	*G*. *cylindricus* clade	*G*. *cylindricus* clade	Not Investigated

In this study we present the most well resolved and taxon-complete phylogeny of *Gymnotus* to date, including all valid species as of this writing and synthesizing a wealth of morphological data (115 characters, including aspects of color pattern, morphometrics and osteology) with a five-gene mitochondrial and nuclear dataset (16S, COX1, CYT-B, RAG2 & ZIC1, approximately 3,000 bp). These data were analyzed using the model-based total evidence (MBTE) framework described in [12], resulting in a well-resolved phylogeny, which was then time-calibrate using biogeographic priors in the absence of pertinent fossils. We propose a new classification of the family Gymnotidae that includes two subfamilies (Electrophorinae and Gymnotinae, following [14]), two genera (*Electrophorus*, *Gymnotus*) and six subgenera within the genus *Gymnotus*. This new classification more completely reflects the current understanding of gymnotid diversity and phylogeny, with subgenera that correspond to previously-recognized and readily recognizable species-groups. This new classification highlights the relatively deep phylogenetic divergences among the gymnotid clades recognized as subgenera and makes gymnotid diversity more accessible to the broader ichthyological community.

## Materials and methods

### Taxon sampling

The gymnotiform species *Hypopomus artedi* and *Sternopygus macrurus* were used as outgroups in all phylogenetic analyses. Morphological data, including aspects of coloration, morphology, meristics and osteology, were collected for 790 specimens including all 43 valid described *Gymnotus* as of this writing, as well as two undescribed species herein designated *G*. n. sp. ‘ARAP’ and *G*. n. sp. ‘ARIP’, following methods in [9] and [1]. Type specimens were included in the analysis whenever possible; the authors personally examined type material for 93% of the Gymnotidae (excluding only *G*. *interruptus*, *G*. *capitimaculatus* and *G*. *pantherinus*).

Written consent for all project activities was approved by UL Lafayette IACUC #2010-8717-064 for all activities related to this specific project and the resulting manuscript. All authors of this manuscript, where applicable, have given written informed consent (as outlined in PLOS consent form) to publish their image as well as any specific case details. Each participant’s copy of The Consent Form for Publication in a PLOS Journal has been securely filed in the individual's case notes.

### Retrieving orthologous DNA sequences from GenBank

The package phyLotaR [15] was used for identifying and retrieving orthologous sequence clusters of *Gymnotus* (NCBI:txid36670) from GenBank release 230 (February 15 2019, available here: https://www.ncbi.nlm.nih.gov/genbank/release/230/). This package is an implementation of the PhyLota Browser [16], a pipeline that implements BLAST searches [17] to both identify and download sequence clusters for listed taxonomic groups to assemble a robust collection of sequences in a reproducible way based on publicly-available gene sequences while avoiding selection bias on the part of the assembler. Importantly, PhyLota always recovers the same groups of sequences given the same starting taxon and the same GenBank release (old releases are available online). We then reviewed all sequence clusters identified for *Gymnotus* (S1 Supplementary Material) and chose those that had at least 25% of the taxon sampling of *Gymnotus* diversity (i.e. > 14 sequences) total 13 independent markers. The sequences assembled by phyLotaR as discussed above were then combined with the gymnotid sequences compiled by [12] and available in the supplemental material for that study.

### Sequence alignments

Each gene was independently aligned using MAFFT 5.3 [18] under default parameters. Automated alignment trimming was performed in TrimAl [19]. To detect potential errors such as amplification of pseudogenes, paralogous copies or potential laboratory cross-contamination, each gene alignment was analyzed in PhyML 3.0 [20]. The final molecular alignment used in our analyses, as well as the accession numbers for all sequences gathered by [12], is presented in S2 Supplementary Material.

This alignment was submitted to the GUIDANCE2 server for identification of unreliably aligned columns or sequences [21]. The final alignment received a GUIDANCE score of 0.585436, indicating high reliability of our alignment. Following the recommendations of [21], who acknowledge that the value of data filtering can vary across evolutionary scenarios and risk losing evolutionary information, we chose to retain all sequences and columns recovered by the PhyLota pipeline to maximize the amount of information used in this analysis.

### Morphological characters

The morphological dataset used in our analyses consists of 115 characters including multiple aspects of osteology, neurology, meristics, morphometrics and color pattern. Characters and states were acquired from multiple sources, including phylogenetic revisions [9,12,22], published species descriptions [1,5–8,23–44] and examination of museum specimens. Detailed definitions of osteological and other terminology, as well as descriptions and illustrations of salient characters, both photographical and camera-lucida, can be found in [9], pages 11–62 and Albert and [8], pages 668–670 and are not reproduced here for brevity. The resulting morphological matrix used in all analyses and a table defining the character codings, is presented in S3 Supplementary Material.

### Nucleotide substitution model selection

For the molecular dataset, optimal partitioning schemes and nucleotide substitution models were estimated in PartitionFinder 2 [45]. Two independent analyses were conducted to estimate the best partitioning schemes including substitution models implemented in Garli 2.01 [46] and MrBayes 3.2 [47]. Each analysis assumed a fully partitioned dataset (by gene and by codon position in protein-coding genes) and the best-fit partitioning scheme with its respective substitution models was selected according to the Akaike Information Criterion with correction (AICc). Substitution models with a proportion of invariant sites (+I) were excluded because the rate of heterogeneity is already accounted by the gamma shape parameters (+C).

For the morphological dataset, the *Mkv* model was applied for discrete character evolution. In the *Mkv* model, *M* refers to Markov chain, *k* refers to the number of discrete character states (with *k*>2) and *v* refers to the number of variable characters [48]. The *Mkv* model used here is discussed in detail in [12,48]; in summary, it applies simple parsimony in a likelihood framework, creating independent partitions for molecular and morphological data which can be treated using different evolutionary models.

### Maximum-likelihood phylogenetic inference

Maximum-likelihood (ML) analyses of combined morphological + molecular (hereafter supermatrix) datasets were conducted in Garli 2.01 [46]. Models of nucleotide evolution were estimated in PartitionFinder 2 [45]. The Mkv model [48] was used for the morphological dataset. The ML analyses consisted of two independent runs, each one starting from a BioNJ starting tree and using the Subtree Pruning and Regrafting (SPR) algorithm to search for tree improvement in terms of likelihood scores. All other parameters were set as default. To assess node support, 100 non-parametric bootstrap replications were performed for each independent tree search resulting in a total of 200 pseudo-replicates. A consensus tree with bootstraps was computed using the function SumTrees from DendroPy 3.7.0 [49].

### Bayesian phylogenetic inference

The Bayesian inference (BI) analysis of supermatrix datasets was conducted in MrBayes 3.2 [47]. Models of nucleotide evolution were estimated in PartitionFinder 2 [45]. Mkv model [48] was used for the morphological dataset, as discussed above. The BI analysis consisted of two runs (four chains each) of the Metropolis-Coupled Markov Chain Monte Carlo (MC^3^). Each run was comprised of 5.0 x 10^7^ generations with generation. All other parameters were set as default. To ensure model parameter values and a single tree sampled every 5 x 10^3^ adequate mixing of the MCMC, effective sample size values (ESS > 200) were inspected for parameter estimates in Tracer 1.5. The two independent runs were summarized with ‘‘sump” and ‘‘sumt” commands in MrBayes 3.2 [47]. The initial 25% of sampled topologies were discarded as burn-in procedure. The remaining topologies were used to construct a 50% majority-rule consensus tree. Posterior probabilities were visualized in FigTree 1.4.0.3 [50].

### Single-dataset trees

For comparative purposes, four additional phylogenies were generated using the morphological and molecular data separately. First, two phylogenies were constructed using the same Bayesian methodology described above but using only the morphological (S4 Supplementary Material) and only the molecular (S5 Supplementary Material) data. Next, a maximum likelihood phylogeny was constructed using RaxML [51] on the Cipres Science Gateway portal [52] using only the molecular alignment (S6 Supplementary Material). Finally, a simple parsimony tree was constructed in TNT [53] using the morphological data only (S7 Supplementary Material). Tree files for the total evidence Bayesian (S8 Supplementary Material) and Likelihood (S9 Supplementary Material) phylogenies are included as supplements as well for comparison.

### Time-calibrated tree

Divergence time estimates were performed in BEAST 2.0 [54] under an uncorrelated lognormal clock and birth-death speciation process. Partition gene schemes and evolutionary models were estimated in PartitionFinder 2 [45]. Due to the absence of *Gymnotus* fossils, we calibrated tree nodes from geological age of the Colombian Eastern Cordillera (c 11.0 Ma). This paleogeographic event is hypothesized to divide many fish lineages between cis and trans-Andean river basins [55]. For this study we used the following two sister-taxon pairs: 1) cis-Andean ((*G*. *tigre*, *G*. *paraguensis*), *G*. *inaequilabiatus*) and trans-Andean (*G*. *esmeraldas*, *G*. *henni*) and 2) cis-Andean (*G*. *carapo septentrionalis*) and trans-Andean (*G*. *ardilai*).

Divergence time in BEAST 2 [54] was comprised of two independent Markov Chain of Monte Carlo (MCMC) of 50 million generations each, sampling a tree topology and log parameters at every 5000 generations. The two runs were combined using LogCombiner v1.8.0. [54]. The diagnosis of MCMC runs and posterior probabilities were evaluated by inspections of the Effective Sample Sizes (ESS) in the program Tracer [56]. As a burn-in, 25% initial posterior trees were removed and the remaining trees were summarized in a Maximum Clade Credibility (MCC) with node heights represented by median heights. To evaluate the effects of geological prior calibrations on the posterior divergence time estimates, analyses from an empty alignment (alignment containing only questions marks) were performed.

## Results

### Comparisons between total-evidence phylogenies

The Bayesian and Maximum Likelihood (ML) total evidence analyses recovered broadly similar relationships within and among clades recognized in previous studies, with a single major exception. The Bayesian analysis recovered the *G*. *anguillaris* clade (*sensu* [12]) as sister to the *G*. *coatesi* clade (*sensu* [12]) with a node support of 100%, whereas the total evidence ML phylogeny recovered *G*. *anguillaris* clade as sister to a clade comprised of the *G*. *carapo* clade (*sensu* [12]), the *G*. *cylindricus* clade (*sensu* [12]) and the *G*. *tigre* clade (*sensu* [12]), with the *G*. *coatesi* clade as the sister all four of these clades. In constructing the revised classification of *Gymnotus* we choose the results of the Bayesian analyses over those of the ML analysis in their support of a sister group relationship between the *G*. *anguillaris* clade and the *G*. *coatesi* clade, due to high support for this topology in the Bayesian analyses and the relatively poor node support (75% bootstrap) for the *G*. *anguillaris* clade as sister to all other *Gymnotus* clades except the *G*. *pantherinus* clade (*sensu* [12]).

### Comparisons between morphological and molecular phylogenies

The topology recovered in the morphology-only analyses, both parsimony and Bayesian, differs from the total-evidence and molecular topologies in two important regards. First, the Central American *G*. *cylindricus* clade (*sensu* [12]) is recovered as monophyletic but as sister to the rest of the genus *Gymnotus*. We propose this may be due to a suite of characters associated with color pattern, dentition and the number of preopercular-mandibular sensory canal pores in the dorsoposterior portion of the preopercle all shared by all species of the *G*. *cylindricus* clade. In all phylogenetic analyses where robust molecular data are included for the *G*. *cylindricus* clade, we recover their location as sister to the *G*. *carapo* clade. Notably, the *G*. *cylindricus* clade is also similar to the *G*. *carapo* clade on many osteological characters (see the full diagnoses of each presented below) further suggesting a deep homology between the two and that the morphology-only analyses fell victim to over-parameterization of the characters associated with color pattern.

Second, the morphology-only parsimony and Bayesian phylogenies recover a monophyletic group comprising the species *G*. *pantanal*, *G*. *riberalta* and *G*. *capanema* as sister to the *G*. *anguillaris* clade. We interpret this result to arise from a shared suite of characters associated with a long, cylindrical body, including a longer body cavity with more precaudal vertebrae and a slender body profile. This topology is unique to the morphology-only trees, whereas in all molecular and total-evidence analyses, the *G*. *carapo* clade is recovered as monophyletic, including the species *G*. *pantanal*, *G*. *riberalta* and *G*. *capanema*. We propose that the morphology-only analyses fail to recover a monophyletic *G*. *carapo* clade, which is well supported by a wealth of genetic data as well as aspects of color pattern, dentition and osteology. Notably in this particular case, *G*. *pantanal*, *G*. *riberalta* and *G*. *capanema* occasionally possess a single preopercular-mandibular sensory canal pore at the dorsoposterior corner of the preopercle, a condition also observed in species of the *G*. *anguillaris* clade, but which appears to be a phylogenetic reversal to the ancestral, single-pored state present other *Gymnotus*.

These two aspects of the morphological analyses, which stand out as incongruent with the molecular and total-evidence analyses, serve to emphasize the importance of character selection and of the power of the total-evidence approach to overcome potentially misleading aspects of the morphological dataset.

### Characterization of major clades

**Fig 2:**
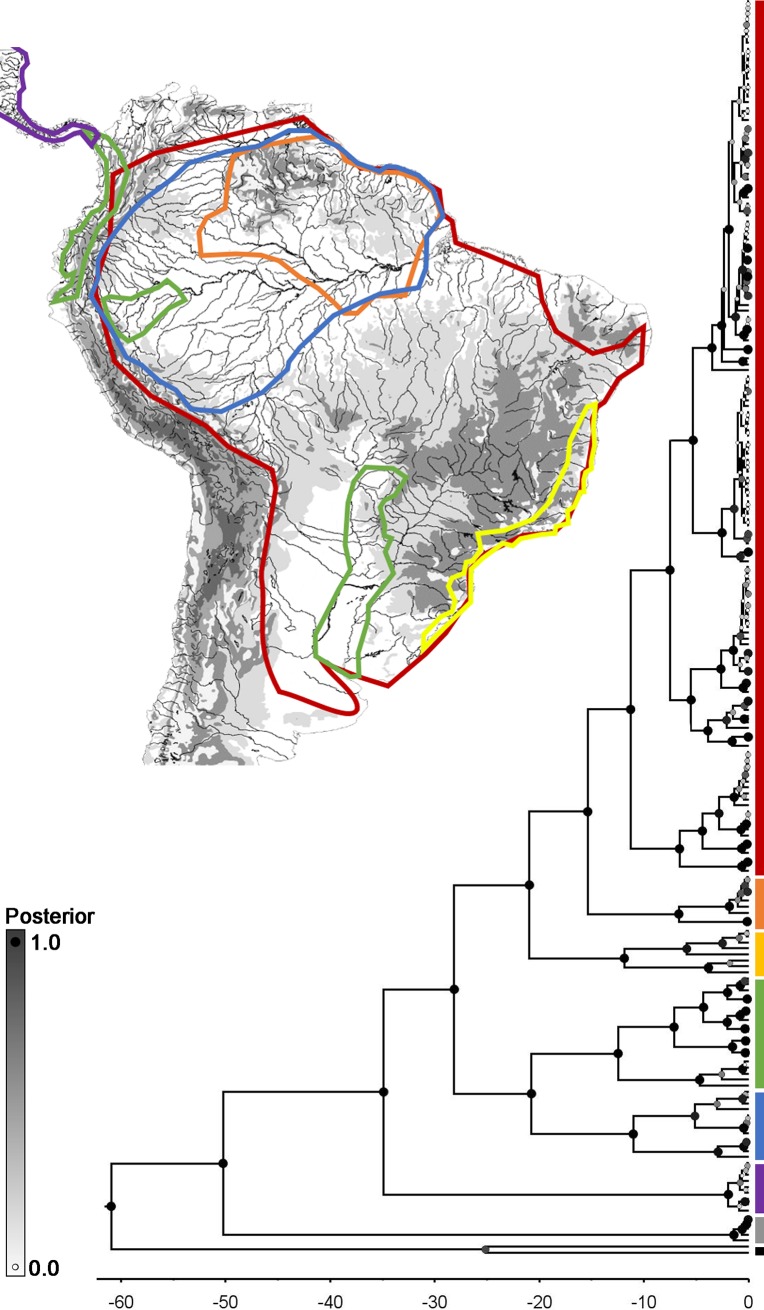
Time–calibrated phylogeny recovered from the Bayesian analysis. N = 211 specimens representing 48 (45 Gymnotinae, one Electrophorinae, two outgroups) species. Colors of clades as follows: red = *Gymnotus*, orange = *Tijax*, Yellow = *Tigre*, green = *Tigrinus*, blue = *Lamontianus*, purple = *Pantherus*, grey = *Electrophorus*, black = outgroups. Inset depicts the documented geographic ranges of each subgenus. Nodes color coded to indicate posterior values, with larger, darker circles representing higher support and smaller, lighter circles indicating lower support.

**Fig 3:**
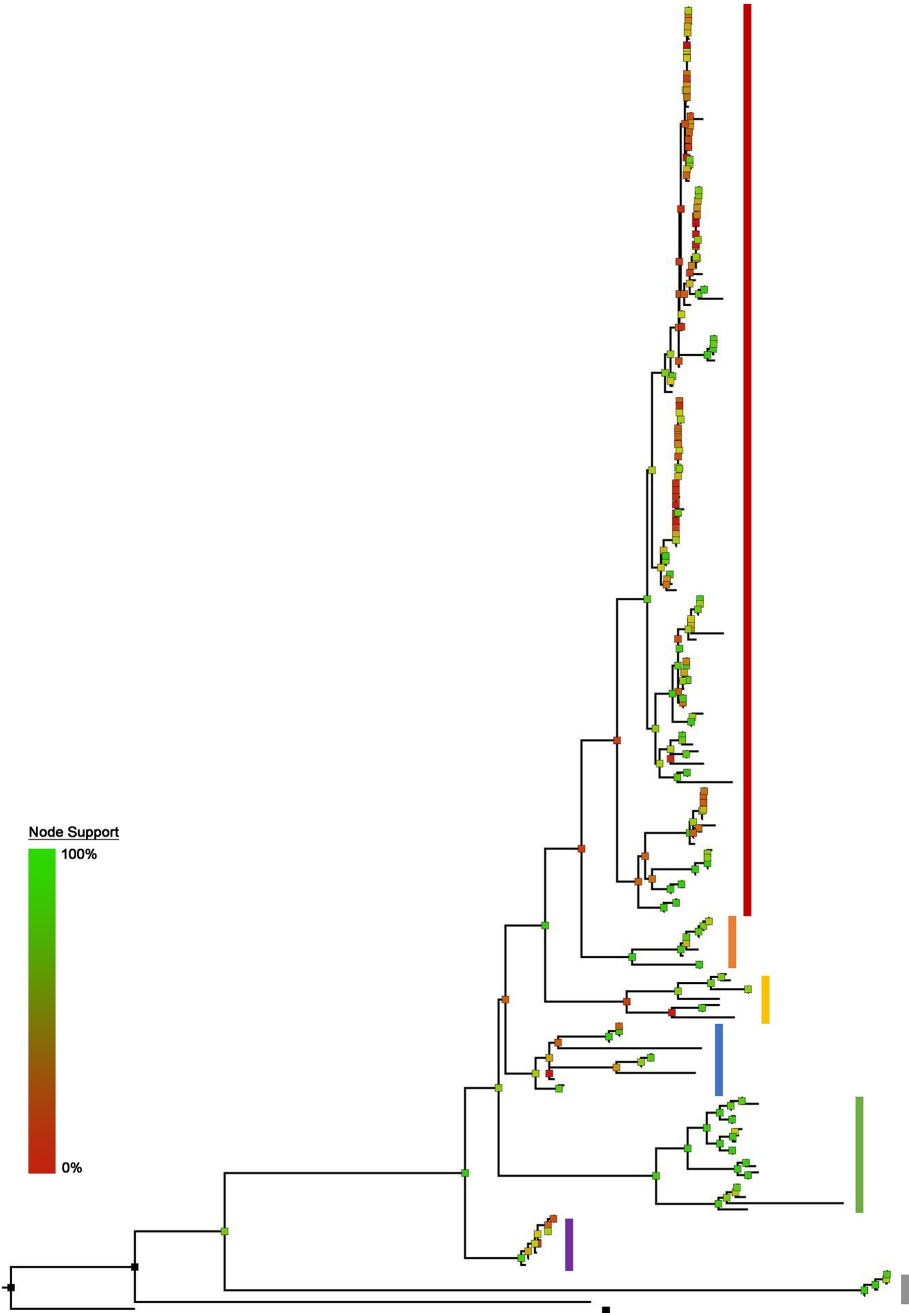
Phylogeny of Gymnotidae from the Maximum–Likelihood analysis of 5 concatenated genes with 5533 bp. N = 211 specimens representing 48 (45 Gymnotinae, one Electrophorinae, two outgroup) species. Colors of clades as follows: red = *Gymnotus*, orange = *Tijax*, Yellow = *Tigre*, green = *Tigrinus*, blue = *Lamontianus*, purple = *Pantherus*, grey = *Electrophorus*, black = outgroups. Node colors indicate bootstrap support values of nodes, from 100% (green) to 0% (red).

In both the Bayesian (Fig 2) and Maximum-Likelihood (Fig 3) phylogenies we recover more than 20 clades within Gymnotinae, including six main clades within *Gymnotus*, all with distinct morphological characteristics, geographic distributions and relatively deep phylogenetic divergences from one another. These clades correspond broadly to those recovered in previous taxonomic and phylogenetic treatments of the family [5,8–13].

We recover the “*G*. *carapo* clade” *sensu* [12], which is equivalent to the subgenus *Gymnotus* diagnosed herein. The subgenus *Gymnotus* is a continentally-distributed clade with 22 valid species ranging from the Pacific slope of Colombia to northern Argentina, making it the most species-rich, phenotypically disparate and geographically widespread subgenus within the family.

We recover the “*G*. *cylindricus* clade” *sensu* [12], which is equivalent to the subgenus *Tijax* designated herein. *Tijax* includes three of the four gymnotid species in Central America, where all three species are endemic [5,8,57].

We recover the “*G*. *tigre* clade” *sensu* [12], which is equivalent to the subgenus *Tigre* designated herein. *Tigre* is the second most geographically widespread subgenus of the Gymnotinae, with its five species distributed from 8°N in southern Panama to 34°S in northern Argentina. Tigre also includes *G*. *henni* from Pacific draining rivers of Colombia and southern Panama.

We recover the “*G*. *coatesi* clade” *sensu* [12], which is equivalent to the subgenus *Tigrinus* designated herein. The seven species of *Tigrinus* are distributed through the Amazon-Orinoco-Guianas core of the Neotropics and are absent from drainages in the Trans-Andean and La Plata regions.

We recover the “*G*. *anguillaris* clade” *sensu* [12], which is equivalent to the subgenus *Lamontianus* designated herein. The six species of *Lamontianus* (including two new species currently in review) are distributed throughout the Eastern Amazon and rivers draining the Guiana and Brazilian Shields.

We recover the “*G*. *pantherinus* clade” *sensu* [12], which is equivalent to the subgenus *Pantherus* designated herein. The three species of *Pantherus* species are limited to coastal drainages of eastern and southeastern Brazil [30,58] and the Tietê river of the Paraná (La Plata) basin.

### Relationships among subgenera

Our total evidence approach recovers the subgenus *Pantherus* as the sister to all other gymnotine subgenera. This result is consistent with the total-evidence and molecular-only results of [12], who applied a similar approach but used substantially fewer taxa and morphological characters. This result was not recovered in the multigene molecular phylogeny of [10], however that phylogeny did not reach a fully-resolved topology with respect to their *G*. *pantherinus* clade, recovering the group in a three-member polytomy consisting of the subgenus *Pantherus* (their *G*. *pantherinus* clade), *Lamontianus* (their “G2 clade”) and the subgenera *Gymnotus* and *Tijax* (represented only by *G*. *cylindricus*).

We recover the subgenus *Tigrinus* as the sister to the other four remaining subgenera. This result is consistent with those of [10] and [12].

We recover the subgenus *Lamontianus* as the sister to the other three remaining subgenera. This result is consistent with that of [12] but not [10], who recover *Lamontianus* within a polytomy as discussed above.

We recover the subgenus *Tigre* as the sister to the other two remaining subgenera. This is consistent with the molecular and total-evidence phylogenies presented by [12], but not recovered in their morphological analysis, which finds *Tigre* nested within *Gymnotus*. Similarly, [10] recovered *G*. *tigre*, the only subgenus *Tigre* species in their analyses, nested within subgenus *Gymnotus* as well. We propose that an improved osteological dataset and higher taxon sampling contributed the increased phylogenetic resolution for this clade.

We recover the subgenera *Gymnotus* and *Tijax* as sister clades. This result is consistent with the total-evidence analyses of [12], but not their morphology-only analysis, which found the subgenus *Tijax* as the sister group to the other clades. The phylogeny of [9], which included *G*. *carapo*, *G*. *anguillaris* and *G*. *cylindricus*, also found *G*. *cylindricus* as sister clade to the other two, but relatively poor taxon sampling likely influenced this result.

This present study follows a similar methodology to that of [12], but draws upon a larger sampling of species within Gymnotinae. The total-evidence dataset presented in [12] includes 67% of the subgenus *Gymnotus*, 67% of the subgenus *Tijax*, 40% of the subgenus *Tigre*, 71% of the subgenus *Tigrinus*, 75% of the subgenus *Lamontianus* (not including undescribed species reported here) and 33% of the subgenus *Pantherus*, whereas the present study includes all but one valid species of Gymnotidae. The topology recovered here is broadly similar to that of [12], with the major exception being the monophyly of a clade comprised of the subgenera *Lamontianus* and *Tigrinus*. This relationship had previously been hypothesized by [22] based on morphology exclusively, but never previously recovered in genetic or total evidence studies.

### Historical biogeography

Our total-evidence phylogeny, relying on biogeographical markers for time-calibration, recovers the age of the crown-group Gymnotidae between 61 and 50 Ma, with the clade Gymnotinae arising between 50 and 35 Ma, *Pantherus* between 36 and 2.5 Ma, *Lamontianus* between 21 and 11 Ma, *Tigrinus* between 21 and 12 Ma, *Tigre* between 21 and 11 Ma, *Tijax* between 14 and 6.0 Ma and *Gymnotus* between 17 and 10 Ma.

Historical biogeographic analysis suggests the clade composed of *Gymnotus*, *Tigre* and *Tijax* originated in the Western Amazon about 20 Ma and subsequently colonized trans-Andean, Central American and Southern (La Plata) drainages on multiple separate occasions. *Pantherus* is unique among gymnotines in being estimated to have originated on the Southeastern Atlantic coast, with one dispersal to the upper Tietê (upper Parana) presumably by river capture [59]. *Tigrinus* is estimated to have originated in the Amazon-Orinoco-Guianas region. *Lamontianus* is estimated to have arisen on the Amazon Craton prior to the origin of the modern trans-continental Amazon river c. 10 Ma [60,61]. The Amazon Craton consists of the Guianas shield and the northern portion of the Brazilian shield, largely corresponding to the “Eastern Highlands” of Eigenmann [62].

We recover a polyphyletic assemblage of trans-Andean and Central American gymnotines, composed of seven species representing three distinct clades, each representing a different subgenus. First, crown group *Tigre* appeared between 25 and 15 Ma. *Gymnotus esmeraldas* and *G*. *henni*, both found only in trans-Andean drainages, arose about 10.0 +/- 3.0 Ma (this date, however, was used for the time calibration and is therefore not a result of this analysis). Second, crown-group *Tijax* appeared between 25 and 2.5 Ma, suggesting that *Tijax* dispersed to Central America during the Miocene or Pliocene, perhaps in the Early to Middle Miocene, before rise of the Panamanian isthmus [63]. Third, the trans-Andean species of *Gymnotus*, (i.e. *G*. *ardilai* and *G*. *choco*), are most closely related to one another and together most closely related to the cis-Andean species *G*. *carapo septentrionalis* from the Orinoco basin [64].

We also recover a polyphyletic fauna of the eastern and southeastern basins of Brazil and the La Plata basin. This fauna is composed of 11 species representing seven distinct clades in three subgenera: *Pantherus* with three species in one clade, *Tigre* with two species in one clade and *Gymnotus* with seven species in five clades. These clades of *Gymnotus* include: 1) *G*. *pantanal* that arose between 3.5 and 3.0 Ma and is most closely related to *G*. *riberalta* from the upper Madeira basin; 2), *G*. *bahianus* and *G*. *interruptus* that arose 1.5 to 0.5 Ma and are most closely related to *G*. *carapo occidentalis* from the Western Amazon; 3) a clade including a population of *G*. *carapo australis*, *G*. *chimarrao*, *G*. *diamantinensis*, *G*. *omarorum* and *G*. *sylvius* from the upper Parana that arose 12.0 to 2.0 Ma that is most closely related to a clade composed of *G*. *mamiraua* from the central Amazon and *G*. *eyra* from the upper Madeira basin; 4) a clade comprised of another population of *G*. *(G*.*) carapo australis* plus *G*. *cuia* that arose 1.5 to 0.5 Ma and is most closely related to the clade listed above (comprising some *G*. *carapo australis*, *G*. *chimarrao*, *G*. *diamantinensis*, *G*. *omarorum* and *G*. *cf*. *sylvius* from the upper Paraná basin; 5) a comprised of yet other population of *G*. *carapo australis*, *G*. *cf*. *carapo* from the Sao Francisco basin and *G*. *sylvius* from Ribeiro de Iguapé basin that arose 1.0 to 0.1 Ma and is most closely related to *G*. *carapo occidentalis* from the Western Amazon. Taken together, these data suggest the southern humid Neotropics was colonized by the Gymnotinae on at least five different occasions between 5.5 and 0.1 Ma, with four of these clades originating in the Western Amazon and dispersing through the upper Madeira and Paraguay-Paraná basins of the Sub-Andean foreland basin [65].

## Discussion

### Utility of newly designated subgenera

**Fig 4:**
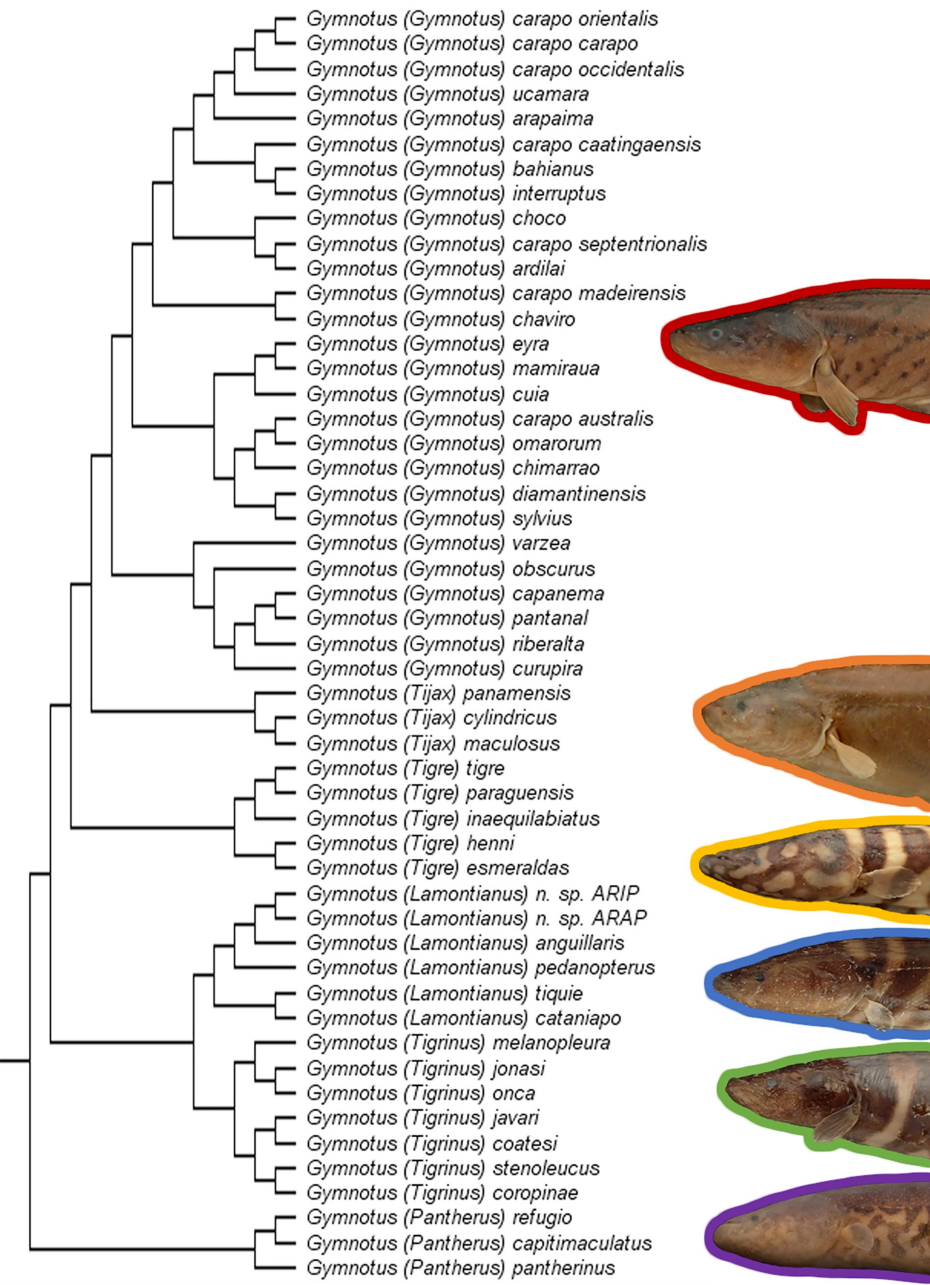
Summary of the phylogeny recovered from the total evidence Bayesian analysis, with each terminal as a single species. Colors as follows: red = *Gymnotus*, orange = *Tijax*, Yellow = *Tigre*, green = *Tigrinus*, blue = *Lamontianus*, purple = *Pantherus*, grey = *Electrophorus*, black = outgroups.

**Fig 5:**
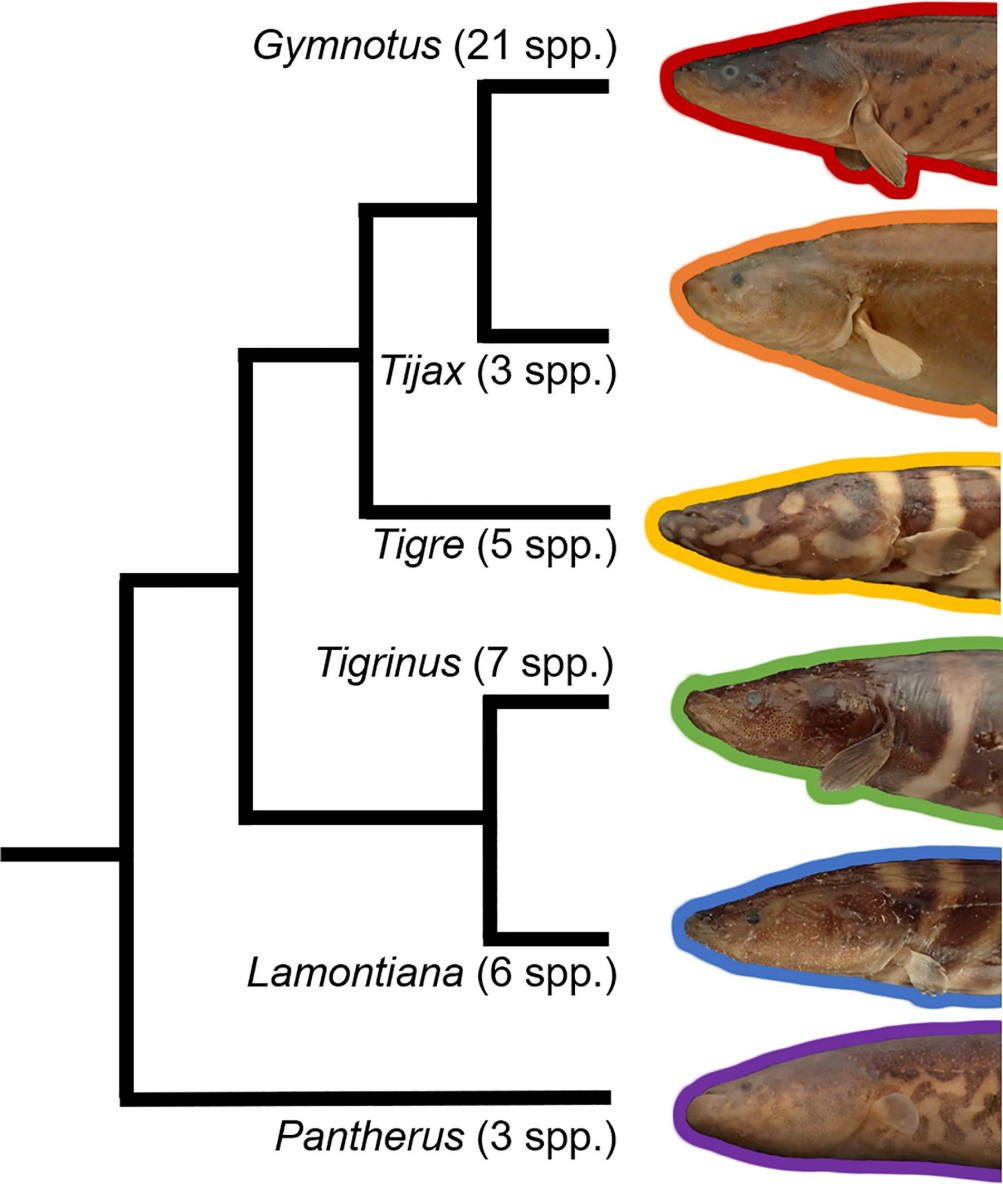
Summary of the relationships among subgenera of the Gymnotinae. Numbers in parentheses indicate number of currently recognized (valid) species.

In this study we constructed the most taxon-complete and character-rich morphological dataset to date for the family Gymnotidae and paired it with a robust multigene dataset (Fig 4). The results of this study improve our phylogenetic understanding of the group, incorporating for the first time all but one of the currently valid species. We recover six major clades of the Gymnotinae, largely similar with results of previously published studies (Fig 5).

The newly proposed classification formalizes an already well-established informal taxonomy of the group [13]. The benefits of applying the subgenus rank to these clades case are threefold. Dividing the 43 valid species of Gymnotidae into six subgenera increases the utility of these named groups for researchers less knowledgeable in the fields of gymnotiform systematics or Neotropical Ichthyology. While introducing new names does create more complexity it also highlights phenotypically and ecologically distinct taxa. For example, it would benefit an ecologist to know that *Gymnotus* (*Tigre*) species are typically large-bodied (up to 500 mm) floodplain predators, whereas *Gymnotus* (*Tigrinus*) species rarely exceed one eighth that size and mostly inhabit small upland rainforest streams [4,28,66,67]. Similarly, a biogeographer may be interested to know that *Gymnotus* (*Tijax*) is an exclusively Central American taxon, while *Gymnotus* (*Pantherus*) is largely endemic to the southeastern coastal drainages of Brazil.

In all these cases, the added work of learning new names rewards researchers with a wealth of information that is phylogenetically structured, information that would otherwise remain buried in “variation” within a single genus. Finally, we offer these new subgeneric names as a more effective taxonomy to summarize the evolutionary relationships of the subfamily. Each of the subgenera in the new classification possesses a distinct suite of morphological and ecological phenotypes and each exhibits a distinctive biogeographical range. Sublimating all the diversity exhibited by these clades within a one undifferentiated genus has made these differences almost invisible to all but the most dedicated researchers. With these ideas in mind, we propose the first major revision to the Linnaean genus *Gymnotus* in its 260-year history.

### Historical biogeography

Our time-calibrated phylogeny reveals a history of multiple colonizations of major biogeographic regions, resulting in the well-documented pattern of polyphyly of local species assemblages [62,68]. The Central American and trans-Andean drainages were colonized at least twice each and the Southern Neotropics was colonized by at least five gymnotine clades in our analysis. In the latter case, these dispersal events appear to have originated in the Western Amazon and proceeded through the upper Madeira basin along the Sub-Andean foreland as described by [69]. While our data support a Western Amazon origin for several clades, *Gymnotus* (*Lamontianus*) appears to have originated in Eigenmann’s “Eastern Highlands” region on the Amazon Craton, prior to the origin of the modern trans-continental Amazon river c. 10 Ma.

### Summary

This study reviews the known diversity and phylogenetic interrelationships of electric fishes of the family Gymnotidae (Gymnotiformes), a species-rich clade distributed throughout most of the humid Neotropics. The family is currently known from 46 species, representing an increase of >400% from the 11 species listed in the seminal review of gymnotiform fishes by [34]. Species of the subfamily Gymnotinae are members of one genus *Gymnotus*, itself divided into six subgenera. Each subgenus is a clade with a readily recognized and distinctive phenotype, a unique biogeographic distribution, multiple species that have largely or entirely allopatric distributions and are diverged from one other by 10–30 million years. These six clades have been previously recognized with informal names and are here described as subgenera within the subfamily Gymnotinae. The taxonomy proposed more equitably partitions species diversity among supraspecific taxa, employs the previously vacant subgenus taxonomic rank and renders the known diversity of the family Gymnotidae more accessible to comparative study.

## Taxonomic accounts

### Electrophorinae ellis

*Electrophorus* Gill 1864 [70]

#### Type species

*E*. *electricus* (L).
*Gymnotus electricus* Linnaeus 1758 [32]
*Gymnotus tremulus* Houttuyn 1764 [71]
*Gymnotus regius* Chiaje 1847 [72]

#### Other included species

*E*. *multivalvulatus*, *E*. *varii*, *E*. *voltai*.

#### Materials examined in morphological analyses

*E*. *electricus*: USNM 225669, 511 mm, Suriname, Nickerie, Rio Corantijn, Amotopa landing; USNM 225671, Suriname, Nickerie, pool in front of Camp Hydro; USNM 228883, 1000 mm, Guyana, Essequibo; CAS 136681, Peru, Loreto Rio Ampiyacu near Pebas. *E*. *varii*: CAS 72183 (3), Ecuador, Amazon drainage, Rio Pichis near Puerto Bermudez; FMNH 103373, Ecuador, Rio Aguarico drainage, Rio Teteya; MBUCV 10064, Venezuela, Bolivar, Rio Cuyuni, Raudal de Kinotovaca, ~40 Km south of El Dorado; MBUCV 2643, Venezuela, Apure, Guaimaral, Orinoco drainage, tributario del Rio Capara; MBUCV 3463 (2), Venezuela, upper Orinoco drainage, Cano Iguapo, east of La Esmeralda; NRM 26263, Colombia, Guainia, Porto Inirida, Amazon drainage; NRM 27643, Peru, Loreto, Rio Samiria drainage, small *terra firme* tributary of Quebrada Santa Elena; NRM 27649 (3), Peru, Loreto, Rio Javari drainage, Rio Yauerana, Colonia Angamos near Cocha Palometal; UMMZ 204426, 940 mm, Bolivia, Costa Marquez, Rio Madeira drainage, Rio Itenez. *E*. *voltai*: MZUSP 19272, Brazil, Goiás, Rio Araguaia e Tocantins.

#### Diagnosis

Readily distinguishable from Gymnotinae by the following characters: larger body size, growing to >1 m, with male-biased sexual size dimorphism, males growing up to 1.5 –>2.0 m (vs. 998 mm TL in Gymnotinae), continuous addition of new vertebrae throughout life (vs. no addition in Gymnotinae), scales absent (vs. cycloid in Gymnotinae), three anatomically-separate hypaxial electric organs (vs. one in Gymnotinae), vascularized oral respiratory organ (vs. absent in Gymnotinae), thick integument with large lateral sensory pores (vs. relatively thin integument with small pores in Gymnotinae), hemal spines absent with body cavity extending to tip of tail (vs. hemal spines present and much shorter body cavity in Gymnotinae), anal-fin rays extending to tip of tail (vs. ending before tip of tail in Gymnotinae), more than 400 anal-fin rays (vs. max. 390 in Gymnotinae), eight pectoral radials and cleithrum elongate, curved (vs. seven or less pectoral radials and cleithrum short and in Gymnotinae), broad parasphenoid enveloping orbitosphenoid and pterosphenoid (vs. narrow not enveloping orbitosphenoid and pterosphenoid in Gymnotinae).

#### Description

Sexually dimorphic, with males larger at maturity. Adult size >2 m TL in males, >1 m TL in females. Adult body shape subcylindrical with a mean ratio of body width to depth of 80.8% HL. Body profile slender, body depth 49.9–63.7% total length. Head length variable, 9.6–17.9% total length. Snout length moderate, 28.6–36.0% head length. Mouth width narrow, 39.4–52.5% head length. Preanal distance long, 72.5–100% head length. Anal-fin long, 70.5–81.8% total length. Scales absent over entire body.

Gape large, extending to or beyond posterior nares. Mouth position superior, lower jaw longer than upper, rictus decurved. Chin round in lateral, dorsal profiles, fleshy and bulbous with mental electroreceptive organ overlying lower jaw. Anterior narial pore partially or entirely included within gape, in small narial fold. Anterior nares small, its diameter less than that of eye. Eye below horizontal with mouth. Circumorbital series ovoid. Premaxilla with >11 teeth disposed in two rows along outer margin, median margin curved. Maxilla-palatine articulation near tip of endopterygoid. Maxilla triangular with a curved ventral margin, length equal to >10 dentary teeth. Dentary with one row of >16 teeth, all conical. Posterodorsal and posteroventral dentary processes abuts ventral. Dentary posteroventral process shorter than or almost as long as posterodorsal, narrow distally. Dentary ventral margin lamella small, depth less than posterior process. Dentary anteroventral margin without a hook. Anguloarticular process short, extending to ventral margin of dentary. Retroarticular with an arched lamella posteriorly forming a small canal, posterior margin squared. Endopterygoid superior and inferior portions approximately equal in size, ascending process robust, straight, tip simple. Interopercle dorsal margin ascending process absent. Dorsal region of hyomandibula with four lateral foramenae, supraorbital and infraorbital nerves divided. Preopercle anteroventral notch absent, posterodorsal laterosensory ramus with one superficial pore, margin of medial shelf smooth, median shelf small, less than half width of symplectic. Opercle dorsal margin concave, posterior margin with ridges or spines.

Cranial fontanels closed in juveniles and adults. Frontal anterior margin straight, postorbital process narrow, less than two times width of supraorbital canal. Lateral ethmoid unossified. Parietal rectangular, length equal to width. Pterophenoid anteroventral process robust, extends to lateral process. Parasphenoid posterior processes robust. Prootic foramen Vp separate V2-3+VII. Adductor mandibula insertion undivided, intermusculars absent. All basibranchials unossified. Gill rakers not contacting gill bar.

Cleithrum very narrow with straight ventral margin, anterior limb short, less than 1.8 times ascending limb, without facet for insertion of muscle from supracleithrum. Postcleithrum thin, discoid or sickle shaped. Body cavity extremely long; 145–200 precaudal vertebrae in examined specimens, with this number increasing through the lifespan of the fish. Rib 5 robust along its entire extent, less than three times width of rib 6. Pectoral fin extremely broad, with 30 rays. Anal fin long, with >400 rays in adult specimens. Lateral-line dorsal rami absent in adults. Single hypaxial electric organ, extending along entire ventral margin of body.

### Gymnotinae ellis

#### Diagnosis

Readily distinguishable from Electrophorinae by the following characters: adult body size <1 m, lacking male-biased sexual size dimorphism in all species except *G*. (*Tigrinus*) *coatesi* and *G*. (*Tigrinus*) *javari* (vs. females >1 TL, males >2m in Electrophorinae), no addition of new vertebrae (vs. continuous addition throughout lifetime in Electrophorinae), cycloid scales over entire body (vs. scales absent in Electrophorinae), one hypaxial electric organ (vs. three anatomically-separate hypaxial electric organs in Electrophorinae), hemal spines present and body cavity short (vs. hemal spines absent with body cavity extending to tip of tail in Electrophorinae), anal-fin rays ending before tip of tail (vs. extending to tip of tail in Electrophorinae), anal fin with 390 or fewer rays (vs. >400 in Electrophorinae), narrow parasphenoid enveloping orbitosphenoid and Pterosphenoid (vs. broad in Electrophorinae).

#### Description

Sexually monomorphic (Except *G*. *(T*.*) javari*). Size up to 998 mm TL with adult body proportions attained at about 150 mm TL. Adult body shape subcylindrical with a mean ratio of body width to depth of 59.7%. Body profile slender, body depth 55.7–133.3% total length. Head length variable, 7.2–15.0% total length. Snout length moderate, 24.4–41.6% head length. Mouth width narrow, 27.4–58.3% head length. Preanal distance long, 45.6–188.2% head length. Anal-fin long, 62.0–92.3% total length. Cycloid or ovoid scales present on entire post-cranial portion of body from nape to caudal appendage.

Scales above lateral line variable, in 4–13 rows. Scales over anal-fin pterygiophores variable, with 4–16 rows. Gape large, extending to or beyond posterior nares. Mouth position superior, lower jaw longer than upper, rictus decurved. Chin round in lateral, dorsal profiles, fleshy and bulbous with mental electroreceptive organ overlying lower jaw. Anterior narial pore partially or entirely included within gape, in small narial fold. Anterior nares small, its diameter less than that of eye. Eye below horizontal with mouth. Circumorbital series ovoid. Premaxilla with 5–15 teeth disposed in two rows along outer margin, median margin straight or curved. Maxilla-palatine articulation near tip of endopterygoid. Maxilla rod- or paddle-shaped, narrow distally with a shorter than to almost-as-long-as ventral margin, length equal to 4–9 dentary teeth. Dentary with one row of 5–16 teeth, 0–10+ arrowhead shaped anteriorly, all others conical posteriorly. Posterodorsal and posteroventral dentary processes abuts ventral. Dentary posteroventral process shorter than or almost as long as posterodorsal, narrow distally. Dentary ventral margin lamella large or small, depth greater or less than posterior process. Dentary anteroventral margin with or without a hook. Anguloarticular process absent to large, extending to or beyond ventral margin of dentary. Retroarticular with an arched lamella posteriorly forming a small canal, posterior margin squared. Endopterygoid superior and inferior portions approximately equal in size, ascending process robust, straight or curved, tip simple or complex. Interopercle dorsal margin ascending process present or absent. Dorsal region of hyomandibula with four lateral foramenae, supraorbital and infraorbital nerves separate or combined. Preopercle anteroventral notch present or absent, posterodorsal laterosensory ramus with 1–2 superficial pores, margin of medial shelf smooth, median shelf small to large, less to greater than half width of symplectic. Opercle dorsal margin convex to concave, posterior margin smooth.

Cranial fontanels closed in juveniles and adults. Frontal anterior margin straight or rounded, postorbital process narrow to broad, less to greater than two times width of supraorbital canal. Lateral ethmoid unossified. Parietal rectangular, length equal to width. Pterophenoid anteroventral process robust or reduced, extends beyond or to lateral process. Parasphenoid posterior processes gracile or robust. Prootic foramen Vp combined with or separate from V2-3+VII. Adductor mandibula insertion undivided, intermusculars absent. All basibranchials unossified. Gill rakers not contacting gill bar. Cleithrum narrow with straight ventral margin, anterior limb long to short, greater to less than 1.8 times ascending limb, with or without facet for insertion of muscle from supracleithrum. Postcleithrum thin, discoid or sickle shaped. Body cavity variable, with 31–58 precaudal vertebrae. Rib 5 robust to broad along its entire extent, less to greater than three times width of rib 6. Displaced hemal spines absent. Pectoral fin variable, with 9–22 rays. Anal fin long, with 130–390 rays. Lateral-line complete, with 2–55 ventral rami. Lateral-line dorsal rami absent in adults. Single hypaxial electric organ, extending along entire ventral margin of body with 3–10 rows of electroplates near caudal insertion of anal fin.

#### *Gymnotus (Gymnotus)* subgen nov. (Table 5)

*Gymnotus carapo* species group, Albert & Miller, 1995
*Gymnotus carapo* species group, Albert, 2001
*Gymnotus carapo* species group, Albert & Crampton, 2003
*Gymnotus carapo* clade, Lovejoy *et al*. 2010
*G*. *carapo*-D clade, Crampton *et al*. 2013
*Gymnotus carapo* clade, Tagliacollo *et al*. 2016
*Gymnotus carapo* group, van der Sleen & Albert, 2018

**Table 5:** Summary of morphometric and meristic data for the six gymnotine subgenera recognized herein. Data for 796 specimens.

* *	*Gymnotus*				*Lamontianus*				*Pantherus*				*Tigre*				*Tigrinus*				*Tijax*			
	N	Min	Max	AVG	N	Min	Max	AVG	N	Min	Max	AVG	N	Min	Max	AVG	N	Min	Max	AVG	N	Min	Max	AVG
**TL**	504	125	419	195	68	92	337	187	35	69	234	156	46	164	998	367	121	80	220	125	16	136	236	183
**HL**	477	12.2	56.2	22.7	68	9.7	31.9	18	34	9.1	20.8	15	42	17.1	82	36.5	108	7	27.5	12.2	16	14.2	21.5	18.4
**HL%**	473	7.2	15	11.4	68	7.2	14.2	9.7	34	8.6	13.1	9.8	42	8.2	13.3	10.6	99	7.8	11.7	9.6	16	9.1	11.6	10.1
**PR%**	461	29.6	41.6	35	57	26.2	39.7	33.3	34	24.4	37.6	32.6	41	31.7	39.9	37.6	87	28.2	37.7	32.9	15	34	41.5	38.4
**MW%**	433	30.2	56.8	41.6	57	27.4	58.3	38.1	34	31.5	51.2	40.8	38	37.2	49.1	43.5	90	27.8	46.6	38.5	16	36.1	50.1	41.3
**PO%**	447	50.5	67.3	61.5	68	51.5	75	62	34	54.8	67.6	62.3	38	57.7	63.4	61.1	89	56.4	67.1	62.3	16	58.9	65.6	62.3
**IO%**	457	28.6	50.3	38.9	68	22.5	53.3	35.1	34	29.6	45.3	38.5	40	31.1	50.8	43.6	84	30.4	45.6	38.8	16	36.7	48.5	45
**BD%**	453	63.4	133.3	89.8	68	59	106.8	77.5	34	69.9	99.6	85.1	38	67.9	100.6	88.6	96	55.7	94.5	75.2	16	71.9	114.3	100.3
**BW%**	241	23.7	61.9	50.5	68	21.6	76.7	49	34	28.8	50.3	40.2	37	37.5	82	63.6	75	37.1	76.7	52.4	16	56.6	85.7	71.6
**BW/BD**	332	54.1	109.4	69.6	68	34.6	83.8	62.3	34	31	60.9	47.6	37	38.6	86.9	72.1	79	51.4	86	69	14	0	79.2	66.4
**HD%**	405	50.7	80.2	63.5	68	47.6	68.8	57.2	34	60.6	73.3	67.9	36	50.7	71.5	57.9	79	50.3	65.7	58	15	62.2	75.6	69.9
**HW%**	454	46	76.8	61.8	68	44.9	73.7	57.5	34	55.9	67.6	62.9	34	57.7	75.1	65.6	79	49.5	73.3	60.5	16	63.8	72.3	69.4
**BO%**	300	27.7	61.9	42	0	_	_	_	34	29	45.4	35.8	26	27	76.5	36.9	47	20.8	40.8	31.4	16	23.5	53.1	39.1
**PA%**	388	60.1	100.1	79.1	68	50.7	188.2	98.8	34	80.7	123.5	101.2	27	45.6	112.3	81.3	72	63.7	124.2	94.9	16	84.2	109.6	97.2
**P1%**	403	38.2	66.8	48.1	68	24.2	52.9	36.3	34	28.7	46.8	37.8	33	37.4	53.2	44.4	89	29.1	56.9	46.1	16	35.5	44.5	40.8
**AF%**	447	67.6	92.3	80.6	68	65.9	85	78.8	34	68.4	81.7	76.3	35	73	87.8	81	82	62	84.8	77.3	13	78.4	84.2	80.4
**BND**	416	0	29	20	63	15	51	22	34	0	0	0	27	13	29	23	69	11	24	14	22	0	24	0
**AFR**	280	130	322	225	60	210	312	247	35	173	232	199	26	190	390	257	61	135	245	210	11	170	270	190
**P1R**	368	10	22	16	64	9	18	13	35	14	19	17	40	13	21	19	70	12	20	14	2	15	16	16
**SAL**	324	4	10	6	64	5	13	6	34	5	8	6	34	6	13	12	76	6	9	7	6	7	11	7
**CEP**	323	3	4	3	37	5	10	9	34	3	3	3	27	3	6	4	54	3	3	3	2	3	3	3
**APS**	247	4	13	8	0	_	_	_	34	4	9	6	35	9	16	11	15	5	7	6	0	_	_	_
**PCV**	162	31	43	35	31	31	58	32	1	48	48	48	14	32	48	44	82	35	44	41	15	32	36	33
**PLR**	275	23	71	45	50	45	78	62	32	28	55	41	36	40	78	53	62	31	67	57	8	35	57	43
**PLL**	254	68	140	90	40	73	130	100	32	44	85	67	15	80	153	125	15	80	114	102	0	_	_	_
**VLR**	119	7	30	19	35	2	18	11	34	5	29	16	13	23	55	33	0	_	_	_	0	_	_	_

#### Type species

*G*. (*G*.) *carapo* (L).

#### Other included species

G. (G.) arapaima, G. (G.) ardilai, G. (G.) bahianus, G. (G.) capanema, G. (G.) eyra, G. (G.) chaviro, G. (G.) chimarrao, G. (G.) choco, G. (G.) curupira, G. (G.) cuia, G. (G.) diamantinensis, G. (G.) mamiraua, G. (G.) interruptus, G. (G.) obscurus, G. (G.) omarorum, G. (G.) pantanal, G. (G.) riberalta, G. (G.) sylvius, G. (G.) ucamara, G. (G.) varzea.

#### Diagnosis

Species of the subgenus *Gymnotus* are readily distinguishable from those of other subgenera of Gymnotinae by the following characters: anteriormost 2–7 dentary teeth anteroposteriorly compressed, resembling arrowheads (vs. conical or needle-shaped teeth in other subgenera), transparent patch in posterior 10–20% of anal fin membrane (vs. evenly pigmented or striped in other subgenera). *Gymnotus* (*Gymnotus*) is morphologically most similar to *Gymnotus (Tijax*), from which is it readily distinguishable by the following characters, a color pattern consisting of 14–29 dark, unevenly spaced, obliquely-oriented, dark pigment bands or band-pairs with wavy, low-contrast margins (except in *G*. *(G*.*) diamantinensis* and *G*. *(G*.*) bahianus* that lack pigment bands) vs. a color pattern lacking dark pigment bands, or with small, irregular dark pigment blotches (except in *G*. (*Tijax*) *panamensis*, which possesses dark pigment bands only on the posterior 33% of the body); 2, one row of premaxillary teeth vs. two. *Gymnotus* (*Gymnotus*) is readily distinguishable from morphologically similar *Gymnotus* (*Tigre*) by the following characters, color pattern lacking white blotches on head vs. color pattern with white blotches on head, ovoid-shaped scales over the whole body surface (vs. axially-elongate scales posteriorly), relatively few, long, curved ventral latera-line rami (VLR 7–30) vs. many, short, straight ventral lateral-line rami (VLR 23–55), body cavity of intermediate length (PCV 31–43) vs. long body cavity (PCV 32–48).

#### Description

Sexually monomorphic. Size up to 419 mm TL with adult body proportions attained at about 150 mm TL, or 125 mm for smaller species. Adult body shape subcylindrical with a mean ratio of body width to depth of about 70%. Body profile intermediate to deep, body depth 63.3–133.3% total length. Head length variable, 7.2–15.0% total length. Snout length moderate, 29.6–41.6% head length. Mouth width narrow, 30.2–56.8% head length. Preanal distance long, 60.1–100.1% head length. Anal-fin long, 67.6–92.3% total length. Ovoid-shaped scales present on entire post-cranial portion of body from nape to caudal appendage.

Scales above lateral line of intermediate size, in 4–10 rows. Scales over anal-fin pterygiophores large, arranged in 4–13 rows. Gape large, extending to or posterior to posterior nares. Mouth position superior, lower jaw longer than upper, rictus decurved. Chin round in lateral, dorsal profiles, fleshy and bulbous with mental electroreceptive organ overlying lower jaw. Anterior narial pore partially or entirely included within gape, in small narial fold. Anterior nares small, its diameter less than that of eye. Eye below horizontal with mouth. Circumorbital series ovoid (or tear-drop shaped in lateral view in *G*. *(G*.*) carapo occidentalis* and *G*. *(G*.*) arapaima*). Premaxilla with 8–14 teeth disposed in one row along outer margin, medial margin curved. Maxilla-palatine articulation near tip of endopterygoid. Maxilla rod- or paddle-shaped, narrow distally (except in *G*. *(G*.*) bahianus*, *G*. *(G*.*) cuia*, *G*. *(G*.*) curupira*, *G*. *(G*.*) eyra* and *G*. *(G*.*) interruptus*, where it is broad distally) with a straight ventral margin, length equal to that of 4–6 dentary teeth (except in *G*. *(G*.*) capanema*, *G*. *(G*.*) pantanal* and *G*. *(G*.*) riberalta*, where length is equal to that of 7–9 dentary teeth). Dentary with one row of 12–19 teeth, 2–8 arrowhead shaped anteriorly, all others conical posteriorly. Posterodorsal and posteroventral dentary processes abuts ventral. Dentary posteroventral process shorter than or almost as long as posterodorsal, narrow distally. Dentary ventral margin lamella large, depth greater than posterior process (except in *G*. *(G*.*) capanema*, *G*. *(G*.*) pantanal* and *G*. *(G*.*) riberalta*, where it is smaller than posterior process). Dentary anteroventral margin lacking a hook (except in *G*. *(G*.*) capanema*, *G*. *(G*.*) pantanal* and *G*. *(G*.*) riberalta*). Mandible long (except in some species with a shorter face; i.e. *G*. *(G*.*) bahianus*, *G*. *(G*.*) chimarrao*, *G*. *(G*.*) curupira*, *G*. *(G*.*) diamantinensis*, *G*. *(G*.*) interruptus*, *G*. *(G*.*) omarorum* and *G*. *(G*.*) sylvius*). Anguloarticular process extending over retroarticular, extending beyond ventral margin of dentary (except in *G*. *(G*.*) ardilai*, *G*. *(G*.*) choco* and *G*. *(G*.*) ucamara*, where it extends to the ventral margin of the dentary). Retroarticular with an arched lamella posteriorly forming a small canal, its posterior margin square. Endopterygoid superior and inferior portions approximately equal in size, endopterygoid ascending process robust, long, its base shorter than length (except in *G*. *(G*.*) cuia* and *G*. *(G*.*) omarorum*), endopterygoid ascending process straight or slightly curved, with a simple tip (except in *G*. *(G*.*) eyra* and *G*. *mamiraua* where it is divided into several smaller processes). Interopercle dorsal margin ascending process present. Dorsal region of hyomandibula with four lateral foramenae, supraorbital and infraorbital nerves connected (except in *G*. *(G*.*) capanema*, *G*. *(G*.*) pantanal* and *G*. *(G*.*) riberalta*). Preopercle anteroventral notch present (except in *G*. *(G*.*) curupira*), posterodorsal laterosensory ramus with two superficial pores, margin of medial shelf entirely smooth, median shelf large, greater than half width of symplectic. Opercle dorsal margin straight or convex, posterior margin entirely smooth. Subopercle dorsal margin concave. Cranial fontanels closed in juveniles and adults. Frontal broad or narrow, anterior margin straight, postorbital process broad or narrow. Lateral ethmoid unossified. Parietal rectangular, length equal to width. Pterosphenoid anteroventral portion robust, extending ventrally to lateral margin of parasphenoid (except in *G*. *(G*.*) capanema*, *G*. *(G*.*) pantanal* and *G*. *(G*.*) riberalta*). Parasphenoid posterior processes robust or gracile. Prootic foramen for nerve Vp separate from that of nerve V2-3+VII (except in *G*. *(G*.*) capanema*, *G*. *(G*.*) pantanal* and *G*. *(G*.*) riberalta* where the nerves have a single foramen). *M*. *adductor mandibula* intermuscular bones absent (except in some *G*. *(G*.*) carapo occidentalis* and *G*. *(G*.*) arapaima*). All basibranchials unossified. Gill rakers not contacting gill bars.

Cleithrum broad with curved ventral margin (except in *G*. *(G*.*) capanema*, *G*. *(G*.*) diamantinensis*, *G*. *(G*.*) pantanal*, *G*. *(G*.*) riberalta* and *G*. *(G*.*) sylvius*, where it is narrow with straight ventral margin), anterior limb long, greater than 1.8 times ascending limb (except in *G*. *(G*.*) bahianus*, *G*. *(G*.*) eyra*, *G*. *mamiraua* and *G*. *(G*.*) interruptus*), large or small facet for insertion of muscle from supracleithrum. Postcleithrum thin, discoid or sickle-shaped, sometimes not ossified. Body cavity of intermediate length, with 31–43 precaudal vertebrae. Rib 5 robust along its entire extent (except in *G*. *(G*.*) capanema*, *G*. *(G*.*) pantanal* and *G*. *(G*.*) riberalta*, which possess an expanded triangular medial shelf greater than three times width of rib 6). Displaced hemal spines absent. Pectoral fin variable, with 10–22 rays. Anal fin long, with 130–322 rays. Lateral line complete, with 7–30 ventral rami. Lateral-line dorsal rami absent in adults (except some *G*. *(G*.*) obscurus*). Single hypaxial electric organ extending along ventral margin of body, with 3–4 electroplate rows near caudal insertion of anal fin.

**Diagnoses and descriptions of each species of subgenus Gymnotus** (**Tables 6–12)**

### Gymnotus (Gymnotus) arapaima Albert & Crampton

#### Materials examined in morphological analyses

INPA 13505 (Holotype), 192 mm, Brazil, Amazonas, Paraná Apara, 10 km NW of confluence of Juruá and Solimões rivers, Mamirauá Reserve (03°02’S, 64°51’W); BMNH 1998.3.11 (Paratypes) (5), 126–150 mm, Brazil, Amazonas, Paraná Apara, Mamirauá lake system, Mamirauá Reserve (03°02’11”S, 64°51’19”W); BMNH 1998.3.11:141–148 (Paratypes) (8), 90–176 mm, same locality as BMNH 1998.3.11; BMNH 1998.3.11:149 (Paratype), 144 mm, Brazil, Amazonas, Cabeçiera do Lago Tefé, delta where Rio Tefe enters Lago Tefe (03°34’S, 64°58’W); BMNH 1998.3.12:2 (Paratype), 144 mm, Brazil, Amazonas, Vila Alencar, Lago Mamirauá, 3°07’49”S, 64°47’58”W); BMNH 1998.3.12:3 (Paratype), 127 mm, Brazil, Amazonas, Cano do Lago Rato, Mamirauá lake system (03°02’48”S, 64°51’22”W); BMNH 1998.3.12:4–6 (Paratypes) (3), 144–199 mm, same locality as BMNH 1998.3.12:3; INPA 9963 (Paratypes) (5), 119–179 mm, same locality as BMNH 1998.3.11; INPA 13506 (Paratypes) (3), 123–222 mm, same locality as INPA 13505; INPA 15833 (Paratypes) (14), 93–349 mm, Brazil, Amazonas, Cano do Lago Rato, Mamirauá lake system; INPA 6398, 545 mm, Brazil, Amazonas, Terra Nova, Rio Amazonas at Ilha Carreiro; MZUSP 52660, 155 mm, Brazil, Para Porto, Igarape Papagaio, Rio Trombetas.

**Table 6:** Summary of morphometric and meristic data for *Gymnotus (G*.*) arapaima*, *G*. *(G*.*) ardilai* and *G*. *(G*.*) bahianus*.

*** ***	***G*. *(G*.*) arapaima***				***G*. *(G*.*) ardilai***				***G*. *(G*.*) bahianus***			
** **	**N**	**Min**	**Max**	**AVG**	**N**	**Min**	**Max**	**AVG**	**N**	**Min**	**Max**	**AVG**
**TL**	27	126	272	171	3	219	430	326	20	129	273	187
**HL**	27	16.3	37.1	22.8	3	24.5	47.0	35.1	20	15.8	28.2	21.1
**HL%**	27	12.1	14.2	13.4	3	10.2	11.2	10.8	20	10.3	12.6	11.4
**PR%**	27	33.5	38.8	35.5	3	22.4	30.6	27.1	19	33.5	36.4	35.4
**MW%**	27	31.2	39.5	34.8	3	42.4	51.6	46.7	20	35.2	44.8	40.4
**PO%**	27	59.5	64.4	61.9	3	59.8	63.7	62.3	20	59.3	63.5	61.0
**IO%**	27	31.0	36.5	33.1	3	39.2	47.7	44.8	19	33.9	42.5	38.4
**BD%**	27	63.4	92.1	76.3	_	_	_	_	20	82.9	113.7	96.0
**BW%**	26	41.1	56.6	48.0	_	_	_	_	11	50.9	60.5	57.5
**BD/BW**	27	55.4	76.9	63.9	_	_	_	_	20	54.3	76.6	64.1
**HD%**	24	50.7	60.5	54.5	3	65.7	71.2	68.1	20	55.4	73.4	61.0
**HW%**	27	46.0	59.0	52.5	3	61.2	77.4	69.5	20	56.9	68.2	61.8
**BO%**	26	32.2	50.7	38.5	3	31.2	38.8	36.1	19	27.7	39.4	33.4
**PA%**	20	60.4	80.6	66.6	3	65.7	82.3	72.9	19	76.2	96.8	85.6
**P1%**	27	40.2	47.9	43.9	3	34.9	52.8	42.2	16	41.9	55.8	49.4
**AF%**	27	76.6	81.5	79.4	3	79.2	84.2	82.4	19	75.7	84.0	80.9
** **	**N**	**Min**	**Max**	**Mode**	**N**	**Min**	**Max**	**Mode**	**N**	**Min**	**Max**	**Mode**
**BND**	20	19	25	24	3	0	0	0	27	0	6	0
**AFR**	17	220	275	235	_	_	_	_	8	190	255	190
**P1R**	20	15	17	15	3	14	14	14	8	16	16	16
**SAL**	18	5	10	7	3	8	9	8	7	7	7	7
**CEP**	24	3	4	4	_	_	_	_	6	4	4	4
**APS**	35	9	13	12	3	9	10	10	7	8	10	9
**PCV**	14	34	37	35	1	34	34	34	12	32	35	32
**PLR**	18	45	65	52	3	47	48	48	6	40	41	40
**PLL**	17	87	108	106	1	84	84	84	6	75	82	78
**VLR**	5	14	18	16	_	_	_	_	_	_	_	_

**Table 7:** Summary of morphometric and meristic data for *Gymnotus (G*.*) capanema*, *G*. *(G*.*) carapo and G*. *(G*.*) chaviro*.

*** ***	***G*. *(G*.*) capanema***				***G*. *(G*.*) carapo***				***G*. *(G*.*) chaviro***			
** **	**N**	**Min**	**Max**	**AVG**	**N**	**Min**	**Max**	**AVG**	**N**	**Min**	**Max**	**AVG**
**TL**	8	79	166	_	173	127	419	225	91	125	275	160
**HL**	8	7.9	16.3	_	168	13.4	56.2	29.1	72	12.2	32.0	16.1
**HL%**	2	8.5	9.3	8.9	164	10.2	15.0	13.5	72	8.8	13.6	10.1
**PR%**	8	36.4	49.8	42.2	164	29.6	40.1	35.7	72	32.2	39.8	35.4
**MW%**	8	40.6	58.0	48.0	147	32.3	49.0	41.2	71	35.5	52.8	44.1
**PO%**	8	56.4	69.0	63.3	164	52.7	66.9	62.4	72	58.4	67.3	63.2
**IO%**	8	41.7	47.7	43.6	165	29.1	44.8	37.9	72	37.3	50.3	42.5
**BD%**	_	_	_	_	163	63.7	106.6	84.8	71	64.9	109.7	88.8
**BW%**	_	_	_	_	61	47.8	61.9	57.4	12	55.4	61.6	60.2
**BD/BW**	2	61.0	65.0	63.0	63	57.4	82.6	69.5	71	64.5	109.4	77.6
**HD%**	8	49.2	72.0	62.5	120	50.8	72.4	59.5	71	60.1	78.1	70.5
**HW%**	8	60.7	72.3	66.3	166	49.1	67.7	60.0	72	55.9	74.7	69.1
**BO%**	8	27.5	39.4	33.7	124	28.1	57.2	39.1	4	33.3	36.0	34.5
**PA%**	8	77.6	125.0	94.5	119	60.3	99.2	76.5	71	61.3	99.4	82.0
**P1%**	6	37.8	53.7	46.1	117	38.6	58.0	46.2	72	45.3	66.8	55.2
**AF%**	2	80.6	82.7	81.7	153	67.6	92.3	79.4	72	80.2	86.2	83.7
** **	**N**	**Min**	**Max**	**Mode**	**N**	**Min**	**Max**	**Mode**	**N**	**Min**	**Max**	**Mode**
**BND**	1	21	21	21	97	16	28	23	100	17	29	20
**AFR**	2	180	205	193	69	173	290	220	28	212	280	250
**P1R**	8	12	15	15	113	13	17	16	37	16	19	19
**SAL**	8	6	8	7	83	5	8	7	29	5	9	8
**CEP**	3	3	4	3	95	3	4	4	36	3	4	4
**APS**	8	5	8	8	53	6	12	9	20	12	13	13
**PCV**	2	36	37	37	53	32	43	33	4	35	38	37
**PLR**	8	47	56	50	94	35	64	45	15	43	61	49
**PLL**	8	70	109	87	80	68	128	98	12	86	107	98
**VLR**	_	_	_	_	3	18	21	19	3	18	23	18

**Table 8:** Summary of morphometric and meristic data for *Gymnotus (G*.*) chimarrao*, *G*. *(G*.*) choco and G*. *(G*.*) cuia*.

*** ***	***G*. *(G*.*) chimarrao***				***G*. *(G*.*) choco***				***G*. *(G*.*) cuia***			
** **	**N**	**Min**	**Max**	**AVG**	**N**	**Min**	**Max**	**AVG**	**N**	**Min**	**Max**	**AVG**
**TL**	8	111	243	194	8	165	260	208	56	125	305	199
**HL**	8	12.5	23.6	20.2	8	21.7	33.1	25.6	56	14.2	30.2	21.6
**HL%**	8	9.7	11.3	10.6	8	11.1	13.3	12.3	56	9.8	12.2	10.9
**PR%**	8	31.7	41.6	37.3	8	31.9	35.9	34.6	54	29.9	38.3	34.2
**MW%**	8	33.8	46.9	40.4	7	31.3	37.2	33.5	46	37.5	52.7	44.9
**PO%**	8	59.2	62.2	60.8	7	57.3	60.4	58.6	46	54.9	83.8	61.0
**IO%**	8	37.3	43.3	40.4	8	28.6	31.9	30.1	54	23.3	47.8	41.8
**BD%**	8	68.3	99.7	87.2	8	68.9	93.3	81.1	46	94.5	130.5	110.6
**BW%**	8	25.3	46.5	33.4	4	57.4	59.2	58.7	46	29.4	77.9	51.3
**BD/BW**	8	26.6	56.7	39.2	8	68.4	83.3	76.9	46	27.6	72.4	46.8
**HD%**	8	61.9	71.6	65.3	8	54.4	59.6	56.6	46	61.9	80.2	68.5
**HW%**	8	58.5	66.2	60.8	8	53.9	62.2	58.5	46	46.2	71.9	62.2
**BO%**	5	33.9	42.4	38.8	8	38.4	43.8	41.8	44	34.3	64.9	50.2
**PA%**	7	68.5	87.1	78.5	8	76.7	90.7	83.5	46	51.2	95.7	78.4
**P1%**	7	43.0	50.4	46.9	7	43.7	55.4	48.6	45	41.7	53.7	45.3
**AF%**	8	76.6	83.8	81.3	8	75.8	83.8	80.8	46	76.3	92.3	83.5
** **	**N**	**Min**	**Max**	**Mode**	**N**	**Min**	**Max**	**Mode**	**N**	**Min**	**Max**	**Mode**
**BND**	7	29	45	32	8	18	22	21	43	21	29	28
**AFR**	7	191	250	210	5	210	255	235	41	141	259	192
**P1R**	7	14	17	15	9	14	16	14	44	11	16	14
**SAL**	7	6	8	7	7	6	6	6	45	5	7	6
**CEP**	5	3	3	3	6	3	3	3	27	3	3	3
**APS**	5	9	13	9	3	8	9	8	42	6	10	8
**PCV**	_	_	_	_	9	32	35	35	3	31	34	33
**PLR**	7	30	44	40	7	45	52	45	43	32	47	37
**PLL**	7	73	106	96	_	_	_	_	39	80	103	85
**VLR**	5	13	24	23	_	_	_	_	40	14	28	22

**Table 9:** Summary of morphometric and meristic data for *Gymnotus (G*.*) curupira*, *G*. *(G*.*) diamantinensis and G*. *(G*.*) eyra*.

*** ***	***G*. *(G*.*) curupira***				***G*. *(G*.*) diamantinensis***				***G*. *(G*.*) eyra***			
** **	**N**	**Min**	**Max**	**AVG**	**N**	**Min**	**Max**	**AVG**	**N**	**Min**	**Max**	**AVG**
**TL**	10	135	235	170	3	125	278	208	19	115	254	148
**HL**	9	13.5	21.0	16.5	3	13.5	33.1	26.0	19	11.3	24.1	15.2
**HL%**	9	8.8	10.5	9.7	3	10.8	14.3	12.3	19	8.9	12.3	10.2
**PR%**	8	34.3	36.0	35.3	2	35.0	35.2	35.1	18	32.0	39.9	34.8
**MW%**	9	41.4	56.8	48.8	2	39.0	44.1	41.6	18	30.9	43.9	37.9
**PO%**	7	59.3	66.2	63.2	2	58.0	58.1	58.1	18	58.3	112.2	65.0
**IO%**	6	42.1	44.5	42.8	2	37.5	38.1	37.8	18	31.2	43.7	37.8
**BD%**	9	73.0	96.4	83.3	2	80.3	84.0	82.2	18	85.4	114.5	94.8
**BW%**	5	56.7	61.7	59.5	2	52.7	60.7	56.7	17	46.1	65.0	52.8
**BD/BW**	9	68.1	82.9	75.8	2	65.6	72.3	69.0	17	48.4	62.8	55.4
**HD%**	9	58.7	70.2	64.9	2	55.2	59.8	57.5	18	61.9	71.9	67.6
**HW%**	7	64.0	72.7	69.0	2	53.3	58.3	55.8	18	57.0	63.9	60.5
**BO%**	7	29.1	39.7	33.2	_	_	_	_	18	43.8	58.5	50.2
**PA%**	5	74.3	98.0	85.4	2	72.7	74.0	73.4	18	61.3	84.5	72.8
**P1%**	9	42.2	55.0	48.2	2	40.3	42.9	41.6	18	39.2	50.7	46.0
**AF%**	9	78.7	83.8	81.4	2	76.8	78.9	77.9	18	78.5	84.9	82.0
** **	**N**	**Min**	**Max**	**Mode**	**N**	**Min**	**Max**	**Mode**	**N**	**Min**	**Max**	**Mode**
**BND**	15	14	20	20	2	21	22	22	17	16	23	20
**AFR**	11	230	322	260	2	194	211	203	18	155	227	193
**P1R**	16	16	17	16	2	14	15	15	18	10	13	11
**SAL**	15	5	8	6	4	5	7	7	18	4	6	5
**CEP**	15	3	3	3	3	3	3	3	18	3	3	3
**APS**	4	7	8	8	1	9	9	9	17	6	8	7
**PCV**	15	34	36	35	2	33	35	34	3	32	34	33
**PLR**	4	59	62	61	3	44	49	48	16	30	41	39
**PLL**	12	104	140	111	1	71	71	71	16	74	95	83
**VLR**	_	_	_	_	_	_	_	_	16	10	15	13

**Table 10:** Summary of morphometric and meristic data for *Gymnotus (G*.*) interruptus*, *G*. *(G*.*) mamiraua and G*. *(G*.*) obscurus*.

*** ***	***G*. *(G*.*) interruptus***				***G*. *(G*.*) mamiraua***				***G*. *(G*.*) obscurus***			
** **	**N**	**Min**	**Max**	**AVG**	**N**	**Min**	**Max**	**AVG**	**N**	**Min**	**Max**	**AVG**
**TL**	3	80	121	97	19	115	254	148	12	141	215	166
**HL**	3	11.4	15.5	13.0	19	11.3	24.1	15.2	12	15.5	21.4	17.9
**HL%**	3	12.8	14.3	13.5	19	8.9	12.3	10.2	12	9.9	12.4	10.9
**PR%**	_	_	_	_	18	32.0	39.9	34.8	12	34.1	39.9	37.6
**MW%**	_	_	_	_	18	30.9	43.9	37.9	12	37.3	46.3	42.8
**PO%**	3	62.1	63.3	62.9	18	58.3	112.2	65.0	12	59.9	64.5	62.1
**IO%**	3	41.2	42.7	41.8	18	31.2	43.7	37.8	12	36.3	43.3	39.9
**BD%**	_	_	_	_	18	85.4	114.5	94.8	11	82.5	98.8	89.4
**BW%**	_	_	_	_	17	46.1	65.0	52.8	11	50.3	60.2	55.6
**BD/BW**	_	_	_	_	17	48.4	62.8	55.4	10	57.1	68.4	63.6
**HD%**	3	59.6	61.6	60.6	18	61.9	71.9	67.6	12	59.4	64.3	62.4
**HW%**	_	_	_	_	18	57.0	63.9	60.5	11	55.9	68.3	62.5
**BO%**	3	40.0	44.5	42.2	18	43.8	58.5	50.2	5	33.2	36.5	35.2
**PA%**	_	_	_	_	18	61.3	84.5	72.8	12	71.3	94.2	80.3
**P1%**	3	35.7	44.5	41.4	18	39.2	50.7	46.0	12	40.6	55.0	49.1
**AF%**	3	80.8	82.0	81.2	18	78.5	84.9	82.0	12	77.0	83.8	81.6
** **	**N**	**Min**	**Max**	**Mode**	**N**	**Min**	**Max**	**Mode**	**N**	**Min**	**Max**	**Mode**
**BND**	3	22	26	24	17	16	23	20	15	14	20	19
**AFR**	1	219	219	219	18	155	227	193	11	208	250	230
**P1R**	1	17	17	17	18	10	13	11	16	20	22	21
**SAL**	3	9	9	9	18	4	6	5	15	4	7	6
**CEP**	_	_	_	_	18	3	3	3	14	3	4	4
**APS**	_	_	_	_	17	6	8	7	6	6	7	6
**PCV**	1	33	33	33	3	32	34	33	16	35	37	35
**PLR**	3	37	40	37	16	30	41	39	6	50	56	54
**PLL**	3	82	97	83	16	74	95	83	11	110	120	110
**VLR**	3	23	28	26	16	10	15	13	_	_	_	_

**Table 11:** Summary of morphometric and meristic data for *Gymnotus (G*.*) omrorum*, *G*. *(G*.*) pantanal and G*. *(G*.*) riberalta*.

*** ***	***G*. *(G*.*) omarorum***				***G*. *(G*.*) pantanal***				***G*. *(G*.*) riberalta***			
** **	**N**	**Min**	**Max**	**AVG**	**N**	**Min**	**Max**	**AVG**	**N**	**Min**	**Max**	**AVG**
**TL**	11	169	262	215	18	144	251	192	23	135	302	193
**HL**	12	19.5	28.5	24.3	18	15.2	24.7	18.9	10	15.4	21.7	18.1
**HL%**	11	10.6	12.4	11.5	18	7.2	11.7	8.9	10	7.3	10.3	9.0
**PR%**	12	34.7	38.7	36.7	18	30.7	38.9	34.9	10	31.0	37.3	34.9
**MW%**	12	37.4	48.9	43.1	18	42.0	50.7	46.0	10	32.7	45.7	41.2
**PO%**	12	53.9	59.3	56.8	18	50.5	63.1	58.4	10	57.4	66.7	61.8
**IO%**	11	35.1	43.6	39.7	18	37.2	46.4	41.7	10	34.3	42.6	39.1
**BD%**	12	84.5	102.3	93.9	18	77.7	111.7	92.2	10	81.8	102.1	94.4
**BW%**	11	26.1	35.5	31.4	3	71.5	76.7	73.7	10	53.3	72.0	61.2
**BD/BW**	11	27.9	37.2	33.1	3	73.2	80.5	75.7	10	55.7	73.3	65.0
**HD%**	12	64.0	70.1	67.2	18	59.4	71.2	65.6	10	58.1	71.2	64.2
**HW%**	12	57.3	66.3	61.6	18	62.2	76.8	69.8	10	54.7	74.7	61.7
**BO%**	12	48.9	60.9	52.9	17	39.7	59.3	52.4	10	42.9	56.7	48.6
**PA%**	12	67.8	89.3	80.8	18	81.7	94.8	86.4	10	71.9	97.9	81.4
**P1%**	12	40.4	47.7	43.6	18	44.5	53.3	48.4	10	42.1	52.0	47.6
**AF%**	11	81.6	85.9	82.9	18	78.1	84.2	81.5	10	79.7	97.4	83.6
** **	**N**	**Min**	**Max**	**Mode**	**N**	**Min**	**Max**	**Mode**	**N**	**Min**	**Max**	**Mode**
**BND**	11	24	29	29	16	21	24	22	10	22	27	24
**AFR**	11	130	184	166	18	131	280	187	10	169	240	205
**P1R**	12	13	14	14	18	14	18	15	9	10	13	13
**SAL**	12	5	7	6	18	6	8	6	10	6	8	6
**CEP**	12	3	3	3	18	3	3	3	10	3	3	3
**APS**	12	5	6	6	16	7	8	7	9	10	11	10
**PCV**	_	_	_	_	4	37	38	37	1	36	36	36
**PLR**	11	23	30	27	18	45	50	46	9	45	57	52
**PLL**	10	70	87	78	16	73	94	85	8	90	103	95
**VLR**	10	28	30	29	13	19	23	20	8	10	16	13

**Table 12:** Summary of morphometric and meristic data for *Gymnotus (G*.*) sylvius*, *G*. *(G*.*) ucamara and G*. *(G*.*) varzea*.

*** ***	***G*. *(G*.*) sylvius***				***G*. *(G*.*) ucamara***				***G*. *(G*.*) varzea***			
** **	**N**	**Min**	**Max**	**AVG**	**N**	**Min**	**Max**	**AVG**	**N**	**Min**	**Max**	**AVG**
**TL**	14	81	291	193	4	136	188	162	28	126	237	162
**HL**	14	10.9	38.5	25.4	4	17.3	22.0	19.9	26	12.6	21.3	15.7
**HL%**	14	10.7	14.1	13.3	4	11.7	12.7	12.3	26	9.0	10.4	9.7
**PR%**	8	30.2	36.1	34.2	4	34.1	38.6	35.5	26	29.7	35.8	33.6
**MW%**	9	30.2	42.7	38.3	4	37.0	46.4	40.4	25	35.0	50.3	40.8
**PO%**	8	57.8	62.4	59.6	4	60.0	62.4	61.4	25	58.7	66.3	62.3
**IO%**	8	30.5	39.2	36.7	4	34.7	40.5	36.9	25	36.2	44.3	39.9
**BD%**	9	79.4	100.5	86.4	4	78.0	90.0	86.5	26	87.8	109.2	97.6
**BW%**	8	26.6	60.4	40.5	4	48.9	60.7	56.8	17	43.8	61.5	54.7
**BD/BW**	8	33.6	70.2	47.5	4	55.0	75.6	66.0	23	54.1	69.5	61.0
**HD%**	8	53.9	63.1	59.3	4	56.4	63.2	59.8	25	59.9	72.2	65.9
**HW%**	8	52.2	64.3	58.3	4	53.2	63.2	57.8	26	51.1	73.6	62.3
**BO%**	8	40.4	57.8	50.3	1	35.8	35.8	35.8	21	29.5	44.4	37.3
**PA%**	5	60.3	68.1	64.3	3	60.1	70.5	65.5	23	68.3	100.1	87.8
**P1%**	11	41.1	75.9	51.3	4	47.9	50.3	49.1	25	38.2	57.5	49.6
**AF%**	9	76.0	85.7	79.3	4	81.4	82.4	81.9	26	75.8	84.2	81.6
** **	**N**	**Min**	**Max**	**Mode**	**N**	**Min**	**Max**	**Mode**	**N**	**Min**	**Max**	**Mode**
**BND**	5	25	28	25	1	21	21	21	29	14	22	18
**AFR**	5	165	189	175	1	215	215	215	20	230	310	250
**P1R**	7	15	16	16	1	15	15	15	31	16	19	18
**SAL**	5	7	7	7	1	7	7	7	30	5	9	6
**CEP**	5	3	4	3	1	3	3	3	28	3	4	4
**APS**	7	6	9	8	_	_	_	_	10	4	6	6
**PCV**	_	_	_	_	1	33	33	33	25	35	40	37
**PLR**	5	42	44	43	1	44	44	44	12	51	71	53
**PLL**	7	79	100	85	_	_	_	_	14	106	135	124
**VLR**	5	19	25	22	_	_	_	_	_	_	_	_

#### Diagnosis

*Gymnotus (Gymnotus) arapaima* can be differentiated from all other members of the subgenus except *G*. *(G*.*) carapo*, *G*. *(G*.*) sylvius* and *G*. *(G*.*) ucamara* on the basis of the following characters: long head (HL 12.1–14.2% TL vs. 10.3–12.6% in *G*. *(G*.*) bahianus*, 8.8–11.7% in *G*. *(G*.*) chaviro*, 9.7–10.7% in *G*. *(G*.*) chimarrao*, 11.1–12.8% in *G*. *(G*.*) choco*, 9.9–12.2% in *G*. *(G*.*) cuia*, 8.8–10.5% in *G*. *(G*.*) curupira*, 10.8–11.9% in *G*. *(G*.*) diamantinensis*, 8.9–12.3% in *G*. *(G*.*) eyra*, 9.6–11.9% in *G*. *(G*.*) mamiraua*, 9.9–11.3% in *G*. *(G*.*) obscurus*, 10.6–12.4% in *G*. *(G*.*) omarorum*, 7.2–11.7% in *G*. *(G*.*) pantanal*, 7.3–10.3% in *G*. *(G*.*) riberalta*, 9.1–10.3% in *G*. *(G*.*) varzea*). *Gymnotus (Gymnotus) arapaima* can be differentiated from *G*. *(G*.*) carapo*, *G*. *(G*.*) sylvius* and *G*. *(G*.*) ucamara* on the basis of the following characters: color pattern with reverse-countershading (lighter coloration dorsally grading to darker coloration ventrally) anterodorsally with a metallic blue or gunmetal colored sheen visible on live and recently-preserved specimens (vs. no countershading or typical countershading (darker coloration dorsally grading to lighter coloration ventrally) in all other *Gymnotus*).

#### Description

Sexually monomorphic. Size up to 272 mm TL with adult body proportions attained at about 150 mm TL. Adult body shape subcylindrical with a mean ratio of body width to depth of 63.9%. Body profile slender, body depth 63.4–92.1% total length. Head length long, 21.1–14.2% total length. Snout length moderate, 33.5–38.8% head length. Mouth width narrow, 31.2–39.5% head length. Preanal distance long, 60.4–80.6%head length. Anal-fin long, 76.6–81.5% total length. Cycloid or ovoid scales present on entire post-cranial portion of body from nape to caudal appendage.

Scales above lateral line intermediate, in 5–10 rows. Scales over anal-fin pterygiophores large, with 9–13 rows. Gape large, extending to or beyond posterior nares. Mouth position superior, lower jaw longer than upper, rictus decurved. Chin round in lateral, dorsal profiles, fleshy and bulbous with mental electroreceptive organ overlying lower jaw. Anterior narial pore partially or entirely included within gape, in small narial fold. Anterior nares small, its diameter less than that of eye. Eye below horizontal with mouth. Circumorbital series ovoid. Premaxilla with >11 teeth disposed in two rows along outer margin, median margin curved. Maxilla-palatine articulation position near tip of endopterygoid. Maxilla rod- or paddle-shaped, broad distally with a curved ventral margin, length equal to 4–6 dentary teeth. Dentary with one row of >16 teeth, >8 arrowhead shaped anteriorly, all others conical posteriorly. Posterodorsal and posteroventral dentary processes abuts ventral. Dentary posteroventral process shorter than or almost as long as posterodorsal, narrow distally. Dentary ventral margin lamella small, depth less than posterior process. Dentary anteroventral margin without a hook. Mandible long.

Anguloarticular process long, extending over ventral margin of dentary. Retroarticular with an arched lamella posteriorly forming a small canal, posterior margin squared. Endopterygoid superior and inferior portions approximately equal in size, ascending process robust, long, base>length, tip simple. Interopercle dorsal margin ascending process present. Dorsal region of hyomandibula with four lateral foramenae, supraorbital and infraorbital nerves divided. Preopercle anteroventral notch present, posterodorsal laterosensory ramus with 2 superficial pores, margin of medial shelf smooth, median shelf large, greater than half width of symplectic. Opercle dorsal margin straight or convex, posterior margin entirely smooth. Subopercle dorsal margin concave. Cranial fontanels closed in juveniles and adults. Frontal anterior margin straight, postorbital process large, greater than two times width of supraorbital canal. Lateral ethmoid unossified. Parietal rectangular, length equal to width. Pterophenoid anteroventral process robust, extends ventrally to lateral process. Parasphenoid posterior processes gracile, elongate. Prootic foramen Vp separate V2-3+VII. Adductor mandibula insertion undivided, intermusculars absent. All basibranchials unossified. Gill rakers not contacting gill bar.

Cleithrum broad with curved ventral margin, anterior limb long, greater than 1.8 times ascending limb, small facet for insertion of muscle from supracleithrum. Postcleithrum thin, discoid or sickle shaped. Body cavity long, with 34–37 precaudal vertebrae. Rib 5 robust along its entire extent, less than three times width of rib 6. Displaced hemal spines absent. Pectoral fin broad, with 15–17 rays. Anal fin long, with 220–275 rays. Lateral-line complete, with 14–18 ventral rami. Lateral-line dorsal rami absent in adults. Single hypaxial electric organ, extending along entire ventral margin of body with 3–4 rows of electroplates near caudal insertion of anal fin.

### *Gymnotus* (*Gymnotus*) *ardilai* Maldonado-Ocampo & Albert

#### Materials examined in morphological analyses

IAvHP 3477 (Holotype), 430 mm, Colombia, Santander, Girón, Río de Oro; IAvHP 4001 (Paratypes) (2), 219–329 mm, same locality as IAvHP 3477.

#### Diagnosis

*Gymnotus (Gymnotus) ardilai* can be differentiated from all other members of the subgenus on the basis of the following characters: color pattern with dark band pairs present over entire body in juveniles and subadults (TL <250 mm), but absent anteriorly in large adults (TL >350 mm) (vs. always banded or spotted in all other *Gymnotus*).

#### Description

Sexually monomorphic. Size up to 272 mm TL with adult body proportions attained at about 150 mm TL. Head length intermediate, 10.2–11.2% total length. Snout length moderate, 22.4–30.6% head length. Mouth width narrow, 42.4–51.6% head length. Preanal distance long, 65.7–82.3%head length. Anal-fin long, 79.2–84.2% total length. Cycloid or ovoid scales present on entire post-cranial portion of body from nape to caudal appendage.

Scales above lateral line intermediate, in 8–9 rows. Scales over anal-fin pterygiophores large, with 9–10 rows. Gape large, extending to or beyond posterior nares. Mouth position superior, lower jaw longer than upper, rictus decurved. Chin round in lateral, dorsal profiles, fleshy and bulbous with mental electroreceptive organ overlying lower jaw. Anterior narial pore partially or entirely included within gape, in small narial fold. Anterior nares small, its diameter less than that of eye. Eye below horizontal with mouth. Circumorbital series ovoid. Premaxilla with >11 teeth disposed in two rows along outer margin, median margin curved. Maxilla-palatine articulation near tip of endopterygoid. Maxilla vertical, narrow distally with a straight ventral margin, length equal to 4–6 dentary teeth. Dentary with one row of >16 teeth, 4–7 arrowhead shaped anteriorly, all others conical posteriorly. Posterodorsal and posteroventral dentary processes abuts ventral. Dentary posteroventral process shorter than or almost as long as posterodorsal, narrow distally. Dentary ventral margin lamella small, depth less than posterior process. Dentary anteroventral margin without a hook. Mandible long. Anguloarticular process short, extending to ventral margin of dentary. Retroarticular with an arched lamella posteriorly forming a small canal, posterior margin squared. Endopterygoid superior and inferior portions approximately equal in size, ascending process robust, curved, tip simple. Interopercle dorsal margin ascending process present. Dorsal region of hyomandibula with four lateral foramenae, supraorbital and infraorbital nerves divided. Preopercle anteroventral notch present, posterodorsal laterosensory ramus with 2 superficial pores, margin of medial shelf entirely smooth, median shelf large, greater than half width of symplectic. Opercle dorsal margin straight, posterior margin smooth. Subopercle dorsal margin concave. Cranial fontanels closed in juveniles and adults. Frontal anterior margin straight, postorbital process broad, greater than two times width of supraorbital canal. Lateral ethmoid unossified. Parietal rectangular, length equal to width. Pterophenoid anteroventral process robust, extends ventral to lateral process. Parasphenoid posterior processes gracile, elongate. Prootic foramen Vp separate from V2-3+VII. Adductor mandibula insertion undivided, intermusculars absent. All basibranchials unossified. Gill rakers not contacting gill bar.

Cleithrum broad with curved ventral margin, anterior limb long, greater than 1.8 times ascending limb, absent facet for insertion of muscle from supracleithrum. Postcleithrum thin, discoid or sickle shaped. Body cavity long, with 34 precaudal vertebrae. Rib 5 robust along its entire extent, less than three times width of rib 6. Displaced hemal spines absent. Pectoral fin intermediate, with 14 rays. Lateral-line complete, with 84 ventral rami. Lateral-line dorsal rami absent in adults.

### *Gymnotus (Gymnotus) bahianus* Campos-da-Paz & Costa

#### Materials examined in morphological analyses

MNRJ 12316 (Holotype), 177 mm, Brazil, Bahia, fazenda Almada, Ilheus, Rio Almada basin (~14°49’S, 39°02’W); MCP 18311 (Paratypes) (2), 108–84 mm, same locality as MNRJ 12316; MNRJ 4346 (Paratypes) (33), 47–241 mm, same locality as MNRJ 12316; MZUSP 48949 (Paratypes) (2), 89–108 mm, same locality as MNRJ 12316; USNM 338274 (Paratypes) (2), 89–105 mm, same locality as MNRJ 12316; MNRJ 4188 (2), 200–208 mm, Brazil, Bahia, fazenda Almada, Ilheus; MNRJ 4381, 107 mm, Brazil, Bahia, Uarucutuca, Ilheus; MNRJ 4382 (10), 10specimens, 57.0–276.0 mm TL, Pirataquice, Ilheus.

#### Diagnosis

*Gymnotus (Gymnotus) bahianus* can be differentiated from all other members of the subgenus except *G*. *(G*.*) interruptus* on the basis of the following characters: color pattern with dark band pairs replaced by very small (1–2 scales across), rounded dark spots over entire body of most specimens, except for posteroventral 25% of some specimens (vs. banded or with larger (3 or more scales across), irregularly-shaped spots in all other *Gymnotus*). *Gymnotus (Gymnotus) bahianus* can be differentiated from *G*. *(G*.*) interruptus* on the basis of the following characters: short interorbital distance (IO 33.9–42.5% HL vs. 44.6–45.9% HL in *G*. *(G*.*) interruptus*), few scales above lateral line (SAL 7 vs. 9 in *G*. *(G*.*) interruptus*).

#### Description

Sexually monomorphic. Size up to 273 mm TL with adult body proportions attained at about 150 mm TL. Adult body shape subcylindrical with a mean ratio of body width to depth of 64.1%. Body profile slender, body depth 82.9–113.7% total length. Head length short, 10.3–12.6% total length. Snout length moderate, 33.5–36.4% head length. Mouth width narrow, 35.2–44.8% head length. Preanal distance long, 76.2–96.8%head length. Anal-fin long, 75.7–84.0% total length. Cycloid or ovoid scales present on entire post-cranial portion of body from nape to caudal appendage.

Scales above lateral line intermediate, in 7 rows. Scales over anal-fin pterygiophores large, with 7–8 rows. Gape large, extending to or beyond posterior nares. Mouth position superior, lower jaw longer than upper, rictus decurved. Chin round in lateral, dorsal profiles, fleshy and bulbous with mental electroreceptive organ overlying lower jaw. Anterior narial pore partially or entirely included within gape, in small narial fold. Anterior nares small, its diameter less than that of eye. Eye below horizontal with mouth. Circumorbital series ovoid. Premaxilla with >11 teeth disposed in two rows along outer margin, median margin curved. Maxilla-palatine articulation near tip of endopterygoid. Maxilla rod- or paddle-shaped, narrow distally with a straight ventral margin, length equal to 4–6 dentary teeth. Dentary with one row of 12–15 teeth, 4–7 arrowhead shaped anteriorly, all others conical posteriorly. Posterodorsal and posteroventral dentary processes abuts ventral. Dentary posteroventral process shorter than or almost as long as posterodorsal, narrow distally. Dentary ventral margin lamella large, depth greater than posterior process. Dentary anteroventral margin without a hook. Mandible short. Anguloarticular process long, extending over ventral margin of dentary. Retroarticular with an arched lamella posteriorly forming a small canal, posterior margin squared. Endopterygoid superior and inferior portions approximately equal in size, ascending process robust, long, tip simple. Interopercle dorsal margin ascending process present. Dorsal region of hyomandibula with four lateral foramenae, supraorbital and infraorbital nerves divided. Preopercle anteroventral notch present, posterodorsal laterosensory ramus with 2 superficial pores, margin of medial shelf smooth, median shelf large, greater than half width of symplectic. Opercle dorsal margin straight, posterior margin smooth. Subopercle dorsal margin concave. Cranial fontanels closed in juveniles and adults. Frontal anterior margin straight, postorbital process broad, greater than two times width of supraorbital canal. Lateral ethmoid unossified. Parietal rectangular, length equal to width. Pterophenoid anteroventral process robust, extends ventral to lateral process. Parasphenoid posterior processes robust. Prootic foramen Vp divided V2-3+VII. Adductor mandibula insertion undivided, intermusculars absent. All basibranchials unossified. Gill rakers not contacting gill bar.

Cleithrum broad with curved ventral margin, anterior limb short, less than 1.8 times ascending limb, without facet for insertion of muscle from supracleithrum. Postcleithrum thin, discoid or sickle shaped. Body cavity long, with 32–35 precaudal vertebrae. Rib 5 robust along its entire extent, less than three times width of rib 6. Displaced hemal spines absent. Pectoral fin intermediate, with 16–16 rays. Anal fin long, with 190–255 rays. Lateral-line complete. Lateral-line dorsal rami absent in adults. Single hypaxial electric organ, extending along entire ventral margin of body with 4 rows of electroplates near caudal insertion of anal fin.

### *Gymnotus* (*Gymnotus*) *capanema* Milhomem, Crampton, Pieczarka, Shetka, Silva & Nagamachi

#### Materials examined in morphological analyses

MPEG 15170 (Holotype), 179 mm, Brazil, Pará, Capanema, Açaiteuazinho River (01°07’54”S, 047°03’53”W); MPEG 15171 (Paratypes) (8), 79–166 mm, same locality as MPEG 15170; MPEG 4260, 142 m, Brazil, Pará, Santa Isabel do Pará; MPEG 18594 (2), 145–160 mm, LT), Brazil, Pará, Bragança, tributary of Rio Caeté (01°04’43”S, 046°44’21”W).

#### Diagnosis

*Gymnotus (Gymnotus) capanema* can be differentiated from all other members of the subgenus except *G*. *(G*.*) pantanal* and *G*. *(G*.*) riberalta* on the basis of the following characters: color pattern with wide (>5X width of interbands) dark bands, where pale interbands restricted to the ventral part of the lateral surface such that the dark interbands fuse into a uniform dark coloration over anterior 60% of body (vs. banded or spotted in all other *Gymnotus*). *Gymnotus (Gymnotus) capanema* can be differentiated from *G*. *(G*.*) pantanal* and *G*. *(G*.*) riberalta* on the basis of the following characters: pectoral fin possessing an intermediate number of rays (P1R 12–15 in *G*. *(G*.*) capanema* vs. 14–18 in *G*. *(G*.*) pantanal*, 10–13 in *G*. *(G*.*) riberalta*).

#### Description

Sexually monomorphic. Size up to 166 mm TL with adult body proportions attained at about 150 mm TL. Adult body shape subcylindrical with a mean ratio of body width to depth of 63.0%. Head length short, 8.5–9.3% total length. Snout length moderate, 36.4–39.8% head length. Mouth width narrow, 40.6–58.0% head length. Preanal distance long, 77.6–125%head length. Anal-fin long, 80.6–82.7% total length. Cycloid or ovoid scales present on entire post-cranial portion of body from nape to caudal appendage.

Scales above lateral line intermediate, in 6–8 rows. Scales over anal-fin pterygiophores large, with 5–8 rows. Gape large, extending to or beyond posterior nares. Mouth position superior, lower jaw longer than upper, rictus decurved. Chin round in lateral, dorsal profiles, fleshy and bulbous with mental electroreceptive organ overlying lower jaw. Anterior narial pore partially or entirely included within gape, in small narial fold. Anterior nares small, its diameter less than that of eye. Eye below horizontal with mouth. Circumorbital series ovoid. Premaxilla with >11 teeth disposed in two rows along outer margin, median margin curved. Maxilla-palatine articulation near tip of endopterygoid. Maxilla vertical, narrow distally with a straight ventral margin, length equal to 7–9 dentary teeth. Dentary with one row of 12 teeth, 2–4 arrowhead shaped anteriorly, all others conical posteriorly. Posterodorsal and posteroventral dentary processes abuts ventral. Dentary posteroventral process shorter than or almost as long as posterodorsal, narrow distally. Dentary ventral margin lamella small, depth less than posterior process. Dentary anteroventral margin without a hook. Anguloarticular process long, extending beyond ventral margin of dentary. Retroarticular with an arched lamella posteriorly forming a small canal, posterior margin squared. Endopterygoid superior and inferior portions approximately equal in size, ascending process robust, straight, tip simple. Interopercle dorsal margin ascending process present. Dorsal region of hyomandibula with four lateral foramenae, supraorbital and infraorbital nerves connected. Preopercle anteroventral notch present, posterodorsal laterosensory ramus with 2 superficial pores, margin of medial shelf smooth, median shelf broad, greater than half width of symplectic. Opercle dorsal margin straight, posterior margin smooth. Subopercle dorsal margin concave. Cranial fontanels closed in juveniles and adults. Frontal anterior margin straight, postorbital process narrow, less than two times width of supraorbital canal. Lateral ethmoid unossified. Parietal rectangular, length equal to width. Pterophenoid anteroventral process reduced, extends to lateral process. Parasphenoid posterior processes short, stout. Prootic foramen Vp combined with V2-3+VII. Adductor mandibula insertion undivided, intermusculars absent. All basibranchials unossified. Gill rakers not contacting gill bar.

Cleithrum narrow with straight ventral margin, anterior limb long, greater than 1.8 times ascending limb, without facet for insertion of muscle from supracleithrum. Postcleithrum thin, discoid or sickle shaped. Body cavity intermediate, with 36–37 precaudal vertebrae. Rib 5 robust along its entire extent, less than three times width of rib 6. Displaced hemal spines absent. Pectoral fin small, with 12–15 rays. Anal fin long, with 180–205 rays. Lateral-line complete. Lateral-line dorsal rami absent in adults. Single hypaxial electric organ, extending along entire ventral margin of body with 3–4 rows of electroplates near caudal insertion of anal fin.

### *Gymnotus (Gymnotus) carapo* linnaeus

#### Materials examined in morphological analyses

*Gymnotus (Gymnotus) carapo australis*: MLP 11221 (Holotype), 341 mm, Argentina, Misiones, Rio Iguazú drainage, Arroyo Verde (25°40’15”S, 053°56’01”W); MLP 11222 (Paratypes) (6), 262–321 mm, same locality as MLP 11221; AI 253, 275 mm, Argentina, Misiones, Verde Creek (25°40’15”S, 053°56’00”W); AI 254, 186 mm, Argentina, Misiones, Arroyo Deseado (25°47’08”S, 054°02’21”W); CFA-IC-2859, Argentina, Pilcomayo, Formosa, Laguna Blanca (25°10’16”S, 058°07’53”W); CFA-IC-2860, same locality as CFA-IC-2859; CFA-IC-2861, Argentina, Pilcomayo, Formosa, Canal San Juan (25°10’32”S, 057°58’11”W); CFA-IC-2862 (4), Argentina, Pilcomayo, Formosa, Río Pilcomayo sur (24°58’46”S, 058°18’11”W); MACN-ict 4638, 340 mm, Argentina, Corrientes, Río Paraná drainage, Esteros de Santa Lucía, Manantiales; MACN-ict 9654 (5), 220–348 mm, Argentina, Corrientes, Río Paraná drainage, Esteros del Riachuelo (27°34’39”S, 058°15’23”W); MLP 5769, Argentina, Buenos Aires, Chascomús, Laguna Chascomús (35°35’01”S, 058°02’50”W); MLP 11223 (7), 110–330 mm, Argentina, Misiones, Arroyo Deseado Chico, cuenca Rio Iguazú (25°47’20”S, 054°01’45”W); MLP 11225 (3), 221–318 mm, Argentina, Misiones, Arroyo Lobo, cuenca Rio Iguazú (25°42’32”S, 054°05’36”W); MLP 11226 (3), 268–274 mm, Argentina, Misiones, Arroyo Tateto, cuenca Rio Iguazú (25°47’13”S, 053°58’13”W); UFRGS 6763 (c&s), Brazil, Rio Grande do Sul, Rosário do Sul/Alegrete, Rio Uruguai, Arroio Gueromana, alongside BR290 (30°00’60”S, 055°23’18”W); MUSM 52428 (2), 260–287 mm, Brazil, Paraná, Londrina, Rio Tibagi drainage, Ribeirão Três Bocas, in Dr. Daisaku Ikeda Ecological Park (23°23’06”S, 051°04’33”W); UFRGS 11907 (4), 275–419 mm, Brazil, Mato Grosso do Sul, small lakes and streams adjacent to the Rio Paraguai, between the cities of Corumbá and Aquiduana (23°23’09”S, 045°39’49”W). *Gymnotus (Gymnotus) carapo caatingaensis*: AUM 20624 (Holotype), 225 mm. Brazil, Piauí, Río Gurgueia aff. Río Parnaíba (08°32’24”S, 044°25’48”W); AUM 20624 (Paratype), 210 mm. same locality as AUM 20624; AUM 20689 (2), 95–138 mm, Brazil, Piauí, Río Gurgueia, 25 km SW Urucui; AUM 2079 (4), 156–192 mm, Brazil, Piauí, Parnaíba, between Santa Filomena and Jurumenha; AUM 20793 (4), 111–195 mm, Brazil, Piauí, Parnaíba, between Santa Filomenha and Jurumenha. *Gymnotus (Gymnotus) carapo G*. *carapo*: NRM 64 (Syntype), 262 mm, Suriname, near Paramaribo, collected in the first half of the 18th Century; NRM 8224 (Syntype), 331 mm, same locality as NRM 64; UUZM Linn. coll. 56 (Syntype), 293 mm, same locality as NRM 64; MCZ 31219, 262 mm, Suriname; UMMZ 190414, 260 mm, Suriname, Brokopondo, Suriname River, Tapeoeripa creek near Brokopondo village (05°04’N, 054°58’W); UMMZ 190414 (5), 71–214 mm, same locality as UMMZ 190414; USNM 225274 (8), 81–318 mm, Suriname, Nickerie, Lucie River, upstream of Amotopo-Camp Geologie Rd. (03º36’N, 57º37’W); USNM 225275 (11), 81–270 mm, Suriname, Nickerie, Corantijn River, 350 m downstream from Wilhelm II falls (03º34’N, 057º15’W); USNM 225276 (16), 75–148 mm, same locality as USNM 225274; USNM 225284 (10), 54–143 mm, same locality as USNM 225275; USNM 225285 (12), 85–257 mm, Suriname, Nickerie, Corantijn River, creek south of Matapi, approx. 2 km downstream of Cow Falls (04º59’N, 057º38’W); USNM 225286 (15), 80–319 mm, Suriname, Nickerie, Corantijn River, Koekwie creek (05º31’N, 057º10’W); USNM 225290 (19), 14–157 mm, Suriname, Nickerie, Corantijn River, Dalibane Creek, Camp Dacclemmen (05º34’N, 057º11’W); USNM 225297 (14), 53–137 mm, Suriname, Nickerie, Corantijn River, stream on S. side Lucie River (03º35’N, 057º39’W); UF 180165 (6), 185–337 mm, Suriname, Para, Kola Kreek aff. Para River (05°27’8.820”N, 055°14’42.060”W); UF 180169 (5), 182–340 mm, Suriname, Brokopondo, Marshall Kreek, aff. Suriname River (05°14’24.9”N, 055°05’58.6”W); UF180173 (11), 34–72 mm; UF 180175 (2), 65–219 mm, Suriname, Marowijne, Cottica River, aff. Commewijne River (05°35.21’N, 054°17.11’W). *Gymnotus (Gymnotus) carapo madeirensis*: MUSM 60285 (Holotype), 227 mm, Peru, Puno, Sandia, Río Candamo (13°31’S, 069°41’W); MUSM 60286 (Paratypes) (2), 101–121 mm, same locality as MUSM 60285; UF 82191, 187 mm, Bolivia, Santa Cruz, Río Guaporé, Río San Pablo, Río San Diablo, Velasco, 71 km N. San José de Chiquitos (17°18’S, 060°35’W); UF 82211 (2), 196–205 mm, same locality as UF 82191; UF 82345 (3) 111–360 mm, Bolivia, Santa Cruz, Río Jorge, W. of Warnes, Río Piray (17º34’12”S, 063º12’36”W); UF 82470 (2), 170–218 mm, Bolivia, Beni, Río Mamoré, Río Ibaré, Cercado, Arroyo San Javier (14°38’S, 064°53’W); UF 82485, 360 mm, Bolivia, Beni, Río Mamoré, Río Ibaré, Cercado, 20 km N San Javier (14°28’S, 64°56’W); UF 82510, 241 mm, Bolivia, Beni, Río Mamoré, Río Ibaré, Cercado, 25 km N San Javier (14°24’S, 064°56’W); UMMZ 66433 (2), 215–331 mm, Bolivia, Beni, Río Beni, Lago Rogoagua (13°57’S, 066°58’W); UMSS 06962, 108 mm, Bolivia, Beni, Río Madeira, Arroyo Tres Cuchillas (10°49’23.016”S, 066°4’50.300” W); UMSS 06963, 121 mm, same locality as UMSS 06962; UMSS 06964, 174 mm, same locality as UMSS 06962; UMSS 06964, 177 mm, Bolivia, Beni, Río Madeira, Arroyo Tres Cuchillas (10°49’23.016”S, 066°4’50.300”W); UMSS 06965, 145 mm, same locality as UMSS 06962; UMSS 06966, 179 mm, same locality as UMSS 06962; UMSS 06969, 302 mm, same locality as UMSS 06962; UMSS 06970, 119 mm, same locality as UMSS 06962; UMSS 06977, 202 mm, Bolivia, Río Beni aff. Río Madeira (11°01’41.200”S, 066°5’58.801”W). *Gymnotus (Gymnotus) carapo occidentalis*: UF 126181 (Holotype), 272 mm, Peru, Loreto, Río Pacaya, Cocha Zapote (05°20’02”S, 074°29’05”W); UF 126181 (Paratype), 245 mm, same locality as UF 126181; ANSP 203173 (20) 105–263 mm, Peru, Loreto, Cocha Santo Tomás aff. Río Nanay near Iquitos; INHS 55038, 323 mm, Peru, Loreto, Río Napo aff. Amazon; INPA 6390, 450 mm, Brazil, Mato Grosso, Aripuanã, Río Aripuanã, Igarapé Castanhal; MUSM 11590 (5), 193–211 mm, Cuenca Ebehuabaeji, Sandia; MUSM 58770 (20) 86–275 mm, Peru, Loreto, Cocha Santo Tomás aff. Río Nanay near Iquitos (03°47’59.92”S, 073°20’14.48”W); MZUSP 76061, 260 mm, Brazil, Amazonas, Río Tefé, Lago Tefé, Cabeçeira do Lago Tefé (03°34’35”S, 064°59’19”W); MZUSP 76063, 298 mm, Brazil, Amazonas, MSDR, Ressaca da Vila Alencar (03°07’42”S, 064°48’02”W); MZUSP 76064, 253 mm, Brazil, Amazonas, MSDR, Cano do Lago Mamirauá (03º04’26”S, 064º48’39”W); NRM 27650, 305 mm, Peru, Loreto, Río Samiria, Maynas, right bank stream tributary between Caño Pastos and Hamburgo (05°2’S, 075°08’W); NRM 27650, 305 mm, Peru, Loreto, Río Samiria, Maynas, right bank stream tributary between Caño Pastos and Hamburgo (05°12’S, 075°08’W); NRM 40772, 91 mm, Peru, Loreto, Río Maniti, Maynas, 50 km NE of Iquitos (03°29’S, 072°44’W); UF 116573 (2), 189–279 mm, Peru, Loreto, Río Amazonas, Maynas, Río Nanay; UF 116665, 298 mm, Peru, Loreto, Río Nanay, Maynas, 3 km upstream Mishana, Reserva Allpahuayo-Mishana (03°52’05”S, 073°29’03”W); UF 116665, 298 mm, same locality as UF 116665; UF 122820, 275 mm, Peru, Loreto, Río Amazonas, Maynas, Río Nanay; UF 122821, 331 mm, Peru, Loreto, acquired from fishermen (03°46’S, 073°15’W); UF 122822, 330 mm, same locality as UF 122820; UF 122822, 334 mm, Peru, Loreto, acquired from fishermen (03°46’S, 073°15’W); UF 122825, 158 mm, same locality as UF 122820; UF 122825, 158 mm, same locality as UF 122820; UF 122847, 188 mm, same locality as UF 122820y; UF 122847, 188 mm, same locality as UF 122820; UF 122848, 112 mm, same locality as UF 122820; UF 122849, 132 mm, same locality as UF 122820; UF 122849, 132 mm, same locality as UF 122820; UF 122852, 92 mm, same locality as UF 122820; UF 128991, 291–307 mm, Peru, Loreto, Ucayali, (05°18’7.3”N, 074°03’36.03”W); UF 131129, 172 mm, Peru, Loreto, Río Amazonas, Maynas, Río Nanay (03°45”S, 073°15’W); UF 131129, 172 mm, Peru, Loreto, Río Amazonas, Maynas, Río Nanay, near Iquitos; UFRGS 22695 (23), 104–266 mm, Peru, Loreto, Cocha Santo Tomás aff. Río Nanay near Iquitos; UMMZ 228999, 162 mm, Peru, Loreto, Río Tahuayo (04°10’S, 073°12’W); UMMZ 230733, 251 mm, Peru, Loreto, Río Yavari, Buen Suceso, Quebrada Carana (approx. 04º08’S, 070º26’W); UMMZ 230734 (2), 190–210 mm, Brazil, Santa Cruz, Benjamin Constante, Río Nanay Río Nanay, Río Cayari (04°22’S, 070°02’W); ROM 83885, 233 mm, Guyana, Essequibo, Cuyuni-Mazaruni; ROM 83886, 238 mm, same locality as ROM 83885; ROM 83887, 210 mm, same locality as ROM 83885; ROM 89520, 189 mm, same locality as ROM 83885; ROM 89583, 278 mm, same locality as ROM 83885; ROM 89660, 176 mm, same locality as ROM 83885; ROM 89661, 158 mm, same locality as ROM 83885; ROM 89662, 132 mm, same locality as ROM 83885; ROM 93388, 246 mm, same locality as ROM 83885; ROM 93389, 238 mm, same locality as ROM 83885; ROM 93407, 248 mm, same locality as ROM 83885; ROM 93424, 245 mm, same locality as ROM 83885; ROM 93425, 205 mm, same locality as ROM 83885; ROM 93426, 186 mm, same locality as ROM 83885. *Gymnotus (Gymnotus) carapo orientalis*: MZUSP 30025 (Holotype) 237 mm, Brazil Pará, Río Tocantins, Río Itacaiuna, Serra Norte, Serra dos Carajás (05°05’S, 050°22’W); MZUSP 30025 (Paratypes) (6), 165–235 mm, same locality as MZUSP 30025; MCZ 45189 (19), 133–210 mm, Brazil Pará, Parauapebas, Ilha de Marajó, Río Tocantins, Río Arari, Cachoeira do Arari (01°01’S, 048°58’W); MZUSP 30008 (8), 173–253 mm, Brazil Pará, Río Trombetas, Río Itacaiunas, Caldeirão, Igarapé do Pojuca (approx. 00°45’S, 056°13’W); MZUSP 30025 (7), 165–237 mm, Brazil, Pará, Río Tocantins, Río Itacaiuna, Serra Norte, Serra dos Carajás (05°05’S, 050°22’W). *Gymnotus (Gymnotus) carapo septentrionalis*: UF 80734 (Holotype), 220 mm, Venezuela, Apure, Río Apure, E. dike in UNELLEZ module (09°55’7.26”N, 068°17’56.82”W); UF 80734 (Paratypes) (46), 165–262 mm, same locality as UF 80734; NRM 27717, 235 mm, Colombia, Meta, Río Meta, Río Ocoa, Laguna Santa Clara, ca. 5 km S Villavicencio (~04°09’N, 073°39’W); UF 26178 (39), 134–239 mm, Colombia, Meta, Río Guatiquia-Meta, Caños Negros, ca. 9 km Villavicencio on road to Puerto Porfia (~04°09’N, 073°39’W); UF 33245, 200 mm, Colombia, Meta, Río Meta, Río Guamal, 7 km E. Río Guayuriba, just N. Guamal (03°52’N, 073°45’W); UF 35402 (3), 148–178 mm, Venezuela, Guarico, Río Apure, ca. 50 km N San Fernando de Apure (~07°54’N, 067°28’W); UF 37030 (20), 101–176 mm, Venezuela, Apure, Río Apure, Río Guaritico, Hato El Frío (09°03’N, 068°20’W); UF 77334 (1), 173 mm, Venezuela Apure, Río Apure, Cano Caicara, ca. 30 km SW La Ye (07°40’N, 072°22’W); UF 78069 (3), 151–190 mm, Venezuela, Apure, Río Apure, 2.3 km N San Fernando de Apure (~07°54’N, 067°28’W); UMMZ 169080 (5), 46–71 mm, Trinidad & Tobago, Mayaro, Gunupia, Mt. Plaisance Village (10°16’N, 061°00’W).

#### Diagnosis

*Gymnotus (Gymnotus) carapo* can be differentiated from all other members of the subgenus except *G*. *(G*.*) arapaima*, *G*. *(G*.*) sylvius* and *G*. *(G*.*) ucamara* on the basis of the following characters: long head (HL 10.2–14.0% TL vs. 10.3–12.6% in *G*. *(G*.*) bahianus*, 8.8–11.7% in *G*. *(G*.*) chaviro*, 9.7–10.7% in *G*. *(G*.*) chimarrao*, 11.1–12.8% in *G*. *(G*.*) choco*, 9.9–12.2% in *G*. *(G*.*) cuia*, 8.8–10.5% in *G*. *(G*.*) curupira*, 10.8–11.9% in *G*. *(G*.*) diamantinensis*, 8.9–12.3% in *G*. *(G*.*) eyra*, 9.6–11.9% in *G*. *(G*.*) mamiraua*, 9.9–11.3% in *G*. *(G*.*) obscurus*, 10.6–12.4% in *G*. *(G*.*) omarorum*, 7.2–11.7% in *G*. *(G*.*) pantanal*, 7.3–10.3% in *G*. *(G*.*) riberalta*, 9.1–10.3% in *G*. *(G*.*) varzea*). *Gymnotus (Gymnotus) carapo* can be differentiated from *G*. *(G*.*) sylvius* and *G*. *(G*.*) ucamara* on the basis of the following characters: color pattern with dark band pairs 1–3X width of pale interbands (vs. dark band pairs equal in width to pale interbands in *G*. *(G*.*) sylvius*, dark band pairs >5X width of interbands in *G*. *(G*.*) ucamara*). *Gymnotus (Gymnotus) carapo* can be differentiated from *G*. *(G*.*) arapaima* on the basis of the following characters: color pattern with no countershading (vs. reverse-countershading in *G*. *(G*.*) arapaima*).

#### Description

Sexually monomorphic. Size up to 419 mm TL with adult body proportions attained at about 150 mm TL. Adult body shape subcylindrical with a mean ratio of body width to depth of 69.5%. Body profile slender, body depth 63.7–106.3% total length. Head length long, 10.2–15.0% total length. Snout length moderate, 29.6–40.1% head length. Mouth width narrow, 32.3–49.0% head length. Preanal distance long, 60.3–99.2%head length. Anal-fin long, 67.6–92.3% total length. Cycloid or ovoid scales present on entire post-cranial portion of body from nape to caudal appendage.

Scales above lateral line intermediate, in 5–8 rows. Scales over anal-fin pterygiophores large, with 6–12 rows. Gape large, extending to or beyond posterior nares. Mouth position superior, lower jaw longer than upper, rictus decurved. Chin round in lateral, dorsal profiles, fleshy and bulbous with mental electroreceptive organ overlying lower jaw. Anterior narial pore partially or entirely included within gape, in small narial fold. Anterior nares small, its diameter less than that of eye. Eye below horizontal with mouth. Circumorbital series ovoid. Premaxilla with >11 teeth disposed in two rows along outer margin, median margin curved. Maxilla-palatine articulation near tip of endopterygoid. Maxilla vertical, rod- or paddle-shaped distally with a straight ventral margin, length equal to 4–6 dentary teeth. Dentary with one row of >16 teeth, 4–7 arrowhead shaped anteriorly, all others conical posteriorly. Posterodorsal and posteroventral dentary processes abuts ventral. Dentary posteroventral process shorter than or almost as long as posterodorsal, narrow distally. Dentary ventral margin lamella large, depth greater than posterior process. Dentary anteroventral margin without a hook. Anguloarticular process long, extending over ventral margin of dentary. Retroarticular with an arched lamella posteriorly forming a small canal, posterior margin squared. Endopterygoid superior and inferior portions approximately equal in size, ascending process robust, curved, tip simple. Interopercle dorsal margin ascending process present. Dorsal region of hyomandibula with four lateral foramenae, supraorbital and infraorbital nerves divided. Preopercle anteroventral notch present, posterodorsal laterosensory ramus with 2 superficial pores, margin of medial shelf smooth, median shelf large, greater than half width of symplectic. Opercle dorsal margin straight, posterior margin smooth. Subopercle dorsal margin concave. Cranial fontanels closed in juveniles and adults. Frontal anterior margin straight, postorbital process broad, greater than two times width of supraorbital canal. Lateral ethmoid unossified. Parietal rectangular, length equal to width. Pterophenoid anteroventral process robust, extends ventral to lateral process. Parasphenoid posterior processes gracile. Prootic foramen Vp separate from V2-3+VII. Adductor mandibula insertion undivided, intermusculars absent. All basibranchials unossified. Gill rakers not contacting gill bar.

Cleithrum board with curved ventral margin, anterior limb long, greater than 1.8 times ascending limb, without facet for insertion of muscle from supracleithrum. Postcleithrum thin, discoid or sickle shaped. Body cavity long, with 32–43 precaudal vertebrae. Rib 5 robust along its entire extent, less than three times width of rib 6. Displaced hemal spines absent. Pectoral fin variable, with 13–17 rays. Anal fin long, with 173–290 rays. Lateral-line complete, with 18–21 ventral rami. Lateral-line dorsal rami absent in adults. Single hypaxial electric organ, extending along entire ventral margin of body with 3–4 rows of electroplates near caudal insertion of anal fin.

### *Gymnotus (Gymnotus) chaviro* maxime & albert

**Materials examined in morphological analyses**

MUSM 33715 (Holotype), 233 mm, Peru, Ucayali, Alto Yuruá, Quebrada Dos y medio, small terra firme stream ~2 km NW of Breu (09°31’10.500”S, 072°45’45.300”W); MUSM 33714 (Paratypes) (40), 95–275 mm, same locality as MUSM 33715; FMNH 118274 (Paratypes) (10), 134–179 mm, same locality as MUSM 33715; CAS 227893 (Paratypes) (10), 123–150 mm, same locality as MUSM 33715; MCZ 168419 (Paratypes) (10), 115–160 mm, same locality as MUSM 33715; MCP 43880 (Paratypes) (10), 116–164 mm, same locality as MUSM 33715; MZUSP 103035 (Paratypes) (10), 130–217 mm, same locality as MUSM 33715; AMNH 248884 (Paratypes) (10), 104–180 mm, same locality as MUSM 33715; MUSM 1406, 127 mm, Peru, Madre de Dios, Parque Nacional Manú, Quebrada Pakitza, Aguajal; MUSM 1759 (2), 142–150 mm, Peru, Madre de Dios, Puerto Maldonado, river near Tambopata, Cochachica; MUSM 21405, 138 mm, Peru, Madre de Dios; MUSM 22731 (10), 143–210 mm, Peru, Madre de Dios, Madre de Dios drainage; MUSM 16662, 325 mm, Peru, Madre de Dios, Tambopata, Madre de Dios drainage, Lago Copamanu.

### Diagnosis

*Gymnotus (Gymnotus) chaviro* can be differentiated from all other members of the subgenus on the basis of the following characters: color pattern with 2–3 of the posteriormost 5 pale interbands with both margins crescent-shaped, bending outward (vs. bands absent or margins of posteriormost interbands straight or margins of posteriormost interbands curved in parallel in all other *Gymnotus*).

### Description

Sexually monomorphic. Size up to 275 mm TL with adult body proportions attained at about 150 mm TL. Adult body shape subcylindrical with a mean ratio of body width to depth of 77.6%. Body profile slender, body depth 69.6–109.7% total length. Head length short, 8.8–13.6% total length. Snout length moderate, 32.3–39.8% head length. Mouth width narrow, 35.5–52.8% head length. Preanal distance long, 61.3–99.4%head length. Anal-fin long, 80.2–86.2% total length. Cycloid or ovoid scales present on entire post-cranial portion of body from nape to caudal appendage.

Scales above lateral line intermediate, in 5–9 rows. Scales over anal-fin pterygiophores large, with 12–13 rows. Gape large, extending to or beyond posterior nares. Mouth position superior, lower jaw longer than upper, rictus decurved. Chin round in lateral, dorsal profiles, fleshy and bulbous with mental electroreceptive organ overlying lower jaw. Anterior narial pore partially or entirely included within gape, in small narial fold. Anterior nares small, its diameter less than that of eye. Eye below horizontal with mouth. Circumorbital series ovoid. Premaxilla with <10 teeth disposed in two rows along outer margin, median margin curved. Maxilla-palatine articulation near tip of endopterygoid. Maxilla vertical, rod- or paddle-shaped distally with a straight ventral margin, length equal to 4–6 dentary teeth. Dentary with one row of <12 teeth, 4–7 arrowhead shaped anteriorly, all others conical posteriorly. Posterodorsal and posteroventral dentary processes abuts ventral. Dentary posteroventral process shorter than or almost as long as posterodorsal, narrow distally. Dentary ventral margin lamella large, depth longer than posterior process. Dentary anteroventral margin without a hook. Anguloarticular process long, extending beyond ventral margin of dentary. Retroarticular with an arched lamella posteriorly forming a small canal, posterior margin squared. Endopterygoid superior and inferior portions approximately equal in size, ascending process robust, straight, tip simple. Interopercle dorsal margin ascending process present. Dorsal region of hyomandibula with four lateral foramenae, supraorbital and infraorbital nerves divided. Preopercle anteroventral notch present, posterodorsal laterosensory ramus with 2 superficial pores, margin of medial shelf smooth, median shelf large, greater than half width of symplectic. Opercle dorsal margin straight, posterior margin smooth. Cranial fontanels closed in juveniles and adults. Frontal anterior margin straight, postorbital process narrow, less than two times width of supraorbital canal. Lateral ethmoid unossified. Parietal rectangular, length equal to width. Pterophenoid anteroventral process robust, extends to lateral process. Parasphenoid posterior processes robust. Prootic foramen Vp separate from V2-3+VII. Adductor mandibula insertion undivided, intermusculars absent. All basibranchials unossified. Gill rakers not contacting gill bar.

Cleithrum board with curved ventral margin, anterior limb long, greater than 1.8 times ascending limb, with facet for insertion of muscle from supracleithrum. Postcleithrum thin, discoid or sickle shaped. Body cavity intermediate, with 35–38 precaudal vertebrae. Rib 5 robust along its entire extent, less than three times width of rib 6. Displaced hemal spines absent. Pectoral fin board, with 16–19 rays. Anal fin long, with 212–280 rays. Lateral-line complete, with 18–23 ventral rami. Lateral-line dorsal rami absent in adults. Single hypaxial electric organ, extending along entire ventral margin of body with 3–4 rows of electroplates near caudal insertion of anal fin.

## *Gymnotus* (*Gymnotus*) *chimarrao* cognato, richer-de-forges albert & crampton

### Materials examined in morphological analyses

UFRGS 6774 (Holotype), 185 mm, Brazil, Rio Grande do Sul, Rio Taquari Drainage, Arroio do Meio, Arroio Grande (29°21’09”S, 051°57’28”W); UFRGS 6770 (Paratype), 190 mm, same locality as UFRGS 6774; UFRGS 6771 (Paratype), 243 mm, same locality as UFRGS 6774; UFRGS 6772 (Paratype), 177 mm, same locality as UFRGS 6774; UFRGS 6773 (Paratype), 206 mm, same locality as UFRGS 6774; UFRGS 6776 (Paratype), 124 mm, same locality as UFRGS 6774; UFRGS 11952, 223 mm, Rio Grande do Sul, Nova Roma do Sul (28°57’18”S, 051°22’32”W); UFRGS 17607 (2), 111–222 mm, Rio Grande do Sul, Canudos do Vale (29°24’22”S, 052°03’19”W).

### Diagnosis

*Gymnotus (Gymnotus) chimarrao* can be differentiated from all other members of the subgenus on the basis of the following characters: color pattern with singly-occurring dark pigment bands in juveniles (29–45) and faint or absent bands in mature specimens (vs. paired bands, rounded spots or irregularly-shaped blotches in all other *Gymnotus*).

### Description

Sexually monomorphic. Size up to 243 mm TL with adult body proportions attained at about 150 mm TL. Adult body shape subcylindrical with a mean ratio of body width to depth of 39.2%. Body profile slender, body depth 68.3–99.7% total length. Head length short, 9.7–11.3% total length. Snout length moderate, 31.7–41.6% head length. Mouth width narrow, 33.8–46.9% head length. Preanal distance long, 68.5–87.1%head length. Anal-fin long, 76.6–83.8% total length. Cycloid or ovoid scales present on entire post-cranial portion of body from nape to caudal appendage.

Scales above lateral line intermediate, in 6–8 rows. Scales over anal-fin pterygiophores large, with 9–13 rows. Gape large, extending to or beyond posterior nares. Mouth position superior, lower jaw longer than upper, rictus decurved. Chin round in lateral, dorsal profiles, fleshy and bulbous with mental electroreceptive organ overlying lower jaw. Anterior narial pore partially or entirely included within gape, in small narial fold. Anterior nares small, its diameter less than that of eye. Eye below horizontal with mouth. Circumorbital series ovoid. Premaxilla with >11 teeth disposed in two rows along outer margin, median margin curved. Maxilla-palatine articulation near tip of endopterygoid. Maxilla rod- or paddle-shaped, narrow distally with a straight ventral margin, length equal to 4–6 dentary teeth. Dentary with one row of 12–15 teeth, 2–4 arrowhead shaped anteriorly, all others conical posteriorly. Posterodorsal and posteroventral dentary processes abuts ventral. Dentary posteroventral process shorter than or almost as long as posterodorsal, narrow distally. Dentary ventral margin lamella large, depth greater than posterior process. Dentary anteroventral margin absent a hook. Anguloarticular process long, extending beyond ventral margin of dentary. Retroarticular with an arched lamella posteriorly forming a small canal, posterior margin squared. Endopterygoid superior and inferior portions approximately equal in size, ascending process robust, curved, tip simple. Interopercle dorsal margin ascending process absent. Dorsal region of hyomandibula with four lateral foramenae, supraorbital and infraorbital nerves divided. Preopercle anteroventral notch present, posterodorsal laterosensory ramus with 2 superficial pores, margin of medial shelf smooth, median shelf large, greater than half width of symplectic. Opercle dorsal margin straight, posterior margin smooth. Subopercle dorsal margin concave. Cranial fontanels closed in juveniles and adults. Frontal anterior margin straight, postorbital process narrow, less than two times width of supraorbital canal. Lateral ethmoid unossified. Parietal rectangular, length equal to width. Pterophenoid anteroventral process robust, extends to ventral lateral process. Parasphenoid posterior processes robust. Prootic foramen Vp separate V2-3+VII. Adductor mandibula insertion undivided, intermusculars absent. All basibranchials unossified. Gill rakers not contacting gill bar.

Cleithrum broad with curved ventral margin, anterior limb long, greater than 1.8 times ascending limb, with facet for insertion of muscle from supracleithrum. Postcleithrum thin, discoid or sickle shaped. Rib 5 robust along its entire extent, less than three times width of rib 6. Displaced hemal spines absent. Pectoral fin broad, with 14–17 rays. Anal fin long, with 191–250 rays. Lateral-line complete, with 13–24 ventral rami. Lateral-line dorsal rami absent in adults. Single hypaxial electric organ, extending along entire ventral margin of body with 3 rows of electroplates near caudal insertion of anal fin.

## *Gymnotus* (*Gymnotus*) *choco* Albert, crampton & maldonado

### Materials examined in morphological analyses

ICNMHN 6621 (Holotype), 237 mm, Colombia, Chocó, Boca de Pepé, Río Baudó (05°03’N, 77°03’W); NRM 27734 (Paratypes) (6), 142–260 mm; same locality as ICNMHN 6621; CAS 72192 (2), 150–179 mm, Colombia, Chocó, Atrato basin, Río Sucio, Río Truando (07°09’N, 77°12’W); ICNMHN 6686, 321 mm, Colombia, Risaralda, Pueblo Rico, upper Río San Juan; IMCN 1050 (2), 208–239 mm, Colombia, Chocó, Resguardo Puerto Pizarro, Río San Juan, Litoral del San Juan; IMCN 1370 (1), 215 mm, same locality as IMCN 1050; FMNH 70511 (5), 124–247 mm, Colombia, Chocó, Río Baudó at Pizarro (Baja Baudó) (04°58’N, 77°22’W); FMNH 56794 (2), 174–175 mm, same locality as CAS 72192; NRM 27744, 350 mm, Colombia, Chocó, Atrato basin, Quebrada Piscindé, close to Pan-American bridge across Río San Pablo (05°42’N, 76°37’W).

### Diagnosis

*Gymnotus (Gymnotus) choco* can be differentiated from all other members of the subgenus on the basis of the following characters: color pattern with intermediate (2–5X width of interbands) dark bands, irregular, wavy band margins, pale interbands restricted to the ventral part of the lateral surface anteriorly and 1–3 dark bands divided ventrally to form inverted-“Y” shapes (vs. spotted, with dark bands <2X or >5X width of pale interbands in all other *Gymnotus*).

### Description

Sexually monomorphic. Size up to 260 mm TL with adult body proportions attained at about 150 mm TL. Adult body shape subcylindrical with a mean ratio of body width to depth of 76.9%. Body profile slender, body depth 68.9–93.3% total length. Head length short, 11.1–13.3% total length. Snout length moderate, 31.9–35.9% head length. Mouth width narrow, 31.3–37.2% head length. Preanal distance long, 76.7–90.7%head length. Anal-fin long, 75.8–83.8% total length. Cycloid or ovoid scales present on entire post-cranial portion of body from nape to caudal appendage.

Scales above lateral line intermediate, in 6 rows. Scales over anal-fin pterygiophores large, with 8–9 rows. Gape large, extending to or beyond posterior nares. Mouth position superior, lower jaw longer than upper, rictus decurved. Chin round in lateral, dorsal profiles, fleshy and bulbous with mental electroreceptive organ overlying lower jaw. Anterior narial pore partially or entirely included within gape, in small narial fold. Anterior nares small, its diameter less than that of eye. Eye below horizontal with mouth. Circumorbital series ovoid. Premaxilla with >11 teeth disposed in two rows along outer margin, median margin curved. Maxilla-palatine articulation near tip of endopterygoid. Maxilla rod- or paddle-shaped, narrow distally with a straight ventral margin, length equal to 4–6 dentary teeth. Dentary with one row of >16 teeth, 4–7 arrowhead shaped anteriorly, all others conical posteriorly. Posterodorsal and posteroventral dentary processes abuts ventral. Dentary posteroventral process shorter than or almost as long as posterodorsal, narrow distally. Dentary ventral margin lamella small, depth less than posterior process. Dentary anteroventral margin without a hook. Anguloarticular process short, extending to ventral margin of dentary. Retroarticular with an arched lamella posteriorly forming a small canal, posterior margin squared. Endopterygoid superior and inferior portions approximately equal in size, ascending process robust, curved, tip simple. Interopercle dorsal margin ascending process present. Dorsal region of hyomandibula with four lateral foramenae, supraorbital and infraorbital nerves divided. Preopercle anteroventral notch present, posterodorsal laterosensory ramus with 2 superficial pores, margin of medial shelf smooth, median shelf large, greater than half width of symplectic. Opercle dorsal margin straight, posterior margin smooth. Cranial fontanels closed in juveniles and adults. Frontal anterior margin straight, postorbital process broad, greater than two times width of supraorbital canal. Lateral ethmoid unossified. Parietal rectangular, length equal to width. Pterophenoid anteroventral process robust, extends to lateral process. Parasphenoid posterior processes gracile. Prootic foramen Vp separate from V2-3+VII. Adductor mandibula insertion undivided, intermusculars absent. All basibranchials unossified. Gill rakers not contacting gill bar.

Cleithrum broad with curved ventral margin, anterior limb long, greater than 1.8 times ascending limb, with facet for insertion of muscle from supracleithrum. Postcleithrum thin, discoid or sickle shaped. Body cavity intermediate, with 32–35 precaudal vertebrae. Rib 5 robust along its entire extent, less than three times width of rib 6. Displaced hemal spines absent. Pectoral fin broad, with 14–16 rays. Anal fin long, with 210–255 rays. Lateral-line complete. Lateral-line dorsal rami absent in adults. Single hypaxial electric organ, extending along entire ventral margin of body with 3 rows of electroplates near caudal insertion of anal fin.

## *Gymnotus* (*Gymnotus*) *cuia* craig, malabarba, crampton & albert

### Materials examined in morphological analyses

UFRGS 23700 (Holotype), 193 mm, Brazil, Rio Grande do Sul, Viamão, Lagoa Verde, Itapuã State Park, Rio Grande, do Sul (30°22’52”S, 051°01’25”W); UFRGS 6854 (Paratypes) (5), 104–169 mm, same locality as UFRGS 23700; UFRGS 6855 (Paratypes) (3), 164–279 mm, same locality as UFRGS 23700; UFRGS 6857 (Paratypes) (4), 152–236 mm, same locality as UFRGS 23700; UFRGS 6858 (Paratypes) (2), 168–223 mm, same locality as UFRGS 23700; UFRGS 6859 (Paratype), 266 mm, same locality as UFRGS 23700; UFRGS 8655 (Paratype), 130 mm, same locality as UFRGS 23700; UFRGS 9103 (Paratypes) (2), 216–237 mm, same locality as UFRGS 23700; UFRGS 9104 (Paratype), 153 mm, same locality as UFRGS 23700; UFRGS 9105 (Paratypes) (2), 125–131 mm, same locality as UFRGS 23700; UFRGS 9106 (Paratypes) (2), 152–187 mm, same locality as UFRGS 23700; UFRGS 9115 (Paratype), 166 mm, same locality as UFRGS 23700; UFRGS 9790 (Paratypes) (4), 188–261 mm, same locality as UFRGS 23700; UFRGS 9794 (Paratypes) (4), 171–217 mm, same locality as UFRGS 23700; MLP 110805 (3), 183–221 mm, Argentina, Corrientes, Río Paraná (~27°27’28.41”S, 058°47’54.13”W); UF 125973 (4), 197–238 mm, Argentina, Formosa, Río Bermejo drainage, ponds near Río Bermejo (~26°13’39”S, 058°09’60”W); MZUSP 59316, 198 mm, Brazil, Mato Grosso do Sul, Corumbá, Rio Vermelho drainage; MCP 19550 (2), 185–270 mm, Brazil, Rio Grande do Sul, São Gabriel, bridge over Banhado do Inhatium (30°15’43”S, 054°31’33”W); MCP 19999 (2), 265–305 mm, Brazil, Rio Grande do Sul, Sapiranga, Arroio Feitoria (29°34’00”S, 051°00’00”W); MCP 41952 (4), 140–205 mm, Brazil, Rio Grande do Sul, Rio Cacequi drainage, stream alongside RS640 to Cacequi (29°55’23”S, 054°49’52”W); MCP 42587, 208 mm, Brazil, Rio Grande do Sul, Viamão, Lagoa Negra, Itapuã State Park (30°21’35”S, 050°58’34”W); UFRGS 10066, Brazil, Rio Grande do Sul, Porto Alegre (30°07’32.57”S, 051°11’22.21”W); UFRGS 16370, 206 mm, Brazil, Rio Grande do Sul, Viamão, Area de Preservação Ambiental Banhado dos Pachecos (30°07’44.63”S, 050°50’17.67”W); UFRGS 5618 (2), 261–264 mm, Brazil, Rio Grande do Sul, Viamão, Lagoa Negra, Itapuã State Park (30°21’35”S, 050°58’34”W); UFRGS 5738, 162 mm, Brazil, Rio Grande do Sul, Santa Rosa, Lageado do Pessegueiro; UFRGS 6536, 208 mm, same locality as UFRGS 5618; UFRGS 6537, 233 mm, same locality as UFRGS 5618; UFRGS 6538, 195 mm, same locality as UFRGS 5618; UFRGS 6539, 195 mm, same locality as UFRGS 5618; UFRGS 6540, 130 mm, same locality as UFRGS 5618; UFRGS 6541, 140 mm, Brazil, Rio Grande do Sul, Sanga do Jacaré, 82 km from Alegrete (30° 12’42”S, 055°03’17”W); UFRGS 6544 (c&s), same locality as UFRGS 6541; UFRGS 6542, 152 mm, same locality as UFRGS 6541; UFRGS 6543, 198 mm, same locality as UFRGS 6541; UFRGS 6548, 241 mm, Brazil, Rio Grande do Sul, Terra de Areia, Rio Três Forquilhas (29°33’22”S, 050°04’19”W); UFRGS 6549, 203 mm, same locality as UFRGS 6548; UFRGS 6550, 116 mm, Brazil, Rio Grande do Sul, Eldorado do Sul, Arroio Passo dos Carros (30°05’54”S, 051°23’18”W); UFRGS 6551, 115 mm, same locality as UFRGS 6550; UFRGS 6553, 172 mm, Brazil, Rio Grande do Sul, Eldorado do Sul, Arroio Passo dos Carros (30°02’55”S, 051°23’34”W); UFRGS 6554, 135 mm, same locality as UFRGS 6553; UFRGS 6555, 128 mm, same locality as UFRGS 6550; UFRGS 6556, 166 mm, Brazil, Rio Grande do Sul, São Gabriel, Rio Uruguai drainage, Arroio Piraí (30°18’56”S, 054°24’22”W); UFRGS 6557, 143 mm, same locality as UFRGS 6556; UFRGS 6558, 191 mm, same locality as UFRGS 6556; UFRGS 6559, (c&s), same locality as UFRGS 6556; UFRGS 6560, 145 mm, same locality as UFRGS 6556; UFRGS 6561, 197 mm, same locality as UFRGS 6556; UFRGS 6573, 217 mm, Brazil, Rio Grande do Sul, Terra de Areia, streams in the Reserva Biológica da Mata Paludosa (29°30’41”S, 050°06’27”W); UFRGS 6581, Brazil, Rio Grande do Sul, Rio Grande do Sul, Agudo, Arroio Corupá along the road from Agudo to UHE Dona Francisca (29°33’54”S, 053°17’09”W); UFRGS 6587 (2), 159–170 mm, same locality as UFRGS 6581; UFRGS 6589, 268 mm, same locality as UFRGS 6587; UFRGS 8265 (2), 212–245 mm, Brazil, Rio Grande do Sul, Charqueadas (29°57’31”S, 051°33’10”W); MZUSP 79348, 222 mm, Brazil, São Paulo, Reservatório de Barra Bonita, Rio Tietê (22° 31’56”S, 048°31’05”W); MZUSP 83409, 195 mm, Brazil, São Paulo, Bariri, Rio Tietê a near UHE Bariri road; MZUSP 83421 (2), 20–181 mm, Brazil, São Paulo, Rio Tietê drainage, Bariri, Queixada stream (22°08’00”S, 048°44’33”W); MZUSP 83427 (5), 90.6–146 mm, Brazil, São Paulo, Rio Tietê drainage, Bariri, Catingueiro stream (22°07’00”S, 048°45’05”W); MNHNP 189, Paraguay, PTE Hayes, General Bruguez, lake on the premises of the General José M. Brugues military base (24°44’33”S, 058°50’10”W); MNHNP 1064, Paraguay, Alto Paraná, Río Aray, dry stream below the dam; MNHNP 1070 (3), Paraguay, PTE Hayes, "La Golondrina" Hotel, small puddle (~24°31’00”S, 058°40”10”W); MNHNP 1620, Paraguay, Cordillera, Piribebuy, Saltos de Pirareta, 500 m below the falls (25°30’24”S, 056°55’32”W); MNHNP 1734 (2); MNHNP 3389 (2), Paraguay, Alto Paraguay, Tajamar, Madrejon, 50 m from the administration building (20°37’34”S, 059°52’47”W); UFRGS 7990 (2), 144–206 mm, Uruguay, Artigas, Río Uruguay, Arroyo Guaviyú (30°37’51”S, 057°41’18”W).

### Diagnosis

*Gymnotus (Gymnotus) cuia* can be differentiated from all other members of the subgenus on the basis of the following combination of characters: short head (HL 9.9–12.2% TL vs. 12.1–14.2% in *G*. *(G*.*) arapaima*, 10.3–12.6% in *G*. *(G*.*) bahianus*, 10.2–14.0% in *G*. *(G*.*) carapo*, 8.8–11.7% in *G*. *(G*.*) chaviro*, 9.7–10.7% in *G*. *(G*.*) chimarrao*, 11.1–12.8% in *G*. *(G*.*) choco*, 8.8–10.5% in *G*. *(G*.*) curupira*, 10.8–11.9% in *G*. *(G*.*) diamantinensis*, 8.9–12.3% in *G*. *(G*.*) eyra*, 9.6–11.9% in *G*. *(G*.*) mamiraua*, 9.9–11.3% in *G*. *(G*.*) obscurus*, 10.6–12.4% in *G*. *(G*.*) omarorum*, 7.2–11.7% in *G*. *(G*.*) pantanal*, 7.3–10.3% in *G*. *(G*.*) riberalta*, 12.3–14.1% in *G*. *(G*.*) sylvius*, 11.7–12.7% in *G*. *(G*.*) ucamara*, 9.1–10.3% in *G*. *(G*.*) varzea*) combined with deep body (BD 94.5–130.5% HL vs. 63.4–93.1 in *G*. *(G*.*) arapaima*, 87.3–103.8 in *G*. *(G*.*) bahianus*, 67.0–106.3 in *G*. *(G*.*) carapo*, 75.3–103.8 in *G*. *(G*.*) chaviro*, 82.0–99.7 in *G*. *(G*.*) chimarrao*, 68.9–93.3 in *G*. *(G*.*) choco*, 73.0–89.3 in *G*. *(G*.*) curupira*, 80.3–84.0 in *G*. *(G*.*) diamantinensis*, 85.4–105.6 in *G*. *(G*.*) eyra*, 89.1–119.1 in *G*. *(G*.*) mamiraua*, 82.5–98.8 in *G*. *(G*.*) obscurus*, 88.5–102.3 in *G*. *(G*.*) omarorum*, 77.8–111.7 in *G*. *(G*.*) pantanal*, 86.9–100.1 in *G*. *(G*.*) riberalta*, 83.2–100.5 in *G*. *(G*.*) sylvius*, 78.0–90.0 in *G*. *(G*.*) ucamara*, 87.8–105.8 in *G*. *(G*.*) varzea*).

### Description

Sexually monomorphic. Size up to 305 mm TL with adult body proportions attained at about 150 mm TL. Adult body shape subcylindrical with a mean ratio of body width to depth of 46.8%. Body profile slender, body depth 94.5–130.5% total length. Head length short, 9.8–12.2% total length. Snout length moderate, 29.9–38.3% head length. Mouth width narrow, 37.5–52.7% head length. Preanal distance long, 51.2–95.7%head length. Anal-fin long, 76.3–92.3% total length. Cycloid or ovoid scales present on entire post-cranial portion of body from nape to caudal appendage.

Scales above lateral line intermediate, in 5–7 rows. Scales over anal-fin pterygiophores large, with 6–10 rows. Gape large, extending to or beyond posterior nares. Mouth position superior, lower jaw longer than upper, rictus decurved. Chin round in lateral, dorsal profiles, fleshy and bulbous with mental electroreceptive organ overlying lower jaw. Anterior narial pore partially or entirely included within gape, in small narial fold. Anterior nares small, its diameter less than that of eye. Eye below horizontal with mouth. Circumorbital series ovoid. Premaxilla with >11 teeth disposed in two rows along outer margin, median margin curved. Maxilla-palatine articulation near tip of endopterygoid. Maxilla rod-shaped, narrow distally with a curved ventral margin, length equal to 4–6 dentary teeth. Dentary with one row of >16 teeth, 4–7 arrowhead shaped anteriorly, all others conical posteriorly. Posterodorsal and posteroventral dentary processes abuts ventral. Dentary posteroventral process shorter than or almost as long as posterodorsal, narrow distally. Dentary ventral margin lamella large, depth greater than posterior process. Dentary anteroventral margin without a hook. Anguloarticular process long, extending beyond ventral margin of dentary. Retroarticular with an arched lamella posteriorly forming a small canal, posterior margin squared. Endopterygoid superior and inferior portions approximately equal in size, ascending process robust, straight, tip simple. Interopercle dorsal margin ascending process present. Dorsal region of hyomandibula with four lateral foramenae, supraorbital and infraorbital nerves divided. Preopercle anteroventral notch present, posterodorsal laterosensory ramus with 2 superficial pores, margin of medial shelf smooth, median shelf large, greater than half width of symplectic. Opercle dorsal margin straight, posterior margin smooth. Cranial fontanels closed in juveniles and adults. Frontal anterior margin narrow, postorbital process broad, greater than two times width of supraorbital canal. Lateral ethmoid unossified. Parietal rectangular, length equal to width. Pterophenoid anteroventral process robust, extends to lateral process. Parasphenoid posterior processes robust. Prootic foramen Vp separate from V2-3+VII. Adductor mandibula insertion undivided, intermusculars absent. All basibranchials unossified. Gill rakers not contacting gill bar.

Cleithrum broad with curved ventral margin, anterior limb long, greater than 1.8 times ascending limb, with facet for insertion of muscle from supracleithrum. Postcleithrum thin, discoid or sickle shaped. Body cavity short, with 31–34 precaudal vertebrae. Rib 5 robust along its entire extent, less than three times width of rib 6. Displaced hemal spines absent. Pectoral fin variable, with 11–16 rays. Anal fin long, with 141–259 rays. Lateral-line complete, with 14–28 ventral rami. Lateral-line dorsal rami absent in adults. Single hypaxial electric organ, extending along entire ventral margin of body with 3 rows of electroplates near caudal insertion of anal fin.

## *Gymnotus* (*Gymnotus*) *curupira* crampton, thorsen & albert

### Materials examined in morphological analyses

MZUSP 60607 (Holotype), 235 mm, Brazil, Amazonas, Tefé, Rio Tefé, Lago Tefé, Igarapé Curupira, terra firme swamp (03°26’01”S, 064°43’47”W); IDSM 425 (Paratype), 172 mm, Brazil, Amazonas, Tefé, Rio Tefé, Lago Tefé, Igarapé Repartimento, terra firme swamp (3°24’28”S, 064°44’10”W); INPA 18381 (Paratype), 129 mm, same locality as IDSM 425; INPA 18382 (Paratype), 155 mm, Brazil, Amazonas, Tefé; INPA 60608 (Paratypes) (3), 102–135 mm, same locality as IDSM 425; INPA 60609 (Paratypes) (3), 87–116 mm, same locality as IDSM 425; MZUSP 75143 (Paratype), 102 mm, same locality as IDSM 425; MZUSP 75144 (Paratype), 99 mm, same locality as IDSM 425; MZUSP 75149 (Paratype), 194 mm, same locality as 18382; MZUSP 75150 (Paratype), 143 mm, same locality as IDSM 425; MZUSP 75151 (Paratype), 121 mm, same locality as IDSM 425; MZUSP 75145 (Paratype), 150 mm (c&s), same locality as MZUSP 60607; MZUSP 75146 (Paratype), 141 mm (c&s), same locality as MZUSP 60607; MZUSP 75147 (Paratype), 173 mm, same locality as MZUSP 60607; MZUSP 75148 (Paratype), 196 mm, same locality as MZUSP 60607; MCZ 51710 (7), 89–197 mm, Ecuador, Napo, Rio Napo, Rio Payamino, Puerto Coca (~00°29’S, 076°58’W); UF 144627 (2), 148–164 mm, Peru, Loreto, Rio Ucayali, small quebrada 3 km South of Jenaro Herrera (04°55.63’S, 73°39.27’W); UF 144628 (2), 79–144 mm (c&s), Peru, Loreto, Rio Ucayali, small quebrada 0.3 km north of km-3.9 on abandoned road from Jenaro Herrera to Colonia Angamos (04°53.90’S, 073°38.36’W); NRM 27647,189 mm, Peru, Loreto, Rio Putumayo, El Estrecho (02°28’S, 072°42’W).

### Diagnosis

*Gymnotus (Gymnotus) curupira* can be differentiated from all other members of the subgenus on the basis of the following characters: color pattern with majority of dark bands divided ventrally to form inverted-“Y” shapes (vs. no bands forming inverted-“Y” shapes in all other *Gymnotus* except *G*. *(G*.*) choco*, with 1–3 inverted-“Y” shapes).

### Description

Sexually monomorphic. Size up to 235 mm TL with adult body proportions attained at about 150 mm TL. Adult body shape subcylindrical with a mean ratio of body width to depth of 75.8%. Body profile slender, body depth 73.0–96.4% total length. Head length short, 8.8–10.5% total length. Snout length moderate, 34.3–36.0% head length. Mouth width narrow, 41.4–56.8% head length. Preanal distance long, 74.3–98.0%head length. Anal-fin long, 78.7–83.8% total length. Cycloid or ovoid scales present on entire post-cranial portion of body from nape to caudal appendage.

Scales above lateral line intermediate, in 5–8 rows. Scales over anal-fin pterygiophores large, with 7–8 rows. Gape large, extending to or beyond posterior nares. Mouth position superior, lower jaw longer than upper, rictus decurved. Chin round in lateral, dorsal profiles, fleshy and bulbous with mental electroreceptive organ overlying lower jaw. Anterior narial pore partially or entirely included within gape, in small narial fold. Anterior nares small, its diameter less than that of eye. Eye below horizontal with mouth. Circumorbital series ovoid. Premaxilla with >11 teeth disposed in two rows along outer margin, median margin curved. Maxilla-palatine articulation near tip of endopterygoid. Maxilla rod- or paddle-shaped, narrow distally with a straight ventral margin, length equal to 4–6 dentary teeth. Dentary with one row of 12–15 teeth, 4–7 arrowhead shaped anteriorly, all others conical posteriorly. Posterodorsal and posteroventral dentary processes abuts ventral. Dentary posteroventral process shorter than or almost as long as posterodorsal, narrow distally. Dentary ventral margin lamella large, depth greater than posterior process. Dentary anteroventral margin without a hook. Anguloarticular process long, extending beyond ventral margin of dentary. Retroarticular with an arched lamella posteriorly forming a small canal, posterior margin squared. Endopterygoid superior and inferior portions approximately equal in size, ascending process robust, straight, tip simple. Interopercle dorsal margin ascending process present. Dorsal region of hyomandibula with four lateral foramenae, supraorbital and infraorbital nerves divided. Preopercle anteroventral notch absent, posterodorsal laterosensory ramus with one superficial pores, margin of medial shelf smooth, median shelf large, greater than half width of symplectic. Opercle dorsal margin straight, posterior margin smooth. Cranial fontanels closed in juveniles and adults. Frontal anterior margin straight, postorbital process narrow, less than two times width of supraorbital canal. Lateral ethmoid unossified. Parietal rectangular, length equal to width. Pterophenoid anteroventral process robust, extends to lateral process. Parasphenoid posterior processes robust. Prootic foramen Vp separate from V2-3+VII. Adductor mandibula insertion undivided, intermusculars absent. All basibranchials unossified. Gill rakers not contacting gill bar.

Cleithrum broad with curved ventral margin, anterior limb long, greater than 1.8 times ascending limb, with facet for insertion of muscle from supracleithrum. Postcleithrum thin, discoid or sickle shaped. Body cavity long, with 34–36 precaudal vertebrae. Rib 5 robust along its entire extent, less than three times width of rib 6. Displaced hemal spines absent. Pectoral fin broad, with 16–17 rays. Anal fin long, with 230–322 rays. Lateral-line complete. Lateral-line dorsal rami absent in adults. Single hypaxial electric organ, extending along entire ventral margin of body with 3 rows of electroplates near caudal insertion of anal fin.

## *Gymnotus (Gymnotus)* diamantinensis campos da paz

### Materials examined in morphological analyses

MZUSP 57505 (Holotype), 125 mm, Brazil, Mato Grosso, Diamantina, creek (riacho 1) tributary of Rio Preto (upper Rio Arinos at Rio Tapajós system), at road to Séio Francisco (~14°20’S, 056°30’W); MZUSP 45320 (Paratype), 100 mm c&s, same locality as MZUSP 57505; MZUSP 45359, 100 mm, Brazil, Mato Grosso, Diamantina, creek (riacho 3) tributary of Rio Preto (upper Rio Arinos at Rio Tapajós system), at road to São Francisco (~14°20’S 56°30’W).

### Diagnosis

*Gymnotus (Gymnotus) diamantinensis* can be differentiated from all other members of the subgenus on the basis of the following characters: color pattern with dark band pairs replaced by intermediate-sized (3–4 scales), irregularly-shaped, evenly-spaced dark blotches (vs. banded in all other *Gymnotus*).

### Description

Sexually monomorphic. Size up to 278 mm TL with adult body proportions attained at about 150 mm TL. Adult body shape subcylindrical with a mean ratio of body width to depth of 69.0%. Body profile slender, body depth 80.3–84.0% total length. Head length variable, 10.8–14.3% total length. Snout length moderate, 35.0–35.2% head length. Mouth width narrow, 39.0–44.1% head length. Preanal distance long, 72.7–74.0%head length. Anal-fin long, 76.8–78.9% total length. Cycloid or ovoid scales present on entire post-cranial portion of body from nape to caudal appendage.

Scales above lateral line intermediate, in 5–7 rows. Scales over anal-fin pterygiophores large, with 9 rows. Gape large, extending to or beyond posterior nares. Mouth position superior, lower jaw longer than upper, rictus decurved. Chin round in lateral, dorsal profiles, fleshy and bulbous with mental electroreceptive organ overlying lower jaw. Anterior narial pore partially or entirely included within gape, in small narial fold. Anterior nares small, its diameter less than that of eye. Eye below horizontal with mouth. Circumorbital series ovoid. Premaxilla with >11 teeth disposed in two rows along outer margin, median margin curbed. Maxilla-palatine articulation near tip of endopterygoid. Maxilla rod- or paddle-shaped, narrow distally with a straight ventral margin, length equal to 4–6 dentary teeth. Dentary with one row of >16 teeth, 4–7 arrowhead shaped anteriorly, all others conical posteriorly. Posterodorsal and posteroventral dentary processes abuts ventral. Dentary posteroventral process shorter than or almost as long as posterodorsal, narrow distally. Dentary ventral margin lamella large, depth greater than posterior process. Dentary anteroventral margin without a hook. Anguloarticular process long, extending beyond ventral margin of dentary. Retroarticular with an arched lamella posteriorly forming a small canal, posterior margin squared. Endopterygoid superior and inferior portions approximately equal in size, ascending process robust, curved, tip simple. Interopercle dorsal margin ascending process present. Dorsal region of hyomandibula with four lateral foramenae, supraorbital and infraorbital nerves divided. Preopercle anteroventral notch present, posterodorsal laterosensory ramus with 2 superficial pores, margin of medial shelf smooth, median shelf large, greater than half width of symplectic. Opercle dorsal margin straight, posterior margin smooth. Cranial fontanels closed in juveniles and adults. Frontal anterior margin straight, postorbital process broad, greater than two times width of supraorbital canal. Lateral ethmoid unossified. Parietal rectangular, length equal to width. Pterophenoid anteroventral process robust, extends to lateral process. Parasphenoid posterior processes narrow. Prootic foramen Vp separate from V2-3+VII. Adductor mandibula insertion undivided, intermusculars absent. All basibranchials unossified. Gill rakers not contacting gill bar.

Cleithrum narrow with straight ventral margin, anterior limb long, greater than 1.8 times ascending limb, large facet for insertion of muscle from supracleithrum. Postcleithrum thin, discoid or sickle shaped. Body cavity intermediate, with 33–35 precaudal vertebrae. Rib 5 robust along its entire extent, less than three times width of rib 6. Displaced hemal spines absent. Pectoral fin intermediate, with 14–15 rays. Anal fin long, with 194–211 rays. Lateral-line complete. Lateral-line dorsal rami absent in adults. Single hypaxial electric organ, extending along entire ventral margin of body with 3 rows of electroplates near caudal insertion of anal fin.

## *Gymnotus* (*Gymnotus*) *eyra* craig, correa-roldán, ortega, crampton & albert

### Materials examined in morphological analyses

MUSM 60276 (Holotype), 122, Peru, Madre de Dios, Manu, Cuenca Río Los Amigos, Aguajal cicra Pozo Pedro (12°33.611’S, 070°06.593’W); MUSM 21404 (Paratypes) (5), 51–119, same locality as MUSM 60276; AUM 23644, 152 mm, Bolivia, Beni, Rio Beni, Rio Madeira drainage, 26 km SSW of Riberalta (11°07’00”S, 066°11’00”W); INHS 37119 (5), 94–176 mm, Bolivia, Beni, Rio Beni, Rio Madeira drainage, Cuneta (borrow pit) 3 km E Estac. (14°47’32.280”S, 066°12’59.688”W); MUSM 14021, 185 mm, Peru, Madre de Dios, Manu, Rio Madre de Dios, Quebrada Pachija (11°57’S, 071°17’W); MUSM 19993 (2), 132–148 mm, Peru, Madre de Dios, Manu, Rio Madre de Dios, Los Amigos, Aguajal (12°33’00”S, 070°00’36”W); MUSM 21388 (6), 67–132 mm, Peru, Madre de Dios, Cuenca Rio Los Amigos, cerca Aguajal, Pozo Pedro (12°33’00”S, 070°06’00”W); MUSM 21778, 147 mm, Peru, Madre de Dios, Madre de Dios, Tambopata, Cuenca Río Madre de Dios, Aguajal, Aguas Negras, Pozo Santa Elena; MUSM 36141 (2), 199–254 mm, Peru, Cusco, La Convención, Echarate, Rio Urubamba Basin, Rio Parotori, Quebrada Piriabindeni (11°46’59.880”S, 073°05’60.000”W).

### Diagnosis

*Gymnotus (Gymnotus) eyra* can be differentiated from all other members of the subgenus except *G*. *(G*.*) mamiraua* and *G*. *(G*.*) obscurus* on the basis of the following characters: color pattern with wide (>5X width of interbands) dark bands, sharp band margins and pale bands extending to dorsal mid-line (vs. spotted, or with dark bands <5X width of interbands and interbands restricted to the ventral part of the lateral surface anteriorly in all other *Gymnotus*). *Gymnotus (Gymnotus) eyra* can be differentiated from *G*. *(G*.*) mamiraua* and *G*. *(G*.*) obscurus* on the basis of the following characters: few pectoral-fin rays (P1R 10–13 vs. 12–16 in *G*. *(G*.*) mamiraua*, 20–22 in *G*. *obsucurus*). *Gymnotus (Gymnotus) eyra* can be differentiated from *G*. *(G*.*) mamiraua* on the basis of the following characters: fewer ventral lateral-line rami (VLR 10–15 vs. 16–18 in *G*. *(G*.*) mamiraua*).

### Description

Sexually monomorphic. Size up to 254 mm TL with adult body proportions attained at about 150 mm TL. Adult body shape subcylindrical with a mean ratio of body width to depth of 55.4%. Body profile slender, body depth 85.4–114.5% total length. Head length short, 8.9–12.3% total length. Snout length moderate, 32.0–39.9% head length. Mouth width narrow, 30.9–43.9% head length. Preanal distance long, 61.3–84.5%head length. Anal-fin long, 78.5–84.9% total length. Cycloid or ovoid scales present on entire post-cranial portion of body from nape to caudal appendage.

Scales above lateral line intermediate, in 4–6 rows. Scales over anal-fin pterygiophores large, with 6–8 rows. Gape large, extending to or beyond posterior nares. Mouth position superior, lower jaw longer than upper, rictus decurved. Chin round in lateral, dorsal profiles, fleshy and bulbous with mental electroreceptive organ overlying lower jaw. Anterior narial pore partially or entirely included within gape, in small narial fold. Anterior nares small, its diameter less than that of eye. Eye below horizontal with mouth. Circumorbital series ovoid. Premaxilla with >11 teeth disposed in two rows along outer margin, median margin curved. Maxilla-palatine articulation near tip of endopterygoid. Maxilla rod- or paddle-shaped, narrow distally with a curved ventral margin, length equal to 4–6 dentary teeth. Dentary with one row of 12–15 teeth, 4–7 arrowhead shaped anteriorly, all others conical posteriorly. Posterodorsal and posteroventral dentary processes abuts ventral. Dentary posteroventral process shorter than or almost as long as posterodorsal, narrow distally. Dentary ventral margin lamella large, depth greater than posterior process. Dentary anteroventral margin without a hook. Anguloarticular process long, extending beyond ventral margin of dentary. Retroarticular with an arched lamella posteriorly forming a small canal, posterior margin squared. Endopterygoid superior and inferior portions approximately equal in size, ascending process robust, straight, tip complex. Interopercle dorsal margin ascending process present. Dorsal region of hyomandibula with four lateral foramenae, supraorbital and infraorbital nerves divided. Preopercle anteroventral notch present, posterodorsal laterosensory ramus with 2 superficial pores, margin of medial shelf smooth, median shelf large, greater than half width of symplectic. Opercle dorsal margin convex, posterior margin smooth. Cranial fontanels closed in juveniles and adults. Frontal anterior margin straight, postorbital process broad, greater than two times width of supraorbital canal. Lateral ethmoid unossified. Parietal rectangular, length equal to width. Pterophenoid anteroventral process robust, extends to lateral process. Parasphenoid posterior processes robust. Prootic foramen Vp separate V2-3+VII. Adductor mandibula insertion undivided, intermusculars absent. All basibranchials unossified. Gill rakers not contacting gill bar.

Cleithrum broad with straight ventral margin, anterior limb short, less than 1.8 times ascending limb, without facet for insertion of muscle from supracleithrum. Postcleithrum thin, discoid or sickle shaped. Body cavity intermediate, with 32–34 precaudal vertebrae. Rib 5 robust along its entire extent, less than three times width of rib 6. Displaced hemal spines absent. Pectoral fin narrow, with 10–13 rays. Anal fin long, with 155–227 rays. Lateral-line complete, with 10–15 ventral rami. Lateral-line dorsal rami absent in adults. Single hypaxial electric organ, extending along entire ventral margin of body with 3 rows of electroplates near caudal insertion of anal fin.

## *Gymnotus (Gymnotus) interruptus* rangel-pereira

### Materials examined in morphological analyses

UFRJ 8218 (Holotype), 91 mm, Brazil, Bahia, Iguaí, Rio de Contas basin, Rio Gongogi drainage, Riacho Cambiriba, Guaíra balneary (14°36’16,7’S, 040°06’08,7”W); UFRJ 8219 (Paratype), 121.1 mm, same locality as UFRJ 8218; UFRJ 8243 (Paratype), 80 mm (c&s), same locality as UFRJ 8218.

### Diagnosis

*Gymnotus (Gymnotus) interruptus* can be differentiated from all other members of the subgenus except *G*. *(G*.*) bahianus* on the basis of the following characters: color pattern with dark band pairs replaced by very small (1–2 scales across), rounded dark spots over entire body of most specimens, except for posteroventral 25% of some specimens (vs. banded or with larger (3 or more scales across), irregularly-shaped spots in all other *Gymnotus*). *Gymnotus (Gymnotus) interruptus* can be differentiated from *G*. *(G*.*) bahianus* on the basis of the following characters: broad interorbital distance (IO 44.6–45.9% HL vs. 33.9–42.5% in *G*. *(G*.*) bahianus*), many scales above lateral line (SAL 9 vs. 7 in *G*. *(G*.*) bahianus*).

### Description

Sexually monomorphic. Size up to 121 mm TL with adult body proportions attained at about 150 mm TL. Head length long, 12.8–14.3% total length. Anal-fin long, 80.8–82.0% total length. Cycloid or ovoid scales present on entire post-cranial portion of body from nape to caudal appendage.

Scales above lateral line intermediate, in 9 rows. Gape large, extending to or beyond posterior nares. Mouth position superior, lower jaw longer than upper, rictus decurved. Chin round in lateral, dorsal profiles, fleshy and bulbous with mental electroreceptive organ overlying lower jaw. Anterior narial pore partially or entirely included within gape, in small narial fold. Anterior nares small, its diameter less than that of eye. Eye below horizontal with mouth. Circumorbital series ovoid. Premaxilla with >11 teeth disposed in two rows along outer margin, median margin curved. Maxilla-palatine articulation near tip of endopterygoid. Maxilla rod- or paddle-shaped, narrow distally with a straight ventral margin, length equal to 4–6 dentary teeth. Dentary with one row of 12–15 teeth, 4–7 arrowhead shaped anteriorly, all others conical posteriorly. Posterodorsal and posteroventral dentary processes abuts ventral. Dentary posteroventral process shorter than or almost as long as posterodorsal, narrow distally. Dentary ventral margin lamella large, depth greater than posterior process. Dentary anteroventral margin without a hook. Retroarticular with an arched lamella posteriorly forming a small canal, posterior margin squared. Endopterygoid superior and inferior portions approximately equal in size, ascending process robust, curved, tip simple. Interopercle dorsal margin ascending process present. Dorsal region of hyomandibula with four lateral foramenae, supraorbital and infraorbital nerves divided. Preopercle anteroventral notch present, posterodorsal laterosensory ramus with 2 superficial pores, margin of medial shelf smooth, median shelf large, greater than half width of symplectic. Opercle dorsal margin straight, posterior margin smooth. Cranial fontanels closed in juveniles and adults. Frontal anterior margin straight, postorbital process broad, greater than two times width of supraorbital canal. Lateral ethmoid unossified. Parietal rectangular, length equal to width. Pterophenoid anteroventral process robust, extends to lateral process. Parasphenoid posterior processes robust. Prootic foramen Vp separate from V2-3+VII. Adductor mandibula insertion undivided, intermusculars absent. All basibranchials unossified. Gill rakers not contacting gill bar.

Cleithrum broad with curved ventral margin, anterior limb short, less than 1.8 times ascending limb, with facet for insertion of muscle from supracleithrum. Postcleithrum thin, discoid or sickle shaped. Body cavity intermediate, with 33 precaudal vertebrae. Rib 5 robust along its entire extent, less than three times width of rib 6. Displaced hemal spines absent. Pectoral fin broad, with 17 rays. Anal fin long, with 219 rays. Lateral-line complete, with 23–28 ventral rami. Lateral-line dorsal rami absent in adults. Single hypaxial electric organ, extending along entire ventral margin of.

## *Gymnotus (Gymnotus) mamiraua* albert & crampton

### Materials examined in morphological analyses

INPA 13503 (Holotype), 178 mm, Brazil, Amazonas, Tefé, Cano do Lago Rato, Mamiraua Reserve (03°02’36"S, 064°51’02"W); INPA 15832 (Paratypes) (20), 34–232 mm, same locality as INPA 13503; BMNH 1998.3.11.289, 172 mm, Brazil, Amazonas, Tefé, Lago Caetano, Jaraua Lake System, Mamirauá Reserve (02°50’05”S, 064°55’58”W); BMNH 1998.3.11.91, 182 mm, Brazil, Amazonas, Tefé, Cano do Lago Sapucaia, Mamirauá lake system (03°02’55”S, 064°49’02”W); BMNH 1998.3.11.92, 230 mm, Brazil, Amazonas, Tefé, Lago Pirarara, Mamirauá lake system (02°56’54”S, 064°50’00”W); BMNH 1998.3.11:69–75 (7), 102–226 mm, Brazil, Amazonas, Tefé, Lago Iuruazinho, 10 km west of ‘Boca do Mamirauá’ (03°02’36”S, 064°51’02”W); BMNH 1998.3.11:76–78 (3), 162–198 mm, same locality as BMNH 1998.3.11:69–75; BMNH 1998.3.11:79–80 (2), 192–225 mm, Brazil, Amazonas, Tefé, Lago Arauaé, Mamirauá lake system, 3 km west of ‘Boca Mamirauá’, Alvarães (03°02’58”S, 064°49’44”W); BMNH 1998.3.11:81–82 (2), 220–228 mm, Brazil, Amazonas, Tefé, Cano do Lago Rato, Mamirauá Lake System (03°02’48”S, 064°51’22”W); BMNH 1998.3.11:83–84 (2), 195–204 mm, same locality as BMNH 1998.3.11:79–80; BMNH 1998.3.11:85–86 (2), 224–240 mm, Brazil, Amazonas, Tefé, Ressaca Vila Alencar, Lago Mamirauá, Mamirauá lake system (03°07’49”S, 064°47’58”W); BMNH 1998.3.11.87, 162 mm, same locality as BMNH 1998.3.11.91; BMNH 1998.3.11:88, 212 mm, Brazil, Amazonas, Tefé, Lago Aracazinho, Mamirauá Lake System (02°59’49”S, 064°51’44”W); BMNH 1998.3.11:90, 180 mm, same locality as BMNH 1998.3.11:88; BMNH 1998.3.12.12, 88 mm, same locality as BMNH 1998.3.11.91; INPA 13504 (6), 99–219 mm, Brazil, Amazonas, Tefé, Lago Rato, nr. Lago Mamirauá, Mamirauá Reserve; INPA 4716, 4, 195–207 mm, same locality as BMNH 1998.3.11.289; INPA 9962, 12, 65–220 mm, Brazil, Amazonas, Tefé, Ilha Janauaca, Lago do Castanho, Rio Solimões nr. Manaus (02°18’00”S, 060°02’54”W); MCP 33282, 191 mm, Brazil, Amazonas, Alvarães, Lago Mamirauá system, Lago Comprido (03°4’53.000”S, 064°46’47.996”W); MCP 33283 (2), 175–195 mm, Brazil, Amazonas, Alvarães, Lago Mamirauá system, 0.5 Km SW of Boca do Mamirauá village (03°06’44.000”S, 64°48’1.000”W); MCP 33349 (3), 199–206 mm, same locality as MCP 33283; WGRC 02.010796, 216 mm, Brazil, Amazonas, Tefé, Lago Arauaé, Mamirauá Lake System (03°02’87”S, 64°50’08”W); WGRC 07.100497, 92 mm, Brazil, Amazonas, same locality as WGRC 02.010796; WGRC 04.170497, 53 mm, Brazil, Amazonas, Tefé, Cano Sapucaia, Mamirauá Lake System, (03°04’09”S, 64°48’53”W); WGRC 10.180497, 34 mm, same locality as BMNH 1998.3.11:81–82; WGRC 13.180497, 36 mm, same locality as BMNH 1998.3.11:81–82; WGRC 02.300497, 192 mm, same locality as BMNH 1998.3.11:81–82; WGRC 34.070597, 61 mm, same locality as BMNH 1998.3.11:85–86; WGRC 22.150597, 205 mm, same locality as BMNH 1998.3.11.289; WGRC 01.150398, 212 mm, Brazil, Amazonas, Tefé, Lago Secretaria, Mamirauá Lake System (03°07’20”S, 064°48’70”W); WGRC 02.300498, 232 mm, same locality as WGRC 01.150398; WGRC 04.060598, 201 mm, Brazil, Amazonas, Tefé, Lago Periquito Comprido, Mamirauá Lake System (03°05’65”S, 064°47’60”W); WGRC 09.290598, 210 mm, Brazil, Amazonas, Tefé, Lago Geraldo, Mamirauá Lake System (03°06’95”S, 064°49’16”W); WGRC 01.050698, 217 mm, Brazil, Amazonas, Tefé, Lago Tracaja, Mamirauá Lake System (03°06’13”S, 64°14’13”W); WGRC 02.050698, 210 mm, same locality as WGRC 01.050698; WGRC 04.060698, 175 mm, Brazil, Amazonas, Tefé, Lago Promessinha, Mamirauá Lake System (02°59’49”S, 064°47’11”W); WGRC 07.060698, 172 mm, same locality as WGRC 04.060698; WGRC 02.070698, 226 mm, Brazil, Amazonas, Tefé, Lago Aragazinho, Mamirauá Lake System (42°59’49”S, 064°51’44”W); WGRC 07.160698, 206 mm, Brazil, Amazonas, Tefé, Cano Mamirauá, Mamirauá Lake System (03°00’37”S, 064°48’26”W); WGRC17.160698, 222 mm, same locality as WGRC 07.160698.

### Diagnosis

*Gymnotus (Gymnotus) mamiraua* can be differentiated from all other members of the subgenus except *G*. *(G*.*) eyra* and *G*. *(G*.*) obscurus* on the basis of the following characters: color pattern with wide (>5X width of interbands) dark bands, sharp band margins and pale bands extending to dorsal mid-line (vs. spotted, or with dark bands <5X width of interbands and interbands restricted to the ventral part of the lateral surface anteriorly in all other *Gymnotus*). *Gymnotus (Gymnotus) mamiraua* and can be differentiated from *G*. *(G*.*) eyra* and *G*. *(G*.*) obscurus* on the basis of the following characters: intermediate number of pectoral-fin rays (P1R 12–16 vs. 10–13 in *G*. *(G*.*) eyra*, 20–22 in *G*. *(G*.*) obscurus*). *Gymnotus (Gymnotus) mamiraua* and can be differentiated from *G*. *(G*.*) eyra* on the basis of the following characters: more ventral lateral-line rami (VLR 16–18 vs. 10–15 in *G*. *(G*.*) eyra*).

### Description

Sexually monomorphic. Size up to 254 mm TL with adult body proportions attained at about 150 mm TL. Adult body shape subcylindrical with a mean ratio of body width to depth of 55.4%. Body profile slender, body depth 85.4–114.5% total length. Head length short, 8.9–12.3% total length. Snout length moderate, 32.0–39.9% head length. Mouth width narrow, 30.9–43.9% head length. Preanal distance long, 61.3–84.5%head length. Anal-fin long, 78.5–84.9% total length. Cycloid or ovoid scales present on entire post-cranial portion of body from nape to caudal appendage.

Scales above lateral line intermediate, in 4–6 rows. Scales over anal-fin pterygiophores large, with 6–8 rows. Gape large, extending to or beyond posterior nares. Mouth position superior, lower jaw longer than upper, rictus decurved. Chin round in lateral, dorsal profiles, fleshy and bulbous with mental electroreceptive organ overlying lower jaw. Anterior narial pore partially or entirely included within gape, in small narial fold. Anterior nares small, its diameter less than that of eye. Eye below horizontal with mouth. Circumorbital series ovoid. Premaxilla with >11 teeth disposed in two rows along outer margin, median margin curved. Maxilla-palatine articulation near tip of endopterygoid. Maxilla rod- or paddle-shaped, narrow distally with a straight ventral margin, length equal to 4–6 dentary teeth. Dentary with one row of 12–15 teeth, 4–7 arrowhead shaped anteriorly, all others conical posteriorly. Posterodorsal and posteroventral dentary processes abuts ventral. Dentary posteroventral process shorter than or almost as long as posterodorsal, narrow distally. Dentary ventral margin lamella large, depth greater than posterior process. Dentary anteroventral margin without a hook. Anguloarticular process long, extending beyond ventral margin of dentary. Retroarticular with an arched lamella posteriorly forming a small canal, posterior margin squared. Endopterygoid superior and inferior portions approximately equal in size, ascending process robust, straight, tip complex. Interopercle dorsal margin ascending process present. Dorsal region of hyomandibula with four lateral foramenae, supraorbital and infraorbital nerves divided. Preopercle anteroventral notch present, posterodorsal laterosensory ramus with 2 superficial pores, margin of medial shelf smooth, median shelf large, greater than half width of symplectic. Opercle dorsal margin straight, posterior margin smooth. Cranial fontanels closed in juveniles and adults. Frontal anterior margin straight, postorbital process broad, greater than two times width of supraorbital canal. Lateral ethmoid unossified. Parietal rectangular, length equal to width. Pterophenoid anteroventral process robust, extends to lateral process. Parasphenoid posterior processes robust. Prootic foramen Vp separate from V2-3+VII. Adductor mandibula insertion undivided, intermusculars absent. All basibranchials unossified. Gill rakers not contacting gill bar.

Cleithrum broad with curved ventral margin, anterior limb short, less than 1.8 times ascending limb, without facet for insertion of muscle from supracleithrum. Postcleithrum thin, discoid or sickle shaped. Body cavity intermediate, with 32–34 precaudal vertebrae. Rib 5 robust along its entire extent, less than three times width of rib 6. Displaced hemal spines absent. Pectoral fin narrow, with 11–13 rays. Anal fin long, with 155–227 rays. Lateral-line complete, with 10–15 ventral rami. Lateral-line dorsal rami absent in adults. Single hypaxial electric organ, extending along entire ventral margin of body with 3 rows of electroplates near caudal insertion of anal fin.

## *Gymnotus* (*Gymnotus*) *obscurus* crampton, thorsen & albert

### Materials examined in morphological analyses

MZUSP 60604 (Holotype), 215 mm, Brazil, Amazonas, Álvares, Mamirauá Reserve, Cano do Lago Mamirauá at Comunidade Boca do Mamirauá (03°06’37”S, 064°47’49”W); BMNH 1998.3.12.19 (Paratype), 161 mm, Brazil, Amazonas, Álvares, Mamirauá Reserve, Ressaca da Vila Alencar (03°07’42”S, 064°48’02”W); BMNH 1998.3.12.20 (Paratype), 151 mm, same locality as BMNH 1998.3.12.19; BMNH 1998.3.12.21 (Paratype), 94 mm (c&s), same locality as BMNH 1998.3.12.19; IDSM 432 (Paratype), 110 mm, same locality as BMNH 1998.3.12.19; INPA 18388 (Paratypes) (2), 111–154 mm, Brazil, Amazonas, Álvares, Mamirauá Reserve, Cano do Lago Sapucaia (03°04’07”S, 064°48’32”W); MZUSP 75152 (Paratype), 160 mm, same locality as INPA 18388; MZUSP 60605 (Paratype), 161 mm (c&s), same locality as BMNH 1998.3.12.19; MZUSP 60606 (Paratype), 122 mm, Brazil, Amazonas, Álvares, Mamirauá Reserve, Cano do Lago Mamirauá near Cano do Lago Arauaé (03°01’38”S, 064°52’35”W); MZUSP 75153 (Paratype), 167 mm, Brazil, Amazonas, Álvares, Mamirauá Reserve, Lago Secretaria (03°06’44”S, 064°48’01’W); MZUSP 75154 (Paratype), 177 mm, same locality as MZUSP 60604; MZUSP 75155 (Paratype), 141 mm, Brazil, Amazonas, Álvares, Mamirauá Reserve, Ressaca do Pau (03°02’18”S, 064°51’58”W); MZUSP 75156 (Paratype), 175 mm, same locality as MZUSP 75155; MZUSP 75157 (Paratype), 165 mm, same locality as MZUSP 75155; UF 118833 (Paratypes) (2), 103–112 mm, same locality as BMNH 1998.3.12.19; UF 118837 (Paratype), 171 mm, same locality as BMNH 1998.3.12.19; UF 118836 (Paratype), 144 mm, Brazil, Amazonas, Álvares, Mamirauá Reserve, Cano do Lago Rato (03°02’58”S, 064°51’31”W).

### Diagnosis

*Gymnotus (Gymnotus) obscurus* can be differentiated from all other members of the subgenus except *G*. *(G*.*) eyra* and *G*. *(G*.*) mamiraua* on the basis of the following characters: color pattern with wide (>5X width of interbands) dark bands, sharp band margins and pale bands extending to dorsal mid-line (vs. spotted, or with dark bands <5X width of interbands and interbands restricted to the ventral part of the lateral surface anteriorly in all other *Gymnotus*). *Gymnotus (Gymnotus) obscurus* and can be differentiated from *G*. *(G*.*) eyra* and *G*. *(G*.*) mamiraua* on the basis of the following characters: many pectoral-fin rays (P1R 20–22 vs. 10–13 in *G*. *(G*.*) eyra*, 12–16 in *G*. *(G*.*) mamiraua*), many pored lateral-line scales prior to the first ventral lateral-line ramus (PLL 110–120 vs. 76–95 in *G*. *(G*.*) eyra*, 62–100 in *G*. *(G*.*) mamiraua*).

### Description

Sexually monomorphic. Size up to 215 mm TL with adult body proportions attained at about 150 mm TL. Adult body shape subcylindrical with a mean ratio of body width to depth of 63.6%. Body profile slender, body depth 82.5–98.8% total length. Head length intermediate, 9.9–12.4% total length. Snout length moderate, 34.1–39.9% head length. Mouth width narrow, 37.3–46.3% head length. Preanal distance long, 71.3–94.2%head length. Anal-fin long, 77.0–83.8% total length. Cycloid or ovoid scales present on entire post-cranial portion of body from nape to caudal appendage.

Scales above lateral line intermediate, in 4–7 rows. Scales over anal-fin pterygiophores large, with 6–7 rows. Gape large, extending to or beyond posterior nares. Mouth position superior, lower jaw longer than upper, rictus decurved. Chin round in lateral, dorsal profiles, fleshy and bulbous with mental electroreceptive organ overlying lower jaw. Anterior narial pore partially or entirely included within gape, in small narial fold. Anterior nares small, its diameter less than that of eye. Eye below horizontal with mouth. Circumorbital series ovoid. Premaxilla with <10 teeth disposed in two rows along outer margin, median margin curved. Maxilla-palatine articulation near tip of endopterygoid. Maxilla rod- or paddle-shaped, narrow distally with a curved ventral margin, length equal to 4–6 dentary teeth. Dentary with one row of 12–15 teeth, 2–4 arrowhead shaped anteriorly, all others conical posteriorly. Posterodorsal and posteroventral dentary processes abuts ventral. Dentary posteroventral process shorter than or almost as long as posterodorsal, narrow distally. Dentary ventral margin lamella large, depth greater than posterior process. Dentary anteroventral margin without a hook. Anguloarticular process long, extending beyond ventral margin of dentary. Retroarticular with an arched lamella posteriorly forming a small canal, posterior margin squared. Endopterygoid superior and inferior portions approximately equal in size, ascending process robust, straight, tip simple. Interopercle dorsal margin ascending process present. Dorsal region of hyomandibula with four lateral foramenae, supraorbital and infraorbital nerves divided. Preopercle anteroventral notch present, posterodorsal laterosensory ramus with 2 superficial pores, margin of medial shelf smooth, median shelf long, greater than half width of symplectic. Opercle dorsal margin straight, posterior margin smooth. Cranial fontanels closed in juveniles and adults. Frontal anterior margin straight, postorbital process broad, greater than two times width of supraorbital canal. Lateral ethmoid unossified. Parietal rectangular, length equal to width. Pterophenoid anteroventral process robust, extends to lateral process. Parasphenoid posterior processes robust. Prootic foramen Vp separate from V2-3+VII. Adductor mandibula insertion undivided, intermusculars absent. All basibranchials unossified. Gill rakers not contacting gill bar.

Cleithrum broad with curved ventral margin, anterior limb long, greater than 1.8 times ascending limb, with facet for insertion of muscle from supracleithrum. Postcleithrum thin, discoid or sickle shaped. Body cavity long, with 35–37 precaudal vertebrae. Rib 5 robust along its entire extent, less than three times width of rib 6. Displaced hemal spines absent. Pectoral fin broad, with 20–22 rays. Anal fin long, with 208–250 rays. Lateral-line complete. Lateral-line dorsal rami absent in adults. Single hypaxial electric organ, extending along entire ventral margin of body with 3–4 rows of electroplates near caudal insertion of anal fin.

## *Gymnotus* (*Gymnotus*) *omarorum* richer-de-forges, crampton & albert

### Materials examined in morphological analyses

ZVC-P 6480 (Holotype), 250 mm, Uruguay, Maldonado, Río Cisne drainage, Laguna del Sauce (34°50’20”S, 055°06’52”W); AMNH 239656 (16) (Paratypes), 32–222 mm, same locality as ZVC-P 6480; MCP 41266 (3) (Paratypes), 150–190 mm, same locality as ZVC-P 6480; ZVC-P 6481 (33) (Paratypes), 23–201 mm, same locality as ZVC-P 6480; ZVC-P 16, 185 mm, Uruguay, Artigas, Río Uruguay drainage, Río Cuareim, del Yucutuja, Rancho El Ombu (31°40’12”S, 057°10’12”W); ZVC-P 404 (2),160–190 mm, Uruguay, Artigas, Río Uruguay drainage, Río Cuareim, Picada Tareira (34°25’12”S, 056°25’12”W); ZVC-P 2273, 278 mm, Canal de Riego; ZVC-P 2906 (3), 95–205 mm, Uruguay, Artigas, Río Uruguay drainage, Río Cuareim, Rancho Pereira Reverbell (30°11’24”S, 057°09’36”W); ZVC-P 3407b (3), 195–240 mm, Uruguay, Artigas, Río Uruguay drainage, Catalán Chico, Rancho Martine; ZVC-P 3634, 286 mm, Salto, Uruguay, Artigas, Río Uruguay drainage, Salto City; ZVC-P 105 (2), 100–170 mm, Uruguay, Florida, Río Uruguay drainage, Laguna en Cañada Invernada (31°00’36”S, 056°00’36”W); ZVC-P 303 (2), 173–215 mm, Uruguay, Florida, Río Uruguay drainage, Río Santa Lucia (34°26’24”S, 056°23’60”W); ZVC-P 3423, 146 mm, Uruguay, Florida, Río Uruguay drainage, Cañada Milano; ZVC-P 5502, 227 mm, Río Uruguay drainage, Río Santa Lucia, Cañada Casupa; ZVC-P 950 (2), 123–158 mm, Uruguay, Lavalleja, Rio Cebollatí, Laguna Merín, Rancho Sosa Diaz, Canada Mariscala (33°26’24”S, 054°23’60”W); ZVC-P 1917, 82 mm, Uruguay, Lavalleja, Laguna Merín drainage, Villa Serrana, Cañada los Chanchos (34°19’12”S, 054°57’36”W); ZVC-P 1351, 245 mm, Uruguay, Rivera, Río Negro, Cañada Cuñapirú (31°11’60”S, 055°36’00”W); ZVC-P 7429 (3), 227–269 mm, Uruguay, Treinta y Tres, Laguna El Tigre; ZVC-P 7430, 222 mm, Uruguay, Tacuarembó, Laguna Lavalle.

### Diagnosis

*Gymnotus (Gymnotus) omarorum* can be differentiated from all other members of the subgenus except *G*. *(G*.*) bahianus* on the basis of the following characters: few pored lateral-line scales prior to the first ventral lateral-line ramus (PLL 70–87 vs. 87–106 in *G*. *(G*.*) arapaima*, 77–128 in *G*. *(G*.*) carapo*, 89–107 in *G*. *(G*.*) chaviro*, 80–106 in *G*. *(G*.*) chimarrao*, 81–103 in *G*. *(G*.*) cuia*, 104–125 in *G*. *(G*.*) curupira*, 76–95 in *G*. *(G*.*) eyra*, 75–100 in *G*. *(G*.*) mamiraua*, 108–132 in *G*. *(G*.*) obscurus*, 82–94 in *G*. *(G*.*) pantanal*, 90–103 in *G*. *(G*.*) riberalta*, 79–100 in *G*. *(G*.*) sylvius*, 106–125 in *G*. *(G*.*) varzea*). *Gymnotus (Gymnotus) omarorum* can be differentiated from *G*. *(G*.*) bahianus* on the basis of the following characters: color pattern with 24–29 obliquely-oriented, dark brown bands with wavy, irregular margins (vs. dark band pairs replaced by very small (1–2 scales across), rounded dark spots over entire body of most specimens in *G*. *(G*.*) bahianus*).

### Description

Sexually monomorphic. Size up to 262 mm TL with adult body proportions attained at about 150 mm TL. Adult body shape subcylindrical with a mean ratio of body width to depth of 33.1%. Body profile slender, body depth 84.5–102.3% total length. Head length intermediate, 10.6–12.4% total length. Snout length moderate, 34.7–38.7% head length. Mouth width narrow, 37.4–48.9% head length. Preanal distance long, 67.8–89.3%head length. Anal-fin long, 81.6–85.9% total length. Cycloid or ovoid scales present on entire post-cranial portion of body from nape to caudal appendage.

Scales above lateral line intermediate, in 5–7 rows. Scales over anal-fin pterygiophores large, with 5–6 rows. Gape large, extending to or beyond posterior nares. Mouth position superior, lower jaw longer than upper, rictus decurved. Chin round in lateral, dorsal profiles, fleshy and bulbous with mental electroreceptive organ overlying lower jaw. Anterior narial pore partially or entirely included within gape, in small narial fold. Anterior nares small, its diameter less than that of eye. Eye below horizontal with mouth. Circumorbital series ovoid. Premaxilla with >11 teeth disposed in two rows along outer margin, median margin curved. Maxilla-palatine articulation near tip of endopterygoid. Maxilla rod- or paddle-shaped, narrow distally with a straight ventral margin, length equal to 4–6 dentary teeth. Dentary with one row of >16 teeth, 4–7 arrowhead shaped anteriorly, all others conical posteriorly. Posterodorsal and posteroventral dentary processes abuts ventral. Dentary posteroventral process shorter than or almost as long as posterodorsal, narrow distally. Anguloarticular process long, extending beyond ventral margin of dentary. Retroarticular with an arched lamella posteriorly forming a small canal, posterior margin squared. Endopterygoid superior and inferior portions approximately equal in size, ascending process robust, straight, tip simple. Interopercle dorsal margin ascending process present. Dorsal region of hyomandibula with four lateral foramenae, supraorbital and infraorbital nerves divided. Preopercle anteroventral notch present, posterodorsal laterosensory ramus with 2 superficial pores, margin of medial shelf smooth, median shelf large, greater than half width of symplectic. Opercle dorsal margin straight, posterior margin smooth. Cranial fontanels closed in juveniles and adults. Frontal anterior margin straight, postorbital process broad, greater than two times width of supraorbital canal. Lateral ethmoid unossified. Parietal rectangular, length equal to width. Pterophenoid anteroventral process robust, extends to lateral process. Parasphenoid posterior processes robust. Prootic foramen Vp separate V2-3+VII. Adductor mandibula insertion undivided, intermusculars absent. All basibranchials unossified. Gill rakers not contacting gill bar.

Cleithrum broad with curved ventral margin, anterior limb broad, greater than 1.8 times ascending limb, with facet for insertion of muscle from supracleithrum. Postcleithrum thin, discoid or sickle shaped. Rib 5 robust along its entire extent, less than three times width of rib 6. Displaced hemal spines absent. Pectoral fin intermediate, with 13–14 rays. Anal fin long, with 130–184 rays. Lateral-line complete, with 28–30 ventral rami. Lateral-line dorsal rami absent in adults. Single hypaxial electric organ, extending along entire ventral margin of body with 3 rows of electroplates near caudal insertion of anal fin.

## *Gymnotus* (*Gymnotus*) *pantanal* Fernandes, Albert, Daniel-Silva, Lopes, Crampton & Almeida-Toledo

### Materials examined in morphological analyses

MZUSP 67874 (Holotype), Brazil, Mato Grosso do Sul, 196 mm, Río Miranda, near Miranda (20°11’78”S, 056°30’13”W); MZUSP 67875 (Paratype), 189 mm, Brazil, Mato Grosso do Sul, Rio Paraguarí (18°59’81”S, 057°39’24”W); MACN-ict 9655 (19), 154–257 mm, Argentina, Corrientes, Río Paraná drainage, Esteros del Riachuelo (27°34’39”S, 058°15’23”W); MZUSP 67875, 192 mm, same locality as MZUSP 67875; MZUSP 67876, 251 mm, same locality as MZUSP 67875; NUP 4554 (5), 195–235 mm, Brazil, Mato Grosso do Sul, upper Rio Paraná drainage, marginal lagoons of Rio Paraná (24°05’33”S, 054°15’17”W); NUP 6044 (2), 152–204 mm, Brazil, Mato Grosso do Sul, Água Queçaba stream; NUP 7934, 152 mm, Brazil, Mato Grosso do Sul, upper Rio Paraná drainage, Rio Paracaí (23°39’30”S, 053°55’10”W); NUP 9290 (17), 139–260 mm, same locality as NUP 9311; NUP 9311 (5), 168–210 mm, Brazil, Mato Grosso do Sul, upper Rio Paraná drainage, Rio Marreco, Jacutinga stream (24°42’56”S, 053°46’21”W); NUP 9312 (11), 113–248 mm, Brazil, Mato Grosso do Sul, upper Rio Paraná drainage, Rio Toledo, Pinheirinho stream (24°44’05”S, 053°42’55”W); UFRGS 11910, Brazil, Mato Grosso do Sul, Corumbá, Rio Paraguai drainage, Rio Miranda or Aquidauna between Corumbá and Aquidauna (23°22’47”S, 052°30’07”W); NRM 42830, 240 mm, Paraguay, Caaguazu, Río Paraná drainage, arroyo crossing Rt. 2 West of J. E. Estringarriba (25°22’40”S, 055°42’32’W”); UMMZ 206080 (21), 82–260 mm, Paraguay, Paraguarí, Río Paraguay drainage, Arroyo in Parque Nacional Ybycui (26°58’00”S, 057°19’60”W).

### Diagnosis

*Gymnotus (Gymnotus) pantanal* can be differentiated from all other members of the subgenus except *G*. *(G*.*) capanema* and *G*. *(G*.*) riberalta* on the basis of the following characters: color pattern with wide (>5X width of interbands) dark bands, where pale interbands restricted to the ventral part of the lateral surface such that the dark interbands fuse into a uniform dark coloration over anterior 60% of body (vs. banded or spotted in all other *Gymnotus*). *Gymnotus (Gymnotus) pantanal* can be differentiated from *G*. *(G*.*) capanema* and *G*. *(G*.*) riberalta* on the basis of the following characters: broad pectoral fin, possessing many rays (P1R 14–18 vs. 12–15 in *G*. *(G*.*) capanema*, 10–13 in *G*. *(G*.*) riberalta*), broad maximum body width (BW 71.5–76.7% HL vs. 53.3–72.0% HL in *G*. *(G*.*) riberalta*).

### Description

Sexually monomorphic. Size up to 251 mm TL with adult body proportions attained at about 150 mm TL. Adult body shape subcylindrical with a mean ratio of body width to depth of 74.7%. Body profile slender, body depth 77.7–11.7% total length. Head length short, 7.2–11.7% total length. Snout length moderate, 30.7–38.9% head length. Mouth width narrow, 42.0–50.7% head length. Preanal distance long, 81.7–94.8%head length. Anal-fin long, 78.1–84.2% total length. Cycloid or ovoid scales present on entire post-cranial portion of body from nape to caudal appendage.

Scales above lateral line intermediate, in 6–8 rows. Scales over anal-fin pterygiophores large, with 7–8 rows. Gape large, extending to or beyond posterior nares. Mouth position superior, lower jaw longer than upper, rictus decurved. Chin round in lateral, dorsal profiles, fleshy and bulbous with mental electroreceptive organ overlying lower jaw. Anterior narial pore partially or entirely included within gape, in small narial fold. Anterior nares small, its diameter less than that of eye. Eye below horizontal with mouth. Circumorbital series ovoid. Premaxilla with >11 teeth disposed in two rows along outer margin, median margin curved. Maxilla-palatine articulation near tip of endopterygoid. Maxilla rod-shaped, narrow distally with a straight ventral margin, length equal to 7–9 dentary teeth. Dentary with one row of <12 teeth, 2–4 arrowhead shaped anteriorly, all others conical posteriorly. Posterodorsal and posteroventral dentary processes abuts ventral. Dentary posteroventral process shorter than or almost as long as posterodorsal, narrow distally. Dentary ventral margin lamella small, depth less than posterior process. Dentary anteroventral margin with a hook. Anguloarticular process long, extending beyond ventral margin of dentary. Retroarticular with an arched lamella posteriorly forming a small canal, posterior margin squared. Endopterygoid superior and inferior portions approximately equal in size, ascending process robust, straight, tip simple. Interopercle dorsal margin ascending process present. Dorsal region of hyomandibula with four lateral foramenae, supraorbital and infraorbital nerves connected. Preopercle anteroventral notch present, posterodorsal laterosensory ramus with 1–2 superficial pores, margin of medial shelf smooth, median shelf large, greater than half width of symplectic. Opercle dorsal margin straight, posterior margin smooth. Cranial fontanels closed in juveniles and adults. Frontal anterior margin straight, postorbital process narrow, less than two times width of supraorbital canal. Lateral ethmoid unossified. Parietal rectangular, length equal to width. Pterophenoid anteroventral process reduced, extends to lateral process. Parasphenoid posterior processes robust. Prootic foramen Vp combined with V2-3+VII. Adductor mandibula insertion undivided, intermusculars absent. All basibranchials unossified. Gill rakers not contacting gill bar.

Cleithrum narrow with straight ventral margin, anterior limb long, greater than 1.8 times ascending limb, without facet for insertion of muscle from supracleithrum. Postcleithrum thin, discoid or sickle shaped. Body cavity long, with 37–38 precaudal vertebrae. Rib 5 robust along its entire extent, less than three times width of rib 6. Displaced hemal spines absent. Pectoral fin broad, with 14–18 rays. Anal fin long, with 131–280 rays. Lateral-line complete, with 13–19 ventral rami. Lateral-line dorsal rami absent in adults. Single hypaxial electric organ, extending along entire ventral margin of body with 3 rows of electroplates near caudal insertion of anal fin.

## *Gymnotus* (*Gymnotus*) *riberalta* Craig, Correa-Roldán, Ortega, Crampton & Albert

### Materials examined in morphological analyses

CBF 10248 (Holotype), 199 mm, Bolivia, Beni, Riberalta, Rio Beni, Rio Madeira drainage, Rio Amazonas drainage, Arroyo near Lago de San Jose (10°54’47”S, 065°59’49”W); CBF 10243 (Paratype), 193 mm, same locality as CBF 10249; CBF 10244, 135 mm, same locality as CBF 10249; CBF 10245 (Paratype), 220 mm, same locality as CBF 10249; CBF 10246 (Paratype), 142 mm, same locality as CBF 10249; CBF 10247 (Paratype), 196 mm, same locality as CBF 10249; CBF 10249 (Paratype), 173 mm, same locality as CBF 10249; UMSS 7008 (Paratype), 138 mm, same locality as CBF 10249; UMSS 7009 (Paratype), 153 mm, same locality as CBF 10249; UMSS 7010 (Paratype), 200 mm, same locality as CBF 10249; UMSS 7011 (Paratype), 152 mm, same locality as CBF 10249; UMSS 7012 (Paratype), 237 mm, same locality as CBF 10249; UMSS 7013 (Paratype), 209 mm, same locality as CBF 10249; UMSS 7014 (Paratype), 247 mm, same locality as CBF 10249; UMMZ 82146, 153 mm, Bolivia, Santa Cruz, Ñuflo de Chaves, Río Blanco near Concepción (~16°07’55”S, 062°01’34”W); UMSS 07015, 194 mm, Brazil, Amazonas, Rio Beni, Rio Madeira drainage, stream near Porto Hamburgo (10°54’7.751”S, 065°59’8.182”W); UMSS 07016, 216 mm, same locality as UMSS 07015; UMSS 07017 170 mm, same locality as UMSS 07015; UF 180238 (3), 187–267 mm, Bolivia, Beni, Riberalta, Rio Beni, Rio Madeira drainage, Rio Amazonas drainage, Small Stream near Puerto Hamburgo, Riberalta (11°01’52”S, 066°05’39”W, 147 m).

### Diagnosis

*Gymnotus (Gymnotus) riberalta* can be differentiated from all other members of the subgenus except *G*. *(G*.*) capanema* and *G*. *(G*.*) pantanal* on the basis of the following characters: color pattern with wide (>5X width of interbands) dark bands, where pale interbands restricted to the ventral part of the lateral surface such that the dark interbands fuse into a uniform dark coloration over anterior 60% of body (vs. banded or spotted in all other *Gymnotus*). *Gymnotus (Gymnotus) riberalta* can be differentiated from *G*. *(G*.*) capanema* and *G*. *(G*.*) pantanal* on the basis of the following characters: narrow mouth (MW 32.7–45.7% HL vs. 40.6–58.0% HL in *G*. *(G*.*) capanema*, 42.0–50.7% HL in *G*. *(G*.*) pantanal*), narrow maximum body width (BW 53.3–72.0% HL vs. 71.5–76.7% HL in *G*. *(G*.*) pantanal*), narrow pectoral fin, possessing fewer rays (P1R 10–13 vs. 12–15 in *G*. *(G*.*) capanema*, 14–18 in *G*. *(G*.*) pantanal*), more anal-fin pterygiophore scales (APS 10–11 vs. 5–8 in *G*. *(G*.*) capanema*, 7–8 in *G*. *(G*.*) pantanal*).

### Description

Sexually monomorphic. Size up to 302 mm TL with adult body proportions attained at about 150 mm TL. Adult body shape subcylindrical with a mean ratio of body width to depth of 65.0%. Body profile slender, body depth 81.1–102.1% total length. Head length short, 7.3–10.3% total length. Snout length moderate, 31.0–37.3% head length. Mouth width narrow, 32.7–45.7% head length. Preanal distance long, 71.9–97.9%head length. Anal-fin long, 79.7–97.4% total length. Cycloid or ovoid scales present on entire post-cranial portion of body from nape to caudal appendage.

Scales above lateral line intermediate, in 6–8 rows. Scales over anal-fin pterygiophores large, with 6–8 rows. Gape large, extending to or beyond posterior nares. Mouth position superior, lower jaw longer than upper, rictus decurved. Chin round in lateral, dorsal profiles, fleshy and bulbous with mental electroreceptive organ overlying lower jaw. Anterior narial pore partially or entirely included within gape, in small narial fold. Anterior nares small, its diameter less than that of eye. Eye below horizontal with mouth. Circumorbital series ovoid. Premaxilla with >11 teeth disposed in two rows along outer margin, median margin curved. Maxilla-palatine articulation near tip of endopterygoid. Maxilla rod- or paddle-shaped, narrow distally with a straight ventral margin, length equal to 7–9 dentary teeth. Dentary with one row of <12 teeth, 2–4 arrowhead shaped anteriorly, all others conical posteriorly. Posterodorsal and posteroventral dentary processes abuts ventral. Dentary posteroventral process shorter than or almost as long as posterodorsal, narrow distally. Dentary ventral margin lamella small, depth less than posterior process. Dentary anteroventral margin with a hook. Anguloarticular process long, extending beyond ventral margin of dentary. Retroarticular with an arched lamella posteriorly forming a small canal, posterior margin squared. Endopterygoid superior and inferior portions approximately equal in size, ascending process robust, straight, tip simple. Interopercle dorsal margin ascending process present. Dorsal region of hyomandibula with four lateral foramenae, supraorbital and infraorbital nerves connected. Preopercle anteroventral notch present, posterodorsal laterosensory ramus with 2 superficial pores, margin of medial shelf smooth, median shelf large, greater than half width of symplectic. Opercle dorsal margin straight, posterior margin smooth. Cranial fontanels closed in juveniles and adults. Frontal anterior margin straight, postorbital process narrow, less than two times width of supraorbital canal. Lateral ethmoid unossified. Parietal rectangular, length equal to width. Pterophenoid anteroventral process reduced, extends to lateral process. Parasphenoid posterior processes robust. Prootic foramen Vp combined with V2-3+VII. Adductor mandibula insertion undivided, intermusculars absent. All basibranchials unossified. Gill rakers not contacting gill bar.

Cleithrum narrow with straight ventral margin, anterior limb long, greater than 1.8 times ascending limb, without facet for insertion of muscle from supracleithrum. Postcleithrum thin, discoid or sickle shaped. Body cavity long, with 36 precaudal vertebrae. Rib 5 robust along its entire extent, less than three times width of rib 6. Displaced hemal spines absent. Pectoral fin narrow, with 10–13 rays. Anal fin long, with 169–240 rays. Lateral-line complete, with 10–16 ventral rami. Lateral-line dorsal rami absent in adults. Single hypaxial electric organ, extending along entire ventral margin of body with 3 rows of electroplates near caudal insertion of anal fin.

## *Gymnotus* (*Gymnotus*) *sylvius* Albert, Fernandes-Matioli & de Almeida-Toledo

### Materials examined in morphological analyses

LGP 0925.1 (Holotype), 259 mm, Brazil, São Paulo, Rio Ribeira de Iguapé, near Miracatu (24°32’50”S, 047°26’13”W); LGP 0925.2 (2) (Paratypes), 251–307 mm, same locality as LGP 0925.1; LGP 0931 (Paratype), 157 mm, Brazil, São Paulo, Rio Pardo drainage, São Simão, Rio Tamanduá (21°30’00”S, 047°31’11”W); UMMZ 234347 (2) (Paratypes), 255–271 mm, same locality as LGP 0931; LGP96 (P2338), 170 mm, same locality as LGP88; MCP 47810, 81 mm, Brazil, Paraná, Cascavel, Iguaçu drainage, Rio do Oeste, between Rio do Salto and Castelo Branco (25°09’19”S, 053°19’41”W); LGP88 (P2336), 180 mm, Brazil, São Paulo, Jacareí, Rio Paraíba do Sul; LGP93, 160 mm, same locality as LGP88; LGP94 (P2333), 230 mm, same locality as LGP88; LGP95 (P2352), 130 mm, same locality as LGP88; LGP91 (P2353), 170 mm, Brazil, São Paulo, Paraibuna, Rio Paraíba do Sul; LGP92, 280 mm, Brazil, São Paulo, Paraibuna, Rio Paraíba do Sul; LGP98 (P2330), 160 mm, same locality as LGP92; UFRGS 14743 (4), 87–168 mm, Brazil, São Paulo, Pindamonhangaba, downstream of the Bosque da Princesa, near Chacrinha (22°55’05”S, 045°27’45”W).

### Diagnosis

*Gymnotus (Gymnotus) sylvius* can be differentiated from all other members of the subgenus except *G*. *(G*.*) arapaima*, *G*. *(G*.*) carapo* and *G*. *(G*.*) ucamara* on the basis of the following characters: long head (HL 12.3–14.1% TL vs. 10.3–12.6% in *G*. *(G*.*) bahianus*, 8.8–11.7% in *G*. *(G*.*) chaviro*, 9.7–10.7% in *G*. *(G*.*) chimarrao*, 11.1–12.8% in *G*. *(G*.*) choco*, 9.9–12.2% in *G*. *(G*.*) cuia*, 8.8–10.5% in *G*. *(G*.*) curupira*, 10.8–11.9% in *G*. *(G*.*) diamantinensis*, 8.9–12.3% in *G*. *(G*.*) eyra*, 9.6–11.9% in *G*. *(G*.*) mamiraua*, 9.9–11.3% in *G*. *(G*.*) obscurus*, 10.6–12.4% in *G*. *(G*.*) omarorum*, 7.2–11.7% in *G*. *(G*.*) pantanal*, 7.3–10.3% in *G*. *(G*.*) riberalta*, 9.1–10.3% in *G*. *(G*.*) varzea*). *Gymnotus (Gymnotus) sylvius* can be differentiated from *G*. *(G*.*) arapaima*, *G*. *(G*.*) carapo* and *G*. *(G*.*) ucamara* on the basis of the following characters: color pattern with dark band pairs equal in width to pale interbands (vs. dark band pairs 1–3X width of pale interbands in *G*. *(G*.*) arapaima*, *G*. *(G*.*) carapo* and dark band pairs >5X width of interbands in *G*. *(G*.*) ucamara*).

### Description

Sexually monomorphic. Size up to 291 mm TL with adult body proportions attained at about 150 mm TL. Adult body shape subcylindrical with a mean ratio of body width to depth of 47.5%. Body profile slender, body depth 33.6–70.2% total length. Head length long, 10.7–14.1% total length. Snout length moderate, 30.2–36.1% head length. Mouth width narrow, 30.2–42.7% head length. Preanal distance long, 60.3–68.1%head length. Anal-fin long, 76.0–85.7% total length. Cycloid or ovoid scales present on entire post-cranial portion of body from nape to caudal appendage.

Scales above lateral line intermediate, in 7 rows. Scales over anal-fin pterygiophores large, with 6–9 rows. Gape large, extending to or beyond posterior nares. Mouth position superior, lower jaw longer than upper, rictus decurved. Chin round in lateral, dorsal profiles, fleshy and bulbous with mental electroreceptive organ overlying lower jaw. Anterior narial pore partially or entirely included within gape, in small narial fold. Anterior nares small, its diameter less than that of eye. Eye below horizontal with mouth. Circumorbital series ovoid. Premaxilla with >11 teeth disposed in two rows along outer margin, median margin curved. Maxilla-palatine articulation near tip of endopterygoid. Maxilla rod- or paddle-shaped, narrow distally with a straight ventral margin, length equal to 4–6 dentary teeth. Dentary with one row of >16 teeth, 4–7 arrowhead shaped anteriorly, all others conical posteriorly. Posterodorsal and posteroventral dentary processes abuts ventral. Dentary posteroventral process shorter than or almost as long as posterodorsal, narrow distally. Dentary ventral margin lamella large, depth greater than posterior process. Dentary anteroventral margin without a hook. Anguloarticular process long, extending beyond ventral margin of dentary. Retroarticular with an arched lamella posteriorly forming a small canal, posterior margin squared. Endopterygoid superior and inferior portions approximately equal in size, ascending process robust, curved, tip simple. Interopercle dorsal margin ascending process absent. Dorsal region of hyomandibula with four lateral foramenae, supraorbital and infraorbital nerves divided. Preopercle anteroventral notch present, posterodorsal laterosensory ramus with 2 superficial pores, margin of medial shelf smooth, median shelf large, greater than half width of symplectic. Opercle dorsal margin straight, posterior margin smooth. Cranial fontanels closed in juveniles and adults. Frontal anterior margin straight, postorbital process broad, greater than two times width of supraorbital canal. Lateral ethmoid unossified. Parietal rectangular, length equal to width. Pterophenoid anteroventral process robust, extends to lateral process. Parasphenoid posterior processes robust. Prootic foramen Vp separate from V2-3+VII. Adductor mandibula insertion undivided, intermusculars absent. All basibranchials unossified. Gill rakers not contacting gill bar.

Cleithrum narrow with straight ventral margin, anterior limb long, greater than 1.8 times ascending limb, with facet for insertion of muscle from supracleithrum. Postcleithrum thin, discoid or sickle shaped. Rib 5 robust along its entire extent, less than three times width of rib 6. Displaced hemal spines absent. Pectoral fin large, with 15–16 rays. Anal fin long, with 165–189 rays. Lateral-line complete, with 19–24 ventral rami. Lateral-line dorsal rami absent in adults. Single hypaxial electric organ, extending along entire ventral margin of body with 3–4 rows of electroplates near caudal insertion of anal fin.

## *Gymnotus* (*Gymnotus*) *ucamara* Crampton, Lovejoy & Albert

### Materials examined in morphological analyses

UF 126182 (Holotype), 156 mm, Peru, Loreto, Rio Ucayali, Rio Pacaya, Cocha Zapote, in Pacaya-Samiria National Reserve (05°20.03’S, 074°29.08’W); UF 126121 (Paratype), 74 mm, same locality as UF 126182; UF 126183 (Paratype), 172 mm, same locality as UF 126182; UF 126184 (Paratypes) (2), 146–190 mm, same locality as UF 126182; MUSM 9274, Peru, Loreto, Contamana, Aguas Calientes (~07°02’S, 74°14’W); MUSM 10184 (7), 117–156 mm (2 c&s), Peru Loreto, Contamana, Rio Ucayali (~07°02’S, 074°14’W).

### Diagnosis

*Gymnotus (Gymnotus) ucamara* can be differentiated from all other members of the subgenus except *G*. *(G*.*) arapaima*, *G*. *(G*.*) carapo* and *G*. *(G*.*) sylvius* on the basis of the following characters: long head (HL 11.7–12.7% TL vs. 10.3–12.6% in *G*. *(G*.*) bahianus*, 8.8–11.7% in *G*. *(G*.*) chaviro*, 9.7–10.7% in *G*. *(G*.*) chimarrao*, 11.1–12.8% in *G*. *(G*.*) choco*, 9.9–12.2% in *G*. *(G*.*) cuia*, 8.8–10.5% in *G*. *(G*.*) curupira*, 10.8–11.9% in *G*. *(G*.*) diamantinensis*, 8.9–12.3% in *G*. *(G*.*) eyra*, 9.6–11.9% in *G*. *(G*.*) mamiraua*, 9.9–11.3% in *G*. *(G*.*) obscurus*, 10.6–12.4% in *G*. *(G*.*) omarorum*, 7.2–11.7% in *G*. *(G*.*) pantanal*, 7.3–10.3% in *G*. *(G*.*) riberalta*, 9.1–10.3% in *G*. *(G*.*) varzea*). *Gymnotus (Gymnotus) ucamara* can be differentiated from *G*. *(G*.*) arapaima*, *G*. *(G*.*) carapo* and *G*. *(G*.*) sylvius* on the basis of the following characters: color pattern with wide (>5X width of interbands) dark bands, sharp band margins and pale bands extending to dorsal mid-line (vs. dark band pairs 1–3X width of pale interbands in *G*. *(G*.*) arapaima*, *G*. *(G*.*) carapo* and *G*. *(G*.*) sylvius*).

### Description

Sexually monomorphic. Size up to 188 mm TL with adult body proportions attained at about 150 mm TL. Adult body shape subcylindrical with a mean ratio of body width to depth of 66.0%. Body profile slender, body depth 78.0–90.0% total length. Head length intermediate, 11.7–12.7% total length. Snout length moderate, 34.1–38.6% head length. Mouth width narrow, 37.0–46.4% head length. Preanal distance long, 60.0–70.5%head length. Anal-fin long, 81.4–82.4% total length. Cycloid or ovoid scales present on entire post-cranial portion of body from nape to caudal appendage.

Scales above lateral line intermediate, in 7 rows. Gape large, extending to or beyond posterior nares. Mouth position superior, lower jaw longer than upper, rictus decurved. Chin round in lateral, dorsal profiles, fleshy and bulbous with mental electroreceptive organ overlying lower jaw. Anterior narial pore partially or entirely included within gape, in small narial fold. Anterior nares small, its diameter less than that of eye. Eye below horizontal with mouth. Circumorbital series ovoid. Premaxilla with >11 teeth disposed in two rows along outer margin, median margin curved. Maxilla-palatine articulation near tip of endopterygoid. Maxilla rod- or paddle-shaped, narrow distally with a straight ventral margin, length equal to 4–6 dentary teeth. Dentary with one row of >16 teeth, 4–7 arrowhead shaped anteriorly, all others conical posteriorly. Posterodorsal and posteroventral dentary processes abuts ventral. Dentary posteroventral process shorter than or almost as long as posterodorsal, narrow distally. Dentary ventral margin lamella small, depth less than posterior process. Dentary anteroventral margin without a hook. Anguloarticular process short, extending to ventral margin of dentary. Retroarticular with an arched lamella posteriorly forming a small canal, posterior margin squared. Endopterygoid superior and inferior portions approximately equal in size, ascending process robust, curved, tip simple. Interopercle dorsal margin ascending process present. Dorsal region of hyomandibula with four lateral foramenae, supraorbital and infraorbital nerves divided. Preopercle anteroventral notch present, posterodorsal laterosensory ramus with 2 superficial pores, margin of medial shelf smooth, median shelf large, greater than half width of symplectic. Opercle dorsal margin straight, posterior margin smooth. Cranial fontanels closed in juveniles and adults. Frontal anterior margin straight, postorbital process broad, greater than two times width of supraorbital canal. Lateral ethmoid unossified. Parietal rectangular, length equal to width. Pterophenoid anteroventral process robust, extends to lateral process. Parasphenoid posterior processes gracile. Prootic foramen Vp separate from V2-3+VII. Adductor mandibula insertion undivided, intermusculars absent. All basibranchials unossified. Gill rakers not contacting gill bar.

Cleithrum broad with curved ventral margin, anterior limb long, greater than 1.8 times ascending limb, with facet for insertion of muscle from supracleithrum. Postcleithrum thin, discoid or sickle shaped. Body cavity short, with 33 precaudal vertebrae. Rib 5 robust along its entire extent, less than three times width of rib 6. Displaced hemal spines absent. Pectoral fin intermediate, with 15 rays. Anal fin long, with 215 rays. Lateral-line complete. Lateral-line dorsal rami absent in adults. Single hypaxial electric organ, extending along entire ventral margin of body with 3 rows of electroplates near caudal insertion of anal fin.

## *Gymnotus* (*Gymnotus*) *varzea* Crampton, Thorsen & Albert

### Materials examined in morphological analyses

MZUSP 60601 (Holotype), 173 mm, Brazil, Amazonas, Municipality of Álvares, Mamirauá Reserve, Ressaca da Vila Alencar (03°07’42”S, 064°48’02’W); BMNH 1998.3.12.7 (Paratype), 176 mm, Brazil, Amazonas, Municipality of Álvares, Mamirauá Reserve, Lago Araçazinho (02°59’16”S, 064°51’28’W); BMNH 1998.3.12.11 (Paratype), 160 mm, Brazil, Amazonas, Municipality of Álvares, Mamirauá Reserve (03°02’58”S, 064°51’31”W); BMNH 1998.3.12.15–18 (Paratypes) (4), 126–151 mm (1 c&s), same locality as MZUSP 60601; IDSM 433 (Paratype), 158 mm, same locality as BMNH 1998.3.12.15–18; INPA 18422 (Paratype), 66 mm, Brazil, Amazonas, Municipality of Álvares, Mamirauá Reserve, Lago Geraldo (03°06’57”S, 064°49’10”W); INPA 18423 (Paratype), 152 mm, same locality as MZUSP 60601; INPA 18424 (Paratype), 89 mm, Brazil, Amazonas, Municipality of Álvares, Mamirauá Reserve (03°06’44”S, 064°48’01’W); MZUSP 75158 (Paratype), 237 mm, Brazil, Amazonas, Municipality of Álvares, Mamirauá Reserve, Cano do Lago Sapucaia (03°04’07’S, 064°48.53’32”W); MZUSP 75159 (Paratype), 149 mm, same locality as INPA 18422; MZUSP 75160 (Paratype), 110 mm, same locality as MZUSP 75158; MZUSP 75161 (Paratype), 142 mm, same locality as MZUSP 75158; MZUSP 75163 (Paratype), 94 mm, Brazil, Amazonas, Municipality of Álvares, Mamirauá Reserve, Lago Mamirauá (03°02’18”S, 064°51’58”W); MZUSP 75164 (Paratype), 190 mm, same locality as MZUSP 75163; MZUSP 60602 (Paratypes) (8), 103–201 mm, same locality as MZUSP 60601; MZUSP 60603 (Paratypes) (6), 145–186 mm, same locality as MZUSP 60601; MZUSP 75162 (Paratype), 170 mm, same locality as MZUSP 60601; UF 118834 (Paratypes) (2), 135–140 mm, same locality as MZUSP 60601; UF 118835 (Paratypes) (2), 168–185 mm; same locality as BMNH 1998.3.12.7; UF 118838 (Paratype), 116 mm, Brazil, Amazonas, Municipality of Álvares, Mamirauá Reserve, Lago Arauaé (03°02’52”S, 064°50’05”W); UF 118839 (Paratype), 122 mm, same locality as MZUSP 75158; UF 116553 (3), 96–108 mm, Peru, Loreto, Maynas, Rio Amazon; UF 133582 (4), 111–217 mm, Peru, Loreto, Reserva Nacional Pacaya Samiria, Rio Pacaya, Cano Yarina near confluence of Rio Maranon and Rio Ucayali (05°18’07”S, 074°30’36”W); UF 133583, 171 mm, Peru, Loreto, Rio Pacaya, Cocha Sapote (05°20’15”S, 074°29’40”W); UF 146953, 285 mm, Peru, Loreto, Rio Pacaya, Cano Yarina (05°20’46”S, 074°30’12”W); UMMZ 224607, 142 mm, Peru, Loreto, Rio Momon, Maynas, Bora village near Amazon camp (~03°42’S, 073°16’W).

### Diagnosis

*Gymnotus (Gymnotus) varzea* can be differentiated from all other members of the subgenus except *G*. *(G*.*) chaviro*, *G*. *(G*.*) mamiraua*, *G*. *(G*.*) eyra* and *G*. *(G*.*) obscurus* on the basis of the following characters: anteriormost 2–4 teeth on either side of dentary anteroposteriorly compressed, resembling arrowheads (vs. 4–8 anteriormost teeth anteroposteriorly compressed in all other *Gymnotus*). *Gymnotus (Gymnotus) varzea* can be differentiated from *G*. *(G*.*) chaviro*, *G*. *(G*.*) mamiraua*, *G*. *(G*.*) eyra* and *G*. *(G*.*) obscurus* on the basis of the following characters: 14–22 dark band pairs <3X width of interbands, all with parallel margins (vs. 2–3 of the posteriormost 5 pale interbands with both margins crescent-shaped, bending outward in *G*. *(G*.*) chaviro*, dark band pairs >5X width of interbands in *G*. *(G*.*) mamiraua*, *G*. *(G*.*) eyra* and *G*. *(G*.*) obscurus*).

### Description

Sexually monomorphic. Size up to 237 mm TL with adult body proportions attained at about 150 mm TL. Adult body shape subcylindrical with a mean ratio of body width to depth of 61.0%. Body profile slender, body depth 87.8–109.2% total length. Head length intermediate, 9.0–10.4% total length. Snout length moderate, 29.7–35.8% head length. Mouth width narrow, 35.0–50.3% head length. Preanal distance long, 68.3–100.1%head length. Anal-fin long, 75.8–84.2% total length. Cycloid or ovoid scales present on entire post-cranial portion of body from nape to caudal appendage.

Scales above lateral line intermediate, in 5–9 rows. Scales over anal-fin pterygiophores large, with 4–6 rows. Gape large, extending to or beyond posterior nares. Mouth position superior, lower jaw longer than upper, rictus decurved. Chin round in lateral, dorsal profiles, fleshy and bulbous with mental electroreceptive organ overlying lower jaw. Anterior narial pore partially or entirely included within gape, in small narial fold. Anterior nares small, its diameter less than that of eye. Eye below horizontal with mouth. Circumorbital series ovoid. Premaxilla with <10 teeth disposed in two rows along outer margin, median margin curved. Maxilla-palatine articulation near tip of endopterygoid. Maxilla rod- or paddle-shaped, narrow distally with a straight ventral margin, length equal to 4–6 dentary teeth. Dentary with one row of <12 teeth, 2–4 arrowhead shaped anteriorly, all others conical posteriorly. Posterodorsal and posteroventral dentary processes abuts ventral. Dentary posteroventral process shorter than or almost as long as posterodorsal, narrow distally. Dentary ventral margin lamella large, depth greater than posterior process. Dentary anteroventral margin without a hook. Anguloarticular process long, extending beyond ventral margin of dentary. Retroarticular with an arched lamella posteriorly forming a small canal, posterior margin squared. Endopterygoid superior and inferior portions approximately equal in size, ascending process robust, straight, tip simple. Interopercle dorsal margin ascending process present. Dorsal region of hyomandibula with four lateral foramenae, supraorbital and infraorbital nerves divided. Preopercle anteroventral notch present, posterodorsal laterosensory ramus with 2 superficial pores, margin of medial shelf smooth, median shelf large, greater than half width of symplectic. Opercle dorsal margin straight, posterior margin smooth. Cranial fontanels closed in juveniles and adults. Frontal anterior margin straight, postorbital process broad, greater than two times width of supraorbital canal. Lateral ethmoid unossified. Parietal rectangular, length equal to width. Pterophenoid anteroventral process robust, extends to lateral process. Parasphenoid posterior processes robust. Prootic foramen Vp separate from V2-3+VII. Adductor mandibula insertion undivided, intermusculars absent. All basibranchials unossified. Gill rakers not contacting gill bar.

Cleithrum broad with curved ventral margin, anterior limb broad, greater than 1.8 times ascending limb, with facet for insertion of muscle from supracleithrum. Postcleithrum thin, discoid or sickle shaped. Body cavity variable, with 35–40 precaudal vertebrae. Rib 5 robust along its entire extent, less than three times width of rib 6. Displaced hemal spines absent. Pectoral fin broad, with 16–19 rays. Anal fin long, with 230–310 rays. Lateral-line complete. Lateral-line dorsal rami absent in adults. Single hypaxial electric organ, extending along entire ventral margin of body with 3–4 rows of electroplates near caudal insertion of anal fin.

## *Gymnotus* (*Lamontianus*), subgen. nov. (Table 5)

### Type species

*G*. (*L*.) *anguillaris*.

### Other included species

G. (L.) n. sp. ‘ARAP’, G. (L.) n. sp. ‘ARIP’, G. (L.) cataniapo, G. (L.) pedanopterus, G. (L.) tiquie.

### Diagnosis

*Lamontianus* is readily distinguishable from all other subgenera of the Gymnotinae by the presence of a single preopercular-mandibular sensory canal pore in the dorsoposterior portion of the preopercle, in combination with the following characters: a color pattern consisting of evenly spaced, dark pigment bands or band-pairs with wavy, high-contrast (sharp) margins, long maximum body cavity (PCV 37–53, except in *G*. *pedanopterus* with 31–32) vs. 48 in *Gymnotus* (*Pantherus*) and 35–44 in *Gymnotus* (*Tigrinus*). *Gymnotus* (*Lamontianus*) is morphologically most similar to *Gymnotus* (*Tigrinus*), from which is it readily distinguishable by the following characters: more anal-fin rays more (AFR 210–312) vs. fewer (AFR 135–245), many small anal-fin pterygiophore scales (APS 9–10) vs. fewer, larger anal-fin pterygiophore scales (APS 5–7), end of maxilla rod-shaped (narrow distally) vs. paddle shaped (broad distally), superior portion of metapterygoid ossified to anterior margin of interorbital vs. less than anterior margin of interorbital; 5, intermediate number of branched anal-fin rays (AFR 10–17) vs. many branched anal-fin rays (AFR >18).

### Description

Sexually monomorphic. Size up to 337 mm TL with adult body proportions attained at about 150 mm TL. Adult body shape subcylindrical with a mean ratio of body width to depth of 62.3%. Body profile slender, body depth 59.0–106.8% total length. Head length moderate, 9.7–31.9% total length. Snout length moderate, 26.2–39.7% head length. Mouth width narrow, 27.4–58.3% head length. Preanal distance long, 50.7–188.2%head length. Anal-fin long, 65.9–85.0% total length. Cycloid or ovoid scales present on entire post-cranial portion of body from nape to caudal appendage.

Scales above lateral line intermediate, in 5–13 rows. Scales over anal-fin pterygiophores large, with 5–10 rows. Gape large, extending to or beyond posterior nares. Mouth position superior, lower jaw longer than upper, rictus decurved. Chin round in lateral, dorsal profiles, fleshy and bulbous with mental electroreceptive organ overlying lower jaw. Anterior narial pore partially or entirely included within gape, in small narial fold. Anterior nares small, its diameter less than that of eye. Eye below horizontal with mouth. Circumorbital series ovoid. Premaxilla with 11 or more teeth disposed in two rows along outer margin, median margin curved. Maxilla-palatine articulation near tip of endopterygoid. Maxilla rod- or paddle-shaped, narrow distally with a straight ventral margin, length equal to 7–9 dentary teeth. Dentary with one row of needle-shaped teeth. Posterodorsal and posteroventral dentary processes abuts ventral. Dentary posteroventral process shorter than or almost as long as posterodorsal, narrow distally. Dentary ventral margin lamella small, depth less than posterior process. Dentary anteroventral margin with a hook present in lateral view. Mandible long and extended. Anguloarticular process long, extending beyond ventral margin of dentary. Retroarticular with an arched lamella posteriorly forming a small canal, posterior margin squared. Endopterygoid superior and inferior portions approximately equal in size, ascending process robust, long, straight (except in *G*. *(L*.*) tiquie*), tip simple. Interopercle dorsal margin ascending process present. Dorsal region of hyomandibula with four lateral foramenae, supraorbital and infraorbital nerves connected. Preopercle anteroventral notch present, posterodorsal laterosensory ramus with one superficial pore, margin of medial shelf smooth (except in *G*. *(L*.*) pedanopterus*), median shelf large, greater than half width of symplectic (except in *G*. *(L*.*) pedanopterus*). Opercle dorsal margin straight or convex, posterior margin entirely smooth. Subopercle dorsal margin concave. Cranial fontanels closed in juveniles and adults. Frontal broad, anterior margin straight (except in *G*. *(L*.*) tiquie*), postorbital process narrow, less than two times width of supraorbital canal. Lateral ethmoid unossified. Parietal rectangular, length equal to width. Pterosphenoid anteroventral portion reduced, extends dorsally to lateral margin of parasphenoid. Parasphenoid posterior processes robust, with shallow, convex posterior margin. Prootic foramen Vp combined with V2-3+VII. *M*. *adductor mandibula* intermusculars absent. All basibranchials unossified. Gill rakers not contacting gill bar.

Cleithrum narrow with straight ventral margin, anterior limb long, greater than 1.8 times ascending limb, lacking large facet for insertion of muscle from supracleithrum. Postcleithrum thin, discoid or sickle shaped. Body cavity long, with 31–58 precaudal vertebrae. Rib 5 with broad medial triangular shelf, greater than three times width of rib 6. Displaced hemal spines absent. Pectoral fin intermediate, with 9–18 rays. Anal fin of moderate length, with 210–312 rays. Lateral-line reduced, with 2–18 ventral rami. Lateral-line dorsal rami absent in adults. Single hypaxial electric organ, extending along entire ventral margin of body with 3 rows of electroplates near caudal insertion of anal fin.

### Etymology

Subgenus name to honor Francesca Raymond LaMonte (1895–1982), Assistant Curator of Ichthyology at the American Museum of Natural History, for contributions to gymnotiform taxonomy and ichthyology as a whole. Among her 86 articles and several books [73], LaMonte (1935) described two gymnotid species, *Gymnotus* (*Tijax*) *cylindricus* and *Gymnotus* (*Tigrinus) coatesi*, each representing the types species of their respective subgenus.

Diagnoses and descriptions of each species of *Lamontianus* (Table 13)

**Table 13:** Summary of morphometric and meristic data for four valid species of *Lamontianus*. Data for 36 specimens.

*** ***	***G*. *(L*.*) anguillaris***				***G*. *(L*.*) cataniapo***				***G*. *(L*.*) pedanopterus***				***G*. *(L*.*) tiquie***			
** **	**N**	**Min**	**Max**	**AVG**	**N**	**Min**	**Max**	**AVG**	**N**	**Min**	**Max**	**AVG**	**N**	**Min**	**Max**	**AVG**
**TL**	6	131	289	221	9	165	316	217	15	128	337	200	6	163	240	188
**HL**	6	12.6	24.1	20.0	9	15.3	29.0	20.7	15	16.0	31.9	23.2	6	15.3	20.8	17.2
**HL%**	6	8.3	9.6	9.1	9	8.8	11.1	9.5	15	8.0	14.2	11.9	6	8.7	9.5	9.2
**PR%**	6	33.2	37.3	35.1	3	36.2	37.4	36.9	10	31.0	36.0	33.5	6	35.4	39.7	38.1
**MW%**	6	41.3	46.4	43.8	3	38.0	44.4	41.6	9	31.5	37.3	35.3	6	48.5	58.3	53.1
**PO%**	6	55.5	63.5	60.2	9	58.4	64.1	61.2	15	59.7	66.1	62.7	6	58.2	65.1	61.1
**IO%**	6	43.1	53.3	45.8	8	34.0	36.2	37.0	15	22.5	34.7	28.5	6	42.1	42.7	45.7
**BD%**	6	91.5	98.3	95.1	9	71.4	106.8	89.9	15	62.4	81.8	70.5	6	85.9	99.8	91.4
**BW%**	6	40.8	74.3	61.2	8	56.9	76.4	67.3	13	38.7	56.1	45.8	6	58.3	76.7	67.4
**BW/BD**	6	42.2	76.1	64.3	8	57.4	83.8	73.4	13	55.1	70.3	64.0	6	62.1	79.3	73.8
**HD%**	6	60.9	65.1	62.8	9	51.2	59.9	56.5	15	47.6	60.0	53.1	6	61.5	68.8	64.6
**HW%**	6	65.9	68.7	68.5	9	58.1	63.6	61.3	15	44.9	57.8	51.5	6	69.7	73.7	70.7
**PA%**	6	81.8	97.1	91.0	9	102.5	188.2	152.3	15	50.7	143.9	85.6	6	90.8	108.3	100.0
**P1%**	6	42.1	46.9	44.3	9	29.3	45.5	35.1	14	29.2	41.8	35.4	6	47.1	52.9	49.4
**AF%**	6	77.1	83.3	80.6	9	81.5	84.7	83.0	15	75.1	85.0	81.5	6	81.0	82.1	81.7
** **	**N**	**Min**	**Max**	**Mode**	**N**	**Min**	**Max**	**Mode**	**N**	**Min**	**Max**	**Mode**	**N**	**Min**	**Max**	**Mode**
**BND**	4	18	26	26	8	27	35	30	14	18	27	22	5	19	24	21
**AFR**	5	210	270	255	9	227	303	245	12	222	312	260	5	210	265	240
**P1R**	5	15	18	16	9	13	15	14	15	12	14	12	5	15	17	15
**SAL**	5	7	8	7	9	8	10	10	15	7	13	9	5	7	8	7
**CEP**	_	_	_	_	_	_	_	_	_	_	_	_	_	_	_	_
**APS**	1	9	9	9	1	6	6	6	1	8	8	8	5	8	9	8
**PCV**	4	37	38	38	4	47	51	50	7	31	32	32	1	45	45	45
**PLR**	5	51	62	58	3	60	67	65	10	45	60	54	5	69	78	76
**PLL**	3	95	130	124	1	99	99	99	1	85	85	85	5	94	108	102
**VLR**	1	16	16	16	_	_	_	_	_	_	_	_	5	2	6	4

## *Gymnotus (Lamontianus) anguillaris* Hoedeman

### Materials examined in morphological analyses

ZMA 100338a (Holotype), 228 mm, Suriname, Coropina creek; ZMA 100338b (Paratype), 233 mm, Same locality as ZMA 100338a; ZMA 105930 (4), 255–302 mm, Suriname, Marowijne, Lawa River, Maka Creek; UMMZ 190413 (3), 131–289 mm, same locality as ZMA 105930.

### Diagnosis

*Gymnotus (Lamontianus) anguillaris* can be differentiated from all other members of the subgenus on the basis of the following characters: color pattern with dark brown band pairs absent or heavily obscured above lateral line in anterior 60% of body (vs. bands visible above lateral line over entire body in all other *Lamontianus*), short body cavity except compared to *G*. *(L*.*) pedanopterus* (PCV 37–38 vs. 47–51 in *G*. *(L*.*) cataniapo*, 45 in *G*. *(L*.*) tiquie*).

### Description

Sexually monomorphic. Size up to 289 mm TL with adult body proportions attained at about 150 mm TL. Adult body shape subcylindrical with a mean ratio of body width to depth of 64.3%. Body profile slender, body depth 91.5–98.3% total length. Head length short, 8.3–9.6% total length. Snout length moderate, 33.2–37.3% head length. Mouth width narrow, 41.3–46.4% head length. Preanal distance long, 81.8–97.1%head length. Anal-fin long, 77.1–83.3% total length. Cycloid or ovoid scales present on entire post-cranial portion of body from nape to caudal appendage.

Scales above lateral line intermediate, in 7–8 rows. Scales over anal-fin pterygiophores large, with 9 rows. Gape large, extending to or beyond posterior nares. Mouth position superior, lower jaw longer than upper, rictus decurved. Chin round in lateral, dorsal profiles, fleshy and bulbous with mental electroreceptive organ overlying lower jaw. Anterior narial pore partially or entirely included within gape, in small narial fold. Anterior nares small, its diameter less than that of eye. Eye below horizontal with mouth. Circumorbital series ovoid. Premaxilla with >11 teeth disposed in two rows along outer margin, median margin curved. Maxilla-palatine articulation near tip of endopterygoid. Maxilla rod- or paddle-shaped, narrow distally with a straight ventral margin, length equal to 7–9 dentary teeth. Dentary with one row of >16 teeth, all conical. Posterodorsal and posteroventral dentary processes abuts ventral. Dentary posteroventral process shorter than or almost as long as posterodorsal, narrow distally. Dentary ventral margin lamella small, depth less than posterior process. Dentary anteroventral margin with a hook. Anguloarticular process long, extending beyond ventral margin of dentary. Retroarticular with an arched lamella posteriorly forming a small canal, posterior margin squared. Endopterygoid superior and inferior portions approximately equal in size, ascending process robust, straight, tip simple. Interopercle dorsal margin ascending process present. Dorsal region of hyomandibula with four lateral foramenae, supraorbital and infraorbital nerves connected. Preopercle anteroventral notch present, posterodorsal laterosensory ramus with one superficial pore, margin of medial shelf smooth, median shelf large, greater than half width of symplectic. Opercle dorsal margin straight, posterior margin smooth. Cranial fontanels closed in juveniles and adults. Frontal anterior margin straight, postorbital process narrow, less than two times width of supraorbital canal. Lateral ethmoid unossified. Parietal rectangular, length equal to width. Pterophenoid anteroventral process reduced, extends to lateral process. Parasphenoid posterior processes robust. Prootic foramen Vp combined with V2-3+VII. Adductor mandibula insertion undivided, intermusculars absent. All basibranchials unossified. Gill rakers not contacting gill bar.

Cleithrum narrow with straight ventral margin, anterior limb long, greater than 1.8 times ascending limb, with facet for insertion of muscle from supracleithrum. Postcleithrum thin, discoid or sickle shaped. Body cavity long, with 37–38 precaudal vertebrae. Rib 5 broad along its entire extent, greater than three times width of rib 6. Displaced hemal spines absent. Pectoral fin broad, with 15–18 rays. Anal fin long, with 210–270 rays. Lateral-line complete, with 16 ventral rami. Lateral-line dorsal rami absent in adults. Single hypaxial electric organ, extending along entire ventral margin of body.

## *Gymnotus (Lamontianus) cataniapo* Mago-Leccia

### Materials examined in morphological analyses

MBUCV-V 14736 (Holotype), Venezuela, Amazonas, lagoon NE of airport of San Carlos de Rio Negro (01°55’N, 67°02’W); AMNH 58650 (Paratype), 184 mm, same locality as MBUCV-V 14736; AMNH 58668 (Paratype), 184 mm, same locality as MBUCV-V 14736; MBUCV-V 14181a, 179 mm; MBUCV-V 14181b, 316 mm; MBUCV 14300, 213 mm.

### Diagnosis

*Gymnotus (Lamontianus) cataniapo* can be differentiated from all other members of the subgenus on the basis of the following combination of characters: color pattern with many dark band pairs (BND 27–35 vs. 18–26 in *G*. *(L*.*) anguillaris*, 18–27 in *G*. *(L*.*) pedanopterus*, 19–24 in *G*. *(L*.*) tiquie*), especially long body cavity (PCV 47–51 vs. 37–38 in *G*. *(L*.*) anguillaris*, 31–32 in *G*. *(L*.*) pedanopterus*, 45 in *G*. *(L*.*) tiquie*), short interorbital distance except compared to *G*. *(L*.*) pedanopterus* (IO 34.0–36.2% HL vs. 43.1–53.3% in *G*. *(L*.*) anguillaris*, 42.1–47.2% in *G*. *(L*.*) tiquie*), slender head except compared to *G*. *(L*.*) pedanopterus* (HD 51.2–59.9% HL vs. 60.9–65.1% in *G*. *(L*.*) anguillaris*, 61.5–68.8% in *G*. *(L*.*) tiquie*), narrow head except compared to *G*. *(L*.*) pedanopterus* (HW 58.1–63.6% HL vs. 65.9–68.7% in *G*. *(L*.*) anguillaris*, 69.7–73.7% in *G*. *(L*.*) tiquie*).

### Description

Sexually monomorphic. Size up to 316 mm TL with adult body proportions attained at about 150 mm TL. Adult body shape subcylindrical with a mean ratio of body width to depth of 73.4%. Body profile slender, body depth 71.4–106.8% total length. Head length short, 8.8–11.1% total length. Snout length moderate, 36.2–37.4% head length. Mouth width narrow, 38.0–44.4% head length. Preanal distance long, 102.5–188.2%head length. Anal-fin long, 81.5–84.7% total length. Cycloid or ovoid scales present on entire post-cranial portion of body from nape to caudal appendage.

Scales above lateral line intermediate, in 8–10 rows. Scales over anal-fin pterygiophores large, with 6 rows. Gape large, extending to or beyond posterior nares. Mouth position superior, lower jaw longer than upper, rictus decurved. Chin round in lateral, dorsal profiles, fleshy and bulbous with mental electroreceptive organ overlying lower jaw. Anterior narial pore partially or entirely included within gape, in small narial fold. Anterior nares small, its diameter less than that of eye. Eye below horizontal with mouth. Circumorbital series ovoid. Premaxilla with >11 teeth disposed in two rows along outer margin, median margin curved. Maxilla-palatine articulation near tip of endopterygoid. Maxilla rod-shaped, narrow distally, length equal to 7–9 dentary teeth. Dentary with one row of >16 teeth, all conical. Posterodorsal and posteroventral dentary processes abuts ventral. Dentary posteroventral process shorter than or almost as long as posterodorsal, narrow distally. Dentary ventral margin lamella small, depth less than posterior process. Dentary anteroventral margin with a hook. Anguloarticular process long, extending beyond ventral margin of dentary. Retroarticular with an arched lamella posteriorly forming a small canal, posterior margin squared. Endopterygoid superior and inferior portions approximately equal in size, ascending process robust, straight, tip simple. Interopercle dorsal margin ascending process absent. Dorsal region of hyomandibula with four lateral foramenae, supraorbital and infraorbital nerves connected. Preopercle anteroventral notch present, posterodorsal laterosensory ramus with one superficial pore, margin of medial shelf smooth, median shelf large, greater than half width of symplectic. Opercle dorsal margin straight, posterior margin smooth. Cranial fontanels closed in juveniles and adults. Frontal anterior margin straight, postorbital process narrow, less than two times width of supraorbital canal. Lateral ethmoid unossified. Parietal rectangular, length equal to width. Pterophenoid anteroventral process reduced, extends to lateral process. Parasphenoid posterior processes robust. Prootic foramen Vp connected to V2-3+VII. Adductor mandibula insertion undivided, intermusculars absent. All basibranchials unossified. Gill rakers not contacting gill bar.

Cleithrum narrow with straight ventral margin, anterior limb long, greater than 1.8 times ascending limb, with facet for insertion of muscle from supracleithrum. Postcleithrum thin, discoid or sickle shaped. Body cavity long, with 47–51 precaudal vertebrae. Rib 5 broad along its entire extent, greater than three times width of rib 6. Displaced hemal spines absent. Pectoral fin narrow, with 13–15 rays. Anal fin long, with 227–303 rays. Lateral-line complete. Lateral-line dorsal rami absent in adults. Single hypaxial electric organ, extending along entire ventral margin of body.

## *Gymnotus (Lamontianus) pedanopterus* Mago-Leccia

### Materials examined in morphological analyses

MBUCV-V 14738 (Holotype), 215 mm, Venezuela, Amazonas, where Caño Temblador crosses road from San Carlos de Rio Negro to Solano (01°58’N, 067°00’W); AMNH 58651 (Paratype), 153mm, Venezuela, Amazonas, Río Orinoco, Río Casiquiare, near mouth of Río Pamoni (02°48’N, 65°58’W); ANSP 141596 (14), 76–340 mm, Venezuela, Amazonas, Bolivar, Jabillal (06°57’N, 64°50’W).

### Diagnosis

*Gymnotus (Lamontianus) pedanopterus* can be differentiated from all other members of the subgenus on the basis of the following characters: Narrow mouth (MW 31.5–37.3% HL vs. 41.3–46.4% in *G*. *(L*.*) anguillaris*, 38.0–44.4% in *G*. *(L*.*) cataniapo*, 50.0–58.8% in *G*. *(L*.*) tiquie*), short interorbital distance (IO 22.5–33.7% HL vs. 43.1–53.3% in *G*. *(L*.*) anguillaris*, 33.2–41.4% in *G*. *(L*.*) cataniapo*, 42.1–51.3% in *G*. *(L*.*) tiquie*), short body cavity (PCV 31–23 vs. 37–38 in *G*. *(L*.*) anguillaris*, 47–51 in *G*. *(L*.*) cataniapo*, 45 in *G*. *(L*.*) tiquie*), preopercle median shelf small (<half width of symplectic vs. >half width of symplectic in all other *Lamontianus*).

### Description

Sexually monomorphic. Size up to 337 mm TL with adult body proportions attained at about 150 mm TL. Adult body shape subcylindrical with a mean ratio of body width to depth of 64.0%. Body profile slender, body depth 38.7–56.1% total length. Head length variable, 8.0–14.2% total length. Snout length moderate, 31.0–26.0% head length. Mouth width narrow, 31.5–37.3% head length. Preanal distance long, 50.7–143.9% head length. Anal-fin long, 75.1–85.0% total length. Cycloid or ovoid scales present on entire post-cranial portion of body from nape to caudal appendage.

Scales above lateral line intermediate, in 7–13 rows. Scales over anal-fin pterygiophores large, with 8 rows. Gape large, extending to or beyond posterior nares. Mouth position superior, lower jaw longer than upper, rictus decurved. Chin round in lateral, dorsal profiles, fleshy and bulbous with mental electroreceptive organ overlying lower jaw. Anterior narial pore partially or entirely included within gape, in small narial fold. Anterior nares small, its diameter less than that of eye. Eye below horizontal with mouth. Circumorbital series ovoid. Premaxilla with >11 teeth disposed in two rows along outer margin, median margin curved. Maxilla-palatine articulation near tip of endopterygoid. Maxilla rod- or paddle-shaped, narrow distally with a straight ventral margin, length equal to 7–9 dentary teeth. Dentary with one row of >16 teeth, all conical. Posterodorsal and posteroventral dentary processes abuts ventral. Dentary posteroventral process shorter than or almost as long as posterodorsal, narrow distally. Dentary ventral margin lamella small, depth less than posterior process. Dentary anteroventral margin with a hook. Anguloarticular process long, extending beyond ventral margin of dentary. Retroarticular with an arched lamella posteriorly forming a small canal, posterior margin squared. Endopterygoid superior and inferior portions approximately equal in size, ascending process robust, straight, tip simple. Interopercle dorsal margin ascending process present. Dorsal region of hyomandibula with four lateral foramenae, supraorbital and infraorbital nerves connected. Preopercle anteroventral notch present, posterodorsal laterosensory ramus with one superficial pore, margin of medial shelf serrate, median shelf small, less than half width of symplectic. Opercle dorsal margin straight, posterior margin smooth. Cranial fontanels closed in juveniles and adults. Frontal anterior margin straight, postorbital process narrow, less than two times width of supraorbital canal. Lateral ethmoid unossified. Parietal rectangular, length equal to width. Pterophenoid anteroventral process reduced, extends to lateral process. Parasphenoid posterior processes robust. Prootic foramen Vp combined with V2-3+VII. Adductor mandibula insertion undivided, intermusculars absent. All basibranchials unossified. Gill rakers not contacting gill bar.

Cleithrum narrow with straight ventral margin, anterior limb long, greater than 1.8 times ascending limb, without facet for insertion of muscle from supracleithrum. Postcleithrum thin, discoid or sickle shaped. Body cavity intermediate, with 31–32 precaudal vertebrae. Rib 5 broad along its entire extent, greater than three times width of rib 6. Displaced hemal spines absent. Pectoral fin narrow, with 12–14 rays. Anal fin long, with 222–312 rays. Lateral-line complete. Lateral-line dorsal rami absent in adults. Single hypaxial electric organ, extending along entire ventral margin of body.

## *Gymnotus (Lamontianus) tiquie* Maxime, Lima & Albert

### Materials examined in morphological analyses

MZUSP 104507 (Holotype), 177 mm, Brazil, Amazonas, Rio Tiquié, comunidade de São José, Igarapé Espuma (00°13’00”N, 69°36’00”W); MZUSP 93050 (Paratypes) (3), 69–177 mm, Brazil, Amazonas; ANSP 188903 (Paratype), 175 mm, same locality as MZUSP 104507; MZUSP 64310 (Paratype), 76 mm, Brazil, Amazonas, Igarapé Yoariwasotoamakúya, trib. Rio Tiquié, Cachoeira Comprida village (00°15’44”N, 70°01’05"W); MZUSP 85002 (Paratypes) (2), 195–240 mm, Brazil, Amazonas, creek at old São Pedro village (00°16’04.4”N, 69°58’21.5”W); MZUSP 81335 (Paratype), 104 mm, Brazil, Amazonas, Rio Tiquié, São Pedro village, between Cachoeira do Caruru and Cachoeira da Abelha (00°16’N, 69°58’W); MZUSP 81492, 201 mm, Rio Tiquié São Pedro village, Igarapé Mipiriyapotemakãya, tributary Igarapé Açaí (00°15’55.4”N, 69°58’16.4”W).

### Diagnosis

*Gymnotus (Lamontianus) tiquie* can be differentiated from all other members of the subgenus on the basis of the following characters: wide mouth (MW 50.0–58.8% HL vs. 41.3–46.4% in *G*. *(L*.*) anguillaris*, 38.0–44.4% in *G*. *(L*.*) cataniapo*, 31.5–37.3% in *G*. *(L*.*) pedanopterus*), broad interorbital distance except compared to *G*. *(L*.*) anguillaris* (IO 42.1–51.3% HL vs. 28.1–41.4% in *G*. *(L*.*) cataniapo*, 24.0–34.7% in *G*. *(L*.*) pedanopterus*), mesethmoid neck narrow (<2X length of lateral process, vs. >2X length of lateral process in all other *Lamontianus*), maxilla sickle-shaped with concave dorsal margin (vs. rod- or paddle-shaped with straight dorsal margin in all other *Lamontianus*), maxilla short (width of 4–6 dentary teeth vs. broad, width of 7–9 dentary teeth in all other *Lamontianus*), end of maxilla paddle-shaped, broad distally (vs. rod-shaped, narrow distally in all other *Lamontianus*), dentary with >5 slender, needle-shaped teeth (vs. all dentary teeth short, conical in all other *Lamontianus*).

### Description

Sexually monomorphic. Size up to 240 mm TL with adult body proportions attained at about 150 mm TL. Adult body shape subcylindrical with a mean ratio of body width to depth of 53.1%. Body profile slender, body depth 85.9–95.8% total length. Head length short, 8.7–9.5% total length. Snout length moderate, 35.4–39.7% head length. Mouth width narrow, 48.5–58.3% head length. Preanal distance long, 90.8–108.3% head length. Anal-fin long, 81.0–82.0% total length. Cycloid or ovoid scales present on entire post-cranial portion of body from nape to caudal appendage.

Scales above lateral line intermediate, in 7–8 rows. Scales over anal-fin pterygiophores large, with 8–9 rows. Gape large, extending to or beyond posterior nares. Mouth position superior, lower jaw longer than upper, rictus decurved. Chin round in lateral, dorsal profiles, fleshy and bulbous with mental electroreceptive organ overlying lower jaw. Anterior narial pore partially or entirely included within gape, in small narial fold. Anterior nares small, its diameter less than that of eye. Eye below horizontal with mouth. Circumorbital series ovoid. Premaxilla with >11 teeth disposed in two rows along outer margin, median margin curved. Maxilla-palatine articulation near tip of endopterygoid. Maxilla rod- or paddle-shaped, broad distally, length equal to >10 dentary teeth. Dentary with one row of >16 teeth, >5 needle-shaped anteriorly, all others conical posteriorly. Posterodorsal and posteroventral dentary processes abuts ventral. Dentary posteroventral process shorter than or almost as long as posterodorsal, narrow distally. Dentary ventral margin lamella small, depth less than posterior process. Dentary anteroventral margin with a hook. Anguloarticular process long, extending beyond ventral margin of dentary. Retroarticular with an arched lamella posteriorly forming a small canal, posterior margin squared. Endopterygoid superior and inferior portions approximately equal in size, ascending process long, curved, tip simple. Interopercle dorsal margin ascending process present. Dorsal region of hyomandibula with four lateral foramenae, supraorbital and infraorbital nerves connected. Preopercle anteroventral notch present, posterodorsal laterosensory ramus with one superficial pore, margin of medial shelf smooth, median shelf large, greater than half width of symplectic. Opercle dorsal margin straight, posterior margin smooth. Cranial fontanels closed in juveniles and adults. Frontal anterior margin rounded, postorbital process narrow, less than two times width of supraorbital canal. Lateral ethmoid unossified. Parietal rectangular, length equal to width. Pterophenoid anteroventral process reduced, extends to lateral process. Parasphenoid posterior processes robust. Prootic foramen Vp combined with V2-3+VII. Adductor mandibula insertion undivided, intermusculars absent. All basibranchials unossified. Gill rakers not contacting gill bar.

Cleithrum narrow with straight ventral margin, anterior limb long, greater than 1.8 times ascending limb, without facet for insertion of muscle from supracleithrum. Postcleithrum thin, discoid or sickle shaped. Body cavity long, with 45 precaudal vertebrae. Rib 5 broad along its entire extent, greater than three times width of rib 6. Displaced hemal spines absent. Pectoral fin broad, with 15–17 rays. Anal fin long, with 210–265 rays. Lateral-line complete. Lateral-line dorsal rami absent in adults. Single hypaxial electric organ, extending along entire ventral margin of body.

## *Gymnotus* (*Pantherus*) subgen. nov. (Table 5)

### Type species

*G*. (*P*.) *pantherinus*.

### Other included species

*G*. (*P*.) *capitimaculatus*, *G*. (*P*.) *refugio*.

### Diagnosis

*Gymnotus* (*Pantherus*) is readily distinguishable from all other subgenera of the Gymnotinae by the following characters: a color pattern consisting of irregular dark color blotches about 3–4 scales in diameter, with blurry, low-contrast margins covering entire body vs. band pairs in all other subgenera except *Gymnotus* (*Tijax*), which possesses small, irregular dark pigment spots 2–3 scales in diameter.

### Description

Sexually monomorphic. Size up to 234 mm TL with adult body proportions attained at about 150 mm TL. Adult body shape subcylindrical with a mean ratio of body width to depth of 47.6%. Body profile slender, body depth 69.9–99.6% total length. Head length moderate, 8.6–13.1% total length. Snout length moderate, 24.4–37.6% head length. Mouth width narrow, 31.5–51.2% head length. Preanal distance long, 80.7–123.5% head length. Anal-fin long, 64.8–81.7% total length. Cycloid or ovoid scales present on entire post-cranial portion of body from nape to caudal appendage. Scales above lateral line intermediate, in 5–9 rows. Scales over anal-fin pterygiophores large, with 4–11 rows. Gape large, extending to or beyond posterior nares. Mouth position superior, lower jaw longer than upper, rictus decurved. Chin round in lateral, dorsal profiles, fleshy and bulbous with mental electroreceptive organ overlying lower jaw. Anterior narial pore partially or entirely included within gape, in small narial fold. Anterior nares small, its diameter less than that of eye. Eye below horizontal with mouth. Circumorbital series ovoid. Premaxilla with more than 11 teeth disposed in two rows along outer margin, median margin curved. Maxilla-palatine articulation near tip of endopterygoid. Maxilla rod- or paddle-shaped, narrow distally with a straight ventral margin, length equal to 7–9 dentary teeth. Dentary with one row of 12–15 teeth, all conical. Posterodorsal and posteroventral dentary processes abuts ventral. Dentary posteroventral process shorter than or almost as long as posterodorsal, narrow distally. Dentary ventral margin lamella small, depth less than posterior process. Dentary anteroventral margin hook present in lateral view. Mandible long and extended. Anguloarticular process long, extending beyond ventral margin of dentary. Retroarticular with an arched lamella posteriorly forming a small canal, posterior margin squared. Endopterygoid superior and inferior portions approximately equal in size, ascending process robust, long, straight, tip simple. Interopercle dorsal margin ascending process present. Dorsal region of hyomandibula with four lateral foramenae, supraorbital and infraorbital nerves connected. Preopercle anteroventral notch present, posterodorsal laterosensory ramus with one superficial pore, margin of medial shelf entirely smooth, median shelf large, greater than half width of symplectic. Opercle dorsal margin straight or convex, posterior margin entirely smooth. Subopercle dorsal margin concave. Cranial fontanels closed in juveniles and adults. Frontal broad, anterior margin straight, postorbital process narrow, less than two times width of supraorbital canal. Lateral ethmoid unossified. Parietal rectangular, length equal to width. Pterosphenoid anteroventral portion reduced, extends dorsally to lateral margin of parasphenoid. Parasphenoid posterior processes robust with a shallow posterior margin. Prootic foramen Vp combined with V2-3+VII. *M*. *adductor mandibula* intermusculars absent. All basibranchials unossified. Gill rakers not contacting gill bar.

Cleithrum narrow with straight ventral margin, anterior limb long, greater than 1.8 times ascending limb, lacking large facet for insertion of muscle from supracleithrum. Postcleithrum thin, discoid or sickle shaped. Body cavity log, with 48 precaudal vertebrae. Rib 5 with broad medial triangular shelf, greater than three times width of rib 6. Displaced hemal spines absent. Pectoral fin large, with 14–19 rays. Anal fin of intermediate length, with 173–232 rays. Lateral-line complete, with 5–29 ventral rami. Lateral-line dorsal rami absent in adults. Single hypaxial electric organ, extending along entire ventral margin of body with 3 rows of electroplates near caudal insertion of anal fin.

### Etymology

Subgenus name derived from the jaguar, *Panthera onca*, following the type species, *G*. *(P*.*) pantherinus* (Steindachner).

**Diagnoses and descriptions of each species of *Pantherus* (Table 14)**

## *Gymnotus (Pantherus) capitimaculatus* Rangel-Pereira

### Materials examined in morphological analyses

UFRJ 9785 (Holotype), 131 mm, Brazil, Bahia, Itamaraju, Rio do Ouro, road crossing perpendicular to BR101, ~7 km north of Itamaraju (16°57’04”S, 39°33’21”W); UFRJ 9625 (Paratypes) (7), 121–158 mm, same locality as UFRJ 9785; UFRJ 9728 (Paratypes) (2 c&s) 101–107 mm, same locality as UFRJ 9785; UFRJ 9964 (2), 116–156 mm, same locality as UFRJ 9785.

**Table 14:** Summary of morphometric and meristic data for three species of *Pantherus*. Data for a total of 35 specimens.

*** ***	***G*. *(P*.*) capitimaculatus***				***G*. *(P*.*) pantherinus***				***G*. *(P*.*) refugio***			
** **	**N**	**Min**	**Max**	**AVG**	**N**	**Min**	**Max**	**AVG**	**N**	**Min**	**Max**	**AVG**
**TL**	10	101	158	_	9	77	189	136	26	69	234	156
**HL**	_	_	_	_	9	8.5	15.1	12.5	25	9.1	20.8	15.0
**HL%**	10	8.8	9.9	9.3	9	7.9	11.1	9.3	25	8.6	13.1	9.8
**PR%**	10	17.7	20.7	19.6	8	27.9	35.3	32.2	25	24.4	37.6	32.6
**MW%**	_	_	_	_	9	30.1	48.6	37.8	25	31.5	51.2	40.8
**PO%**	10	60.2	64.6	61.8	9	60.4	68.7	64.3	25	54.8	67.6	62.3
**IO%**	10	39.8	43.4	41.8	9	32.7	39.4	36.1	25	29.6	45.3	38.5
**BD%**	_	_	_	_	9	68.5	87.7	78.9	25	69.9	99.6	85.1
**BW%**	_	_	_	_	9	30.4	44.5	38.3	25	28.8	50.3	40.2
**BW/BD**	_	_	_	_	9	41.1	56.4	48.5	25	31.0	60.9	47.6
**HD%**	10	58	69.0	59.5	9	57.0	70.5	63.6	25	60.6	73.3	67.9
**HW%**	_	_	_	_	9	55.2	63.7	59.2	25	55.9	67.6	62.9
**BO%**	10	38.5	45.0	42.1	9	23.8	41.7	33.4	25	29.0	45.4	35.8
**PA%**	_	_	_	_	9	72.4	126.7	102.7	25	80.7	123.5	101.2
**P1%**	10	37.5	49.6	45.2	9	31.3	49.8	42.4	25	28.7	46.8	37.8
**AF%**	10	75.2	78.6	77.1	9	63.6	78.5	72.6	25	68.4	81.7	76.3
** **	**N**	**Min**	**Max**	**Mode**	**N**	**Min**	**Max**	**Mode**	**N**	**Min**	**Max**	**Mode**
**BND**	10	0	0	0	9	0	0	0	25	0	0	0
**AFR**	2	227	243	235	9	158	237	198	26	173	232	192
**P1R**	2	15	15	15	9	15	17	15	26	14	19	17
**SAL**	1	9	9	9	9	6	9	7	25	5	8	6
**CEP**	_	_	_	_	9	3	3	3	25	3	3	3
**APS**	_	_	_	_	9	6	11	8	25	4	9	6
**PCV**	1	45	45	45	_	_	_	_	1	48	48	48
**PLR**	10	21	36	28	7	36	50	46	25	28	55	39
**PLL**	10	56	74	65	7	63	87	67	25	44	85	64
**VLR**	10	21	26	25	8	14	31	16	25	5	29	16

### Diagnosis

*Gymnotus* (*Pantherus*) *capitimaculatus* can be differentiated from all other members of the subgenus on the basis of the following characters: color pattern with irregular dark color blotches with blurry, low contrast margins and a pair of white blotches on ventral portion of the head, anterior to or directly below eye (vs. irregular dark color blotches with blurry, low contrast margins with evenly pigmented head, lacking any blotches).

### Description

Sexually monomorphic. Size up to 158 mm TL with adult body proportions attained at about 150 mm TL. Head length short, 8.8–9.9% total length. Snout length moderate, 17.7–20.7% head length. Anal-fin long, 75.2–78.5% total length. Cycloid or ovoid scales present on entire post-cranial portion of body from nape to caudal appendage.

Scales above lateral line intermediate, in 9 rows. Gape large, extending to or beyond posterior nares. Mouth position superior, lower jaw longer than upper, rictus decurved. Chin round in lateral, dorsal profiles, fleshy and bulbous with mental electroreceptive organ overlying lower jaw. Anterior narial pore partially or entirely included within gape, in small narial fold. Anterior nares small, its diameter less than that of eye. Eye below horizontal with mouth. Circumorbital series ovoid. Premaxilla with >11 teeth disposed in two rows along outer margin, median margin curved. Maxilla-palatine articulation near tip of endopterygoid. Maxilla paddle-shaped, broad distally with a straight ventral margin, length equal to >10 dentary teeth. Dentary with one row of 12–15 teeth, all conical. Posterodorsal and posteroventral dentary processes abuts ventral. Dentary posteroventral process shorter than or almost as long as posterodorsal, narrow distally. Dentary ventral margin lamella small, depth less than posterior process. Dentary anteroventral margin with a hook. Anguloarticular process long, extending beyond ventral margin of dentary. Retroarticular with an arched lamella posteriorly forming a small canal, posterior margin squared. Endopterygoid superior and inferior portions approximately equal in size, ascending process robust, straight, tip simple. Interopercle dorsal margin ascending process present. Dorsal region of hyomandibula with four lateral foramenae, supraorbital and infraorbital nerves connected. Preopercle anteroventral notch present, posterodorsal laterosensory ramus with one superficial pore, margin of medial shelf smooth, median shelf large, greater than half width of symplectic. Opercle dorsal margin straight, posterior margin smooth. Cranial fontanels closed in juveniles and adults. Frontal anterior margin straight, postorbital process narrow, less than two times width of supraorbital canal. Lateral ethmoid unossified. Parietal rectangular, length equal to width. Pterophenoid anteroventral process reduced, extends to lateral process.

Parasphenoid posterior processes robust. Prootic foramen Vp combined with V2-3+VII. Adductor mandibula insertion undivided, intermusculars absent. All basibranchials unossified.

Gill rakers not contacting gill bar.

Cleithrum narrow with straight ventral margin, anterior limb long, greater than 1.8 times ascending limb, with facet for insertion of muscle from supracleithrum. Postcleithrum thin, discoid or sickle shaped. Body cavity long, with 45 precaudal vertebrae. Rib 5 broad along its entire extent, greater than three times width of rib 6. Displaced hemal spines absent.

Pectoral fin intermediate, with 15 rays. Anal fin long, with 227–243 rays. Lateral-line complete, with 21–26 ventral rami. Lateral-line dorsal rami absent in adults. Single hypaxial electric organ, extending along entire ventral margin of body.

## *Gymnotus (Pantherus) pantherinus* Steindachner

### Materials examined in morphological analyses

USNM 297933 (94), 35–220 mm, Brazil, Saõ Paolo, Blackwater stream crossing SP193 (24°56’S, 47°58’W).

### Diagnosis

*Gymnotus (Pantherus) pantherinus* is most morphologically similar to *G*. (*P*.) *refugio*, from which it differs on the basis of a relatively slender head (HD 57.0–70.5% TL vs. 60.6–73.3% TL in *G*. (*P*.) *refugio*).

### Description

Sexually monomorphic. Size up to 189 mm TL with adult body proportions attained at about 150 mm TL. Adult body shape subcylindrical with a mean ratio of body width to depth of 48.5%. Body profile slender, body depth 68.5–87.7% total length. Head length short, 7.9–11.1% total length. Snout length moderate, 27.9–35.3% head length. Mouth width narrow, 30.1–48.6% head length. Preanal distance long, 72.4–126.7% head length. Anal-fin long, 63.6–78.5% total length. Cycloid or ovoid scales present on entire post-cranial portion of body from nape to caudal appendage.

Scales above lateral line intermediate, in 6–9 rows. Scales over anal-fin pterygiophores large, with 6–11 rows. Gape large, extending to or beyond posterior nares. Mouth position superior, lower jaw longer than upper, rictus decurved. Chin round in lateral, dorsal profiles, fleshy and bulbous with mental electroreceptive organ overlying lower jaw. Anterior narial pore partially or entirely included within gape, in small narial fold. Anterior nares small, its diameter less than that of eye. Eye below horizontal with mouth. Circumorbital series ovoid. Premaxilla with >11 teeth disposed in two rows along outer margin, median margin curved. Maxilla-palatine articulation near tip of endopterygoid. Maxilla rod- or paddle-shaped, narrow distally with a straight ventral margin, length equal to >10 dentary teeth. Dentary with one row of 12–15 teeth, all conical. Posterodorsal and posteroventral dentary processes abuts ventral. Dentary posteroventral process shorter than or almost as long as posterodorsal, narrow distally. Dentary ventral margin lamella small, depth less than posterior process. Dentary anteroventral margin with a hook. Anguloarticular process long, extending beyond ventral margin of dentary. Retroarticular with an arched lamella posteriorly forming a small canal, posterior margin squared. Endopterygoid superior and inferior portions approximately equal in size, ascending process robust, straight, tip simple. Interopercle dorsal margin ascending process present. Dorsal region of hyomandibula with four lateral foramenae, supraorbital and infraorbital nerves connected. Preopercle anteroventral notch present, posterodorsal laterosensory ramus with 1 superficial pore, margin of medial shelf smooth, median shelf large, greater than half width of symplectic. Opercle dorsal margin straight, posterior margin smooth. Cranial fontanels closed in juveniles and adults. Frontal anterior margin straight, postorbital process narrow, less than two times width of supraorbital canal. Lateral ethmoid unossified. Parietal rectangular, length equal to width. Pterophenoid anteroventral process reduced, extends to lateral process. Parasphenoid posterior processes robust. Prootic foramen Vp combined with V2-3+VII. Adductor mandibula insertion undivided, intermusculars absent. All basibranchials unossified. Gill rakers not contacting gill bar.

Cleithrum narrow with straight ventral margin, anterior limb long, greater than 1.8 times ascending limb, with facet for insertion of muscle from supracleithrum. Postcleithrum thin, discoid or sickle shaped. Rib 5 broad along its entire extent, greater than three times width of rib 6. Displaced hemal spines absent. Pectoral fin broad, with 15–17 rays. Anal fin long, with 158–237 rays. Lateral-line complete, with 14–31 ventral rami. Lateral-line dorsal rami absent in adults. Single hypaxial electric organ, extending along entire ventral margin of body with 3 rows of electroplates near caudal insertion of anal fin.

## *Gymnotus (Pantherus) refugio* Giora & Malabarba

### Materials examined in morphological analyses

UFRGS 8752 (Holotype), 172 mm, Brazil, Rio Grande do Sul State, Laguna dos Patos drainage, Amaral Ferrador, creek in the former Ferraria farm (30°50’54”S, 52°23’19”W); MCP 20197 (Paratypes) (2), 203–214 mm, Brazil, Rio Grande do Sul State, Laguna dos Patos drainage, Viamão, puddle in the swampy forest located at Refúgio da Vida Silvestre Banhado dos Pachecos (30°05’44”S, 50°50’59:W); MZUSP 8749 (Paratypes) (2), 125–157 mm, same locality as UFRGS 8752; UFRGS 8749 (Paratypes) (2), 137–149 mm, same locality as UFRGS 8752; UFRGS 13603 (Paratype), 194 mm, Brazil, Rio Grande do Sul State, Laguna dos Patos drainage, Amaral Ferrador, creek in the former Ferraria farm (30°50’46”S, 52°23’17’W); UFRGS 13618 (Paratypes) (3), 126–160 mm, same locality as UFRGS 13603; UFRGS 13296 (Paratype), 84 mm, Brazil, Rio Grande do Sul State, Laguna dos Patos drainage, Turuçu, Corrientes creek (31°29’48”S, 52°17’42”W); UFRGS 13328 (Paratype), 69 mm, Brazil, Rio Grande do Sul State, Laguna dos Patos drainage, Pelotas, Carmelitas creek (31°44’53”S, 52°13’27”W); UFRGS 20196 (2), 116–168 mm, same locality as UFRGS 13328; UFRGS 13607 (Paratypes) (3), 130–188 mm, Brazil, Rio Grande do Sul State, Laguna dos Patos drainage, Pelotas, Laranjal beach, Carmelitas creek near its mouth in laguna dos Patos (31°44’52”S, 52°13’22”W); UFRGS 13813 (Paratypes) (3), 81–161 mm, Brazil, Rio Grande do Sul State, Laguna dos Patos drainage, Viamão, puddle in the swampy forest at Refúgio da Vida Silvestre Banhado dos Pachecos (30°05’43”S, 50°50’59”W); UFRGS 16371 (Paratypes) (3), 151–186 mm, Brazil, Rio Grande do Sul State, Laguna dos Patos drainage, Viamão, puddle in the swampy forest located at Refúgio da Vida Silvestre Banhado dos Pachecos (30°05’44”S, 50°50’59”W); UFRGS 20197 (Paratypes) (2), 190–214 mm, same locality as UFRGS 16371; UFRGS 20198 (Paratypes) (2), 228–230 mm, same locality as UFRGS 16371; UFRGS 20199 (Paratypes) (2), 203–207 mm, same locality as UFRGS 16371; MNRJ 9439 (Paratypes) (2), 101–117 mm, Brazil, Rio Grande do Sul State, Rio Tramandaí drainage, Torres, Parque Estadual de Itapeva (29°21’23”S, 49°45’57”W); UFRGS 9439 (Paratypes) (2), 80–117 mm, same locality as MNRJ 9439; UFRGS 19595 (Paratypes) (3), 102–216 mm, Brazil, Rio Grande do Sul State, Rio Tramandaí drainage, Maquiné, Barra do Ouro, road to Garapiá waterfall (29°32’14”S, 50°14’45”W); UFRGS 10829 (Paratypes) (2), 68–108 mm, Brazil, Rio Grande do Sul State, Rio Mampituba drainage, Praia Grande, creek tributary of the rio Mampituba (29°10’36”S, 49°58’14”W); UFRGS 10873 (Paratype), 128 mm, Brazil, Rio Grande do Sul State, Rio Mampituba drainage, Praia Grande, flooded area near rio Mampituba (29°15’10”S, 50°07’00”W).

### Diagnosis

*Gymnotus (Pantherus) refugio* is most morphologically similar to *G*. (*P*.) *pantherinus*, from which it differs on the basis of a relatively wide head (HL 60.6–73.3% TL vs. 57.0–70.5% TL in *G*. (*P*.) *pantherinus*).

### Description

Sexually monomorphic. Size up to 234 mm TL with adult body proportions attained at about 150 mm TL. Adult body shape subcylindrical with a mean ratio of body width to depth of 47.6%. Body profile slender, body depth 31.0–60.9% total length. Head length short, 8.6–13.1% total length. Snout length moderate, 24.4–37.6% head length. Mouth width narrow, 31.5–51.2% head length. Preanal distance long, 80.7–123.5% head length. Anal-fin long, 68.4–81.7% total length. Cycloid or ovoid scales present on entire post-cranial portion of body from nape to caudal appendage.

Scales above lateral line intermediate, in 5–8 rows. Scales over anal-fin pterygiophores large, with 4–9 rows. Gape large, extending to or beyond posterior nares. Mouth position superior, lower jaw longer than upper, rictus decurved. Chin round in lateral, dorsal profiles, fleshy and bulbous with mental electroreceptive organ overlying lower jaw. Anterior narial pore partially or entirely included within gape, in small narial fold. Anterior nares small, its diameter less than that of eye. Eye below horizontal with mouth. Circumorbital series ovoid. Premaxilla with >11 teeth disposed in two rows along outer margin, median margin curved. Maxilla-palatine articulation near tip of endopterygoid. Maxilla paddle-shaped, broad distally with a straight ventral margin, length equal to >10 dentary teeth. Dentary with one row of 12–15 teeth, all conical. Posterodorsal and posteroventral dentary processes abuts ventral. Dentary posteroventral process shorter than or almost as long as posterodorsal, narrow distally. Dentary ventral margin lamella small, depth less than posterior process. Dentary anteroventral margin with a hook. Anguloarticular process long, extending beyond ventral margin of dentary. Retroarticular with an arched lamella posteriorly forming a small canal, posterior margin squared. Endopterygoid superior and inferior portions approximately equal in size, ascending process robust, straight, tip simple. Interopercle dorsal margin ascending process present. Dorsal region of hyomandibula with four lateral foramenae, supraorbital and infraorbital nerves connected. Preopercle anteroventral notch present, posterodorsal laterosensory ramus with 1 superficial pore, margin of medial shelf smooth, median shelf large, greater than half width of symplectic. Opercle dorsal margin straight, posterior margin smooth. Cranial fontanels closed in juveniles and adults. Frontal anterior margin straight, postorbital process narrow, less than two times width of supraorbital canal. Lateral ethmoid unossified. Parietal rectangular, length equal to width. Pterophenoid anteroventral process reduced, extends to lateral process. Parasphenoid posterior processes robust. Prootic foramen Vp combined with V2-3+VII. Adductor mandibula insertion undivided, intermusculars absent. All basibranchials unossified. Gill rakers not contacting gill bar.

Cleithrum narrow with straight ventral margin, anterior limb long, greater than 1.8 times ascending limb, with facet for insertion of muscle from supracleithrum. Postcleithrum thin, discoid or sickle shaped. Body cavity long, with 48 precaudal vertebrae. Rib 5 broad along its entire extent, greater than three times width of rib 6. Displaced hemal spines absent. Pectoral fin variable, with 14–19 rays. Anal fin long, with 173–232 rays. Lateral-line complete, with 5–29 ventral rami. Lateral-line dorsal rami absent in adults. Single hypaxial electric organ, extending along entire ventral margin of body with 3 rows of electroplates near caudal insertion of anal fin.

## *Gymnotus* (*Tigre*), subgen. nov. (Table 5)

### Type species

*G*. (*T*.) *tigre*.

### Other included species

G. (T.) esmeraldas, G. (T.) henni, G. (T.) inaequilabiatus, G. (T.) paraguensis.

### Diagnosis

*Tigre* is readily distinguishable from all other subgenera of the Gymnotinae by the following characters: large adult size (except *G*. *(T*.*) paraguensis*) with *G*. *(T*.*) inaequilabiatus* to 998 mm TL and *G*. *(T*.*) tigre* to 533 mm TL representing the two largest species in the subfamily (vs. *G*. *(G*.*) carapo* to 419 mm TL representing the third largest species), color pattern including large white blotches up to 3 times eye diameter (except in *G*. *(T*.*) inaequilabiatus*) covering the head (vs. absent in all other subgenera), dark pigment stripes at caudal end of anal-fin membrane (vs. clear patch or evenly pigmented in all other subgenera), more than 16 dentary teeth, all conical, some decurved with tips oriented outward in large specimens (vs. all straight and conical or needle-shapedneedle-shaped in the subgenera *Lamontianus*, *Pantherus*, *Tigrinus* and *Tijax*, or anteriormost 2–9 arrowhead-shaped in the subgenus *Gymnotus*), four to six rows of electrocytes caudally (vs. two to four rows in other subgenera), axially-elongate scales covering posterior 20% of body and caudal appendage (vs. ovoid scales over entire body in other subgenera). *Gymnotus* (*Tigre*) is morphologically most similar to *Gymnotus* (*Gymnotus*), from which is it readily distinguishable by the following characters: many, short, straight ventral lateral-line rami (VLR 23–55) vs. relatively few, long, curved ventral lateral-line rami (VLR 7–30), long body cavity (PCV 32–48) vs. body cavity of intermediate length (PCV 31–43).

### Description

Sexually monomorphic. Size up to 998 mm TL in *G*. *(T*.*) inaequilabiatus*, with adult body proportions attained by about 150 mm TL. Adult body shape subcylindrical with a mean ratio of body width to depth of about 72.1%. Body profile deep, body depth 68.9–100.6% total length. Head length intermediate, 8.2–13.3% total length. Snout length moderate, 31.7–39.9% head length. Mouth width intermediate, 37.2–49.1% head length. Preanal distance variable, 45.6–112.3%head length. Anal-fin long, 73.0–87.8% total length. Cycloid or ovoid scales present on entire post-cranial portion of body from nape to caudal appendage.

Scales above lateral line small, in 6–13 rows. Scales over anal-fin pterygiophores small, with 9–16 rows. Gape large, extending to or beyond posterior nares. Mouth position superior, lower jaw longer than upper, rictus decurved. Chin round in lateral, dorsal profiles, fleshy and bulbous with mental electroreceptive organ overlying lower jaw. Anterior narial pore partially or entirely included within gape, in small narial fold. Anterior nares small, its diameter less than that of eye. Eye below horizontal with mouth. Circumorbital series ovoid. Premaxilla with fewer than ten teeth disposed in two rows along outer margin, median margin straight. Maxilla-palatine articulation near tip of endopterygoid. Maxilla vertical, rod- or paddle-shaped distally, length equal to width of 7–9 dentary teeth. Dentary with one row of comical teeth, occasionally decurved (points facing outward from body) in large adult specimens, all others conical. Posterodorsal and posteroventral dentary processes abuts ventral. Dentary posteroventral process shorter than or almost as long as posterodorsal, narrow distally. Dentary ventral margin lamella small, depth less than posterior process (except in *G*. *(T*.*) paraguensis*). Dentary anteroventral margin lacking a hook. Mandible long and extended. Anguloarticular process short, extending to ventral margin of dentary. Retroarticular with an arched lamella posteriorly forming a small canal, posterior margin squared. Endopterygoid superior and inferior portions approximately equal in size, ascending process robust, long, straight (except in *G*. *(T*.*) inaequilabiatus* and *G*. *(T*.*) tigre*), tip simple. Interopercle dorsal margin ascending process present. Dorsal region of hyomandibula with four lateral foramenae, supraorbital and infraorbital nerves divided. Preopercle anteroventral notch present, posterodorsal laterosensory ramus with two superficial pores, margin of medial shelf entirely smooth, median shelf large, greater than half width of symplectic. Opercle dorsal margin concave, posterior margin entirely smooth. Subopercle dorsal margin concave. Cranial fontanels closed in juveniles and adults. Frontal broad, anterior margin straight, postorbital process broad, greater than two times width of supraorbital canal. Lateral ethmoid unossified. Parietal rectangular, length equal to width. Pterosphenoid anteroventral portion robust, extends ventrally to lateral margin of parasphenoid. Parasphenoid posterior processes robust, with shallow, convex posterior margin. Prootic foramen Vp separate from V2-3+VII. *M*. *adductor mandibula* intermusculars absent. All basibranchials unossified. Gill rakers not contacting gill bar.

Cleithrum broad with curved ventral margin, anterior limb long, greater than 1.8 times ascending limb, lacking large facet for insertion of muscle from supracleithrum. Postcleithrum thin, discoid or sickle shaped. Body cavity long, with 32–48 precaudal vertebrae. Rib 5 robust along its entire extent, less than three times width of rib 6. Displaced hemal spines absent. Pectoral fin large to very large, with 13–21 rays. Anal fin long, with 190–390 rays. Lateral-line complete, with 23–55 ventral rami. Lateral-line dorsal rami absent in adults. Single hypaxial electric organ, extending along entire ventral margin of body with 3–6 rows of electroplates near caudal insertion of anal fin.

### Etymology

Subgenus name derived from the local aquarium trade name of the type species, referring to the color pattern of alternating dark and light bands.

Diagnoses and descriptions of each species of *Tigre* (Table 15)

**Table 15:** Summary of morphometric and meristic data for five species of *Tigre*. Data for 46 specimens.

*** ***	***G*. *(T*.*) esmeraldas***				***G*. *(T*.*) henni***				***G*. *(T*.*) inaequilabiatus***				***G*. *(T*.*) paraguensis***				***G*. *(T*.*) tigre***			
** **	**N**	**Min**	**Max**	**AVG**	**N**	**Min**	**Max**	**AVG**	**N**	**Min**	**Max**	**AVG**	**N**	**Min**	**Max**	**AVG**	**N**	**Min**	**Max**	**AVG**
**TL**	8	200	356	266	5	254	312	289	26	130	998	431	4	164	240	205	5	173	411	307
**HL**	8	21.5	36.5	27.6	5	23.8	30.9	28.1	22	13.5	82	42.5	4	19.6	27.3	23.2	5	17.1	46.5	35.2
**HL%**	8	9.1	12.4	10.4	5	9.2	10.7	9.7	22	8.2	12.8	10.6	4	9.8	12.2	11.4	5	11	13.3	11.3
**PR%**	8	34.8	39.6	37.8	5	35.7	39.9	38.5	21	31.7	39.4	37.5	4	34	35.7	35.2	5	35.1	39.6	38.1
**MW%**	8	41.5	46	42.9	5	43.2	49.4	46.7	17	39.5	49.1	44.1	4	37.2	39	38.4	5	40.4	43.8	42.5
**PO%**	8	58.1	63.1	61.4	5	59.1	62.2	60.7	17	58.5	63.4	61	4	57.7	62.2	60.8	5	61.4	62.9	62.2
**IO%**	7	41.8	47.9	44.4	5	42	47.6	45.1	21	35.8	50.8	43.5	4	31.1	40.7	34.6	5	44.7	48	45.9
**BD%**	7	82.4	91.4	87.2	5	73.2	98.7	84.8	16	81.1	100.5	93.2	4	84.3	96.2	87.6	5	72.8	100.6	84.7
**BW%**	8	52.3	64.9	57.9	5	61.1	76.1	67.5	15	37.5	80.8	65.9	4	51.5	76.5	61.1	4	55.8	65.4	59.4
**BW/BD**	8	58.1	77	68.6	5	77	86.9	79.9	15	38.6	82.5	70.8	4	60.5	79.6	69.4	5	64.8	83.1	75.6
**HD%**	8	54.5	58.9	56.8	5	52.8	56.8	55.1	15	50.7	72.6	60.4	4	56.7	69.2	60.9	5	53.7	60.2	55.5
**HW%**	7	63.3	68.8	66	4	62.6	70.6	66.8	15	59.8	75.1	68	4	57.7	66.3	60.1	5	58.1	67.3	61.5
**BO%**	8	27.4	33	29.9	5	30.5	32.8	31.8	5	43.7	76.5	63.1	3	31.8	35.7	34.2	5	27	31.8	28.9
**PA%**	8	81.9	101.8	92.8	5	90.1	112.3	99.7	6	45.6	74.6	55.9	4	62.8	85	73	5	69.7	81.9	75
**P1%**	8	40.8	47.4	43.3	5	44.9	53.2	48.4	12	39	47.9	44.7	4	37.4	48.3	42.3	4	40.1	45.9	42.9
**AF%**	6	76.5	82.9	80.5	5	79.5	81.5	80.6	16	73.2	87.8	82.6	4	79.7	81.7	80.6	5	73	80.1	77.8
** **	**N**	**Min**	**Max**	**Mode**	**N**	**Min**	**Max**	**Mode**	**N**	**Min**	**Max**	**Mode**	**N**	**Min**	**Max**	**Mode**	**N**	**Min**	**Max**	**Mode**
**BND**	8	0	0	0	7	13	16	15	12	21	29	22	3	23	26	24	5	17	23	23
**AFR**	3	226	260	253	5	223	275	249	11	214	390	370	2	210	260	235	5	190	240	225
**P1R**	8	17	19	17	7	17	19	17	18	13	21	19	3	17	21	19	4	17	19	17
**SAL**	8	8	9	9	7	10	13	10	11	6	13	12	3	7	12	11	5	11	12	12
**CEP**	8	4	5	4	7	3	6	6	4	4	4	4	3	3	4	3	5	3	6	5
**APS**	8	11	13	11	7	10	13	12	12	9	14	9	3	14	15	14	5	13	16	15
**PCV**	5	41	46	44	2	43	44	44	2	45	45	45	2	32	35	34	3	41	48	46
**PLR**	8	52	56	54	7	59	68	68	13	43	54	48	3	40	49	48	5	62	78	67
**PLL**	_	_	_	_	4	95	125	105	4	98	153	130	3	80	135	114	4	125	140	131
**VLR**	_	_	_	_	_	_	_	_	10	23	38	32	3	49	55	50	_	_	_	_

## *Gymnotus (Tigre) esmeraldas* Albert & Crampton

### Materials examined in morphological analyses

MCZ 58729 (Holotype), 296 mm, Ecuador, Río Esmeraldas drainage, Ríos Cayapas at Hoja Blanca near San Miguel (01°05’N, 79°03’W); MCZ 162745 (Paratypes) (4), 200–309 mm same locality as MCZ 58729; CAS 164103, 355 mm, Ecuador, Río Esmeraldas drainage, Río Blanco, Río Toachi near mouth of Río Quinide; FMNH 92041 (2), 229–288 mm, Ecuador, Los Rios, Río Guayaquil drainage, Río Palenque Biological Station near Station 16.

### Diagnosis

*Gymnotus* (*Tigre*) *esmeraldas* can be differentiated from all other members of the subgenus on the basis of the following characters: color pattern with dark band pairs absent over anterior 80% of body, replaced by irregular pale blotches about 2–3 scales across.

### Description

Sexually monomorphic. Size up to 356 mm TL with adult body proportions attained at about 150 mm TL. Adult body shape subcylindrical with a mean ratio of body width to depth of 68.6%. Body profile slender, body depth 82.4–91.4% total length. Head length long, 9.1–12.4% total length. Snout length moderate, 34.8–39.6% head length. Mouth width narrow, 41.5–46.0% head length. Preanal distance long, 81.9–101.8% head length. Anal-fin long, 76.5–82.9% total length. Cycloid or ovoid scales present on entire post-cranial portion of body from nape to caudal appendage.

Scales above lateral line intermediate, in 8–9. rows Scales over anal-fin pterygiophores large, with 11–13 rows. Gape large, extending to or beyond posterior nares. Mouth position superior, lower jaw longer than upper, rictus decurved. Chin round in lateral, dorsal profiles, fleshy and bulbous with mental electroreceptive organ overlying lower jaw. Anterior narial pore partially or entirely included within gape, in small narial fold. Anterior nares small, its diameter less than that of eye. Eye below horizontal with mouth. Circumorbital series ovoid. Premaxilla with <10 teeth disposed in two rows along outer margin, median margin straight. Maxilla-palatine articulation near tip of endopterygoid. Maxilla rod- or paddle-shaped with a straight ventral margin, length equal to 7–9 dentary teeth. Dentary with one row of 12–15 teeth, 2–4 arrowhead shaped anteriorly, all others conical posteriorly. Posterodorsal and posteroventral dentary processes abuts ventral. Dentary posteroventral process shorter than or almost as long as posterodorsal, narrow distally. Dentary ventral margin lamella small, depth less than posterior process. Dentary anteroventral margin with a hook. Anguloarticular process short, extending to ventral margin of dentary. Retroarticular with an arched lamella posteriorly forming a small canal, posterior margin squared. Endopterygoid superior and inferior portions approximately equal in size, ascending process robust, straight, tip simple. Interopercle dorsal margin ascending process present. Dorsal region of hyomandibula with four lateral foramenae, supraorbital and infraorbital nerves divided. Preopercle anteroventral notch present, posterodorsal laterosensory ramus with 2 superficial pores, margin of medial shelf smooth, median shelf long, greater than half width of symplectic. Opercle dorsal margin concave, posterior margin smooth. Cranial fontanels closed in juveniles and adults. Frontal anterior margin straight, postorbital process broad, greater than two times width of supraorbital canal. Lateral ethmoid unossified. Parietal rectangular, length equal to width. Pterophenoid anteroventral process robust, extends to lateral process. Parasphenoid posterior processes robust. Prootic foramen Vp separate from V2-3+VII. Adductor mandibula insertion undivided, intermusculars absent. All basibranchials unossified. Gill rakers not contacting gill bar.

Cleithrum broad with curved ventral margin, anterior limb long, greater than 1.8 times ascending limb, with facet for insertion of muscle from supracleithrum. Postcleithrum thin, discoid or sickle shaped. Body cavity long, with 41–46 precaudal vertebrae. Rib 5 robust along its entire extent, less than three times width of rib 6. Displaced hemal spines absent. Pectoral fin broad, with 17–19 rays. Anal fin long, with 226–260 rays. Lateral-line complete. Lateral-line dorsal rami absent in adults. Single hypaxial electric organ, extending along entire ventral margin of body with 4–5 rows of electroplates near caudal insertion of anal fin.

## *Gymnotus* (*Tigre*) *henni* Albert & Crampton

### Materials examined in morphological analyses

CAS 47290 (Holotype), 308 mm, Colombia, Valle de Cauca, north of Buenaventura, Río San Juan drainage, creek near mouth of Río Calima (03°53’N, 77°04’W); CAS 217162 (Paratype), 145 mm, same locality as CAS 47290; FMNH 56793 (Paratypes) (2), 254–273 mm, same locality as CAS 47290; ICNMHN 96 (7), 127–371 mm Colombia, Chocó, Río Baudó; ICNMHN 102, 237 mm, Colombia, Chocó, Río Baudó near Pavarandó; ICNMHN 2284, 346 mm, Colombia, Risaralda, Pueblo Rico, upper Río San Juan, Río Baudó over road to Pie de Pepe (05°07’N, 76°50’W); IMCN 1369, 333 mm, Colombia, Chocó, Litoral del San Juan, Río San Juan, Resguardo Puerto Pizarro; USNM 246793 (3), 131–314 mm, Colombia, Chocó, creek off Río Juradó (07°06’N, 77°46’W).

### Diagnosis

*Gymnotus* (*Tigre*) *henni* can be differentiated from all other members of the subgenus on the basis of the following characters: color pattern with few dark band pairs (BND 13–16 vs. absent in *G*. *(T*.*) inaequilabiatus*, 23–26 in *G*. *(T*.*) paraguensis*, 17–23 in *G*. *(T*.*) tigre*). *Gymnotus (Tigre) henni* is most morphologically similar to *G*. *(T*.*) tigre*, from which it differs on the basis of a relatively wide mouth (MW 43.2–49.4% HL vs. 40.4–43.8% HL in *G*. *(T*.*) tigre*), relatively short head (HL 9.2–10.7% TL vs. 11.0–13.3% TL in in *G*. *(T*.*) tigre*).

### Description

Sexually monomorphic. Size up to 312 mm TL with adult body proportions attained at about 150 mm TL. Adult body shape subcylindrical with a mean ratio of body width to depth of 79.9%. Body profile slender, body depth 77.0–86.9% total length. Head length intermediate, 9.2–10.7% total length. Snout length moderate, 35.7–39.9% head length. Mouth width narrow, 43.2–49.4% head length. Preanal distance long, 90.1–112.3% head length. Anal-fin long, 79.5–81.5% total length. Cycloid or ovoid scales present on entire post-cranial portion of body from nape to caudal appendage.

Scales above lateral line intermediate, in 10–13 rows. Scales over anal-fin pterygiophores large, with 10–13 rows. Gape large, extending to or beyond posterior nares. Mouth position superior, lower jaw longer than upper, rictus decurved. Chin round in lateral, dorsal profiles, fleshy and bulbous with mental electroreceptive organ overlying lower jaw. Anterior narial pore partially or entirely included within gape, in small narial fold. Anterior nares small, its diameter less than that of eye. Eye below horizontal with mouth. Circumorbital series ovoid. Premaxilla with <10 teeth disposed in two rows along outer margin, median margin straight. Maxilla-palatine articulation near tip of endopterygoid. Maxilla rod- or paddle-shaped, with a straight ventral margin. Dentary with one row of 12–15 teeth, 2–4 arrowhead shaped anteriorly, all others conical posteriorly. Posterodorsal and posteroventral dentary processes abuts ventral. Dentary posteroventral process shorter than or almost as long as posterodorsal, narrow distally. Dentary ventral margin lamella small, depth less than posterior process. Dentary anteroventral margin with a hook. Anguloarticular process short, extending to ventral margin of dentary. Retroarticular with an arched lamella posteriorly forming a small canal, posterior margin squared. Endopterygoid superior and inferior portions approximately equal in size, ascending process robust, straight, tip simple. Interopercle dorsal margin ascending process absent. Dorsal region of hyomandibula with four lateral foramenae, supraorbital and infraorbital nerves divided. Preopercle posterodorsal laterosensory ramus with 2 superficial pores, margin of medial shelf smooth, median shelf large, greater than half width of symplectic. Opercle posterior margin smooth. Cranial fontanels closed in juveniles and adults. Frontal anterior margin straight, postorbital process broad, greater than two times width of supraorbital canal. Lateral ethmoid unossified. Parietal rectangular, length equal to width. Parasphenoid posterior processes robust. Prootic foramen Vp separate V2-3+VII. Adductor mandibula insertion undivided, intermusculars absent. All basibranchials unossified. Gill rakers not contacting gill bar.

Cleithrum broad with curved ventral margin, anterior limb long, greater than 1.8 times ascending limb, with facet for insertion of muscle from supracleithrum. Postcleithrum thin, discoid or sickle shaped. Body cavity intermediate, with 43–44 precaudal vertebrae. Rib 5 robust along its entire extent, less than three times width of rib 6. Displaced hemal spines absent. Pectoral fin broad, with 17–19 rays. Anal fin long, with 223–275 rays. Lateral-line complete. Lateral-line dorsal rami absent in adults. Single hypaxial electric organ, extending along entire ventral margin of body with 3–6 rows of electroplates near caudal insertion of anal fin.

## *Gymnotus (Tigre) inaequilabiatus* Valenciennes

### Materials examined in morphological analyses

MNHN 4615 (neotype), 920 mm (600 mm according to the original description, 850 mm according to the label), Argentina, Buenos Aires, Rio de La Plata (~30°40’S, 058°30’W according to MNHN database or ~34°37’S, 058°23’W); MACN 5979, 250 mm, Argentina, Rosario, Río La Plata (~32°56’S, 060°38’W); MLP 3353, 250 mm, Argentina, Misiones, Río Paraná (~26°49’S, 055°00’W); LBP 12616, ~650 mm, Brazil, Mato Grosso, Corumbá, Rio Paraguai drainage, Rio Cuiabá (~18°09’S, 057°33’W); MCP 15818, 535 mm, Brazil, Mato Grosso do Sul, Rio Paraguai near Cáceres; USNM 1643, 791 mm, Brazil, Mato Grosso do Sul, Rio Paraguai drainage (~17°53’S, 057°29’W); MCP 6059 (3), 173–355 mm, Brazil, Rio Grande do Sul, Iraí, Rio Uruguai drainage, Sanga das Águas Frias, 100 m from Rio Uruguai (27°12’00”S, 053°16’60”W); MCP 6956, 602 mm, Brazil, Rio Grande do Sul, Rio Uruguai at Santana Velha, near Uruguaiana (~29°45’S, 057°50’W); MCP 19552 (3), 145–336 mm, Brazil, Rio Grande do Sul, Rosário do Sul, pool next to Olaria in the floodplain along Rio Santa Maria, North side of BR290 (30°14’16”, 054°53’33”W); MCP 31177, 560 mm, Brazil, Rio Grande do Sul, Roque Gonzales, Rio Uruguai drainage, Rio Ijuí near small hydroelectric power station (PCH) Pirapó (~28°30’S, 055°10’W); MCP 39375, 550 mm, same locality as MCP 31177; UFRGS 14457, 498 mm, Brazil, Rio Grande do Sul (~27°11’S, 053°06’W); MCP 19044, 440 mm, Brazil, Santa Catarina, Itá, Rio Uruguai, Rio do Engano, road between Itá and Seara (27°08’60”S, 52°13’00”W); MCP 20683, 410 mm, Brazil, Santa Catarina, Itapiranga, Rio Uruguai, near Fortaleza (27°11’42”S, 053°38’34”W); MZUSP 22246 (3), 130–282 mm, Brazil, São Paulo, Represa do Boa, Corumbataí, Rio Paraná drainage, Rio Tietê (~21°46’S, 048°58’W); MZUSP 22862, 238 mm, Brazil, São Paulo, Corumbataí, Rio Paraná drainage, Rio Tietê, Lagoa Ponte Seca (~21°46’S, 048°58’W); MZUSP 46001, 998 mm, Brazil, São Paulo, Rio Paraná drainage, Porto Primavera (~22°31’S, 053°10’W).

### Diagnosis

*Gymnotus* (*Tigre*) *inaequilabiatus* can be differentiated from all other members of the subgenus on the basis of the following characters: Largest adult size of any Gymnotinae (max. 998 mm TL, vs. 356 mm TL in *G*. *(T*.*) esmeraldas*, 312 mm TL in *G*. *(T*.*) henni*, 240 mm TL in *G*. *(T*.*) paraguensis*, 411 mm TL in *G*. *(T*.*) tigre*), color pattern with obliquely oriented, dark brown band pairs with wavy, irregular margins in juveniles (TL <250 mm), replaced in adults by large (5 or more scales across), irregular dark brown blotches on a pale brown ground color in adults (TL >250 mm) (vs. dark band pairs absent over anterior 80% of body, replaced by irregular pale blotches about 2–3 scales across in *G*. *(T*.*) esmeraldas*, BND 13–16 in *G*. *(T*.*) henni*, 23–26 in *G*. *(T*.*) paraguensis*, 17–23 in *G*. *(T*.*) tigre*).

### Description

Sexually monomorphic. Size up to 998 mm TL with adult body proportions attained at about 150 mm TL. Adult body shape subcylindrical with a mean ratio of body width to depth of 70.8%. Body profile slender, body depth 81.1–100.5% total length. Head length variable, 8.2–12.8% total length. Snout length moderate, 31.7–39.4% head length. Mouth width narrow, 39.5–49.1% head length. Preanal distance long, 46.5–74.6% head length. Anal-fin long, 73.2–87.8% total length. Cycloid or ovoid scales present on entire post-cranial portion of body from nape to caudal appendage.

Scales above lateral line intermediate, in 6–13 rows. Scales over anal-fin pterygiophores large, with 9–14 rows. Gape large, extending to or beyond posterior nares. Mouth position superior, lower jaw longer than upper, rictus decurved. Chin round in lateral, dorsal profiles, fleshy and bulbous with mental electroreceptive organ overlying lower jaw. Anterior narial pore partially or entirely included within gape, in small narial fold. Anterior nares small, its diameter less than that of eye. Eye below horizontal with mouth. Circumorbital series ovoid. Premaxilla with <10 teeth disposed in two rows along outer margin, median margin straight. Maxilla-palatine articulation near tip of endopterygoid. Maxilla rod- or paddle-shaped with a straight ventral margin. Dentary with one row of 12–15 teeth, 2–4 arrowhead shaped anteriorly, all others conical posteriorly. Posterodorsal and posteroventral dentary processes abuts ventral. Dentary posteroventral process shorter than or almost as long as posterodorsal, narrow distally. Dentary ventral margin lamella small, depth less than posterior process. Dentary anteroventral margin with a hook. Anguloarticular process short, extending to ventral margin of dentary. Retroarticular with an arched lamella posteriorly forming a small canal, posterior margin squared. Endopterygoid superior and inferior portions approximately equal in size, ascending process robust, curved, tip simple. Interopercle dorsal margin ascending process present. Dorsal region of hyomandibula with four lateral foramenae, supraorbital and infraorbital nerves divided. Preopercle anteroventral notch present, posterodorsal laterosensory ramus with 2 superficial pores, margin of medial shelf smooth, median shelf large, greater than half width of symplectic. Opercle margin smooth. Cranial fontanels closed in juveniles and adults. Frontal anterior margin straight, postorbital process broad, greater than two times width of supraorbital canal. Lateral ethmoid unossified. Parietal rectangular, length equal to width. Pterophenoid anteroventral process robust, extends to lateral process. Parasphenoid posterior processes broad. Prootic foramen Vp separate from V2-3+VII. Adductor mandibula insertion undivided, intermusculars absent. All basibranchials unossified. Gill rakers not contacting gill bar.

Cleithrum broad with curved ventral margin, anterior limb long, greater than 1.8 times ascending limb, with facet for insertion of muscle from supracleithrum. Postcleithrum thin, discoid or sickle shaped. Body cavity intermediate, with 45 precaudal vertebrae. Rib 5 robust along its entire extent, less than three times width of rib 6. Displaced hemal spines absent. Pectoral fin variable, with 13–21 rays. Anal fin long, with 214–390 rays. Lateral-line complete, with 23–38 ventral rami. Lateral-line dorsal rami absent in adults. Single hypaxial electric organ, extending along entire ventral margin of body with 4 rows of electroplates near caudal insertion of anal fin.

## *Gymnotus (Tigre) paraguensis* Albert & Crampton

### Materials examined in morphological analyses

UMMZ 206155 (holotype), 222 mm, Paraguay, Department of Paraguay, Itapua, Río Paraná drainage, Arroyo Tembey, 7.4 km SW of San Rafael de Paraná (26°35’S, 055°34’W); UMMZ 240700 (paratype), 193 mm, same locality as UMMZ 206155; ANSP uncat., Uruguay, Río Negro, Nuevo Berlín, Río de La Plata drainage, Río Uruguay, along shore near municipal pier (32°58’46”S, 058°3’48”W); FMNH 108546, 164 mm, Brazil, Mato Grosso do Sul, Aquiduana, Brejo do Santa Sofia, Rio Novo (19°35’89”S, 056°20’47”W); MCP 13415, Brazil, Santa Catarina, Rio Uruguai drainage, Rio Uruguai/Itá (27°17’60”S, 052°19’60”W); NRM 42380, 240 mm, Paraguay, Canindeyú, Saltos da Guaira, Mulle Ytaipú, Río Paraná drainage (24°03’44”S, 054°18’W); NRM 42397, 171 mm, Paraguay, Central Province, Río Paraguay drainage, swamp close to Lago Ypacarai; MNHNP 4169, Paraguay, Department of Paraguay, Itapua, Capitan Meza Río de La Plata drainage, Río Paraná, Capitan Meza, Club de Caza y Pesca de Capitan Meza (26°57’44”S, 055°10’57”W).

### Diagnosis

*Gymnotus* (*Tigre*) *paraguensis* can be differentiated from all other members of the subgenus on the basis of the following characters: short snout (PR 34.0–35.7% HL vs. 36.0–39.6% HL in *G*. *(T*.*) esmeraldas*, 35.7–39.8% HL in *G*. *(T*.*) henni*, 36.3–39.5% HL in *G*. *(T*.*) inaequilabiatus*, 35.1–39.6% HL in *G*. *(T*.*) tigre*), short body cavity (PCV 32–35 vs. 41–46 in *G*. *(T*.*) esmeraldas*, 43–44 in *G*. *(T*.*) henni*, 44–45 in *G*. *(T*.*) inaequilabiatus*, 41–48 in *G*. *(T*.*) tigre*), premaxilla with straight median margin (vs. curved in all other *Tigre*), dentary ventral margin lamella large (>posterior process vs. <posterior process in all other *Tigre*).

### Description.

Sexually monomorphic. Size up to 240 mm TL with adult body proportions attained at about 150 mm TL. Adult body shape subcylindrical with a mean ratio of body width to depth of 69.4%. Body profile slender, body depth 84.3–96.2% total length. Head length short, 9.8–12.2% total length. Snout length moderate, 34.0–35.7% head length. Mouth width narrow, 37.2–39.0% head length. Preanal distance long, 62.8–85.0% head length. Anal-fin long, 79.7–81.7% total length. Cycloid or ovoid scales present on entire post-cranial portion of body from nape to caudal appendage.

Scales above lateral line intermediate, in 7–12 rows. Scales over anal-fin pterygiophores large, with 14–14 rows. Gape large, extending to or beyond posterior nares. Mouth position superior, lower jaw longer than upper, rictus decurved. Chin round in lateral, dorsal profiles, fleshy and bulbous with mental electroreceptive organ overlying lower jaw. Anterior narial pore partially or entirely included within gape, in small narial fold. Anterior nares small, its diameter less than that of eye. Eye below horizontal with mouth. Circumorbital series ovoid. Premaxilla with <10 teeth disposed in two rows along outer margin, median margin curved. Maxilla-palatine articulation near tip of endopterygoid. Maxilla rod- or paddle-shaped, narrow distally with a straight ventral margin. Dentary with one row of 12–15 teeth, 2–4 arrowhead shaped anteriorly, all others conical posteriorly. Posterodorsal and posteroventral dentary processes abuts ventral. Dentary posteroventral process shorter than or almost as long as posterodorsal, narrow distally. Dentary ventral margin lamella short, depth less than posterior process. Dentary anteroventral margin with a hook. Anguloarticular process short, extending to ventral margin of dentary. Retroarticular with an arched lamella posteriorly forming a small canal, posterior margin squared. Endopterygoid superior and inferior portions approximately equal in size, ascending process robust, long, tip simple. Interopercle dorsal margin ascending process present. Dorsal region of hyomandibula with four lateral foramenae, supraorbital and infraorbital nerves divided. Preopercle anteroventral notch present, posterodorsal laterosensory ramus with 2 superficial pores, margin of medial shelf smooth, median shelf large, greater than half width of symplectic. Opercle dorsal margin smooth. Cranial fontanels closed in juveniles and adults. Frontal anterior margin straight, postorbital process broad, greater than two times width of supraorbital canal. Lateral ethmoid unossified. Parietal rectangular, length equal to width. Pterophenoid anteroventral process robust, extends to lateral process. Parasphenoid posterior processes robust. Prootic foramen Vp separate from V2-3+VII. Adductor mandibula insertion undivided, intermusculars absent. All basibranchials unossified. Gill rakers not contacting gill bar.

Cleithrum broad with curved ventral margin, anterior limb long, greater than 1.8 times ascending limb, with facet for insertion of muscle from supracleithrum. Postcleithrum thin, discoid or sickle shaped. Body cavity short, with 32–35 precaudal vertebrae. Rib 5 robust along its entire extent, less than three times width of rib 6. Displaced hemal spines absent. Pectoral fin broad, with 17–21 rays. Anal fin long, with 210–260 rays. Lateral-line complete, with 49–55 ventral rami. Lateral-line dorsal rami absent in adults. Single hypaxial electric organ, extending along entire ventral margin of body with 4–4 rows of electroplates near caudal insertion of anal fin.

## *Gymnotus* (*Tigre*) *tigre* Albert & Crampton

### Materials examined in morphological analyses

UF 25552 (Holotype), 411 mm, Colombia, floating macrophytes along north shore of Río Amazonas near Leticia (04°09’S, 69°57’W); UF 128412 (Paratype), 332 mm, same locality as UF 25552; ICNMHN 6690 (Paratype), 340 mm, same locality as UF 25552; IAVHP 0615, 179 mm, Colombia, Amazonas, Rio Amazonas near Leticia (04°09’S, 69°57’W); INPA 6814, 113 mm, Brazil, Pará, Rio Tapajós drainage, Rio Jamanxim at Ilha Terra Grande; FMNH 97389, 292 mm, Peru, Pastaza, Río Pastaza drainage, Río Bobonaza, near Canelos (01°39’S, 77°46’W); UF 117112 (5), 89–171 mm, Peru, Loreto, acquired from fishermen on Río Amazonas near Iquitos (~03°46’S, 73°15’W); UF 122821, 331 mm, same locality as UF 117112; NRM 27644, 104 mm, Peru, Loreto, Río Putumayo at El Estrecho (02°28’S, 72°42’W).

### Diagnosis

*Gymnotus* (*Tigre*) *tigre* can be differentiated from all other members of the subgenus on the basis of the following characters: color pattern with large (5 or more scales across) irregular pale blotches on ventral and posterior portions of head and over opercle (vs. head evenly-pigmented in all other *Tigre*). *Gymnotus (Tigre) tigre* is most morphologically similar to *G*. *(T*.*) henni*, from which it differs on the basis of a relatively narrow mouth (40.4–43.8% HL vs. MW 43.2–49.4% HL in *G*. *(T*.*) henni*), relatively long head (HL11.0–13.3% TL vs. 9.2–10.7% TL vs. in *G*. *(T*.*) henni*).

### Description

Sexually monomorphic. Size up to 411 mm TL with adult body proportions attained at about 150 mm TL. Adult body shape subcylindrical with a mean ratio of body width to depth of 75.6%. Body profile slender, body depth 72.8–100.6% total length. Head length long, 11.0–13.3% total length. Snout length moderate, 35.1–39.6% head length. Mouth width narrow, 40.4–43.8% head length. Preanal distance long, 69.7–81.9% head length. Anal-fin long, 73.0–80.1% total length. Cycloid or ovoid scales present on entire post-cranial portion of body from nape to caudal appendage.

Scales above lateral line intermediate, in 11–12 rows. Scales over anal-fin pterygiophores large, with 13–16 rows. Gape large, extending to or beyond posterior nares. Mouth position superior, lower jaw longer than upper, rictus decurved. Chin round in lateral, dorsal profiles, fleshy and bulbous with mental electroreceptive organ overlying lower jaw. Anterior narial pore partially or entirely included within gape, in small narial fold. Anterior nares small, its diameter less than that of eye. Eye below horizontal with mouth. Circumorbital series ovoid. Premaxilla with <10 teeth disposed in two rows along outer margin, median margin straight. Maxilla-palatine articulation near tip of endopterygoid. Maxilla rod- or paddle-shaped, narrow distally with a straight ventral margin. Dentary with one row of 12–15 teeth, 2–4 arrowhead shaped anteriorly, all others conical posteriorly. Posterodorsal and posteroventral dentary processes abuts ventral. Dentary posteroventral process shorter than or almost as long as posterodorsal, narrow distally. Dentary ventral margin lamella small, depth less than posterior process. Dentary anteroventral margin without a hook. Anguloarticular process short, extending to ventral margin of dentary. Retroarticular with an arched lamella posteriorly forming a small canal, posterior margin squared. Endopterygoid superior and inferior portions approximately equal in size, ascending process robust, curved, tip simple. Interopercle dorsal margin ascending process present. Dorsal region of hyomandibula with four lateral foramenae, supraorbital and infraorbital nerves divided. Preopercle anteroventral notch present, posterodorsal laterosensory ramus with 2 superficial pores, margin of medial shelf smooth, median shelf large, greater than half width of symplectic. Opercle posterior margin smooth. Cranial fontanels closed in juveniles and adults. Frontal anterior margin straight, postorbital process broad, greater than two times width of supraorbital canal. Lateral ethmoid unossified. Parietal rectangular, length equal to width. Pterophenoid anteroventral process robust, extends to lateral process. Parasphenoid posterior processes robust. Prootic foramen Vp separate from V2-3+VII. Adductor mandibula insertion undivided, intermusculars absent. All basibranchials unossified. Gill rakers not contacting gill bar.

Cleithrum broad with curved ventral margin, anterior limb long, greater than 1.8 times ascending limb, with facet for insertion of muscle from supracleithrum. Postcleithrum thin, discoid or sickle shaped. Body cavity long, with 41–48 precaudal vertebrae. Rib 5 robust along its entire extent, less than three times width of rib 6. Displaced hemal spines absent. Pectoral fin broad, with 17–19 rays. Anal fin long, with 190–240 rays. Lateral-line complete. Lateral-line dorsal rami absent in adults. Single hypaxial electric organ, extending along entire ventral margin of body with 3–6 rows of electroplates near caudal insertion of anal fin.

## *Gymnotus* (*Tigrinus*), subgen. nov. (Table 5)

### Type species

*G*. *(T*.*) coatesi*.

### Other included species

*G*. *(T*.*) coropinae*, *G*. *(T*.*) javari*, *G*. *(T*.*) jonasi*, *G*. *(T*.*) melanopleura*, *G*. *(T*.*) onca*, *G*. *(T*.*) stenoleucus*.

### Diagnosis

Gymnotus (*Tigrinus*) is readily distinguishable from all other subgenera of the Gymnotinae by the following characters: a color pattern consisting of evenly spaced, often unpaired, straight dark pigment bands with high-contrast (sharp) margins (except *G*. *(T*.*) onca* with large dark blotches, equal in width to 10+ scales) vs. wavy dark pigment band pairs in the subgenera *Gymnotus*, *Lamontianus* and *Tigre and* dark spots or blotches in the subgenera *Pantherus* and *Tijax*, dentary with five or more narrow, needle-shaped teeth vs. all conical in the subgenera *Lamontianus* (except *G*. *(L*.*) tiquie*), *Pantherus*, *Tigre* and *Tijax* and some arrowhead-shaped in the subgenus *Gymnotus*, very narrow cleithrum with a straight ventral margin vs. narrow with a straight ventral margin in the subgenera *Lamontianus* and *Pantherus* and broad with a curved ventral margin in the subgenera *Gymnotus*, *Tigre* and *Tijax*, more than 18 unbranched anal-fin rays vs.10–17 in other subgenera. *Gymnotus* (*Tigrinus*) is morphologically most similar to *Gymnotus* (*Lamontianus*), from which is it distinguishable by the following characters: fewer anal-fin rays (AFR 135–245) vs. more (AFR 210–312), few, large anal-fin pterygiophore (APS) scales (5–7) vs. many small APS scales (9–10), end of maxilla paddle shaped (broad distally) vs. rod-shaped (narrow distally).

### Description

Sexually monomorphic (except in *G*. *(T*.*) javari*). Size up to 220 mm TL with adult body proportions attained at about 125 mm TL. Adult body shape subcylindrical with a mean ratio of body width to depth of 69%. Body profile slender, body depth 55.7–94.4% total length. Head length moderate, 7.8–11.7% total length. Snout length moderate, 28.2–37.7% head length. Mouth width narrow, 27.8–46.6% head length. Preanal distance long, 63.7–124.4%head length. Anal-fin long, 62.0–84.8% total length. Cycloid or ovoid scales present on entire post-cranial portion of body from nape to caudal appendage.

Scales above lateral line of intermediate size, in 6–9 rows. Scales over anal-fin pterygiophores large, with 5–7 rows. Gape large, extending to or beyond posterior nares. Mouth position superior, lower jaw longer than upper, rictus decurved. Chin round in lateral, dorsal profiles, fleshy and bulbous with mental electroreceptive organ overlying lower jaw. Anterior narial pore partially or entirely included within gape, in small narial fold. Anterior nares small, its diameter less than that of eye. Eye below horizontal with mouth. Circumorbital series ovoid. Premaxilla with few to more than 11 teeth (except in *G*. *(T*.*) jonasi*, *G*. *(T*.*) melanopleura* and *G*. *(T*.*) onca*) disposed in two rows along outer margin, median margin curved. Maxilla-palatine articulation near tip of endopterygoid. Maxilla rod- or paddle-shaped, broad distally with a straight ventral margin, length equal to 7–9 dentary teeth (except in *G*. *(T*.*) jonasi*, *G*. *(T*.*) melanopleura* and *G*. *(T*.*) onca* where length is equal to 4–6 dentary teeth). Dentary with one row of 16 or more needle-shaped teeth (except in *G*. *(T*.*) jonasi*, *G*. *(T*.*) melanopleura* and *G*. *(T*.*) onca* which possess 12 or fewer dentary teeth). Posterodorsal and posteroventral dentary processes abuts ventral. Dentary posteroventral process shorter than or almost as long as posterodorsal, narrow distally. Dentary ventral margin lamella small, depth less than posterior process (except in *G*. *(T*.*) jonasi*, *G*. *(T*.*) melanopleura* and *G*. *(T*.*) onca*). Dentary anteroventral margin hook present in lateral view (except in *G*. *(T*.*) coropinae* and *G*. *(T*.*) stenoleucus*). Mandible long and extended. Anguloarticular process short, extending to ventral margin of dentary (except in *G*. *(T*.*) coropinae* and *G*. *(T*.*) stenoleucus*, where it is long and extends beyond the ventral margin of the dentary). Retroarticular with an arched lamella posteriorly forming a small canal, posterior margin squared. Endopterygoid superior and inferior portions approximately equal in size, ascending process robust, long, straight, tip simple. Interopercle dorsal margin ascending process present. Dorsal region of hyomandibula with four lateral foramenae, supraorbital and infraorbital nerves connected. Preopercle anteroventral notch present, posterodorsal laterosensory ramus with one superficial pore, margin of medial shelf entirely smooth, median shelf small, less than half width of symplectic. Opercle dorsal margin straight or convex, posterior margin entirely smooth. Subopercle dorsal margin concave. Cranial fontanels closed in juveniles and adults. Frontal broad, anterior margin rounded (except in *G*. *(T*.*) coatesi* and *G*. *(T*.*) javari*), postorbital process narrow, less than two times width of supraorbital canal. Lateral ethmoid unossified. Parietal rectangular, length equal to width. Pterosphenoid anteroventral portion robust or reduced, extends to lateral margin of parasphenoid. Parasphenoid posterior processes robust or gracile. Prootic foramen Vp combined with V2-3+VII. *M*. *adductor mandibula* intermusculars absent. All basibranchials unossified. Gill rakers not contacting gill bar.

Cleithrum very narrow with straight ventral margin, anterior limb long, greater than 1.8 times ascending limb, lacking large facet for insertion of muscle from supracleithrum. Postcleithrum thin, discoid or sickle shaped. Body cavity of intermediate length, with 35–44 precaudal vertebrae. Rib 5 broad with triangular medial shelf, greater than three times width of rib 6. Displaced hemal spines absent. Pectoral fin variable, with 12–20 rays. Anal fin of intermediate length, with 135–245 rays. Lateral-line dorsal rami absent in adults. Single hypaxial electric organ, extending along entire ventral margin of body with 3 rows of electroplates near caudal insertion of anal fin.

### Etymology

Subgenus name derived from the Northern Tiger Cat, *Leopardus tigrinus* (Schreber 1775), a relatively smaller and distinctly-colored species of Felidae. Introducing this name, in combination with the subgenera *Tigre* and *Pantherus*, continues the convention of naming gymnotids for felids, which are often similarly nocturnal, predatory and banded or spotted.

**Diagnoses and descriptions of each species of *Tigrinus* (Table 16)**

## *Gymnotus (Tigrinus) coatesi* LaMonte

### Materials examined in morphological analyses

AMNH 12624 (Holotype), 180 mm, Rio Amazonas, Amazonas; MCP 34471, 81 mm, Brazil, Amazonas, Tefé, Rio Tefé, Lago Tefé, Igarape do Xidarini, stream southeast of city of Tefe (03°22’S, 64°40’W); MCP 34472, 83 mm, same locality as MCP 34471; MCP 34473, 100 mm, same locality as MCP 34471; MCP 34474, 166 mm, same locality as MCP 34471; MCP 34475, 74 mm, same locality as MCP 34471; MCP 34838 (2), 98–144 mm; Peru, Loreto, Maynas, Rio Nanay, stream ~26 km south of Iquitos (03°56.63’S, 73°23.90’W); MCP 34839 (3), 47–72 mm, Peru, Loreto, Sapuena, Rio Ucayali, stream ~2 km north of Instituto de Investigaciones de la Amazonia Peruana field station (04°52’S, 073°38’); MCP 34840, 192 mm, same locality as MCP 34471; MCP 34841 (5), 141–196 mm, same locality as MCP 34471; MUSM 20681 (2), 126–190 mm, same locality as MCP 34838; MUSM 20682 (8), 36–133 mm, Peru, Loreto, Rio Ucayali, stream ~2.5 km north of IIAP field station (04°52.70’S, 73°38.85’W); MUSM 20683, 121 mm, Peru, Loreto, Rio Ucayali, stream ~3 km south of Jenaro Herrera (04°55.63’S, 73°39.23’W); UF 137570 (2), 145–167 mm, same locality as MCP 34838; UF 137571, 32 mm, same locality as MCP 34838; UF 137572 (2), 135–186 mm, Peru, Loreto, Rio Ucayali, tributary of Quebrada Parnayari, ~2 km south of town of Jenaro Herrera (04°55.10’S, 73°39.20’W).

**Table 16:** Summary of morphometric and meristic data for seven species of *Tigrinus*. Data for 121 specimens.

*** ***	***G*. *(T*.*) coatesi***				***G*. *(T*.*) coropinae***				***G*. *(T*.*) javari***				***G*. *(T*.*) jonasi***				***G*. *(T*.*) melanopleura***				***G*. *(T*.*) onca***				***G*. *(T*.*) stenoleucus***			
** **	**N**	**Min**	**Max**	**AVG**	**N**	**Min**	**Max**	**AVG**	**N**	**Min**	**Max**	**AVG**	**N**	**Min**	**Max**	**AVG**	**N**	**Min**	**Max**	**AVG**	**N**	**Min**	**Max**	**AVG**	**N**	**Min**	**Max**	**AVG**
**TL**	6	81	180	113	60	82	160	117	35	80	220	152	10	80	115	100	1	99	99	99	1	116	116	116	9	84	142	115
**HL**	4	9.4	18	11.8	52	7	14.4	11	34	8.8	27.5	14.7	9	9	12.1	10.4	1	11.6	11.6	11.6	1	10.2	10.2	10.2	9	9.2	15.2	12
**HL%**	4	10	11.7	10.9	49	7.9	11.2	9.2	28	7.8	11.1	9.4	9	9.6	11.3	10.6	1	11.7	11.7	11.7	1	8.8	8.8	8.8	8	9.7	11.1	10.7
**PR%**	4	30.9	37.2	34.8	40	29.3	37.7	33.7	31	30.2	36.9	33.2	8	28.2	29.3	28.7	1	32.8	32.8	32.8	1	30.4	30.4	30.4	3	29.3	30.8	29.8
**MW%**	4	27.8	35.1	31.5	41	34.6	46.6	40.8	32	31.1	43.2	38.5	9	29.2	33.3	31.5	1	38.8	38.8	38.8	1	41.2	41.2	41.2	3	30.4	37.7	34.8
**PO%**	3	58.5	60	59.1	40	59.7	67.1	63.4	33	56.4	66.9	61	9	62	63.7	62.8	1	61.2	61.2	61.2	1	65.7	65.7	65.7	3	61	64.1	62.6
**IO%**	4	33.3	36.5	35.5	35	36.9	45.6	40.9	32	31.5	41.6	38	9	36.3	38.5	37.4	1	38.8	38.8	38.8	1	39.2	39.2	39.2	3	30.4	32.2	31.5
**BD%**	4	56.8	71.2	63.8	48	64.5	87.9	76.6	25	55.7	89.1	74	9	64.4	80.7	71.5	1	90.5	90.5	90.5	1	87.3	87.3	87.3	8	69.1	82.2	76
**BW%**	4	48.4	55.6	52.5	35	40.8	61.3	53.1	14	37.1	76.7	57.2	9	40	50.9	46.5	1	52.6	52.6	52.6	1	56.9	56.9	56.9	8	40.9	51.3	47.2
**BW/BD**	4	75.7	86	82.5	36	51.4	82.9	69.7	15	60.9	79.4	70.2	9	57.9	74.7	65.2	1	58.1	58.1	58.1	1	65.2	65.2	65.2	9	57.7	65.5	62.2
**HD%**	3	51.1	57.7	53.4	37	52.7	65.3	58.1	27	50.3	65.5	58.3	8	51.6	60.6	56.9	1	69.8	69.8	69.8	1	65.7	65.7	65.7	3	56.8	60.8	58.4
**HW%**	4	49.5	55.8	52.7	38	53.8	73.3	61.3	24	51.1	67.1	61.4	9	56.7	63.6	60.2	1	56.9	56.9	56.9	1	58.8	58.8	58.8	3	53.2	58.7	55.7
**BO%**	_	_	_	_	29	20.8	40.8	30.8	15	26.9	34	32	_	_	_	_	1	31.9	31.9	31.9	1	30.4	30.4	30.4	2	32.1	40.8	36.5
**PA%**	3	80.9	83.7	81.9	41	81	123.8	101.1	15	70.6	120.9	89.6	8	72.2	90.8	84.3	1	72.4	72.4	72.4	1	63.7	63.7	63.7	3	77.2	78.7	78.1
**P1%**	4	41.7	50	46.3	39	37.4	54.8	45.5	29	40.2	56.6	46.6	9	50	56.9	53.3	1	43.1	43.1	43.1	1	50	50	50	7	29.1	51.8	37.5
**AF%**	3	77.8	81	79.4	39	71.4	84.8	79.3	29	70.8	81.1	76.5	9	62	80	72.5	1	74.7	74.7	74.7	1	72.4	72.4	72.4	1	69.9	69.9	69.9
	**N**	**Min**	**Max**	**Mode**	**N**	**Min**	**Max**	**Mode**	**N**	**Min**	**Max**	**Mode**	**N**	**Min**	**Max**	**Mode**	**N**	**Min**	**Max**	**Mode**	**N**	**Min**	**Max**	**Mode**	**N**	**Min**	**Max**	**Mode**
**BND**	4	15	17	16	35	12	20	16	12	13	21	18	9	11	20	15	1	11	11	12	_	_	_	_	9	18	24	21
**AFR**	4	214	223	217	29	183	245	214	13	180	240	210	6	135	165	160	1	160	160	160	1	180	180	180	8	190	245	219
**P1R**	3	15	15	15	45	12	14	13	13	13	20	14	_	_	_	_	1	14	14	14	1	13	13	13	8	12	14	13
**SAL**	3	6	8	6	50	6	8	7	13	7	9	8	_	_	_	_	1	8	8	8	1	8	8	8	9	8	9	9
**CEP**	1	3	3	3	33	3	3	3	10	3	3	3	6	3	3	3	1	3	3	3	_	_	_	_	4	3	3	3
**APS**	3	6	7	6	6	5	6	6	_	_	_	_	6	5	5	5	_	_	_	_	_	_	_	_	_	_	_	_
**PCV**	8	36	43	43	40	39	43	41	16	40	44	42	9	36	39	37	1	36	36	36	1	35	35	35	8	41	43	42
**PLR**	3	61	65	65	36	46	64	56	12	52	67	60	6	31	40	36	1	46	46	46	1	44	44	44	4	50	59	55
**PLL**	3	85	102	98	11	82	114	101	_	_	_	_	_	_	_	_	1	69	69	69	1	80	80	80	_	_	_	_
**VLR**	_	_	_	_	_	_	_	_	_	_	_	_	_	_	_	_	_	_	_	_	_	_	_	_	_	_	_	_

### Diagnosis

*Gymnotus* (*Tigrinus*) *coatesi* can be differentiated from all other members of the subgenus on the basis of the following characters: color pattern with 15–17 unpaired dark bands, pale interbands wider dorsally on anterior 50% of body, up to 50% width of dark band pairs, or 25% in ventral portion of same band (vs. 12–20 wide (>5X width of interbands) dark bands in *G*. *(T*.*) coropinae*, 13–21 paired dark bands in *G*. *(T*.*) javari*, 11–20 paired or unpaired dark bands in *G*. *(T*.*) jonasi*, 11 unpaired dark bands in *G*. *(T*.*) melanopleura*, dark band pairs replaced by dark brown blotches in *G*. *(T*.*) onca*, 18–24 paired dark bands in *G*. *(T*.*) stenoleucus*).

### Description

Sexually monomorphic. Size up to 180 mm TL with adult body proportions attained at about 150 mm TL. Adult body shape subcylindrical with a mean ratio of body width to depth of 82.5%. Body profile slender, body depth 56.8–71.2% total length. Head length intermediate, 10.0–11.7% total length. Snout length moderate, 30.9–37.2% head length. Mouth width narrow, 27.8–35.1% head length. Preanal distance long, 80.9–83.7% head length. Anal-fin long, 77.8–81.0% total length. Cycloid or ovoid scales present on entire post-cranial portion of body from nape to caudal appendage.

Scales above lateral line intermediate, in 6–8 rows. Scales over anal-fin pterygiophores large, with 6–7 rows. Gape large, extending to or beyond posterior nares. Mouth position superior, lower jaw longer than upper, rictus decurved. Chin round in lateral, dorsal profiles, fleshy and bulbous with mental electroreceptive organ overlying lower jaw. Anterior narial pore partially or entirely included within gape, in small narial fold. Anterior nares small, its diameter less than that of eye. Eye below horizontal with mouth. Circumorbital series ovoid. Premaxilla with >11 teeth disposed in two rows along outer margin, median margin curved. Maxilla-palatine articulation near tip of endopterygoid. Maxilla rod- or paddle-shaped, narrow distally with a straight ventral margin, length equal to 7–9 dentary teeth. Dentary with one row of >16 teeth, >5 needle-shaped anteriorly, all others conical posteriorly. Posterodorsal and posteroventral dentary processes abuts ventral. Dentary posteroventral process shorter than or almost as long as posterodorsal, narrow distally. Dentary ventral margin lamella small, depth less than posterior process. Dentary anteroventral margin with a hook. Anguloarticular process short, extending to ventral margin of dentary. Retroarticular with an arched lamella posteriorly forming a small canal, posterior margin squared. Endopterygoid superior and inferior portions approximately equal in size, ascending process robust, straight, tip simple. Interopercle dorsal margin ascending process present. Dorsal region of hyomandibula with four lateral foramenae, supraorbital and infraorbital nerves connected. Preopercle anteroventral notch present, posterodorsal laterosensory ramus with 1 superficial pore, margin of medial shelf smooth, median shelf small, less than half width of symplectic. Opercle dorsal margin straight, posterior margin smooth. Cranial fontanels closed in juveniles and adults. Frontal anterior margin straight, postorbital process narrow, less than two times width of supraorbital canal. Lateral ethmoid unossified. Parietal rectangular, length equal to width. Pterophenoid anteroventral process robust, extends to lateral process. Parasphenoid posterior processes robust. Prootic foramen Vp combined with V2-3+VII. Adductor mandibula insertion undivided, intermusculars absent. All basibranchials unossified. Gill rakers not contacting gill bar.

Cleithrum narrow with straight ventral margin, anterior limb long, greater than 1.8 times ascending limb, without facet for insertion of muscle from supracleithrum. Postcleithrum thin, discoid or sickle shaped. Body cavity variable, with 36–43 precaudal vertebrae. Rib 5 broad along its entire extent, greater than three times width of rib 6. Displaced hemal spines absent. Pectoral fin intermediate, with 15 rays. Anal fin long, with 214–223 rays. Lateral-line complete. Lateral-line dorsal rami absent in adults. Single hypaxial electric organ, extending along entire ventral margin of body with 3 rows of electroplates near caudal insertion of anal fin.

## *Gymnotus (Tigrinus) coropinae* Hoedeman

### Materials examined in morphological analyses

ZMA 100.185 (Holotype), 49 mm, Surinam, Commewijne, Coropina Creek (05°32’N, 55°10’W); FMNH 106694, 68 mm, Bolivia, Pando, Río Abuna, Río Nareuda (~11°18’S, 68°46’W); UF 127275, 141 mm, Brazil, Amazonas, Barcelos, Rio Demini (00º16’S, 62º46’W); UF 127323, 127 mm, Brazil, Amazonas; INPA 9811, 73 mm, Brazil, Amazonas, Itacoatiara, Rio Preta da Eva, Manaus-Itacoatiara km 15 (02º45’S, 59º35’W); INPA 16004, 82 mm, Brazil, Amazonas, Manaus, Rio Negro, Rio Tarumã-Açu (03º00’S, 60º04’W); INPA 13427 (3), 54–67 mm, Brazil, Amazonas, Rio Negro, Igarapé Tarumã-Mirim (03°02’S, 60°09’W); INPA 13428 (3) 60–115 mm, Brazil, Amazonas; INPA 14993 (3) 41–58 mm, Brazil, Amazonas; INPA 9739, 65 mm, Brazil, Amazonas, Novo Airão, Rio Negro, Rio Jaú, Igarapé Miratuca (~01º54’S, 61º26’W); INPA 9743, 58 mm, Brazil, Amazonas, Rio Jaú, Rio Carabinani (01º58’S, 61º31’W); INPA 9807, 126 mm, Brazil, Amazonas, Presidente Figueiredo, Rio Uatamã (~01º52’S, 60º08’W); INPA 14214 (3), 46–76 mm, Brazil, Amazonas, Rio Urubú (~01º16’S, 59º49’W); INPA 14227 (3), 41–55 mm, same locality as INPA 14214; INPA 14258 (2), 81–86 mm, same locality as INPA 14214; INPA 6594, 84 mm, Brazil, Amazonas, Santa Isabel do Rio Negro, Rio Negro, Igarapé Santo Antônio (01º60’S, 67º14’W); INPA 9131, 60 mm, Brazil, Amazonas, São Gabriel da Cachoeira, Rio Negro (~00°07’S, 67°05’W); BMNH 1998.3.12.211, 52 mm, Brazil, Amazonas, Tefé, Rio Tefé, Lago Tefé, Igarapé Curupira (03º26’01”S, 64º43’47”W); BMNH 1998.3.12.212, 93 mm, Brazil, Amazonas; BMNH 1998.3.12.213–214 (2), 84–87 mm, Brazil, Amazonas; BMNH 1998.3.12.215, 84 mm, Brazil, Amazonas; BMNH 1998.3.12.216, 76 mm, Brazil, Amazonas; BMNH 1998.3.12.217, 90 mm, Brazil, Amazonas; IDSM 426 (8), 55–73 mm, Brazil, Amazonas; INPA 9964, 92 mm, Brazil, Amazonas; INPA 9965A, 74 mm, Brazil, Amazonas; INPA 15831 (4), 74–109 mm, Brazil, Amazonas; INPA 18182 (3), 87–100 mm, Brazil, Amazonas; INPA 18385, 49 mm, Brazil, Amazonas; MZUSP 60610, 145 mm, Brazil, Amazonas; MZUSP 60611 (4), 113–122 mm, Brazil, Amazonas; MZUSP 60612 (2), 112–123 mm, Brazil, Amazonas; MZUSP 75188, 113 mm, Brazil, Amazonas; INPA 18184 (4), 87–107 mm, Brazil, Amazonas, Rio Tefé, Lago Tefé, Igarapé Curupira, swamp pools (03º26’01”S, 64º43’52”W); INPA 18185 (5), 98–112 mm, Brazil, Amazonas; MCP 30679 (3), 80–93 mm, Brazil, Amazonas; MZUSP 60613, 101 mm, Brazil, Amazonas; MZUSP 75181, 132 mm, Brazil, Amazonas; MZUSP 75182, 100 mm, Brazil, Amazonas); MZUSP 75183, 112 mm, Brazil, Amazonas; MZUSP 75184, 106 mm, Brazil, Amazonas; MZUSP 75185, 156 mm, Brazil, Amazonas; MZUSP 75186, 137 mm, Brazil, Amazonas; MZUSP 75187, 125 mm, Brazil, Amazonas; INPA 9965B (2), 87–98 mm, Brazil, Amazonas, Rio Tefé, Lago Tefé, Igarapé Repartimento (03º24’28”S, 64º44’10”W); INPA 18181, 117 mm, Brazil, Amazonas; INPA 18183 (3), 79–97 mm, Brazil, Amazonas; INPA 18186 (3), 28–105 mm, Brazil, Amazonas; INPA 18187 (12), 47–127 mm, Brazil, Amazonas; INPA 18384, 85 mm, Brazil, Amazonas; INPA 18386 (3), 52–128 mm, Brazil, Amazonas; INPA 18387, 45 mm, Brazil, Amazonas; MCP 30673, 90 mm, Brazil, Amazonas; MCP 30674 (2), 95–102 mm, Brazil, Amazonas; MCP 30675 (6), 70–122 mm, Brazil, Amazonas; MCP 30676 (6), 83–98 mm, Brazil, Amazonas; MCP 30677 (3), 75–128 mm, Brazil, Amazonas; MCP 30678 (2), 54–77 mm, Brazil, Amazonas; MCP 30680 (2), 94–109 mm, Brazil, Amazonas; MCP 30681, 97 mm, Brazil, Amazonas; MCP 30682 (2), 112–118 mm, Brazil, Amazonas; UF 118840, 97 mm, Brazil, Amazonas; INPA 9806, 84 mm, Brazil, Amazonas, Pará: Oriximinã, Rio Trombetas, Rio Mapuera, Igarapé do Patauá (~01°05’S, 57°02’W); INPA 9809 (21), 51–126 mm, Brazil, Amazonas; INPA 9819, 39 mm, Brazil, Amazonas, Rio Trombetas, Igarapé Porteiro, Cachoeira Porteiro (~01°05’S, 57°01’W); INPA 1153 (2), 36–41 mm, Brazil, Amazonas, Rondônia, Ariquemes, Rio Madeira, Bacia do Igarapé Agua Azul (09°46’S, 62°22’W); INPA 9839 (4), 55–130 mm, Brazil, Amazonas, Porto Velho, Rio Madeira, Rio Jamari, Igarapé Jatuarana (08°45’S, 63°28’W); INPA 9841 (2), 115–116 mm, Brazil, Amazonas; CAS 167969, 108 mm, Colombia, Vichada, Río Orinoco, Río Guaviare, Río Guayabero (~05º54’N, 68º28’W); UF 19471, 112 mm, Colombia, Vichada, Río Orinoco, Río Meta, RíoYucao (04°19’N, 02°04’W); UF 33470, 87 mm, Colombia, Vichada; FMNH 103352 (3), 19–155 mm, Ecuador, Napo, Río Payamino (00º30’S, 77º15’W); ANSP 177445, 50 mm, Guyana, Demerara, Essequibo River (04º45’N, 58º42’W); FMNH 97300, 124 mm, Guyana, Demerara, Essequibo River, Kumaka (05°38’N, 57°52’W); USNM (3), 35–118 mm, Guyana, Demerara, Rupunini, Moco-Moco creek, (03°21’N, 59°47’59W); USNM, 157 mm, Guyana, Demerara; ANSP 17746, 107 mm, Guyana, Demerara, Siparuni, Siparuni River (04º48’N, 58º51’W); ANSP 177444, 170 mm, Guyana, Demerara, Tiger Creek, 3 km Kurupukari (04º38’N, 58º43’W); ANSP 177444, 170 mm, Guyana, Demerara, Dog Falls; ANSP 175947 (2), 88–93 mm, Guyana, Demerara, Turtle Pond; ANSP 175949, 126 mm, Guyana, Demerara; ANSP 17591, 131 mm, Guyana, Demerara, near Kurupukari; ANSP 175950, 63 mm, Guyana, Demerara, near Burro Burro; FMNH 96980, 98 mm, Peru, Amazonas, Río Marañon, Río Santiago, Quebrada Caterpiza (03°50’S, 77°42’W); MUSM 17596 (3), 90–112 mm, Peru, Madre de Dios, Río Madre de Dios (12°36’S, 69°11’W); MUSM 20146, 76 mm, Peru, Madre de Dios, Río Madre de Dios, Parque Nacio-nal Manu, Río de los Amigos (12°34’36”S, 70°04’14”W); MUSM 4176, 81 mm, Peru, Madre de Dios, Río Madre de Dios, Río Tambopata (~12°44’S, 69°11’W); MUSM 4503 (2), 66–70 mm, Peru, Madre de Dios, Río Tambopata, cuenca del Río Heath, Quebrada San Antonio (~12°44’S, 69°11’W); MUSM 3013, 105 mm, Peru, Madre de Dios, Río Tambopata, Cochachica (~12°44’S, 69°11’W); MUSM 535 (13), 79–136, Peru, Madre de Dios; USNM 264102 (2), 112–114 mm, Peru, Madre de Dios, Río Tambopata, near Cochachica (12°50’30”S, 69°17’31”W); USNM 264108 (2), 117–121 mm, Peru, Madre de Dios, Río Tambopata, near Río la Torre (12°49’40”S, 69°18’00”W); USNM 366207 (3), 86–142 mm, Peru, Madre de Dios, Río Tambopata, near Cochachica (12°49’45”S, 69°16’15”W); USNM 366208, 97 mm, Peru, Madre de Dios, Río Tambopata, near Río la Torre (12°50’S, 69°18’W); AMNH 54843 (4), 63–94 mm, Suriname, Commewijne, Nickerie, stream near Devis Falls, near Avanavero (04°50’N, 57°14’W); FMNH 84584 (3), 127–160 mm, Suriname, Commewijne, Corantijn River; AMNH 54888 (2), 65–79 mm, Suriname, Commewijne, Mataway Creek; AMNH 54758 (2), 71–112 mm, Suriname, Commewijne, Kapoeri Creek (05°16’N, 57°13’W); AMNH 54875, 46 mm, Suriname, Commewijne, Toeboeroe Creek (05°00’N, 57°31’W); AMNH 55000 (2), 58–96 mm, Suriname, Commewijne, Lucie River, Paramaribo River Road, km 212; AMNH 5496, 73 mm, Suriname, Commewijne, Corantijn River, stream south of Tiger Falls; USNM 225272 in part (8), 86–127 mm, Suriname, Commewijne, Corantijn River; USNM 225263 (2), 44–51 mm, Suriname, Commewijne, Lucie River ~(03°35’N, 57°40’W); USNM 225264 (9), 58–87 mm, Suriname, Commewijne, Lana Creek (05°26’N, 57°15’00"W); USNM 225265 (2), 91–113 mm, Suriname, Commewijne, near Mataway (~05°01’N, 55°42’W); USNM 225277 (3), 72–93 mm, Suriname, Commewijne, Lucie River (~03°35’N, 57°40’W); USNM 225278 (19), 33–138 mm, Suriname, Commewijne, near Cow Falls (~05°00’N, 57°38’W); USNM 225279 (4), 52–81 mm, Suriname, Commewijne, near Amotopo, (~05°33’N, 57°38’W); USNM 225281 (2), 54–67 mm, Suriname, Commewijne, near Mataway (~05°01’N, 55°42’W); USNM 225282 (10), 44–68 mm, Suriname, Commewijne, Dalibane Creek (~05°34’N, 57°10’W); USNM 225287 (25), 47–114 mm, Suriname, Commewijne, near Tiger Falls (~05°16’N, 58°57’W); USNM 225298, 67 mm, Suriname, Commewijne, near Dalibane Creek (~05°34’N, 57°10’W); ANSP 139849 (8), 97–144 mm, Venezuela, Bolivar, Río Mato (07º08’N, 65º10’W); UF 97641 (3), 131–132 mm, Venezuela, Bolivar, Río Caura (06º38’N, 64º37’W).

### Diagnosis

*Gymnotus* (*Tigrinus*) *coropinae* can be differentiated from all other members of the subgenus on the basis of the following characters: color pattern with 12–20 wide (>5X width of interbands) dark bands, where pale interbands restricted to the ventral part of the lateral surface such that the dark interbands fuse into a uniform dark coloration over anterior 60% of body (vs. 15–17 unpaired dark bands in *G*. *(T*.*) coatesi*, 13–21 paired dark bands in *G*. *(T*.*) javari*, 11–20 paired or unpaired dark bands in *G*. *(T*.*) jonasi*, 11 unpaired dark bands in *G*. *(T*.*) melanopleura*, dark band pairs replaced by dark brown blotches in *G*. *(T*.*) onca*, 18–24 paired dark bands in *G*. *(T*.*) stenoleucus*).

### Description

Sexually monomorphic. Size up to 160 mm TL with adult body proportions attained at about 150 mm TL. Adult body shape subcylindrical with a mean ratio of body width to depth of 69.7%. Body profile slender, body depth 64.5–87.9% total length. Head length short, 7.9–11.2% total length. Snout length moderate, 29.3–37.7% head length. Mouth width narrow, 34.6–46.6% head length. Preanal distance long, 81.0–123.8% head length. Anal-fin long, 71.4–84.8% total length. Cycloid or ovoid scales present on entire post-cranial portion of body from nape to caudal appendage.

Scales above lateral line intermediate, in 6–8 rows. Scales over anal-fin pterygiophores large, with 5–6 rows. Gape large, extending to or beyond posterior nares. Mouth position superior, lower jaw longer than upper, rictus decurved. Chin round in lateral, dorsal profiles, fleshy and bulbous with mental electroreceptive organ overlying lower jaw. Anterior narial pore partially or entirely included within gape, in small narial fold. Anterior nares small, its diameter less than that of eye. Eye below horizontal with mouth. Circumorbital series ovoid. Premaxilla with >11 teeth disposed in two rows along outer margin, median margin curved. Maxilla-palatine articulation near tip of endopterygoid. Maxilla rod- or paddle-shaped, narrow distally with a straight ventral margin, length equal to 7–9 dentary teeth. Dentary with one row of >16 teeth, >5 needle-shaped anteriorly, all others conical posteriorly. Posterodorsal and posteroventral dentary processes abuts ventral. Dentary posteroventral process shorter than or almost as long as posterodorsal, narrow distally. Anguloarticular process long, extending beyond ventral margin of dentary. Retroarticular with an arched lamella posteriorly forming a small canal, posterior margin squared. Endopterygoid superior and inferior portions approximately equal in size, ascending process robust, straight, tip simple. Interopercle dorsal margin ascending process present. Dorsal region of hyomandibula with four lateral foramenae. Preopercle anteroventral notch present, posterodorsal laterosensory ramus with 1 superficial pore, margin of medial shelf smooth, median shelf small, less than half width of symplectic. Opercle dorsal margin straight, posterior margin smooth. Cranial fontanels closed in juveniles and adults. Frontal anterior margin rounded, postorbital process narrow, less than two times width of supraorbital canal. Lateral ethmoid unossified. Parietal rectangular, length equal to width. Adductor mandibula insertion undivided, intermusculars absent. All basibranchials unossified. Gill rakers not contacting gill bar.

Cleithrum very narrow with straight ventral margin, anterior limb long, greater than 1.8 times ascending limb, with facet for insertion of muscle from supracleithrum. Postcleithrum thin, discoid or sickle shaped. Body cavity long, with 39–43 precaudal vertebrae. Rib 5 broad along its entire extent, greater than three times width of rib 6. Displaced hemal spines absent. Pectoral fin narrow, with 12–14 rays. Anal fin long, with 183–245 rays. Lateral-line complete. Lateral-line dorsal rami absent in adults. Single hypaxial electric organ, extending along entire ventral margin of body with 3 rows of electroplates near caudal insertion of anal fin.

## *Gymnotus (Tigrinus) javari* Albert & Crampton

### Materials examined in morphological analyses

UMMZ 224599 (Holotype), 197 mm, Peru, Loreto, Rio Javarí, Quebrada Caraná near Buen Sucesso (04°22’S, 70°31’W); UMMZ 224596 (Paratypes) (10), 45–175 mm, same locality as UMMZ 224599; UMMZ 240971 (Paratype), 162 mm, same locality as UMMZ 224599; MCZ 60005 (8), 70–208 mm, Brazil, Amazonas, Rio Solimões at Tabatinga (04°16’S, 69°56’W); MUSM 14480b, 85 mm, Ecuador, Napo, Río Napo drainage at Río Aguarico (01°00’S, 75°12’W); MUSM 14481 (8), 72–124 mm, same locality as MUSM 14480b; MUSM 3234 (2), 51–102 mm, Peru, Loreto, Río Ucayali at Jenaro Herrera (05°03’S, 73°50’W); NRM 13521, 93 mm, Peru, Loreto, Río Ampiyacu near Pebas in floating meadows (03°20’S, 71°49’W); NRM 27702 (2), 47–51 mm, Peru, Loreto, Río Yavari at stream nr. Colonia Angamos (05°11’S, 72°53’W); UF 122824, 141 mm, Peru, Loreto, near Iquitos (03°46’S, 73°15’W); UMMZ 224601, 132 mm, Peru, Loreto, Río Tahwayo near Santa Ana (04°5’S, 73°00’W); UMMZ 224607 in part (5), 29–106 mm, Peru, Loreto, Río Momon, near Iquitos, Bora Indian village near Amazon camp (03°46’S, 73°15’W).

### Diagnosis

*Gymnotus* (*Tigrinus*) *javari* can be differentiated from all other members of the subgenus on the basis of the following characters: color pattern with 13–21 paired dark bands, narrow pale interbands (25% width of dark band pairs) anteriorly and 5–8 partially divided, often “H”-shaped dark band pairs posteriorly (vs. 15–17 unpaired dark bands in *G*. *(T*.*) coatesi*, 12–20 wide (>5X width of interbands) dark bands in *G*. *(T*.*) coropinae*, 11–20 paired or unpaired dark bands in *G*. *(T*.*) jonasi*, 11 unpaired dark bands in *G*. *(T*.*) melanopleura*, dark band pairs replaced by dark brown blotches in *G*. *(T*.*) onca*, 18–24 paired dark bands in *G*. *(T*.*) stenoleucus*), many scales above lateral line at midbody except when compared to *G*. *(T*.*) stenoleucus* (SAL 8–9 vs. 6–8 *G*. *(T*.*) coatesi*, 6–8 in *G*. *(T*.*) coropinae*).

### Description

Sexually dimorphic, with adult males longer than females [74]. Size up to 220 mm TL with adult body proportions attained at about 150 mm TL. Adult body shape subcylindrical with a mean ratio of body width to depth of 70.2%. Body profile slender, body depth 55.7–89.1% total length. Head length short, 7.8–11.1% total length. Snout length moderate, 30.2–36.9% head length. Mouth width narrow, 31.1–43.2% head length. Preanal distance long, 70.6–120.9% head length. Anal-fin long, 70.8–81.1% total length. Cycloid or ovoid scales present on entire post-cranial portion of body from nape to caudal appendage.

Scales above lateral line intermediate, in 7–9 rows. Gape large, extending to or beyond posterior nares. Mouth position superior, lower jaw longer than upper, rictus decurved. Chin round in lateral, dorsal profiles, fleshy and bulbous with mental electroreceptive organ overlying lower jaw. Anterior narial pore partially or entirely included within gape, in small narial fold. Anterior nares small, its diameter less than that of eye. Eye below horizontal with mouth. Circumorbital series ovoid. Premaxilla with >11 teeth disposed in two rows along outer margin, median margin curved. Maxilla-palatine articulation near tip of endopterygoid. Maxilla rod- or paddle-shaped, broad distally, length equal to 7–9 dentary teeth. Dentary with one row of >16 teeth, >5 needle-shaped anteriorly, all others conical posteriorly. Posterodorsal and posteroventral dentary processes abuts ventral. Dentary posteroventral process shorter than or almost as long as posterodorsal, narrow distally. Dentary ventral margin lamella small, depth less than posterior process. Dentary anteroventral margin with a hook. Anguloarticular process short, extending to ventral margin of dentary. Retroarticular with an arched lamella posteriorly forming a small canal, posterior margin squared. Endopterygoid superior and inferior portions approximately equal in size, ascending process robust, straight, tip simple. Interopercle dorsal margin ascending process present. Dorsal region of hyomandibula with four lateral foramenae, supraorbital and infraorbital nerves connected. Preopercle anteroventral notch present, posterodorsal laterosensory ramus with 1 superficial pore, margin of medial shelf smooth, median shelf small, less than half width of symplectic. Opercle dorsal margin straight, posterior margin smooth. Cranial fontanels closed in juveniles and adults. Frontal anterior margin straight, postorbital process narrow, less than two times width of supraorbital canal. Lateral ethmoid unossified. Parietal rectangular, length equal to width. Pterophenoid anteroventral process robust, extends to lateral process. Parasphenoid posterior processes robust. Prootic foramen Vp combined with V2-3+VII. Adductor mandibula insertion undivided, intermusculars absent. All basibranchials unossified. Gill rakers not contacting gill bar.

Cleithrum very narrow with straight ventral margin, anterior limb long, greater than 1.8 times ascending limb, without facet for insertion of muscle from supracleithrum. Postcleithrum thin, discoid or sickle shaped. Body cavity long, with 40–44 precaudal vertebrae. Rib 5 broad along its entire extent, greater than three times width of rib 6. Displaced hemal spines absent. Pectoral fin variable, with 13–20 rays. Anal fin long, with 180–240 rays. Lateral-line complete. Lateral-line dorsal rami absent in adults. Single hypaxial electric organ, extending along entire ventral margin of body with 3 rows of electroplates near caudal insertion of anal fin.

## *Gymnotus (Tigrinus) jonasi* Albert & Crampton

### Materials examined in morphological analyses

INPA 13507 (Holotype), 114 mm, Brazil, Amazonas, cano do lago rato, Mamirauá reserve (03°02’48”S, 64°51’22”W); BMNH1998.3.11 (Paratypes) (23), 39–115 mm, same locality as INPA 13507; BMNH 1998.3.11:116–127 (Paratypes) (12), 93–118 mm, Brazil, Amazonas, Ressaca Vila Alencar, Mamirauá reserve (03°07’49”S, 64°47’58”W); BMNH 1998.3.11:128–134 (Paratypes) (7), 72–100 mm, same locality as INPA 13507; INPA 13508 (Paratypes) (6), 70–99 mm, same locality as INPA 13507; MUSM 14481 (8), Peru, Loreto, Maynas, Rio Aguarico, Napo; MUSM 14490 (2), same locality as MUSM 14481.

### Diagnosis

*Gymnotus* (*Tigrinus*) *jonasi* can be differentiated from all other members of the subgenus on the basis of the following characters: a variable color pattern with 13–20 paired or unpaired dark bands with straight margins over entire body (vs. band margins curved in all other *Tigrinus* except *G*. *(T*.*) melanopleura*, which possesses 11 bands with straight margins).

### Description

Sexually monomorphic. Size up to 115 mm TL with adult body proportions attained at about 150 mm TL. Adult body shape subcylindrical with a mean ratio of body width to depth of 65.2%. Body profile slender, body depth 57.9–74.7% total length. Head length short, 9.6–11.3% total length. Snout length moderate, 28.2–29.3% head length. Mouth width narrow, 29.2–33.3% head length. Preanal distance long, 72.2–90.8% head length. Anal-fin long, 62.0–80.0% total length. Cycloid or ovoid scales present on entire post-cranial portion of body from nape to caudal appendage.

Scales over anal-fin pterygiophores large, with 5 rows. Gape large, extending to or beyond posterior nares. Mouth position superior, lower jaw longer than upper, rictus decurved. Chin round in lateral, dorsal profiles, fleshy and bulbous with mental electroreceptive organ overlying lower jaw. Anterior narial pore partially or entirely included within gape, in small narial fold. Anterior nares small, its diameter less than that of eye. Eye below horizontal with mouth. Circumorbital series ovoid. Premaxilla with <10 teeth disposed in two rows along outer margin, median margin straight. Maxilla-palatine articulation near tip of endopterygoid. Maxilla rod- or paddle-shaped, narrow distally with a straight ventral margin, length equal to 4–6 dentary teeth. Dentary with one row of <12 teeth, >5 needle-shaped anteriorly, all others conical posteriorly. Posterodorsal and posteroventral dentary processes abuts ventral. Dentary posteroventral process shorter than or almost as long as posterodorsal, narrow distally. Dentary ventral margin lamella large, depth greater than posterior process. Dentary anteroventral margin with a hook. Anguloarticular process short, extending to ventral margin of dentary. Retroarticular with an arched lamella posteriorly forming a small canal, posterior margin squared. Endopterygoid superior and inferior portions approximately equal in size, ascending process robust, straight, tip simple. Interopercle dorsal margin ascending process absent. Dorsal region of hyomandibula with four lateral foramenae, supraorbital and infraorbital nerves connected. Preopercle anteroventral notch smooth, posterodorsal laterosensory ramus with 1 superficial pore, margin of medial shelf smooth, median shelf small, less than half width of symplectic. Opercle dorsal margin straight, posterior margin smooth. Cranial fontanels closed in juveniles and adults. Frontal anterior margin rounded, postorbital process narrow, less than two times width of supraorbital canal. Lateral ethmoid unossified. Parietal rectangular, length equal to width. Pterophenoid anteroventral process reduced, extends to lateral process. Parasphenoid posterior processes gracile. Prootic foramen Vp combined with V2-3+VII. Adductor mandibula insertion undivided, intermusculars absent. All basibranchials unossified. Gill rakers not contacting gill bar.

Cleithrum narrow with straight ventral margin, anterior limb long, greater than 1.8 times ascending limb, without facet for insertion of muscle from supracleithrum. Postcleithrum thin, discoid or sickle shaped. Body cavity intermediate, with 36–39 precaudal vertebrae. Rib 5 broad along its entire extent, greater than three times width of rib 6. Displaced hemal spines absent. Anal fin long, with 135–165 rays. Lateral-line complete. Lateral-line dorsal rami absent in adults. Single hypaxial electric organ, extending along entire ventral margin of body with 3 rows of electroplates near caudal insertion of anal fin.

## *Gymnotus (Tigrinus) melanopleura* Albert & Crampton

### Materials examined in morphological analyses

INPA 9966 (Holotype), 99 mm, Brazil, Amazonas, Cano do Lago Rato, Mamirauá Reserve (03°02’36”S, 64°51’02”W).

### Diagnosis

*Gymnotus* (*Tigrinus*) *melanopleura* can be differentiated from all other members of the subgenus on the basis of the following characters: color pattern with 11 unpaired dark bands (vs. 15–17 unpaired dark bands in *G*. *(T*.*) coatesi*, 12–20 wide (>5X width of interbands) dark bands in *G*. *(T*.*) coropinae*, 13–21 paired dark bands in *G*. *(T*.*) javari*, 11–20 paired or unpaired dark bands in *G*. *(T*.*) jonasi*, dark band pairs replaced by dark brown blotches in *G*. *(T*.*) onca*, 18–24 paired dark bands in *G*. *(T*.*) stenoleucus*).

### Description

Sexually monomorphic. Size up to 99 mm TL with adult body proportions attained at about 150 mm TL. Adult body shape subcylindrical with a mean ratio of body width to depth of 58.1%. Body profile slender, body depth 90.5% total length. Head length intermediate, 11.7% total length. Snout length moderate, 32.8% head length. Mouth width narrow, 38.8% head length. Preanal distance long, 72.4% head length. Anal fin long, 74.7% total length. Cycloid or ovoid scales present on entire post-cranial portion of body from nape to caudal appendage.

Scales above lateral line intermediate, in 8 rows. Gape large, extending to or beyond posterior nares. Mouth position superior, lower jaw longer than upper, rictus decurved. Chin round in lateral, dorsal profiles, fleshy and bulbous with mental electroreceptive organ overlying lower jaw. Anterior narial pore partially or entirely included within gape, in small narial fold. Anterior nares small, its diameter less than that of eye. Eye below horizontal with mouth. Circumorbital series ovoid. Premaxilla with <10 teeth disposed in two rows along outer margin. Dentary with one row of <12 teeth. Preopercle posterodorsal laterosensory ramus with 1 superficial pore. Body cavity short, with 36 precaudal vertebrae. Displaced hemal spines absent. Pectoral fin intermediate, with 14 rays. Anal fin intermediate, with 160 rays. Lateral-line complete. Lateral-line dorsal rami absent in adults. Single hypaxial electric organ, extending along entire ventral margin of body with 3 rows of electroplates near caudal insertion of anal fin.

## *Gymnotus (Tigrinus) onca* Albert & Crampton

### Materials examined in morphological analyses

INPA 11512 (Holotype), 116 mm, Brazil, Amazonas, Cano do Lago Rato, Mamirauá Reserve (03°02’36”S, 64°51’02”W).

### Diagnosis

*Gymnotus* (*Tigrinus*) *onca* can be differentiated from all other members of the subgenus on the basis of the following characters: color pattern with dark band pairs replaced by large (>10 scales across), irregular dark brown blotches over entire body (vs. dark bands or band pairs in all other *Tigrinus*).

### Description

Sexually monomorphic. Size up to 116 mm TL with adult body proportions attained at about 150 mm TL. Adult body shape subcylindrical with a mean ratio of body width to depth of 65.2%. Body profile slender, body depth 87.3% total length. Head length short, 8.8% total length. Snout length moderate, 30.4% head length. Mouth width narrow, 41.2% head length. Preanal distance long, 63.7% head length. Anal fin long, 72.4% total length. Cycloid or ovoid scales present on entire post-cranial portion of body from nape to caudal appendage.

Scales above lateral line intermediate, in 8 rows. Gape large, extending to or beyond posterior nares. Mouth position superior, lower jaw longer than upper, rictus decurved. Chin round in lateral, dorsal profiles, fleshy and bulbous with mental electroreceptive organ overlying lower jaw. Anterior narial pore partially or entirely included within gape, in small narial fold. Anterior nares small, its diameter less than that of eye. Eye below horizontal with mouth. Circumorbital series ovoid. Premaxilla with <10 teeth disposed in two rows along outer margin. Dentary with one row of <12 teeth. Body cavity intermediate, with 35 precaudal vertebrae. Pectoral fin narrow, with 13 rays. Anal fin long, with 180 rays. Lateral-line complete. Lateral-line dorsal rami absent in adults. Single hypaxial electric organ, extending along entire ventral margin of body.

## *Gymnotus (Tigrinus) stenoleucus* Mago-Leccia

### Materials examined in morphological analyses

AMNH 59047 (Paratype), 84 mm, Venezuela, Amazonas, Río Orinoco, Río Cataniapo (~05°36’N, 67°36’W); ANSP 162127 (Paratype), 136 mm, Venezuela, Amazonas, Río Orinoco, Río Cunucunuma, (03°30’N, 65°56’W); FMNH 94776 (2), 76–114 mm, Colombia, Vaupes Pamopeta, Caño Ti; ANSP 162607, 105 mm, Venezuela, Amazonas, Río Orinoco, Río Casiquiare (03°05’N, 65°55’W); ANSP 162608, 89 mm, Venezuela, Amazonas, Río Orinoco, Río Casiquiare, Caño Caripo, (03°06’N, 65°50’W); FMNH 85592, 60 mm, Venezuela, Amazonas, Río Orinoco, near Puerto Ayacucho (~05°39’N, 67°37’W).

### Diagnosis

*Gymnotus (Tigrinus) stenoleucus* can be differentiated from all other members of the subgenus on the basis of the following characters: many scales above lateral line at midbody except when compared to *G*. *(T*.*) javari* (SAL 8–9 vs. 6–8 *G*. *(T*.*) coatesi*, 6–8 in *G*. *(T*.*) coropinae*).

### Description

Sexually monomorphic. Size up to 142 mm TL with adult body proportions attained at about 150 mm TL. Adult body shape subcylindrical with a mean ratio of body width to depth of 62.2%. Body profile slender, body depth 69.1–82.2% total length. Head length short, 9.7–11.1% total length. Snout length moderate, 29.3–30.8% head length. Mouth width narrow, 30.4–37.7% head length. Preanal distance long, 77.2–78.2% head length. Anal-fin long, 69.9–69.9% total length. Cycloid or ovoid scales present on entire post-cranial portion of body from nape to caudal appendage.

Scales above lateral line intermediate, in 8–9 rows. Gape large, extending to or beyond posterior nares. Mouth position superior, lower jaw longer than upper, rictus decurved. Chin round in lateral, dorsal profiles, fleshy and bulbous with mental electroreceptive organ overlying lower jaw. Anterior narial pore partially or entirely included within gape, in small narial fold. Anterior nares small, its diameter less than that of eye. Eye below horizontal with mouth. Circumorbital series ovoid. Premaxilla with >11 teeth disposed in two rows along outer margin, median margin curved. Maxilla-palatine articulation near tip of endopterygoid. Maxilla rod- or paddle-shaped, narrow distally with a straight ventral margin, length equal to 7–9 dentary teeth. Dentary with one row of >16 teeth, >5 needle-shaped anteriorly, all others conical posteriorly. Posterodorsal and posteroventral dentary processes abuts ventral. Dentary posteroventral process shorter than or almost as long as posterodorsal, narrow distally. Dentary ventral margin lamella short, depth less than posterior process. Dentary anteroventral margin with a hook. Anguloarticular process long, extending beyond ventral margin of dentary. Retroarticular with an arched lamella posteriorly forming a small canal, posterior margin squared. Endopterygoid superior and inferior portions approximately equal in size, ascending process robust, straight, tip simple. Interopercle dorsal margin ascending process present. Preopercle anteroventral notch present, posterodorsal laterosensory ramus with 1 superficial pore, margin of medial shelf smooth, median shelf small, less than half width of symplectic. Opercle dorsal margin straight, posterior margin smooth. Cranial fontanels closed in juveniles and adults. Frontal anterior margin round, postorbital process narrow, less than two times width of supraorbital canal. Lateral ethmoid unossified. Parietal rectangular, length equal to width. Adductor mandibula insertion undivided, intermusculars absent. All basibranchials unossified. Gill rakers not contacting gill bar.

Cleithrum very narrow with straight ventral margin, anterior limb long, greater than 1.8 times ascending limb, without facet for insertion of muscle from supracleithrum. Postcleithrum thin, discoid or sickle shaped. Body cavity intermediate, with 41–43 precaudal vertebrae. Rib 5 broad along its entire extent, greater than three times width of rib 6. Displaced hemal spines absent. Pectoral fin narrow, with 12–14 rays. Anal fin long, with 190–245 rays. Lateral-line complete. Lateral-line dorsal rami absent in adults. Single hypaxial electric organ, extending along entire ventral margin of body with 3 rows of electroplates near caudal insertion of anal fin.

## *Gymnotus* (*Tijax*), subgen. nov. (Table 5)

### Type species

*G*. *(T*.*) cylindricus*.

### Other included species

*G*. *(T*.*) maculosus*, *G*. *(T*.*) panamensis*.

### Diagnosis

*Gymnotus* (*Tijax*) is readily distinguishable from all other subgenera of the Gymnotinae by the following characters: a color pattern lacking dark pigment bands entirely, with small, irregular dark pigment spots 2–3 scales in diameter (except in *G*. *(T*.*) panamensis*, which possesses dark pigment bands only on the posterior 33% of the body), a sickle-shaped maxilla with a curved dorsal margin (shared only with *G*. *(L*.*) tiquie*), a short ascending process of the endopterygoid (shared only with *G*. *(G*.*) cuia* and *G*. *(G*.*) omarorum*), range of all species restricted to Central America (shared only with *G*. *(T*.*) henni*). *Tijax* is morphologically most similar to *Gymnotus*, from which is it readily distinguishable by the following characters: two rows of premaxillary teeth vs. one, small preopercular marginal shelf, less than half of the width of the symplectic vs. greater, prootic foramenae Vp combined with V2 & VII vs. separate from V2 & VII.

### Description

Sexually monomorphic. Size up to 236 mm TL with adult body proportions attained at about 150 mm TL. Adult body shape subcylindrical with a mean ratio of body width to depth of 66.4%. Body profile moderate, body depth 71.9–114.3% total length. Head length short, 9.1–11.6% total length. Snout length moderate, 34.0–41.5% head length. Mouth width narrow, 36.1–50.1% head length. Preanal distance long, 84.2–109.6% head length. Anal-fin long, 78.4–84.2% total length. Cycloid or ovoid scales present on entire post-cranial portion of body from nape to caudal appendage.

Scales above lateral line intermediate, in 7–11 rows. Gape large, extending to or beyond posterior nares. Mouth position superior, lower jaw longer than upper, rictus decurved. Chin round in lateral, dorsal profiles, fleshy and bulbous with mental electroreceptive organ overlying lower jaw. Anterior narial pore partially or entirely included within gape, in small narial fold. Anterior nares small, its diameter less than that of eye. Eye below horizontal with mouth. Circumorbital series ovoid. Premaxilla with <10 teeth disposed in two rows along outer margin, median margin curved. Maxilla-palatine articulation near tip of endopterygoid. Maxilla sickle-shaped, broad distally with a concave ventral margin, length equal to width of 4–6 dentary teeth. Dentary with one row of conical teeth. Posterodorsal and posteroventral dentary processes over ventral. Dentary posteroventral process shorter than or almost as long as posterodorsal, narrow distally. Dentary ventral margin lamella small, depth less than posterior process. Dentary anteroventral margin lacking a hook. Mandible short and compressed. Anguloarticular process long, extending beyond ventral margin of dentary. Retroarticular with an arched lamella posteriorly forming a small canal, posterior margin squared. Endopterygoid superior and inferior portions approximately equal in size, ascending process robust, straight, short, tip complex (except in *G*. *(T*.*) panamensis*). Interopercle dorsal margin ascending process present (except in *G*. *(T*.*) panamensis*). Dorsal region of hyomandibula with four lateral foramenae, supraorbital and infraorbital nerves connected. Preopercle anteroventral notch absent, posterodorsal laterosensory ramus with one superficial pore, margin of medial shelf entirely smooth, median shelf small, less than half width of symplectic. Opercle dorsal margin straight or convex, posterior margin entirely smooth. Subopercle dorsal margin concave (except in *G*. *(T*.*) panamensis*). Cranial fontanels closed in juveniles and adults. Frontal broad, anterior margin straight, postorbital process broad, greater than two times width of supraorbital canal (except in *G*. *(T*.*) panamensis*). Lateral ethmoid unossified. Parietal rectangular, length equal to width. Pterosphenoid anteroventral portion robust, extends ventrally to lateral margin of parasphenoid. Parasphenoid posterior processes gracile, elongate, posterior margin convex, deep. Prootic foramen Vp combined with V2-3+VII. All basibranchials unossified. Gill rakers not contacting gill bar.

Cleithrum broad with curved ventral margin, anterior limb short, less than 1.8 times ascending limb, lacking large facet for insertion of muscle from supracleithrum. Postcleithrum thin, discoid or sickle shaped. Body cavity of intermediate length, with 32–36 precaudal vertebrae. Rib 5 robust along its entire extent, greater than three times width of rib 6 (except in *G*. *(T*.*) panamensis*, which has a broad medial triangular shelf). Displaced hemal spines absent. Pectoral fin large, with 15–16 rays. Anal fin of moderate length, with 170–270 rays. Lateral-line dorsal rami absent in adults. Single hypaxial electric organ, extending along entire ventral margin of body with 3 rows of electroplates near caudal insertion of anal fin.

### Etymology

Subgenus name derived from the Mayan word (and astrological sign) *Tijax*, meaning “knife”, often made of obsidian. The Mayan civilization occupied Central America, to which the Gymnotine subgenus is endemic, for almost four thousand years and made numerous cultural and technological advancements, including the only pre-Columbian writing system developed in the Americas.

**Diagnoses and descriptions of each species of *Tijax* (Table 17)**

## *Gymnotus (Tijax) cylindricus* LaMonte

### Materials examined in morphological analyses

UMMZ 193986 (10), 98–185 mm, Guatemala, Quebrada de Vegega, Los Amates, Rio Izabal.

**Table 17:** Summary of morphometric and meristic data for three species of *Tijax*. Data for 22 specimens.

*** ***	***G*. *(T*.*) cylindricus***				***G*. *(T*.*) maculosus***				***G*. *(T*.*) panamensis***			
** **	**N**	**Min**	**Max**	**AVG**	**N**	**Min**	**Max**	**AVG**	**N**	**Min**	**Max**	**AVG**
**TL**	8	136	185	162	6	161	231	196	2	221	236	229
**HL**	8	14.2	19.9	17.3	6	17.1	21.1	18.9	2	21.2	21.5	21.4
**HL%**	8	9.9	11.6	10.6	6	9.1	10.6	9.7	2	9.1	9.6	9.4
**PR%**	8	37.1	41.5	39.8	5	34.2	40.7	37.4	2	34.0	37.3	35.6
**MW%**	8	36.1	45.2	41.9	6	36.1	40.8	38.8	2	41.8	50.1	46.0
**PO%**	8	58.9	64.5	61.9	6	59.1	65.6	62.8	2	61.4	63.7	62.6
**IO%**	8	43.5	48.1	46.0	6	42.9	48.5	45.7	2	36.7	41.3	39.0
**BD%**	8	101.4	114.3	105.9	6	84.0	112.2	100.8	2	71.9	81.6	76.7
**BW%**	8	64.5	79.3	71.6	6	62.9	85.7	75.7	2	56.6	62.6	59.6
**BW/BD**	7	0.0	73.0	57.0	5	70.3	79.2	74.9	2	76.7	78.7	77.7
**HD%**	8	68.3	75.6	72.2	5	65.3	71.3	69.1	2	62.2	62.3	62.3
**HW%**	8	63.8	72.3	69.4	6	69.1	72.2	70.6	2	64.9	66.5	65.7
**BO%**	8	23.5	42.0	34.3	6	36.8	48.5	42.8	2	40.2	53.1	46.7
**PA%**	8	87.6	100.5	94.9	6	94.8	109.6	102.7	2	84.2	94.7	89.4
**P1%**	8	36.8	43.1	41.1	6	35.5	44.5	40.3	2	39.3	42.7	41.0
**AF%**	6	78.4	82.2	80.0	5	78.6	81.4	79.9	2	81.4	84.2	82.8
** **	**N**	**Min**	**Max**	**Mode**	**N**	**Min**	**Max**	**Mode**	**N**	**Min**	**Max**	**Mode**
**BND**	10	0	0	0	10	0	0	0	2	23	24	24
**AFR**	5	190	200	190	4	170	200	200	2	269	270	270
**P1R**	_	_	_	_	_	_	_	_	2	15	16	16
**SAL**	2	11	11	11	2	7	7	7	2	7	8	8
**CEP**	_	_	_	_	_	_	_	_	2	3	3	3
**APS**	_	_	_	_	_	_	_	_	_	_	_	_
**PCV**	_	_	_	_	_	_	_	_	_	_	_	_
**PLR**	8	32	34	33	5	34	36	34	2	36	36	36
**PLL**	3	41	43	41	3	35	44	42	2	54	57	56
**VLR**	_	_	_	_	_	_	_	_	_	_	_	_

### Diagnosis

*Gymnotus* (*Tijax*) *cylindricus* can be differentiated from all other members of the subgenus on the basis of the following characters: color pattern lacking dark pigment bands, with dark pigment blotches absent except in some Costa Rican localities (vs. small dark pigment spots over entire body in *G*. *(T*.*) maculosus*, band pairs clearly visible on the posterior 33% of the body in *G*. *(T*.*) panamensis*); many scales above lateral-line (SAL 11, vs. 7 in *G*. *(T*.*) maculosus*, 7–8 in *G*. *(T*.*) panamensis*).

### Description

Sexually monomorphic. Size up to 185 mm TL with adult body proportions attained at about 150 mm TL. Adult body shape subcylindrical with a mean ratio of body width to depth of 57.0%. Body profile slender, body depth 101.4–114.3% total length. Head length short, 9.9–11.6% total length. Snout length moderate, 37.1–41.5% head length. Mouth width narrow, 36.1–45.2% head length. Preanal distance long, 87.6–100.5% head length. Anal-fin long, 78.4–82.2% total length. Cycloid or ovoid scales present on entire post-cranial portion of body from nape to caudal appendage.

Scales above lateral line intermediate, in 11 rows. Gape large, extending to or beyond posterior nares. Mouth position superior, lower jaw longer than upper, rictus decurved. Chin round in lateral, dorsal profiles, fleshy and bulbous with mental electroreceptive organ overlying lower jaw. Anterior narial pore partially or entirely included within gape, in small narial fold. Anterior nares small, its diameter less than that of eye. Eye below horizontal with mouth. Circumorbital series ovoid. Premaxilla with <10 teeth disposed in two rows along outer margin, median margin curved. Maxilla-palatine articulation near tip of endopterygoid. Maxilla sickle-shaped, narrow distally with a curved ventral margin, length equal to 4–6 dentary teeth. Dentary with one row of >16 teeth, all conical. Posterodorsal and posteroventral dentary processes abuts ventral. Dentary posteroventral process shorter than or almost as long as posterodorsal, narrow distally. Dentary ventral margin lamella small, depth less than posterior process. Dentary anteroventral margin without a hook. Anguloarticular process long, extending beyond ventral margin of dentary. Retroarticular with an arched lamella posteriorly forming a small canal, posterior margin squared. Endopterygoid superior and inferior portions approximately equal in size, ascending process robust, straight, tip complex. Interopercle dorsal margin ascending process present. Dorsal region of hyomandibula with four lateral foramenae, supraorbital and infraorbital nerves connected. Preopercle anteroventral notch absent, posterodorsal laterosensory ramus with 1 superficial pore, margin of medial shelf smooth, median shelf small, less than half width of symplectic. Opercle dorsal margin straight, posterior margin smooth. Cranial fontanels closed in juveniles and adults. Frontal anterior margin straight, postorbital process broad, greater than two times width of supraorbital canal. Lateral ethmoid unossified. Parietal rectangular, length equal to width. Pterophenoid anteroventral process robust, extends to lateral process. Parasphenoid posterior processes gracile. Prootic foramen Vp combined with V2-3+VII. Adductor mandibula insertion undivided, intermusculars absent. All basibranchials unossified. Gill rakers not contacting gill bar.

Cleithrum board with curved ventral margin, anterior limb short, less than 1.8 times ascending limb, without facet for insertion of muscle from supracleithrum. Postcleithrum thin, discoid or sickle shaped. Rib 5 robust along its entire extent, less than three times width of rib 6. Displaced hemal spines absent. Anal fin long, with 190–200 rays. Lateral-line complete. Lateral-line dorsal rami absent in adults. Single hypaxial electric organ, extending along entire ventral margin of body.

## *Gymnotus (Tijax) maculosus* Albert & Miller

### Materials examined in morphological analyses

UMMZ 197103 (10), 79–231 mm, Costa Rica, Santa Rosa, near Taxisco.

### Diagnosis

*Gymnotus* (*Tijax*) *maculosus* can be differentiated from all other members of the subgenus on the basis of the following characters: color pattern lacking dark pigment bands entirely, with small, irregular dark pigment blotches over entire body (vs. dark pigment blotches absent except in some Costa Rican localities in *G*. *(T*.*) cylindricus*, band pairs clearly visible on the posterior 33% of the body in *G*. *(T*.*) panamensis*); endocoracoid proximal portion thin (vs. broad in all other *Tijax*); endocoracoid distal portion not ossified (vs. ossified in all other *Tijax*).

### Description

Sexually monomorphic. Size up to 231 mm TL with adult body proportions attained at about 150 mm TL. Adult body shape subcylindrical with a mean ratio of body width to depth of 74.9%. Body profile slender, body depth 84.0–112.2% total length. Head length short, 9.1–10.6% total length. Snout length moderate, 34.2–40.7% head length. Mouth width narrow, 36.1–40.8% head length. Preanal distance long, 94.8–109.6% head length. Anal-fin long, 78.6–81.4% total length. Cycloid or ovoid scales present on entire post-cranial portion of body from nape to caudal appendage.

Scales above lateral line intermediate, in 7 rows. Gape large, extending to or beyond posterior nares. Mouth position superior, lower jaw longer than upper, rictus decurved. Chin round in lateral, dorsal profiles, fleshy and bulbous with mental electroreceptive organ overlying lower jaw. Anterior narial pore partially or entirely included within gape, in small narial fold. Anterior nares small, its diameter less than that of eye. Eye below horizontal with mouth. Circumorbital series ovoid. Premaxilla with <10 teeth disposed in two rows along outer margin, median margin curved. Maxilla-palatine articulation near tip of endopterygoid. Maxilla rod- or paddle-shaped, narrow distally with a broad ventral margin, length equal to 4–6 dentary teeth. Dentary with one row of >16 teeth, all conical. Posterodorsal and posteroventral dentary processes abuts ventral. Dentary posteroventral process shorter than or almost as long as posterodorsal, narrow distally. Dentary ventral margin lamella small, depth less than posterior process. Dentary anteroventral margin with a hook. Anguloarticular process long, extending beyond ventral margin of dentary. Retroarticular with an arched lamella posteriorly forming a small canal, posterior margin squared. Endopterygoid superior and inferior portions approximately equal in size, ascending process robust, straight, tip complex. Interopercle dorsal margin ascending process present. Dorsal region of hyomandibula with four lateral foramenae, supraorbital and infraorbital nerves connected. Preopercle anteroventral notch absent, posterodorsal laterosensory ramus with 1 superficial pore, margin of medial shelf smooth, median shelf small, less than half width of symplectic. Opercle dorsal margin convex, posterior margin smooth. Cranial fontanels closed in juveniles and adults. Frontal anterior margin straight, postorbital process broad, greater than two times width of supraorbital canal. Lateral ethmoid unossified. Parietal rectangular, length equal to width. Pterophenoid anteroventral process robust, extends to lateral process. Parasphenoid posterior processes gracile. Prootic foramen Vp combined with V2-3+VII. Adductor mandibula insertion undivided, intermusculars absent. All basibranchials unossified. Gill rakers not contacting gill bar.

Cleithrum broad with curved ventral margin, anterior limb short, less than 1.8 times ascending limb, without facet for insertion of muscle from supracleithrum. Postcleithrum thin, discoid or sickle shaped. Rib 5 robust along its entire extent, less than three times width of rib 6. Displaced hemal spines absent. Anal fin long, with 170–200 rays. Lateral-line complete. Lateral-line dorsal rami absent in adults. Single hypaxial electric organ, extending along entire ventral margin of body.

## *Gymnotus (Tijax) panamensis* Albert & Crampton

### Materials examined in morphological analyses

CAS 72209 (Holotype), 236 mm, Panama, Bocas del Toro, small creek into the Río Cricamola, near Konkitu (08°59’N, 81°55’W); Paratype: CAS 217109 (Paratype), 221 mm, same locality as CAS 72209.

### Diagnosis

*Gymnotus* (*Tijax*) *panamensis* can be differentiated from all other members of the subgenus on the basis of the following characters: color pattern with band pairs clearly visible on the posterior 33% of the body (vs. lacking dark pigment bands, with dark pigment blotches absent except in some Costa Rican localities in *G*. *(T*.*) cylindricus*, lacking dark pigment bands entirely, with small, irregular dark pigment blotches over entire body in *G*. *(T*.*) maculosus*); many pored lateral-line scales anterior to the first ventral lateral-line ramus (PLR 54–57 vs. 41–43 in *G*. *(T*.*) cylindricus*, 35–44 in *G*. *(T*.*) maculosus*); long anal fin (AFR 269–207, vs. 190–200 in *G*. *(T*.*) cylindricus*, 170–200 in *G*. *(T*.*) maculosus*); tip of ascending process of endopterygoid simple (vs. complex in all other *Tijax*); metapterygoid superior portion ossified to anterior margin of inferorbital (vs. not reaching anterior margin of inferorbital in all other *Tijax*); interopercle ascending process absent (vs. present in all other *Tijax*); subopercle dorsal margin convex (vs. concave in all other *Tijax*); hyomandibular posterior lateral-line canal not contacting posterior margin (vs. contacting posterior margin in all other *Tijax*); frontal postorbital process narrow (<2X width of supraorbital, vs. >2x width od supraorbital in all other *Tijax*).

### Description

Sexually monomorphic. Size up to 236 mm TL with adult body proportions attained at about 150 mm TL. Adult body shape subcylindrical with a mean ratio of body width to depth of 77.7%. Body profile slender, body depth 71.9–81.6% total length. Head length short, 9.1–9.6% total length. Snout length moderate, 34.0–37.3% head length. Mouth width narrow, 41.8–50.1% head length. Preanal distance long, 84.2–94.7% head length. Anal-fin long, 81.4–84.2% total length. Cycloid or ovoid scales present on entire post-cranial portion of body from nape to caudal appendage.

Scales above lateral line intermediate, in 7–8 rows. Gape large, extending to or beyond posterior nares. Mouth position superior, lower jaw longer than upper, rictus decurved. Chin round in lateral, dorsal profiles, fleshy and bulbous with mental electroreceptive organ overlying lower jaw. Anterior narial pore partially or entirely included within gape, in small narial fold. Anterior nares small, its diameter less than that of eye. Eye below horizontal with mouth. Circumorbital series ovoid. Premaxilla with <10 teeth disposed in two rows along outer margin, median margin curved. Maxilla-palatine articulation near tip of endopterygoid. Maxilla sickle-shaped, narrow distally with a concave ventral margin, length equal to 4–6 dentary teeth. Dentary with one row of >16 teeth, all conical. Posterodorsal and posteroventral dentary processes abuts ventral. Dentary posteroventral process shorter than or almost as long as posterodorsal, narrow distally. Dentary ventral margin lamella small, depth less than posterior process. Dentary anteroventral margin without a hook. Anguloarticular process long, extending beyond ventral margin of dentary. Retroarticular with an arched lamella posteriorly forming a small canal, posterior margin squared. Endopterygoid superior and inferior portions approximately equal in size, ascending process robust, straight, tip simple. Interopercle dorsal margin ascending process absent. Dorsal region of hyomandibula with four lateral foramenae, supraorbital and infraorbital nerves connected. Preopercle anteroventral notch absent, posterodorsal laterosensory ramus with 1 superficial pore, margin of medial shelf smooth, median shelf small, less than half width of symplectic. Opercle dorsal margin straight, posterior margin smooth. Cranial fontanels closed in juveniles and adults. Frontal anterior margin straight, postorbital process narrow, less than two times width of supraorbital canal. Lateral ethmoid unossified. Parietal rectangular, length equal to width. Pterophenoid anteroventral process robust, extends to lateral process. Parasphenoid posterior processes gracile. Prootic foramen Vp combined with V2-3+VII. Adductor mandibula insertion undivided, intermusculars absent. All basibranchials unossified. Gill rakers not contacting gill bar.

Cleithrum broad with curved ventral margin, anterior limb short, less than 1.8 times ascending limb, without facet for insertion of muscle from supracleithrum. Postcleithrum thin, discoid or sickle shaped. Rib 5 broad along its entire extent, greater than three times width of rib 6. Displaced hemal spines absent. Pectoral fin broad, with 15–16 rays. Anal fin long, with 269–270 rays. Lateral-line complete. Lateral-line dorsal rami absent in adults. Single hypaxial electric organ, extending along entire ventral margin of body with 3 rows of electroplates near caudal insertion of anal fin.

## Keys to all subgenera and species of Gymnotinae

Here we present a dichotomous taxonomic key to the six new subgenera of the Gymnotinae, plus a further six keys to the species of those genera. These keys are meant to facilitate identification of these fishes by observers with some background knowledge on Neotropical fishes but who are not necessarily experts on the Gymnotiformes, such as other working ichthyologists or experienced collectors and curators. Therefore, the keys focus on readily-observable aspects of color pattern, external morphology and meristics rather than osteology, which is treated in depth in the diagnoses and descriptions above. We also include geographic ranges in rare cases where readily-observable external characters are unavailable, but the species possess well-documented, circumscribed ranges.

### Key to the subgenera of Gymnotinae

**1A:** color pattern lacking obliquely-oriented dark pigment bands or band pairs **… 2**

**1B:** color pattern consisting of obliquely-oriented dark pigment bands or band pairs (restricted to tail in *G*. *(G*.*) bahianus*, absent in *G*. *(T*.*) esmeraldas*, *G*. *(G*.*) diamantinensis* and *G*. *onca*) … **3**

**2A:** color pattern consisting of irregular dark color blotches with blurry, low contrast margins; geographic range restricted to Southeastern Brazil … *Gymnotus* (*Pantherus*)

**2B:** color pattern lacking bands or band pairs (except in *G*. *(T*.*) panamensis*); geographic range restricted to Central America … *Gymnotus* (*Tijax*)

**3A:** oral teeth small and needle-shaped; single laterosensory canal pore in dorsoposterior portion of preopercle … **4**

**3B:** oral teeth either robust and conical or anteroposteriorly compressed; two laterosensory canal pores in dorsoposterior portion of preopercle (except *G*. *(G*.*) curupira*) … **5**

**4A:** pigment bands with wavy, irregular margins (except in *G*. *(L*.*) pedanopterus*); larger adult body sizes (> 250 mm TL) … *Gymnotus* (*Lamontianus*)

**4B:** pigment bands with straight, regular margins; smaller adult body sizes (< 250 mm TL) … *Gymnotus* (*Tigrinus*)

**5A:** head color evenly counter-shaded with no white blotches; anteroposteriorly compressed first 2–9 dentary teeth, resembling arrowheads; some elongate and obliquely oriented posterior ventral lateral-line rami; all scales ovoid; clear or white pigment patch at posterior end of anal-fin membrane (anal-fin all black in in *G*. *(G*.*) arapaima*, many *G*. *(G*.*) carapo*, *G*. *(G*.*) sylvius* and *G*. *(G*.*) ucamara*); juveniles with broad black patch on anal-fin at posterior mid-body … *Gymnotus* (*Gymnotus*)

**5B:** white blotches with high-contrast margins on head (except in adult *G*. *(T*.*) inaequilabiatus*); teeth robust and conical, occasionally decurved with outwards-pointing tips in large (TL >300 mm TL) specimens; many and straight ventral lateral-line rami; scales on posterior 20% of body axially elongate; longitudinal stripes at posterior end of anal-fin membrane; juveniles with no black patch on anal fin … *Gymnotus* (*Tigre*)

### Key to the species of subgenus *Gymnotus* (*Gymnotus*)

**1A:** dark bands or band pairs with pale interbands present … **2**

**1B:** dark bands or band pairs absent or highly irregular in adults … **3**

**2A:** pale interbands rarely extend above lateral line on anterior 50% of body … **4**

**2B:** dark band pairs and pale interbands extend to middorsum … **9**

**3A:** dark band pairs present over entire body in juveniles and subadults (TL <250 mm), but absent anteriorly in large adults (TL >350 mm); endemic to the Rio Magdalena Basin of Colombia … *G*. *(G*.*) ardilai*

**3B:** band pairs replaced by dark colored blotches or spots anteriorly in adults and some juveniles … **5**

**4A:** long head (HL 11.1–13.3% TL) and short body cavity (PCV 32–35); endemic to the Río Atrato basin and Pacific slope of Colombia … *G*. *(G*.*) choco*

**4B:** short head (HL 7.2–11.7% TL) and long body cavity (PCV 36–38) … **6**

**5A:** dark band pairs replaced by intermediately-sized (3–4 scales), evenly spaced brown blotches … *G*. *(G*.*) diamantinensis*

**5B:** dark band pairs replaced by small (1–2 scales), rounded black spots over anterodorsal portion of body … **7**

**6A:** two preopercular-mandibular sensory canal pores in the dorsoposterior portion of the preopercle; endemic the Eastern Amazon basin near the estuary, Pará state, Brazil… *G*. *(G*.*) capanema*

**6B:** one preopercular-mandibular sensory canal pores in the dorsoposterior portion of the preopercle in most juveniles (TL <250 mm) and many mature specimens … **8**

**7A:** small (1–2 scales), rounded black spots over entire body of most specimens, except for posteroventral 25% of some; short interorbital distance (IO 33.9–42.5% HL) and few scales above lateral line (SAL 7) … *G*. *(G*.*) bahianus*

**7B:** small (1–2 scales), rounded black spots above lateral line and band pairs highly fragmented and intermingled below lateral line on all specimens; long interorbital distance (IO 44.6–45.9% HL) and many scales above lateral line (SAL 9) … *G*. *(G*.*) interruptus*

**8A:** wide mouth (MW 42.0–50.7% HL), wide body (BW 71.6–76.7% HL), many pectoral-fin rays (P1R 14–18), few anal-fin pterygiophore scales (APS 7–8) and long body cavity (PCV 37–38); endemic to the greater La Plata (Paraguay-Paraná) basin in the Southern humid Neotropics … *G*. *(G*.*) pantanal*

**8B:** narrow mouth (MW 32.7–45.7% HL), narrow body (BW 53.3–72.0% HL), few pectoral-fin rays (P1R 10–13), few anal-fin pterygiophore scales (APS 10–11) and short body cavity (PCV 36); endemic to the upper Madeira basin of Peru and Bolivia … *G*. *(G*.*) riberalta*

**9A:** dark band pairs branched ventrally to form inverted *Y*-shaped patterns in adults … *G*. *(G*.*) curupira*

**9B:** dark band pairs never branched ventrally, not fragmented or overlapping ventrally … **10**

**10A:** pale interbands narrow, roughly 25% width of dark band pairs … **11**

**10B:** pale interbands intermediate to wide, 50%–100% width of dark band pairs … **12**

**11A:** long head (HL 12.2–13.4% TL) … *G*. *(G*.*) ucamara*

**11B:** short head (HL 8.9–12.4% TL) … **13**

**12A:** reverse countershading anterodorsally with a metallic blue or gunmetal colored sheen visible on live and recently-preserved specimens; especially long (HL 12.1–14.2% TL) and slender (HD 50.7–60.5% HL) head … *G*. *(G*.*) arapaima*

**12B:** lacking reverse countershading or metallic sheen; head length rarely exceeding 12.0% TL and head depth rarely less than 60% HL … **14**

**13A:** many pectoral-fin rays (P1R 20–22), many pored lateral-line scales prior to the first ventral lateral-line ramus (PLR 50–56) and many pored lateral-line scales (PLL 108–132) … *G*. *(G*.*) obscurus*

**13B:** few pectoral-fin rays (P1R 10–16), few pored lateral-line scales prior to the first ventral lateral-line ramus (PLR 30–49) and few pored lateral-line scales (PLL 62–100) … **15**

**14A:** anal fin clear or lightly pigmented; anteriormost two to four teeth on either side of dentary anteroposteriorly compressed, resembling arrowheads; small to intermediate adult body size (TL 170–280 mm) … **16**

**14B:** anal fin dark with clear patch posteriorly adults; anteriormost five or more teeth on either side of dentary anteroposteriorly compressed, resembling arrowheads; large adult body size (TL 300+ mm) … **17**

**15A:** few pectoral-fin rays (P1R 10–13) and few ventral lateral-line rami (VLR 10–15) … *G*. *(G*.*) eyra*

**15B:** many pectoral-fin rays (P1R 12–16) and few ventral lateral-line rami (VLR 16–18) … *G*. *(G*.*) mamiraua*

**16A:** 17–29 dark band pairs, two–three of the anteriormost five pale interbands with both margins crescent-shaped, bending outward; endemic to the Fitzcarrald region of southeastern Peru … *G*. *(G*.*) chaviro*

**16B:** 14–22 dark band pairs, all with parallel margins; endemic to the central Amazon basin … *G*. *(G*.*) varzea*

**17A:** long head (HL 10.7–15.5% TL), slender head (HD 50.8–72.4% HL) and slender body (BD 42.7–106.6% HL) … **18**

**17B:** short head (HL 9.2–12.4% TL), deep head (HD 61.9–80.2% HL) and deep body (BD 68.3–133.3% HL) … **19**

**18A:** pale interbands 33–50% width of dark band pairs; … *G*. *(G*.*) carapo*

**18B:** pale interbands roughly equal in width to dark band pairs … *G*. *(G*.*) sylvius*

**19A:** few pored lateral-line scales anterior to the first ventral lateral-line ramus (PLR 23–30) and many ventral lateral-line rami (VLR 28–30) … *G*. *(G*.*) omarorum*

**19B:** many pored lateral-line scales anterior to the first ventral lateral-line ramus (PLR 30–47) and few ventral lateral-line rami (VLR 13–28) … **20**

**20A:** many band pairs (BND 29–49) with band pairs often separated into single bands or replaced by irregular blotches over some portions of body … *G*. *(G*.*) chimarrao*

**20B:** few band pairs (BND 21–29) with band pairs remaining regular and not fragmented over entire body of most specimens … *G*. *(G*.*) cuia*

### Key to the species of subgenus *Gymnotus* (*Lamontianus*)

**1A:** narrow mouth (MW 32.6–37.7% HL), short interorbital distance (IO 23.6–32.2% HL), slender body (BW 32.6–47.9% HL), short pectoral fins (P1 26.6–39.8% HL) and short body cavity (PCV 31–23) … *G*. *(L*.*) pedanopterus*

**1B:** wide mouth (MW 41.4–58.3% HL), broad interorbital distance (IO 41.2–51.3% HL), wide body (BW 59.9–76.3% HL), long pectoral fins (P1 29.3–54.9% HL) and long body cavity (PCV 37–51) … **2**

**2A:** dark brown band pairs absent or heavily obscured above lateral line in anterior 60% of body; short body cavity (PCV 37–38) and few pored lateral-line scales prior to the first ventral lateral-line ramus (PLR 58–62) … *G*. *(L*.*) anguillaris*

**2B:** dark brown band pairs present throughout body; long body cavity (PCV 45–51) and many pored lateral-line scales prior to the first ventral lateral-line ramus (PLR 60–78) … **3**

**3A:** wide mouth (MW 42.2–58.3% HL), short interorbital distance (IO 41.2–51.3% HL), slender head (HD 59.6–66.7% HL), narrow head (HW 62.1–73.7% HL) and short preanal distance (PA 96.4–109.6% HL); especially long body cavity (PCV 53) … **4**

**3B:** narrow mouth (MW 32.0–40.5% HL), long interorbital distance (IO 31.9–36.2% HL), deep head (HD 52.7–59.5% HL), wide head (HW 49.3–61.2% HL) and long preanal distance (PA 68.6–94.4% HL); intermediate body cavity (PCV 45–51) … **5**

**4A:** 23–25 dark brown band pairs with wavy, irregular margins; short preorbital distance (PR 36.0–37.0% HL), narrow mouth (MW 42.2–44.4% HL), short interorbital distance (IO 41.2–41.3% HL), narrow head (HW 62.1–62.7% HW) and short pectoral fins (P1 29.3–41.5% HL); few pored lateral-line scales prior to the first ventral lateral-line ramus (PLR 60–65) and a long body cavity (PCV 47–51) … *G*. *(L*.*) cataniapo*

**4B:** 19–24 dark brown band pairs with wavy, irregular margins; long preorbital distance (PR 37.3–39.7% HL), wide mouth (MW 50.0–58.3% HL), long interorbital distance (IO 42.8–51.3% HL), wide head (HW 66.7–73.7% HW) and long pectoral fins (P1 47.1–54.9% HL); many pored lateral-line scales prior to the first ventral lateral-line ramus (PLR 69–78) and a short body cavity (PCV 45) … *G*. *(L*.*) tiquie*

**5A:** few pectoral-fin rays (P1R 9–12), few scales over the anal-fin pterygiophores (APS 5–8) and long body cavity (PCV 54–58) … *G*. *(L*.*) n*. *sp*. *‘ARAP’*

**5B:** many pectoral-fin rays (P1R 13–14), many scales over the anal-fin pterygiophores (APS 9–10) and short body cavity (PCV 53) … *G*. *(L*.*) n*. *sp*. *‘ARIP’*

Key to the species of subgenus *Gymnotus* (*Pantherus*)

**1A:** irregular dark color blotches with blurry, low contrast margins and a pair of white blotches on ventral portion of the head, anterior to or directly below eye … *G*. (*P*.) *capitimaculatus*

**1B:** irregular dark color blotches with blurry, low contrast margins with evenly pigmented head, lacking any blotches… **2**

**2A:** slender head (HL 57.0–70.5% TL); endemic to coastal rivers of Santa Catarina, Paraná, São Paulo, Rio de Janeiro and Bahia states, Brazil … *G*. (*P*.) *pantherinus*

**2B:** deep head (HL 60.6–73.3% TL); endemic to coastal rivers of Rio Grande do Sul and Santa Catarina states, Brazil … *G*. (*P*.) *refugio*

Key to the species of subgenus *Gymnotus* (*Tigre*)

**1A:** large adult size (max. 998 mm TL); color pattern consisting of obliquely oriented, dark brown band pairs with wavy, irregular margins in juveniles (TL <250 mm), replaced in adults by large, irregular dark brown blotches on a pale brown ground color … *G*. (*T*.) *inaequilabiatus*

**1B:** adult size rarely exceeds 500 mm TL; obliquely oriented, dark brown band pairs with wavy, irregular margins in all specimens (except *G*. (*T*.) *esmeraldas*) … **2**

**2A:** short body cavity (PCV 32–35) and many ventral lateral-line rami (VLR 49–55) … *G*. (*T*.) *paraguensis*

**2B:** long body cavity (PCV 41–54) and few ventral lateral-line rami (VLR 23–38) … **3**

**3A:** dark band pairs absent over anterior 80% of body, replaced by irregular pale blotches; endemic to trans-Andean rivers of Ecuador … *G*. (*T*.) *esmeraldas*

**3B:** obliquely oriented, dark brown band pairs with wavy, irregular margins … **4**

**4A:** many dark pigment band pairs (BND 16–23); narrow mouth (MW 40.4–43.8% HL) and long head (HL 11.0–13.3% TL); endemic to cis-Andean South America … *G*. (*T*.) *tigre*

**4B:** few dark pigment band pairs (BND 13–15); wide mouth (MW 43.2–49.4% HL) and short head (HL 9.2–10.7% TL); endemic to trans-Andean South America … *G*. (*T*.) *henni*

### Key to the species of subgenus *Gymnotus* (*Tigrinus*)

**1A:** band pairs replaced by large, irregular dark brown blotches over entire body … *G*. (*T*.) *onca*

**1B:** band pairs present … **2**

**2A:** pale interbands rarely extend above lateral line on anterior 50% of body (except in upper Madeira populations) … *G*. (*T*.) *coropinae*

**2B:** pale interbands extend vertically past lateral line and meet at middorsum … **3**

**3A:** 11 unpaired, straight dark bands, roughly equal in width to pale interbands … *G*. (*T*.) *melanopleura*

**3B:** 13–24 dark band pairs, greater in width than pale interbands … **4**

**4A:** pale interbands wider dorsally on anterior 50% of body, up to 50% width of dark band pairs (vs. 25% in ventral portion of same band) … *G*. (*T*.) *coatesi*

**4B:** margins of bands roughly parallel … **5**

**5A:** narrow pale interbands (25% width of dark band pairs) anteriorly and 5–8 partially divided, often *H*-shaped dark band pairs posteriorly … *G*. (*T*.) *javari*

**5B:** color pattern occasionally variable, but always consisting of narrow pale interbands (~25% width of dark band pairs) over entire body, dark band pairs infrequently partially divided and never *H*-shaped … **6**

**6A:** 15–17 dark band pairs; endemic to the Amazon basin … *G*. (*T*.) *jonasi*

**6B:** 18–23 dark band pairs; endemic to the Orinoco basin … *G*. (*T*.) *stenoleucus*

### Key to the species of subgenus *Gymnotus* (*Tijax*)

**1A:** band pairs only clearly visible on the posterior 33% of the body, obscured by small (1–2 scales wide), irregular pale blotches anteriorly … *G*. (*T*.) *panamensis*

**1B:** band pairs absent entirely, with small, irregular dark pigment blotches … **2**

**2A:** dark pigment blotches absent except in some Costa Rican localities; many scales above lateral line (SAL 11); distributed in Atlantic drainages of Central America … *G*. (*T*.) *cylindricus*

**2B:** small dark pigment spots located over entire body; few scales above lateral line (SAL 7); distributed in Pacific drainages of Central America … *G*. (*T*.) *maculosus*

## Supporting information

S1 Supplementary Material All sequence clusters identified for Gymnotus (NCBI:txid36670) from GenBank release 230 (February 15, 2019, available here: https://www.ncbi.nlm.nih.gov/genbank/release/230/) using the PhyLota Browser. (ZIP)

S2 Supplementary Material The sequence alignment used in all molecular analyses, plus a table of all Genbank accession numbers for sequences drawn from Tagliacollo et. al., (2016). (ZIP)

S3 Supplementary Material The character matrix used in all morphological analyses, plus descriptions of character states corresponding to each character in the matrix. (ZIP)

S4 Supplementary Material Bayesian phylogeny constructed using only morphological data. Nodes color coded to indicate support, with darker circles representing higher support. (ZIP)

S5 Supplementary Material Bayesian phylogeny constructed using only molecular data. Nodes color coded to indicate support, with darker circles representing higher support. (ZIP)

S6 Supplementary Material Maximum-likelihood phylogeny constructed using only molecular data present in S2 Supplementary Material. (ZIP)

S7 Supplementary Material Maximum parsimony phylogeny constructed using only morphological data present in S3 Supplementary Material. (ZIP)

S8 Supplementary Material Time-calibrated phylogeny recovered from the Bayesian analysis. Includes labels of all samples. N = 211 specimens representing 48 (45 Gymnotinae, one Electrophorinae, two outgroups) species.
(ZIP)

S9 Supplementary Material Phylogeny recovered from the Maximum-Likelihood analysis. Includes labels of all samples. N = 211 specimens representing 48 (45 Gymnotinae, one Electrophorinae, two outgroup) species. (ZIP)
