# Supplementary material for: Phylogenetic revision of Gymnotidae (Teleostei: Gymnotiformes), with descriptions of six subgenera
Source: PLoS One. 2019 Nov 7;14(11):e0224599. doi: 10.1371/journal.pone.0224599 (PMC6837465; doi:10.1371/journal.pone.0224599)
Supplement: S3 Supplementary Material — (ZIP) [file pone.0224599.s003.zip › S3 - Character States.docx]

| **Character Type** | **Character** | **State 0** | **State 1** | **Stae 2** | **State 3** | **State 4** |
| --- | --- | --- | --- | --- | --- | --- |
| Pigmentation | Band orientation | no bands | oblique |  |  |  |
| Pigmentation | Band spacing at midbody | no bands | even distance between, irregular widths | irregular distance between, even widths | even distances and widths | irrregular distances and widths |
| Pigmentation | Band margin shape | no bands | wavy | straight |  |  |
| Pigmentation | Band margin contrast | no bands | low/blurry | high/sharp |  |  |
| Pigmentation | Band pigment density | no bands | dark bands paired, pale in middle | dark bands evenly pigmented | retic. in abdom. region | retic. in sup. region |
| Pigmentation | Dark bands above lateral line | all continuous on anterior half of body | most broken or absent in adults | no bands | absent anteriorly |  |
| Pigmentation | Band (pair) width at midbody | no bands | dark bands 2-3X broader than pale | dark bands 4-5X broader than pale | dark bands narrower than pale |  |
| Pigmentation | Band pair number (BND) | median 0-16 | median 17-29 |  |  |  |
| Pigmentation | Head color pattern | not blotched | blotched |  |  |  |
| Pigmentation | Ground color middorsum at midbody | dark | light |  |  |  |
| Pigmentation | White cheek patch | absent | present |  |  |  |
| Pigmentation | Nape pale yellow patch | absent | present |  |  |  |
| Pigmentation | Anal fin posterior clear patch | absent | present |  |  |  |
| Pigmentation | Anal fin color | anterior half hyalin to brown | anterior half black |  |  |  |
| Pigmentation | Anal fin posterior stripes | absent from posterior region | present in posterior region |  |  |  |
| Pigmentation | Body squamation | present | absent |  |  |  |
| Morphological | Body size (small) | more than 160 mm max TL | less than 160 mm max TL |  |  |  |
| Morphological | Body size (large) | less than 999 mm max TL | more than 1 m max TL |  |  |  |
| Morphological | Body profile (BD) | deep: mean BD = 9.1-11.7% TL | slender: mean BD = 6.1-9.0% TL |  |  |  |
| Morphological | Head length (HL) | short: mean HL = 8.4-9.4% TL | moderate: mean HL =9.5-11.0% TL | long: mean HL = 11.1-13.5% TL |  |  |
| Morphological | Head depth (HD) | deep: mean HD = 66-75% HL | moderate: mean HD = 60-65% HL | shallow: mean HD = 53-59% HL |  |  |
| Morphological | Head width (HW) | wide: mean HW = 66-74% HL | moderate: mean HW = 57-65% HL | narrow: mean HW = 51-56% HL |  |  |
| Morphological | Snout length (PR) | short: mean PR = 28-33% HL | moderate: mean PR = 34-36% HL | long: mean PR = 37-40% HL |  |  |
| Morphological | Mouth width (MW) | narrow: mean MW = 27-35% HL | moderate: mean MW 36-44% HL | wide: mean MW = 45-56% HL |  |  |
| Morphological | Interorbital distance (IO) | narrow: mean IO = 26-35% HL | broad: IO = mean 36-40% HL | very broad: mean IO = 41-55% HL |  |  |
| Morphological | Branchial opening (BO) | broad: mean BO = 33-44% HL | narrow: mean BO = 25-32% HL |  |  |  |
| Morphological | Preanal distance (PA) | long: mean PA = 90-128% HL | short: mean PA 56-89% HL | very long: mean PA 129-155% |  |  |
| Morphological | Pectoral fin length (P1) | long: mean P1 = 45-58% HL | short: mean P1 34-44% HL |  |  |  |
| Morphological | Anal-fin length (AF) | long: mean AF = 76-83% TL | short: mean AF = 67-75% TL |  |  |  |
| Morphological | Body shape (BW/BD) | laterally compressed: mean BW/BD = 58-73% HL | cylindrical: mean BW/BD = 74-90% HL |  |  |  |
| Morphological | Gape size in mature specimens | small: ant. to post. nares | large: at or post. to post. nares |  |  |  |
| Meristic | Scales above lateral line (SAL) | small: mode 9-13 | large: mode 5-8 |  |  |  |
| Meristic | Scale shape in mature specimens | cycloid or slightly ovoid | elongate: length >1.5X depth |  |  |  |
| Meristic | Posterior lateral line scales (PLL) | median <110 | median >110 |  |  |  |
| Meristic | Scales to first VLR (PLR) | median 0-49 | median 50-61 | median 62-70 |  |  |
| Meristic | Scales over AF pterygiophores (APS) | large: mode 5-7 | moderate: mode 8-11 | small: mode 12-16 rows |  |  |
| Meristic | Lateral line ventral rami (VLR) | absent | median 1-8 long VLRs | median 9-24 long VLRs | median >25 short VLRs |  |
| Meristic | Lateral line pores | every scale | stitches of 3-4 |  |  |  |
| Meristic | Lateral line dorsal rami (DLR) | absent | present |  |  |  |
| Meristic | Body cavity length (PCV) | mode 17-22 | mode 23-29 | mode 30-35 | mode 36-39 | mode >40 |
| Meristic | Anal-fin rays (AFR) | mean 150-225 | mean >226 |  |  |  |
| Meristic | Anal-fin pterygiophore length | shorter than hemal spines | equal to or longer than hemal spines |  |  |  |
| Meristic | Hypaxial electric organs | 1 | 3 |  |  |  |
| Meristic | Caudal electroplate rows (CEP) | mode 2-3 | mode 4 | mode 5-6 |  |  |
| Osteological | Mouth position | terminal: rictus horizontal | superior: rictus decurved |  |  |  |
| Osteological | Eye position | above horzontal of mouth | below horizontal of mouth |  |  |  |
| Osteological | Mesethmoid anterior margin | convex, rounded or straight | concave with paired anteriolateral procs. |  |  |  |
| Osteological | Mesethmoid neck | narrow: <2X lat. proc. | broad: 3-4X lat. proc. |  |  |  |
| Osteological | Anterior narial pore | flush with head surface | pipe-shaped, partially or entirely in gape |  |  |  |
| Osteological | Circumorbial series | ovoid | tear-drop shaped |  |  |  |
| Osteological | Maxilla-palatine position | near tip of mesopterygoid | post. to tip of mesepterygoid |  |  |  |
| Osteological | Maxilla orientation | horizontal | vertical |  |  |  |
| Osteological | Maxilla shape | sickle-shaped with concave dorsal margin | rod- or paddle-shaped with straight dorsal margin | triangular with concave dorsal margin |  |  |
| Osteological | Maxilla length | = width of 4-6 dent. teeth | = width of 7-9 dent. teeth | = width of >10 dent. teeth |  |  |
| Osteological | Maxilla end shape | paddle-shaped, broad distally | rod-shaped, narrow distally |  |  |  |
| Osteological | Premaxilla teeth in outer row | many: >11 | few: <10 |  |  |  |
| Osteological | Premaxilla shape | Curved median margin | Straight median margin |  |  |  |
| Osteological | Premaxilla tooth rows | one row on outer margin | two rows or anterior patch |  |  |  |
| Osteological | Dentary needle-shaped teeth | all conical or flattened | >5 needle shaped |  |  |  |
| Osteological | Dentary arrowhead-shaped teeth | all conical or needle-shaped | 2-4 arrowhead-shaped | 4-7 arrowhead-shaped | >8 arrowhead-shaped |  |
| Osteological | Dentary teeth in outer row | mode 0-12 | mode 12-15 | >16 |  |  |
| Osteological | Dentary tooth rows | one row in outer margin | inner row anteriorly |  |  |  |
| Osteological | Dentary post. procs. | dorsal over ventral | dorsal abutts ventral |  |  |  |
| Osteological | Dentary vent. post. proc. | shorter than dorsopost. proc. | almost as long as dorsopost. proc. |  |  |  |
| Osteological | Dentary dorsopost. proc. | narrow distally | broad distally |  |  |  |
| Osteological | Dentary vent. margin | lamella small: <post. proc. | lamella large: >post. proc. |  |  |  |
| Osteological | Dentary ant. hook | absent in lat. view | present in lat. view |  |  |  |
| Osteological | Operculum dorsal margin | straight or convex | concave |  |  |  |
| Osteological | Operculum post. margin | entirely smooth | ridges or spines |  |  |  |
| Osteological | Preopercle laterosensory pore | dorsopost. pore single | dorsopost. pore double |  |  |  |
| Osteological | Preopercle anterior notch | absent | present |  |  |  |
| Osteological | Preopercle shelf margin | entirely smooth | serrate |  |  |  |
| Osteological | Preopercle median shelf | small: <half width of symplectic | large: >half width of symplectic |  |  |  |
| Osteological | Mesopterygoid ascending proc. base | robust | gracile |  |  |  |
| Osteological | Mesopterygoid ascending proc. length | short: base >length | long: base <length |  |  |  |
| Osteological | Mesopterygoid asecending proc. shape | straight | curved |  |  |  |
| Osteological | Mesopterygoid asecending proc. tip | simple | complex |  |  |  |
| Osteological | Metapterygoid sup. portion | ossifies to ant. margin of infer. | ossifies less than ant. margin of infer. |  |  |  |
| Osteological | Interopercle asecending proc. | present | absent |  |  |  |
| Osteological | Subopercle dorsal margin | concave | convex |  |  |  |
| Osteological | Retroarticular posterior margin | squared | pointed |  |  |  |
| Osteological | Angulo-articular ventrolat. lamellae | absent or small | extending over retroarticular |  |  |  |
| Osteological | Angulo-articular proc. | long: beyond vent. margin of dentary | short: to vent. margin of dentary |  |  |  |
| Osteological | Mandible shape | short, compressed | long, extended |  |  |  |
| Osteological | Hyomandibular trigeminal canals | SO and IO connected | SO and IO divided |  |  |  |
| Osteological | Hyomandibular PLL canal | not contacting post. margin | contacting post. margin |  |  |  |
| Osteological | Cranial fontanels | open | closed |  |  |  |
| Osteological | Frontal ant. margin | straight | rounded |  |  |  |
| Osteological | Frontal postorbital process | narrow: <2X width of SO | broad: >2X width of SO |  |  |  |
| Osteological | Frontal shape | broad: width at LF4 = 0.25-0.35 | narrow: width at LF4 = 0.20-0.24 |  |  |  |
| Osteological | Lateral ethmoid | ossified | unossified or absent |  |  |  |
| Osteological | Parasphenoid shape | elongate: length >3X width | narrow: length 2.2-3X width | broad: length <2.2X width |  |  |
| Osteological | Vomer | short: <half distance to PaS lat. proc. | long: >half distance to PaS lat. proc. |  |  |  |
| Osteological | Parasphenoid post. proc. | robust, stout, post. margin convex, shallow | gracile, elongate, post. margin convex, deep |  |  |  |
| Osteological | Parietal shape | square, length subequal to width | rectangular length, width |  |  |  |
| Osteological | Pterosphenoid anteroventral proc. | robust, extends vent. to lat. proc. | reduced, extends dorsal to lat. proc. |  |  |  |
| Osteological | Prootic foramenae | Vp separate from V2-3+VII | Vp combined with V2-3+VII |  |  |  |
| Osteological | Adductor mandibula insertion | only to maxilla | also to 1st infraorbital | undivided |  |  |
| Osteological | Adductor mandibula intermuscular | absent | ossified |  |  |  |
| Osteological | Basibranchials | ossified | unossified |  |  |  |
| Osteological | Gill rakers | contacting gill bar | not contacting gill bar |  |  |  |
| Osteological | Pectoral-fin rays (P1R) | mode = 17-21 | mode = 14-16 | mode = 12-13 |  |  |
| Osteological | Pectoral fin medial radial | large | small |  |  |  |
| Osteological | Mesocoracoid prox. portion | thin | broad |  |  |  |
| Osteological | Mesocoracoid dist. portion | ossified | not ossified |  |  |  |
| Osteological | Postcleithra | robust | thin, discoid or sickle-shaped |  |  |  |
| Osteological | Cleithrum shape | broad, ventral margin curved | narrow, ventral margin straight | very narrow, ventral margin straight |  |  |
| Osteological | Cleithrum ant. limb length | short: 1.5X length of ascending limb | long: >1.8X of ascending limb |  |  |  |
| Osteological | Cleithrum ant. notch | absent | present | semi-lunar |  |  |
| Osteological | Cleithrum dorsopost. facet | absent or small | large |  |  |  |
| Osteological | Rib 5 shape | robust along entire extent, <3X width of rib 6 | broad medial triangular shelf >3X width of rib 6 |  |  |  |
| Osteological | Hemal spines | present | absent |  |  |  |
| Osteological | Displaced hemal spines | absent | present |  |  |  |
| Osteological | Anal-fin ray branching | 10-17 branched AFR | >18 branched AFR | 0 branched AFR |  |  |
| Electric Organ Discharge | EOD phases from baseline in adults | monophasic | biphasic | triphasic | tetraphasic or more |  |
