# Supplementary figures and images for: Phylogenetic revision of Gymnotidae (Teleostei: Gymnotiformes), with descriptions of six subgenera
Source: PLoS One. 2019 Nov 7;14(11):e0224599. doi: 10.1371/journal.pone.0224599 (PMC6837465; doi:10.1371/journal.pone.0224599)

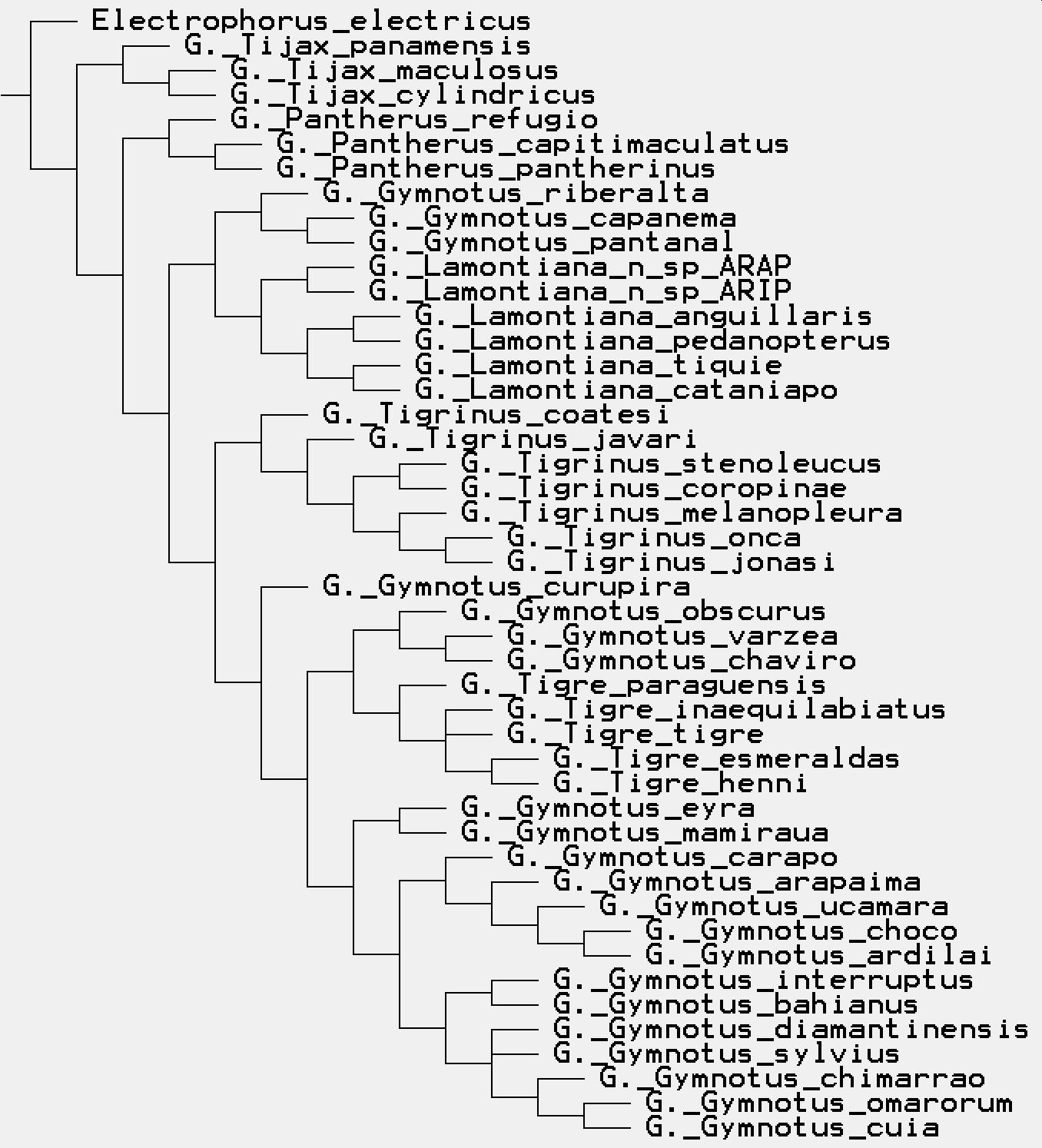

Supplement: S7 Supplementary Material — (ZIP) [file pone.0224599.s007.zip › S7 - Parsimony Morphological Phylogeny TNT Output.jpg]
